# Abietane Diterpenes from *Medusantha martiusii* and Their Anti-Neuroinflammatory Activity

**DOI:** 10.3390/molecules29122723

**Published:** 2024-06-07

**Authors:** Edileuza B. de Assis, Rodrigo S. de Andrade, Joanda P. R. e Silva, Lucas H. Martorano, Geraldo M. W. Amorim, Paulo B. A. Loureiro, Lucas S. Abreu, Marianna V. Sobral, Marcus T. Scotti, Fernando M. dos Santos Junior, Maria de Fátima Agra, Josean F. Tavares, Marcelo S. da Silva

**Affiliations:** 1 Postgraduate Program in Natural and Synthetic Bioactive Products, Federal University of Paraiba, João Pessoa 58051-900, Brazil; edileuzabezerra@ltf.ufpb.br (E.B.d.A.); rodrigo@ltf.ufpb.br (R.S.d.A.); joandapaolla.1@gmail.com (J.P.R.e.S.); moiseswand@ltf.ufpb.br (G.M.W.A.); paulobrunoaloureiro@ltf.ufpb.br (P.B.A.L.); mariannavbs@gmail.com (M.V.S.); mtscotti@ccae.ufpb.br (M.T.S.); agramf@ltf.ufpb.br (M.d.F.A.); 2 Department of Organic Chemistry, Fluminense Federal University, Niterói 24020-141, Brazil; lucashm@id.uff.br (L.H.M.); abreu_lucas@id.uff.br (L.S.A.); fernando_martins@id.uff.br (F.M.d.S.J.)

**Keywords:** *Medusantha martiusii*, Caatinga, diterpenes, aromatic abietane, neurodegenerative diseases, TNF-α

## Abstract

Seven new abietane diterpenoids, comprising medusanthol A–G (**1**–**3**, **5**, **7**–**9**) and two previously identified analogs (**4** and **6**), were isolated from the hexane extract of the aerial parts of *Medusantha martiusii.* The structures of the compounds were elucidated by HRESIMS, 1D/2D NMR spectroscopic data, IR spectroscopy, NMR calculations with DP4+ probability analysis, and ECD calculations. The anti-neuroinflammatory potential of compounds **1**–**7** was evaluated by determining their ability to inhibit the production of nitric oxide (NO) and the proinflammatory cytokine TNF-α in BV2 microglia stimulated with LPS and IFN-γ. Compounds **1**–**4** and **7** exhibited decreased NO levels at a concentration of 12.5 µM. Compound **1** demonstrated strong activity with an IC_50_ of 3.12 µM, and compound **2** had an IC_50_ of 15.53 µM; both compounds effectively reduced NO levels compared to the positive control quercetin (IC_50_ 11.8 µM). Additionally, both compounds significantly decreased TNF-α levels, indicating their potential as promising anti-neuroinflammatory agents.

## 1. Introduction

The Lamiaceae family, which consists of plants and shrubs, is composed of approximately 258 genera and 7193 species. In Brazil, over 500 species from the Lamiaceae family are distributed across 46 genera, with nearly half of these species belonging to the subfamily Nepetoideae [1,2], which is a well-known source of abietane-type diterpenoids [3,4]. The broad spectrum of biological activities associated with these compounds has garnered special attention, with many demonstrating anti-inflammatory [5], anticancer [6], antimicrobial [7], and antiprotozoal [8] properties.

*Medusantha martiusii* (Benth.) Harley and J. F. B. Pastore (syn: *Hyptis martiusii*), commonly known as “cidreira brava” or “cidreira-do-campo,” is a shrub native and endemic to Brazil and belongs to the subfamily Nepetoideae. This species is predominantly found in the Northeast region, specifically in the Caatinga, a unique semiarid biome exclusive to Brazil. In traditional Brazilian medicine, the infusion or decoction of *M. martiusii* leaves is used to combat intestinal and stomach disorders, while the decoction of roots is commonly used to combat inflammation of the ovaries [9,10]. Previous phytochemical investigations of *M. martiusii* have documented the isolation of abietane diterpenes from its roots and aerial parts, as well as the phytochemical profiling of its essential oil and its pharmacological activities [11,12,13,14]. However, to the best of our knowledge, there is no evidence in the literature with respect to the anti-inflammatory activity of the isolated compounds. As part of our investigation into species from the Brazilian semiarid region, we conducted a chemical reinvestigation of *M. martiusii*, which led to the isolation of nine abietane diterpenes, including seven previously undescribed compounds named medusanthol A–G (**1**–**3**, **5**, **7**–**9**) and two known analogs (**4** and **6**). Here, we describe the isolation, structural elucidation, and anti-neuroinflammatory activity of these isolates.

## 2. Results and Discussion

### 2.1. Structure Elucidation of the Compounds

**Figure 1:**
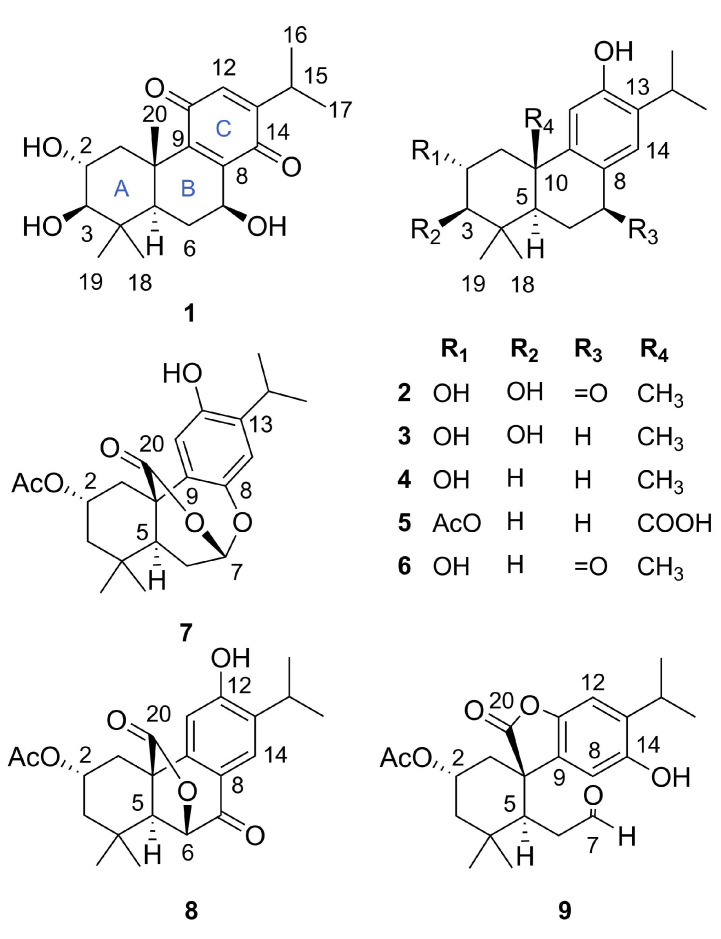
Chemical structures of the isolated abietanes **1**–**9**.

The hexanic extract of the aerial parts was fractionated into six fractions by vacuum liquid chromatography. The EtOAc fraction was purified using HPLC, yielding seven previously unknown (**1**–**3**, **5**, **7**–**9**) and two known (**4** and **6**) abietanes (Figure 1).

**Figure 2:**
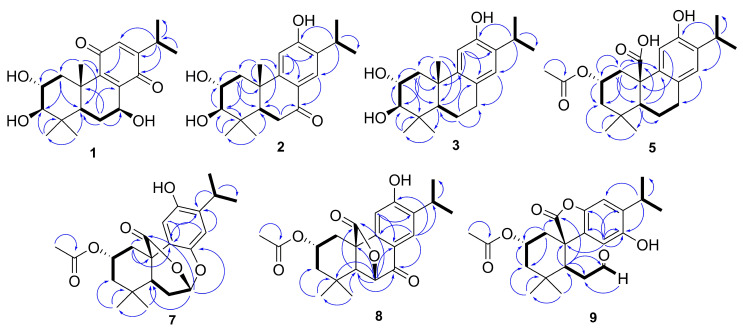
Key ^1^H–^1^H COSY (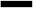) and HMBC (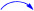) of compounds **1**–**3**, **5,** and **7**–**9**.

Compound **1** was isolated as an amorphous brown powder and assigned the molecular formula C_20_H_28_O_5_ based on its HRESIMS ion at *m*/*z* 331.1896 ([M − H_2_O + H]^+^, calcd for C_20_H_27_O_4_, 331.1904, Δ = 2.2 ppm), corresponding to seven degrees of hydrogen deficiency. The IR spectrum displayed absorption bands for hydroxy (3363 cm^−1^) and conjugated keto carbonyl (1706 and 1645 cm^−1^) groups. The ^13^C NMR (Table 1) spectrum exhibited resonances for 20 carbons, including signals for two conjugated carbonyls at *δ*_C_ 188.0 and 190.1 and four olefinic carbons (three non-hydrogenated at *δ*_C_ 141.9, 150.6, and 153.6, and one hydrogenated at *δ*_C_ 132.4), consistent with the presence of a *para*-benzoquinone unit [15]. With the aid of the HSQC experiment, the remaining signals were assigned to five methyl carbons (*δ*_C_ 21.5, 21.4, 28.8, 17.0, 21.1), two methylene carbons (*δ*_C_ 42.3, 26.1), five methine carbons (including three *sp*^3^ oxymethine at *δ*_C_ 82.8, 68.8, and 67.9 and one nonoxygenated *sp*^3^ methine at *δ*_C_ 48.3), and two quaternary carbons (*δ*_C_ 38.9, 40.0). An analysis of ^1^H NMR data (Table 2) revealed the presence of an isopropyl group connected to *para*-benzoquinone through the signals of a methine hydrogen at *δ*_H_ 2.97 (1H, m, H-15) and two methyl groups at *δ*_H_ 1.10 and 1.08 (6H, s, H_3_-16/H_3_-17) [12]. Similarly, signals for three oxymethine hydrogens at *δ*_H_ 4.79 (1H, dd, *J* = 10.2, 7.5 Hz, H-7), *δ*_H_ 3.01 (1H, d, *J* = 9.6 Hz, H-3), and *δ*_H_ 3.82 (1H, ddd, *J* = 11.5, 9.6, 4.4 Hz, H-2) were observed, as shown in Table 1. Based on the HSQC spectrum, the signal at *δ*_H_ 6.36 (1H, d, *J* = 1.2 Hz, H-12) was attributed to the olefinic hydrogen of the *para*-benzoquinone unit, the signals for methylene hydrogens at *δ*_H_ 1.15 (1H, m) and 3.06 (1H, dd, *J* = 12.7, 4.4 Hz) were assigned to H_2_-1, and *δ*_H_ 2.20 (1H, m) and 1.64 (1H, m) were assigned to H_2_-6. The aforementioned evidence suggests that compound **1** is an abietane quinone. In the HMBC spectrum, the correlations of the signal at *δ*_H_ 1.40 (3H, s) with the carbons at *δ*_C_ 42.3, 48.3, and 150.6 defined the methyl group CH_3_-20 and the chemical shifts of carbons C-1, C-5, and C-9, respectively (Figure 2). 

Furthermore, the correlations of the signals at *δ*_H_ 1.07 and 0.91 (6H, s, H_3_-18/H_3_-19) with the carbon signals at *δ*_C_ 38.9, 82.8, and 48.3 identified the two geminal methyl groups linked to C-4 and defined the chemical shifts of carbons C-4, C-3, and C-5, respectively. The presence of vicinal hydroxyl groups at C-2 and C-3 in the A ring of compound **1** was supported by the HSQC correlations of the oxymethine proton at *δ*_H_ 3.01 (1H, d, *J* = 9.6 Hz, H-3) with carbon at *δ*_C_ 82.8 (C-3), as well as the spin system H_2_-1(*δ*_H_ 3.01)/H-2/H-3 observed in the COSY spectrum. The correlation between the signals at *δ*_H_ 4.79 (1H, dd, *J* = 10.2, 7.5 Hz, H-7) and *δ*_C_ 67.9 (C-7) in the HSQC spectrum suggested the presence of a third hydroxyl group at C-7. The correlation of H-5/H_2_-6/H-7 in the COSY spectrum established the location of the hydroxyl group at this position. The correlations of the methine proton H-12 (*δ*_H_ 6.36, d, *J* = 1.2 Hz) with C-9, C-14, and C-15 and H-15 (*δ*_H_ 2.97, m) with C-12, C-14, C-16, and C-17 in the HMBC spectrum substantiated the attachment of the isopropyl group to *para*-benzoquinone and suggested that the quaternary carbon at δC 188.0 was linked to C-11. 

**Figure 3:**
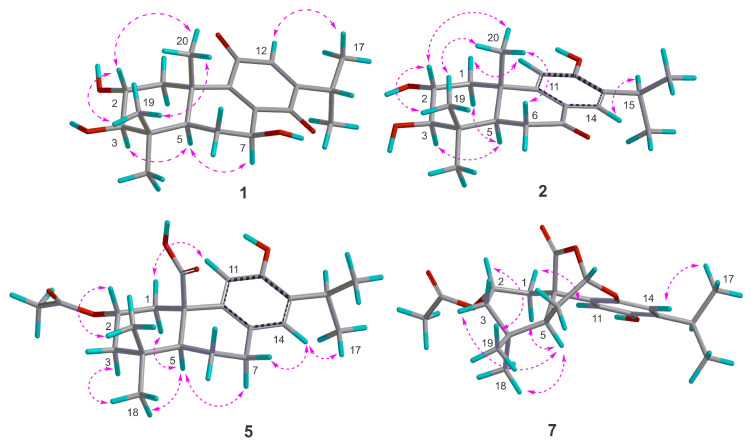
Key ^1^H–^1^H NOESY (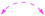) of compounds **1**, **2**, **5,** and **7**.

**Figure 4:**
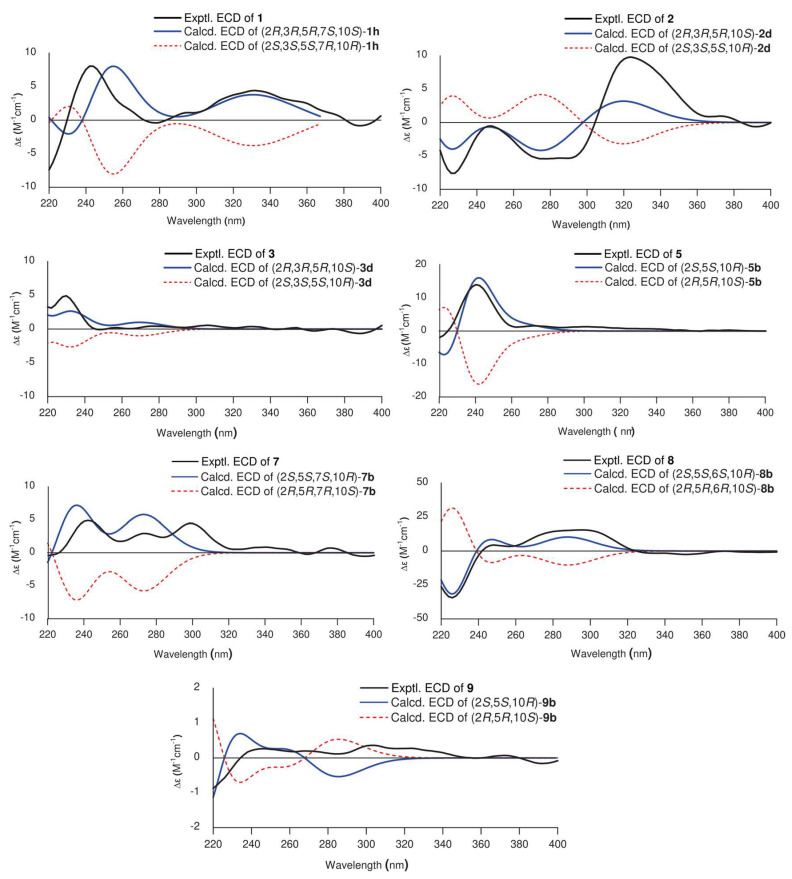
Comparison of experimental and calculated ECD curves of compounds **1**–**3**, **5**, and **7**–**9**.

Based on biosynthetic considerations and chemotaxonomic data, this study provides support for the connection between transfused A/B rings in abietanes of the genus *Medusantha*, with CH_3_-20 *β*-axial and H-5 *α*-axial rings [11,12,13,16,17,18]. The relative configuration of compound **1** was determined through NOESY correlations and coupling constant analysis (Figure 3). The NOESY correlation with H-2/H_3_-19/H_3_-20 confirmed the cofacial arrangement of these protons, confirming their *β* orientation. Furthermore, a coupling constant of 9.6 Hz, consistent with an approximate dihedral angle of 168° between H-2 (ddd, *J* = 11.5, 9.6, 4.4 Hz) and H-3 (d, *J* = 9.6 Hz), supported the proposition of a *trans*-diaxial orientation of these protons, indicating an *α*-axial orientation for H-3. The NOESY correlation of H-5/H-7 confirmed the *β* orientation of the 7-OH. To corroborate the proposed relative configuration for C-2, C-3, and C-7, ^1^H and ^13^C NMR data for eight isomers (1a–1h) were calculated using the gauge including atomic orbital (GIAO) method at the GIAO-mPW1PW91/6-31+G(d,p) level and then subjected to DP4+ probability analysis. The isomer (2R*,3R*,5R*,7S*,10S*)–1h exhibited a DP4+ probability of 100% (Figure S150). The absolute configuration was determined by comparing the experimental and calculated ECD data and was assigned as 2*R*,3*R*,5*R*,7*S*,10*S* (Figure 4). Thus, compound **1** was identified as a new abietane named medusanthol A.

Compound **2** was obtained as a white amorphous powder and exhibited a molecular formula of C_20_H_28_O_4_ with seven degrees of hydrogen deficiency, as determined by its HRESIMS peak at *m*/*z* 687.3863 [2M + Na]^+^ (calcd for C_40_H_56_NaO_8_, 687.3867, Δ = 0.6 ppm). The IR spectrum showed bands attributed to hydroxyl groups (3448 cm^−1^), conjugated keto carbonyl groups (1658 cm^−1^), and aromatic rings (1595 and 1460 cm^−1^). The ^13^C NMR spectrum exhibited 20 carbon signals (Table 1), including resonances assigned to one conjugated keto carbonyl carbon (*δ*_C_ 200.6), six aromatic carbons (two *sp*^2^ methine carbons at *δ*_C_ 110.6 and 127.3), two *sp*^3^ methine carbons (*δ*_C_ 50.3 and 27.9), and five methyl groups, as shown in Table 1. In the ^1^H NMR spectrum, a septet at *δ*_H_ 3.22 (1H, *J* = 6.8 Hz, H-15) and two doublet methyl groups at *δ*_H_ 1.19 (3H, d, *J* = 6.8 Hz, H-16) and 1.21 (3H, d, *J* = 6.8 Hz, H-17) suggested the presence of an isopropyl group characteristic of the diterpene abietane. Furthermore, the singlets at *δ*_H_ 6.76 (1H, H-11) and 7.80 (1H, H-14) were assigned to the *para*-aromatic hydrogens of the tetrasubstituted C ring (Table 2). 

Analysis of the NMR data of compound **2** indicated a close resemblance to **6**, identified as 2α-hydroxysugiol, a recognized aromatic abietane [19]. The only distinction between the two compounds was the replacement of a methylene carbon signal at *δ*_C_ 50.4 in C-3 with an oxymethine carbon at *δ*_C_ 83.5, suggesting the presence of an additional hydroxyl group at this position in compound **2**. In the HMBC spectrum, the correlation between *δ*_H_ 1.06 and 0.98 (6H, s, H_3_-18/H_3_-19) and C-3 (*δ*_C_ 83.5) confirmed the presence of the proposed connectivity (Figure 2). Similar to medusanthol A (**1**), compound **2** also contained vicinal hydroxyl groups attached to C-2 (*δ*_C_ 69.4) and C-3 (*δ*_C_ 83.5). Additionally, the correlation between the signal at *δ*H 7.80 (1H, s, H-14) and the carbon at *δ*_C_ 200.6 in the HMBC spectrum confirmed the insertion of the carbonyl group at C-7. The NOESY correlations were consistent with the same relative configuration as medusanthol A (**1**). The NOESY correlations of H-2/H_3_-19/H_3_-20 and H-3/H-5, as well as the coupling constant *^3^J*_H-2/H-3_ = 9.2 Hz, suggested that H-2/H_3_-19/H_3_-20 were *β*-oriented, while H-3/H-5 adopted the *α* orientation (Figure 3). NMR shift calculations and DP4+ probability analysis supported the relative configuration assigned, with 100% probability ascribed to the 2R*,3R*,5R*,10S*–2b isomer (Figure S153). The absolute configuration was determined by comparing the experimental and calculated ECD data, and the products were assigned as 2*R*,3*R*,5*R*,10*S* (Figure 4). Accordingly, compound **2** was designated medusanthol B.

Compound **3**, a needle crystal, was shown to have a molecular formula of C_20_H_30_O_2_ according to its HRESIMS peak at *m*/*z* 659.4276 ([2M + Na]^+^, calcd for C_40_H_60_NaO_6_, 659.4282, Δ = 0.9 ppm), indicating six degrees of hydrogen deficiency. In the IR spectrum, characteristic absorption bands were observed for a hydroxyl group (3361 cm^−1^) and an aromatic ring (1618 and 1425 cm^−1^). The 1D and 2D NMR data revealed that compound **3** is also an aromatic abietane, displaying significant structural similarity to medusanthol B (**2**), except for the substitution of the carbonyl group at *δ*_C_ 200.6 for the methylene carbon at *δ*_C_ 31.1 in C-7 (Table 1). In the ^1^H NMR spectrum, the shielding of the aromatic proton H-14 (*δ*_H_ 6.75, 1H, s) compared to that of medusanthol B (**2**) (Table 2), along with the cross-peak of H-14 with C-7 (*δ*_C_ 31.1) in the HMBC spectrum, supported the absence of a carbonyl group at C-7 (Figure 2). 

Further comprehensive analysis of the NMR data revealed that compound **3** shares an identical planar structure with 2,3-dihydroxyferruginol, which was isolated from the leaves of *Podocarpus nagi* [20]. The only distinction between these compounds is observed in the configuration at the C-2 center, suggesting a potential stereoisomer. In the NOESY spectrum, correlations between H-2 and H_3_-19/H_3_-20, along with those between H-3 and H-5/H_3_-18, allowed the determination of the *α* orientation of the 2-OH in compound **3**, which contrasts with the *β* orientation reported for this group in 2,3-dihydroxyferruginol. Furthermore, the 9.6 Hz coupling constant between H-2 and H-3 in compound **3** is distinct from the 2.9 Hz observed for these protons in 2,3-dihydroxyferruginol, further substantiating the aforementioned proposition. NMR calculations and DP4+ analyses supported the relative configuration of **3** as 2R*,3R*,5R*,10S*–3b with a probability of 100% (Figure S156). Ultimately, the absolute configuration was determined to be 2*R*,3*R*,5*R,*10*S* by comparing the experimental and calculated ECD data (Figure 4), suggesting that **3** is an epimer of 2,3-dihydroxyferruginol. Biogenetically, the configuration of **3** is proposed to be the same as that of **1** and **2**. Therefore, compound **3** was identified as a new abietane named medusanthol C.

Compound **5**, a white amorphous powder, exhibited a molecular formula of C_22_H_30_NaO_5_ (*m*/*z* 397.1974 [M + Na]^+^, calcd for C_22_H_30_NaO_5_, 397.1985, Δ = 2.8 ppm), suggesting the presence of an aromatic abietane with eight degrees of hydrogen deficiency. The infrared spectrum displayed characteristic absorptions at 3431 cm^−1^ (hydroxyl), 1735 and 1269 cm^−1^ (ester), 1710 cm^−1^ (carboxylic acid), and 1658, 1510, and 1421 cm^−1^ (aromatic ring). The ^13^C NMR data of the compound indicated a significant resemblance to medusanthol C (**3**) (Table 1). However, the presence of a single oxygenation on ring A for compound **5** was suggested by the replacement of the oxymethine carbon at *δ*_C_ 84.3 (C-3) in medusanthol C with a methylene carbon at *δ*_C_ 47.7. Furthermore, the 1D NMR spectrum revealed the deshielding of the oxymethine proton H-2 (*δ*_H_ 5.42, 1H, tt, *J* = 11.7, 4.5 Hz), as well as the presence of characteristic signals for an acetoxy group at *δ*_H_ 2.02 (3H, s), *δ*_C_ 21.4, and *δ*_C_ 172.6 (Table 1 and Table 2). This finding was consistent with the presence of a 2-OCOCH_3_ group in **5**, similar to miltiorin A, which is isolated from the roots of *Salvia miltiorrhiza* [21]. In the HMBC spectrum, the correlation between the signals at *δ*_H_ 1.02 and 0.93 (6H, s, H_3_-18/H_3_-19) and the signal at *δ*_C_ 47.7 assigned to C-3 confirmed the absence of a hydroxyl group at this position (Figure 2). According to the ^13^C NMR spectrum, compound **5** also differed from compound **3** in that it displayed signals for only four methyl groups, indicating the absence of a signal corresponding to CH_3_-20, as observed for compound **3** (*δ*_C_ 26.3). Therefore, the presence of a signal at *δ*_C_ 179.0 was attributed to C-20, indicating oxidation to a carboxylic acid at this position. The HMBC correlations of H-1 (*δ*_H_ 3.09, 1H, ddd, *J* = 12.1, 4.5, 2.8 Hz) with C-2 (*δ*_C_ 70.8) and C-20 (*δ*_C_ 179.0) confirmed the localization of the acetoxy and carboxylic acid functionalities, respectively (Figure 2).

The relative configuration of **5** was proposed by the NOESY correlations. The NOESY cross peaks of H-2/H_3_-19, H-1(*δ*_H_ 3.09)/H_3_-19, H-1(*δ*_H_ 3.09)/H-11 and H-5/H_3_-18 indicated that H-2 and CH_3_-19 were *β*-oriented, whereas CH_3_-18 and H-5 were *α*-oriented (Figure 3). NMR calculations and DP4+ analysis confirmed that the relative stereochemistry of the C-2 center was 2S*, with a probability of 100% (Figure S162). The absolute configuration of compound **5** was determined by ECD analysis. The experimental ECD spectrum matched well with the calculated curve (Figure 4), defined as (2*S*,5*S*,10*R*). Thus, **5** was designated medusanthol D.

Compound **7** was obtained as a white amorphous powder with a molecular formula of C_22_H_28_O_6_, as determined by its HRESIMS peak at *m*/*z* 799.3652 [2M + Na]^+^ (calculated for C_44_H_56_NaO_12_, 799.3664, Δ = 1.5 ppm), implying nine degrees of hydrogen deficiency. The IR spectrum displayed characteristic bands for hydroxyl (3446 cm^−1^) and lactone (1741 cm^−1^) groups. The 1D and 2D NMR data of **7** showed similarities to those of medusanthol D (**5**), with an acetoxy group at C-2, a tetra-substituted aromatic ring, and four methyl groups in its structure, as shown in Table 1. However, differences between the two compounds were also detected. The ^1^H NMR and HSQC spectra of compound **7** revealed an extra oxymethine signal at *δ*_H_ 5.94 (1H, d, *J* = 6.5 Hz) and correlations for only three methylene hydrogen groups. Similarly, the HMBC correlation of the signal at *δ*_H_ 1.85 (1H, t, *J* = 11.7 Hz, H-1) with the carbon at *δ*_C_ 170.9 suggested oxidation at the C-20 position, consistent with a lactone carbonyl [22] (Figure 2). In particular, the data of **7** notably differed from those of **5** due to the presence of a carbon at *δ*_C_ 95.4 and the deshielding of C-8 (Δ*δ*_C_ + 17.1 ppm) (Table 1).

Moreover, the correlation between *δ*_H_ 5.94 and *δ*_C_ 95.4 in the HSQC spectrum, along with the spin system H-5/H_2_-6/H-7 as determined by the COSY spectrum, provided substantial evidence for the presence of an acetal group at C-7. The aforementioned data, along with the HMBC correlations of the acetalic hydrogen H-7 (*δ*_H_ 5.94, d, *J* = 6.5 Hz) and resonances at *δ*_C_ 50.6 (C-5), *δ*_C_ 145.8 (C-8), and *δ*_C_ 170.9 (C-20), established the C-20-O-C-7 and C-7-O-C-8 connections, confirming a δ-lactone ring between C-20 and C-7 and an acetal functional group at C-7 with ring closure via C-8 (Figure 2).

The relative stereochemistry of compound **7** was deduced from NOESY correlations, similar to those observed for medusanthol E (**5**). The cross-peaks between H-2/H_3_-19 and H-5/H_3_-18 in the NOESY spectrum suggested that H-2 and H_3_-19 adopted a *β* orientation, while CH_3_-18 and H-5 assumed an *α* orientation (Figure 3). To further determine the relative configuration of compound **7**, the NMR data of two candidates (7a and 7b) were calculated. DP4+ analyses indicated that (2S*,5S*,7S*,10R*)–7b was highly likely at 99.81% (Figure S168). Furthermore, the absolute configuration of **7** was determined to be 2*S*,5*S*,7*S*,10*R* through comparison of the experimental and calculated ECD spectra (Figure 4). Ultimately, **7** was denominated medusanthol E.

Compound **8** was isolated as a white amorphous powder with the molecular formula C_22_H_26_O_6_, as deduced from its HRESIMS signal at *m*/*z* 409.1612 [M + Na]^+^ (calcd for C_22_H_26_NaO_6_, 409.1622, Δ = 2.3 ppm), corresponding to ten degrees of hydrogen deficiency. In the IR spectrum, characteristic absorption bands for hydroxyl (3446 cm^−1^), lactonic (1786 cm^−1^), ester (1720 cm^−1^), and conjugated ketone (1695 cm^−1^) groups were observed. The ^13^C NMR spectrum of **8** showed signals between *δ*_C_ 189.1 and 111.0, similar to those observed for compound **2**, which were attributed to the aromatic carbons of the C ring and the carbonyl of the ketone at C-7 (Table 1). On the other hand, compound **8** also exhibited structural similarities to compound **7**, as evidenced by the signals detected at *δ*_C_ 176.0 and *δ*_C_ 66.4, suggesting the presence of a lactone group at C-20 and an acetoxy group at C-2, respectively. In addition, the signals at *δ*_C_ 22.3, 22.4, 22.7, and 31.4, corresponding to the four methyl groups, also align with those found in compound **7**. However, the absence of signals at *δ*_C_ 95.4 and 145.8 and the presence of a signal at *δ*_C_ 81.4 in the ^13^C NMR spectrum of **8** suggested the formation of a lactonic ring via C-20 and C-6, supporting the keto carbonyl at C-7 (*δ*_C_ 189.1). The shielding of the oxymethylene hydrogen signal from *δ*_H_ 5.94 (1H, d, *J* = 6.5 Hz, H-7) in **7** to *δ*_H_ 4.77 (1H, s, H-6) in conjunction with the signal at *δ*_C_ 81.4 (C-6) in the HSQC spectrum was consistent with the proposed lactonization of **8**. The HMBC correlations of H-6 (*δ*_H_ 4.77) with signals at *δ*_C_ 34.0 (C-4), *δ*_C_ 49.2 (C-10), *δ*_C_ 121.8 (C-8), and *δ*_C_ 176.0 (C-20) confirmed that the bridge between C-20 and C-6 formed a *γ*-lactone ring (Figure 2).

The relative stereochemistry of C-6 was determined by analyzing the dihedral angle between singlets H-5 (*δ*_H_ 2.41, 1H) and H-6 (*δ*_H_ 4.77, 1H). These protons displayed an approximately 90° dihedral angle, indicating a pseudoequatorial arrangement for H-6, while H-5 exhibited an *α*-axial disposition. However, the relative configuration of the C-2 chiral center could not be conclusively determined by NOESY. In this way, the quantum GIAO method was utilized to calculate the ^13^C and ^1^H NMR chemical shifts of two potential isomers, (2R*,5S*,6S*,10R*)–8a and (2S*,5S*,6S*,10R*)–8b. Subsequently, comparison of these computed values with experimental data through DP4+ probability analysis indicated that the most likely relative configuration was (2S*,5S*,6S*,10R*)–8b, with a 76.92% probability (Figure S171). To determine the absolute configuration, the calculated and experimental ECD data were compared (Figure 4). The calculated ECD spectrum of 8b aligned closely with the experimental curve for **8**, suggesting the absolute configuration of 2*S*,5*S*,6*S*,10*R*. Ultimately, its structure was denoted as medusanthol F.

Compound **9** was obtained as a yellow amorphous powder, with an HRESIMS peak at *m*/*z* 799.3637 [2M + Na]^+^ (calcd for C_44_H_56_NaO_12_, 799.3664, Δ = 3.4 ppm), indicating a molecular formula of C_22_H_28_O_6_ and suggesting nine degrees of hydrogen deficiency. The infrared spectrum exhibited absorption bands for hydroxyl (3427 cm^−1^), lactonic (1791 cm^−1^), and aldehydic (1724 cm^−1^) groups. ^13^C NMR analysis of compound **9** indicated similar chemical shifts in the A ring to those of compounds **5**–**8**. However, differences in the chemical shifts of the B and C rings were observed compared to those of compounds **1**–**8** identified in this study (Table 1). According to the ^13^C NMR and DEPT spectra of compound **9**, six aromatic carbons were identified, including two oxygenated carbons at *δ*_C_ 150.2 and 146.6, two methine carbons at *δ*_C_ 110.8 and 108.6, and two nonhydrogenated carbons at *δ*_C_ 137.1 and 128. Furthermore, the resonance observed at *δ*_C_ 177.9 in the ^13^C NMR spectrum was assigned to C-20, indicating the presence of a lactone carbonyl in **9**. In the HMBC spectrum, the correlation of the signal at *δ*_H_ 3.16 (1H, sept., *J* = 6.9 Hz, H-15) with the signals at *δ*_C_ 108.6 and 150.2 confirmed the chemical shifts of C-12 and C-14, respectively (Figure 2). Consequently, the HSQC correlation between the proton at *δ*_H_ 6.57 (1H, s) and the carbon at *δ*_C_ 110.2, as well as the signal at *δ*_H_ 6.86 (1H, s) with the carbon at *δ*_C_ 108.6, confirmed the chemical shifts of the two hydrogenated aromatic carbons at C-8 and C-12, respectively. According to the information provided, it is suggested that the lactone ring in compound **9** formed via the C ring. HMBC correlations from H-8 (*δ*_H_ 6.57, 1H, s) to C-14 (*δ*_C_ 150.2), C-11 (*δ*_C_ 146.6), C-13 (*δ*_C_ 137.1), C-9 (*δ*_C_ 128.3), and C-10 (*δ*_C_ 53.1) confirmed that C-9, C-10, C-11, and C-20, along with an oxygen atom, formed a *γ*-lactone ring (Figure 2). 

In addition, the presence of a signal at *δ*_H_ 9.19 (1H, t, *J* = 1.5 Hz) in the ^1^H NMR spectrum, along with the correlation of this proton with the carbon at *δ*_C_ 199.5 in the HSQC spectrum, suggested the presence of an aldehydic group in **9**. The correlation of the aldehydic proton (*δ*_H_ 9.19, 1H, t, *J* = 1.5 Hz) and H-5 (*δ*_H_ 2.34, 1H, dd, *J* = 6.1, 4.6 Hz) with the C-6 carbon (*δ*_C_ 41.8) in the HMBC spectrum (Figure 2), along with the COSY spin system H-5/H-6/H-7, determined the position of the aldehyde group at C-7. The conjunction of these correlations established that **9** is a 7-8-*seco*-abietane.

For the same reason as mentioned for compound **8**, the relative configuration of C-2 in **9** was proposed using quantum GIAO NMR chemical shift calculations and DP4+ analysis. The ^13^C and ^1^H NMR data of two possible isomers, (2R*,5S*,10R*)–9a and (2S*,5S*,10R*)–9b, were calculated. The DP4+ probability assessment indicated that the (2S*,5S*,10R*)–9b isomer was highly probable, at 100% (Figure S174). To determine the absolute configuration of **9**, the experimental and calculated ECD results were compared, and 9 was identified as 2*S*,5*S*,10*R* (Figure 4). Ultimately, compound **9** was named medusanthol F.

Furthermore, the structures of the identified known diterpenoids, salviol (**4**) [23] and 2*α*-hydroxysugiol (**6**) [19], were confirmed by comparing their spectroscopic data with reported values in the literature. Here, we present the 1D NMR, HRESIMS, ECD, and IR data, along with ^13^C and ^1^H NMR calculations and DP4+ probability analysis for compounds **4** and **6** (see Supplementary Materials). 

### 2.2. Biological Activity

#### Anti-Neuroinflammatory Activity

Neuroinflammation is characterized by the prolonged activation of glial cells and the influx of immune cells into the nervous system and plays a significant role in the progression of neurodegenerative disorders such as Alzheimer’s disease, Parkinson’s disease, amyotrophic lateral sclerosis, and traumatic brain injury [24]. Research has shown that abietanes in the Lamiaceae family have the potential to reduce neuroinflammation and act as antioxidants [25,26].

The noncytotoxic concentrations of compounds **1**–**7** in BV2 cells were determined using the MTT assay. Our results showed that at 50 µM, most compounds reduced cell viability by more than 20%. On the other hand, at 12.5 and 25 µM, cell viability greater than 80% was observed for all the compounds (Table 3). Therefore, 12.5 µM was considered a safe concentration for assessing the anti-neuroinflammatory effects of compounds **1**–**7**.

**Figure 5:**
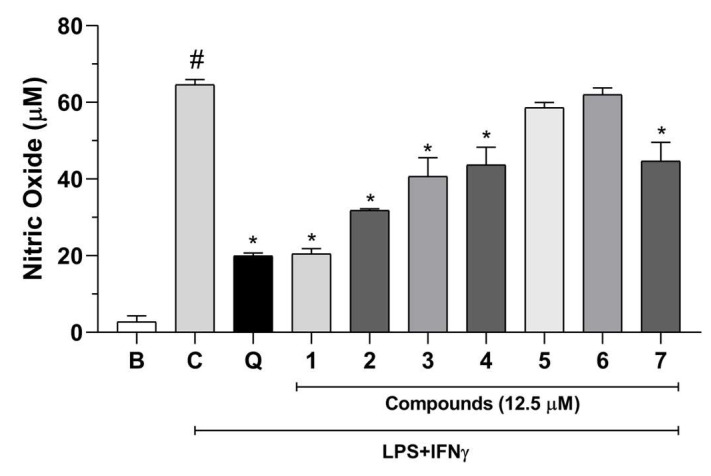
Effects of compounds **1**–**7** (12.5 μM) on the nitric oxide measurement in LPS and IFN-γ-stimulated BV2 cells. Results are expressed as the mean ± SEM (*n* = 5) of two independent experiments. B: basal. C: control. Q: quercetin (positive control, 20 µM). # *p* < 0.05 versus basal group; * *p* < 0.05 versus control group.

The anti-neuroinflammatory effects of compounds **1**–**7** were initially evaluated by determining the levels of nitrite, a stable metabolite of NO. As shown in Figure 5, the LPS/IFN-γ-induced inflammatory response was greater in the control group than in the basal group (unstimulated). At 12.5 μM, compounds **1**–**4** and **7** significantly reduced nitrite levels compared to those in the control group. No significant effect was recorded for compounds **5** and **6**. As expected, the positive control quercetin (20 μM) also significantly reduced nitrite levels in stimulated BV2 cells. Nitric oxide plays a crucial role in inflammation, including its involvement in neurodegenerative diseases [27]. Thus, our results suggest that compounds **1**, **2**, **3**, **4**, and **7** exert anti-neuroinflammatory effects.

Considering the promising results for compounds **1** and **2**, a new set of experiments was performed to calculate the IC_50_ values at concentrations of 3.125, 6.250, 12.5, and 25 μM. Compounds **1** and **2** exhibited IC_50_ values of 3.12 and 15.53 μM, respectively (Table 4). The IC_50_ value for the positive control quercetin was 11.8 μM. These results support the potent anti-neuroinflammatory effect, especially for compound **1**. Moreover, considering that TNF-α acts as an important inflammatory mediator [28], the inhibitory effects of compounds **1** and **2** on LPS- and IFN-γ-induced TNF-α release from BV2 cells were assessed.

**Figure 6:**
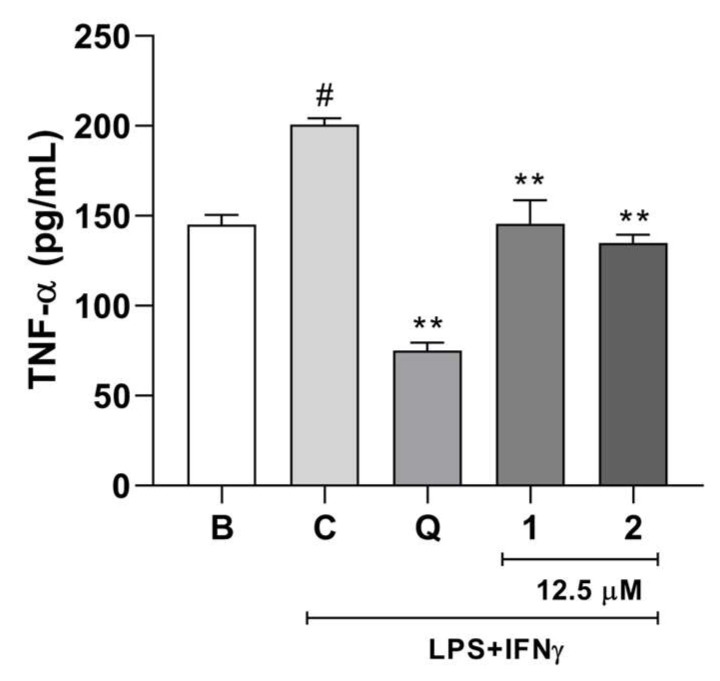
Effects of compounds **1** and **2** (12.5 μM) on TNF-α measurement in LPS and IFN-γ-stimulated BV2 cells. Results are expressed as the mean ± SEM (*n* = 5). B: basal. C: control. Q: quercetin (positive control, 20 μM). # *p* < 0.01 versus basal group; ** *p* < 0.01 versus control group.

Compounds **1** and **2** significantly reduced TNF-α levels in stimulated BV2 cells compared to those in the control group (Figure 6). Data from the literature have shown that inflammation induced in BV2 cells increases the activation of signaling pathways such as the NF-κB and MAPK pathways, leading to the production of cytokines, including TNF-α [29]. TNF-α is a proinflammatory cytokine that modulates the immune system and plays a role in all types of inflammatory disorders, such as central nervous system disorders [30]. Therefore, the anti-neuroinflammatory effects of compounds **1** and **2** are linked to the inhibition of NO and TNF-α release from BV2 cells.

## 3. Experimental Section 

### 3.1. General Experimental Procedures

Optical rotations were measured on a JASCO P-2000 polarimeter (JASCO, Tokyo Japan). Infrared (IR) spectra were recorded on a Shimadzu IRPrestige-21 spectrometer (Shimadzu, Kyoto, Japan) using the KBr disk method. NMR data were acquired on Bruker Ascend 400 MHz and Bruker AvanceNeo 500 MHz spectrometers (Bruker, Billerica, MA, USA) using the residual nondeuterated solvent peaks as an internal standard. The experimental ECD spectra were obtained on a JASCO J-1100 CD Spectrometer (JASCO, Tokyo Japan). The vacuum-liquid chromatography (VLC) system was constructed in a Büchner funnel, and an Erlenmeyer flask was connected to a vacuum system using silica gel (60–200 μm, 70–230 mesh, SiliaCycle, Quebec, QC, Canada) as the packing material. High-resolution electrospray ionization mass spectrometry (HRESIMS) analyses were carried out using a Bruker micrOTOF II spectrometer (Bruker, Billerica, MA, USA) operating in positive mode. Analytical high-performance liquid chromatography (HPLC) was performed on a Prominence Shimadzu instrument (Shimadzu, Kyoto, Japan) equipped with an SPD-M20A diode array detector and a YMC C-18 (250 mm × 4.6 mm × 5 µm) column. Semipreparative HPLC separations were conducted on a Shimadzu 10AVP instrument (Shimadzu, Kyoto, Japan) with an SPD-M10AVP detector on a Venusil XBP C-18 (259 mm × 10 mm × 10 μm) column. For preparative HPLC isolations, a Shimadzu apparatus with an SPD-M10A diode array detector and a YMC-Triart^®^ C-18 (250 mm × 20 mm × 5 µm) column was used.

### 3.2. Plant Material

The aerial parts of *Medusantha martiusii* (Benth.) Harley and J. F. B. Pastore were collected in July 2019 at Maturéia, a Caatinga region of Paraíba, Brazil (07°16′01″ S, 37°21′05″ W). The sample was authenticated by Maria de Fátima Agra. A specimen is housed under the code JPB 37884 at the Herbarium Prof. Lauro Pires Xavier (JPB) at the Federal University of Paraíba (UFPB), Brazil. This species was registered under the code AB7F3C9 in the National System for the Management of Genetic Heritage and Associated Traditional Knowledge (SisGen-Brasil).

### 3.3. Extraction, Isolation, and Purification Process

The dried and pulverized aerial parts of *M. martiusii* (1.2 kg) were macerated in hexane four times (4 L) and then in 96% ethanol (4 L) five times, with each cycle lasting 72 h. The filtrates were concentrated under reduced pressure, producing 17.5 g and 43.3 g of hexanic and ethanolic extract, respectively. The hexanic extract (10.0 g) was fractionated by vacuum liquid chromatography using a solvent gradient of Hex–CHCl_3_–EtOAc (20:80:0 → 60:40:0 → 50:50:0 → 40:60:0 → 20:80:0 → 0:0:100, *v*/*v*/*v*) to obtain six fractions A–F. Fraction F (400 mg) was subjected to preparative HPLC using the following system: solvent A = Milli-Q water with 0.1% formic acid; solvent B = CH_3_CN; elution profile = 0.0–38.0 min (50–62% B); 38.0–60.0 min (62–70% B); YMC-Triart^®^ C-18 column; volume injection 200 μL and flow rate of 8 mL/min to yield compounds **1** (5 mg, *t*_R_ = 12.2 min), **2** (4 mg, *t*_R_ = 12.7 min), **6** (2 mg, *t*_R_ = 21.5 min), **3** (24.1 mg, *t*_R_ = 29.6 min), **9** (2 mg, *t*_R_ = 39.3 min), **8** (1.5 mg, *t*_R_ = 43.3 min), and **7** (1.5 mg, *t*_R_ = 46.1 min), as well as the F_1_ subfraction (62.2 mg, *t*_R_ = 52.3 min), which contained a mixture of substances. The F_1_ fraction was further purified by semipreparative HPLC using the following method: solvent A = Milli-Q water; solvent B = CH_3_CN; elution profile = 0.0–80.0 min (50% B); Venusil XBP C-18 column; volume injection 100 μL and flow rate of 3 mL/min to obtain compounds **4** (2.6 mg, *t*_R_ = 71.5 min) and **5** (10.7 mg, *t*_R_ = 77.4 min).

### 3.4. Characterization Data 

Medusanthol A (**1**): brown amorphous powder; ${\left[\alpha \right]}_{D}^{23}$ − 6.8 (*c* 0.1, MeOH); IR (KBr) *ν*_max_ 3432, 1655, 1640 cm^−1^; ^1^H and ^13^C NMR data, see Table 1 and Table 2; HRESIMS *m*/*z* 331.1896 [M − H_2_O + H]^+^ (calcd for C_20_H_27_O_4_, 331.1904, Δ = 2.2 ppm).

Medusanthol B (**2**): white amorphous powder; ${\left[\alpha \right]}_{D}^{23}$ + 6.4 (*c* 0.1, MeOH); IR (KBr) *ν*_max_ 3448, 1658, 1595, 1460 cm^−1^; ^1^H and ^13^C NMR data, see Table 1 and Table 2; HRESIMS *m*/*z* 687.3863 [2M + Na]^+^ (calcd for C_40_H_56_NaO_8_, 687.3867, Δ = 0.6 ppm).

Medusanthol C (**3**): needle crystal; ${\left[\alpha \right]}_{D}^{23}$ + 27.9 (*c* 0.1, MeOH); IR (KBr) *ν*_max_ 3336, 1618, 1425 cm^−1^; ^1^H and ^13^C NMR data, see Table 1 and Table 2; HRESIMS *m*/*z* 659.4276 [2M + Na]^+^ (calcd for C_40_H_60_NaO_6_, 659.4282, Δ = 0.9 ppm).

Medusanthol D (**5**): white amorphous powder; ${\left[\alpha \right]}_{D}^{23}$ + 23.5 (*c* 0.1, MeOH); IR (KBr) *ν*_max_ 3431, 1735, 1269, 1710, 1658, 1510, 1421 cm^−1^; ^1^H and ^13^C NMR data, see Table 1 and Table 2; HRESIMS *m*/*z* 397.1974 [M + Na]^+^ (calcd for C_22_H_30_NaO_5_, 397.1985, Δ = 2.8 ppm).

Medusanthol E (**7**): white amorphous powder; ${\left[\alpha \right]}_{D}^{23}$ − 7.9 (*c* 0.1, CHCl_3_); IR (KBr) *ν*_max_ 3446, 1786 cm^−1^; ^1^H and ^13^C NMR data, see Table 1 and Table 2; HRESIMS *m*/*z* 799.3652 [2M + Na]^+^ (calcd for C_44_H_56_NaO_12_, 799.3664, Δ = 1.5 ppm).

Medusanthol F (**8**): white amorphous powder; ${\left[\alpha \right]}_{D}^{23}$ + 5.7 (*c* 0.1, CHCl_3_); IR (KBr) *ν*_max_ 3446, 1786, 1720, 1695 cm^−1^; ^1^H and ^13^C NMR data, see Table 1 and Table 2; HRESIMS *m*/*z* 409.1612 [M + Na]^+^ (calcd for C_22_H_26_NaO_6_, 409.1622, Δ = 2.3 ppm).

Medusanthol G (**9**): yellow amorphous powder; ${\left[\alpha \right]}_{D}^{23}$ − 32.8 (*c* 0.1, CHCl_3_); IR (KBr) *ν*_max_ 3427, 1791, 1745, 1724 cm^−1^; ^1^H and ^13^C NMR data, see Table 1 and Table 2; HRESIMS *m*/*z* 799.3637 [2M + Na]^+^ (calcd for C_44_H_56_NaO_12_, 799.3664, Δ = 3.4 ppm).

### 3.5. NMR and ECD Calculations

The three-dimensional molecular structures of the compounds were obtained using ChemSketch software version C25E41 [30]. Stochastic conformational searches were performed for all possible stereoisomers of each compound using the Monte Carlo method and the molecular mechanic force field (MMFF) in SPARTAN’10 software version 1.1.0 [31]. All conformers within a relative free energy window of 10 kcal mol^−1^ were reoptimized using the B3LYP/6-31G(d) level of theory. The conformations within the energy range of 2.5 kcal mol^−1^ above the minimum energy conformer, corresponding to more than 90% of the total Boltzmann population, were selected for the GIAO NMR calculations and the simulations of the ECD spectra. To simulate nuclear magnetic shielding, the GIAO-mPW1PW91/6-31+G(d,p) level of theory was used, employing a polarizable continuum model with integral equation formalism (IEF-PCM) to implicitly simulate chloroform as a solvent. The ^1^H and ^13^C NMR chemical shifts (δ) were obtained using δ_i_ = σ_0_ − σ_i_ after the calculation of the shielding constant of the tetramethylsilane (σ_0_) using the same levels of theory. For the application of the DP4+ method, as recommended by the author, the nuclear magnetic shields for all candidates of each compound were added to the DP4+ Excel spreadsheet [32]. For the ECD simulations, TD-DFT was performed in acetonitrile at the CAM-B3LYP/TZVP level. The IEF-PCM model for acetonitrile was used. The final ECD spectra were obtained based on the weighted average Boltzmann statistics of the selected conformers and plotted using Origin 8 software [33]. All quantum-mechanical calculations were performed using the Gaussian 09 software package [34].

### 3.6. Anti-Neuroinflammatory Assay 

#### 3.6.1. Cell Viability (MTT Assay)

The cytotoxicity of compounds **1**–**7** was evaluated using the MTT (3-(4,5-dimethylthiazol-2-yl)-2,5-diphenyltetrazolium bromide) assay [35]. The microglial BV2 cell line was obtained from the Rio de Janeiro Cell Bank (BCRJ), Brazil. The cells were cultured in Roswell Park Memorial Institute medium (RPMI; Sigma Aldrich, St. Louis, MO, USA) supplemented with 10% fetal bovine serum (FBS; Gibco, Grand Island, NY, USA) and 1% penicillin-streptomycin (Sigma Aldrich) at 37 °C with 5% CO_2_. Cells were seeded into 96-well plates at 1 × 10^5^ cells/mL and incubated overnight. After that, the cells were incubated with compounds **1** to **7** (12.5, 25, or 50 μM) in five replicates for 24 h. Then, 110 μL of the supernatant were removed, and 10 μL of MTT solution (5 mg/mL) (Sigma Aldrich, St. Louis, MO, USA) were added. The plates were further incubated for four hours, followed by the addition of sodium dodecyl sulfate (SDS) (100 µL/well) to dissolve the formazan. Optical densities were measured using a spectrophotometer (BioTek Instruments microplate reader, Sinergy HT, Winooski, VT, USA) at a wavelength of 570 nm.

#### 3.6.2. Nitric Oxide (NO) and TNF-α Measurement

To determine the NO and TNF-α levels, BV2 cells were seeded in 96-well plates (1 × 10^6^ cells/mL) in RPMI medium supplemented with 10% FBS and 1% penicillin-streptomycin in a 5% CO_2_ incubator at 37 °C. After four hours, the cells were exposed to LPS (500 ng/mL, Sigma Aldrich) and IFN-γ (5 ng/mL, Thermo Fisher) in the absence or presence of compounds **1** to **7** at a final concentration of 12.5 µM (for NO and TNF-α measurement) or 3.125–25 µM (to calculate the IC_50_ values on NO production inhibition), in five replicates. Quercetin (20 µM) was used as a positive control. After 24 h, cell-free supernatants were collected for NO quantification using the Griess method [36] or stored at −80 °C for cytokine concentration determination. The TNF-α concentrations in the BV2 cell culture supernatants were assessed via enzyme-linked immunosorbent assay (ELISA) with an Invitrogen kit (Thermo Fisher, Viena, Austria).

#### 3.6.3. Statistical Analysis

The results are expressed as the mean ± standard error of the mean (SEM), and group comparisons were conducted using one-way analysis of variance (ANOVA) followed by Tukey’s post hoc test (*p* < 0.05).

## 4. Conclusions

Seven new abietane diterpenoids, comprising medusanthol A–G (**1**–**3**, **5**, **7**–**9**) and two previously identified analogs (**4** and **6**), were isolated from the hexane extract of the aerial parts of *Medusantha martiusii*. Compounds **1**–**4** and **7** exhibited significant anti-neuroinflammatory activity in BV2 microglia. Notably, compound **1** exhibited a potent anti-neuroinflammatory effect with an IC_50_ value of 3.12 μM, while compound **2** displayed an IC_50_ of 15.53 μM, effectively decreasing NO levels. Additionally, these compounds also reduced TNF-α levels, suggesting their involvement in pathways that mitigate neuroinflammation. Overall, these results not only emphasize the diversity of diterpenes in Nepetoideae but also establish a foundation for its properties in accordance with its traditional usage, thereby reaffirming the potential of the Caatinga biome in uncovering new bioactive compounds.

## Figures and Tables

**Table 1:** ^13^C NMR data of compounds **1**–**3**, **5**, and **7**–**9**.

No.	1 ^a^	2 ^b^	3 ^b^	5 ^b^	7 ^a^	8 ^a^	9 ^a^
1	42.3	45.6	46.6	43.0	36.8	32.0	40.0
2	68.8	69.4	70.0	70.8	67.9	67.3	66.4
3	82.8	83.5	84.3	47.7	45.3	43.6	46.1
4	38.9	40.5	40.5	35.8	36.3	34.0	36.3
5	48.3	50.3	51.5	53.2	50.6	59.5	45.7
6	26.1	36.6	20.4	19.6	26.7	81.4	41.8
7	67.9	200.6	31.1	30.5	95.4	189.1	199.5
8	141.9	123.7	126.5	128.7	145.8	121.8	110.2
9	150.6	157.2	148.0	139.1	121.7	143.8	128.3
10	40.0	39.9	39.5	49.5	47.3	49.2	53.1
11	188.0	110.6	111.6	112.4	112.3	111.0	146.6
12	132.4	162.6	153.4	153.5	148.1	159.1	108.6
13	153.6	135.2	133.8	135.3	136.7	136.1	137.1
14	190.1	127.3	127.4	128.2	117.3	128.2	150.2
15	26.4	27.9	27.7	27.9	26.8	27.0	27.4
16	21.5	22.8	23.3	23.1	22.3	22.3	22.5
17	21.4	22.9	23.2	23.2	22.2	22.4	22.8
18	28.8	28.6	29.4	32.6	30.8	31.4	33.3
19	17.0	16.9	17.4	21.5	20.8	22.7	22.0
20	21.1	24.6	26.3	179.0	170.9	176.0	177.9
2-OCOCH_3_	-	-	-	21.4	21.3	21.4	21.4
2-OCOCH_3_	-	-	-	172.6	169.9	170.0	170.3

^a^ Recorded in CDCl_3_, 125 MHz; ^b^ recorded in methanol-*d*4, 100 MHz.

**Table 2:** ^1^H NMR data of compounds **1**–**3**, **5,** and **7**–**9** (*J* in Hz).

No.	1 ^a^	2 ^b^	3 ^b^	5 ^b^	7 ^c^	8 ^c^	9 ^c^
1	1.15, m 3.06, dd (12.7, 4.4)	1.56, t (11.9) 2.55, dd (12.5, 4.3)	1.40, t (12.0) 2.49, dd (12.4, 4.4)	1.27, overlap 3.09, ddd (12.1, 4.5, 2.8)	1.85, t (11.7) 2.77 ddd (11.8, 3.7, 2.4)	1.76, t (12.0) 3.01, dd (12.6, 4.3)	1.71, dd (13.2, 11.7) 2.23, ddd (13.3, 4.0, 2.5)
2	3.82, ddd (11.5, 9.6, 4.4)	3.83, ddd (11.7, 9.6, 4.3)	3.78, ddd (11.6, 9.6, 4.4)	5.42, tt (11.7, 4.5)	5.22, tt (11.5, 3.9)	5.04, tt (11.6, 4.1)	5.50, tt (11.7, 3.9)
3	3.01, d (9.6)	3.02, d (9.6)	2.99, d (9.6)	1.86, overlap 1.27, overlap	1.14–1.23, m 1.97, ddd (12.4, 4.1, 2.4)	1.88, m 1.24, overlap	2.03, ddd (12.7, 4.0, 2.5) 1.49, t (12.3)
5	1.15, m	1.89, dd (13.4, 4.2)	1.32, dd (12.4, 2.4)	1.50, dd (12.8, 2.4)	2.15, dd (10.5, 2.0)	2.41, s	2.34, dd (6.1, 4.6)
6	2.20, m 1.64, m	2.64, m 2.64, m	1.84, m 1.71, m	2.54, m 1.86, overlap	2.24 ddd (15.9, 6.5, 2.0) 2.35 dd (15.9, 10.5)	4.77, s	2.46, ddd (17.8, 4.6, 1.5) 1.94, ddd (17.8, 6.1, 1.5)
7	4.79, dd (10.2, 7.5)	-	2.71, m 2.82, m	2.87, m 2.76, m	5.94 d (6.5)	-	9.19, t (1.5)
8	-	-	-	-	-	-	6.57, s
11	-	6.76, s	6.65, s	6.68, s	6.69, s	6.71, s	-
12	6.36, d (1.2)	-	-	-	-	-	6.86, s
14	-	7.80, s	6.75, s	6.85, s	6.70, s	7.91, s	-
15	2.97, m	3.22, sept (6.8)	3.16, sept (6.8)	3.18, sept (6.8)	3.08, sept (6.8)	3.16, sept (6.8)	3.16, sept (6.9)
16	1.10, s	1.19, d (6.8)	1.16, d (6.8)	1.17, d (6.8)	1.18, d (6.8)	1.23, s	1.20, d (6.9)
17	1.08, s	1.21, d (6.8)	1.15, d (6.8)	1.17, d (6.8)	1.19, d (6.8)	1.24, s	1.18, d (6.9)
18	1.07, s	1.06, s	1.07, s	1.02, s	0.86, s	1.07, s	0.94, s
19	0.91, s	0.98, s	0.89, s	0.93, s	0.96, s	1.05, s	1.23, s
20	1.40, s	1.27, s	1.19, s	-	-	-	-
2-OCOCH_3_	-	-	-	2.02, s	2.05, s	2.06, s	1.99, s

^a^ Recorded in CDCl_3_, 400 MHz; ^b^ recorded in methanol-*d*4, 400 MHz; ^c^ recorded in CDCl3, 500 MHz.

**Table 3:** Cell viability (%) of BV2 cells treated with compounds **1**–**7**.

Compound	Cell Viability (%)		
	12.5 µM	25 µM	50 µM
1	87.95 ± 2.57	85.09 ± 1.54	76.28 ± 2.16
2	81.93 ± 0.65	85.84 ± 0.85	74.95 ± 3.11
3	87.39 ± 2.35	82.19 ± 0.96	76.37 ± 3.14
4	92.29 ± 0.60	85.41 ± 0.82	73.61 ± 2.73
5	86.18 ± 4.53	86.80 ± 2.52	82.90 ± 3.63
6	85.71 ± 1.11	81.71 ± 1.37	66.81 ± 3.61
7	82.74 ± 0.62	84.91 ± 1.40	81.83 ± 1.24

Results are expressed as the mean ± SEM (*n* = 5) of two independent experiments.

**Table 4:** The IC_50_ values of compounds **1** and **2** on nitric oxide production inhibition in LPS and IFN-γ-stimulated BV2 cells.

Compound	IC_50_ (µM) ^2^
1	3.12 ± 0.75
2	15.53 ± 7.56
Quercetin ^1^	11.8 ± 1.5

^1^ IC_50_ means half maximal (50%) inhibitory concentration. Results are presented as the mean ± SEM (95% confidence interval). ^2^ Quercetin was used as positive control.

## Data Availability

The authors declare that all relevant data supporting the results of this study are available within the article and its Supplementary Materials.

## Supplementary Materials

The following supporting information can be downloaded at https://www.mdpi.com/article/10.3390/molecules29122723/s1: Figures S1–S149: NMR, IR, HRESIMS of compounds **1**–**9**. Tables S1–S12: Comparative analysis of theoretical and experimental ^1^H and ^13^C NMR data of compounds **1**–**9**. Figures S150–S176: DP4+ data, lowest energy conformer data at the B3LYP/6-31G(d) level, and comparison of experimental and calculated ECD data of compounds **1**–**9**.

## References

[1] Harley R.M., Pastore J.F.B. (2012). A Generic Revision and New Combinations in the Hyptidinae (Lamiaceae), Based on Molecular and Morphological Evidence. Phytotaxa.

[2] Monteiro F.K.D.S., Melo J.I.M.D. (2020). Flora da Paraíba, Brasil: Subfamília Nepetoideae (Lamiaceae). Rodriguésia.

[3] Bornowski N., Hamilton J.P., Liao P., Wood J.C., Dudareva N., Buell C.R. (2020). Genome Sequencing of Four Culinary Herbs Reveals Terpenoid Genes Underlying Chemodiversity in the Nepetoideae. DNA Res..

[4] Ortiz-Mendoza N., Martínez-Gordillo M.J., Martínez-Ambriz E., Basurto-Peña F.A., González-Trujano M.E., Aguirre-Hernández E. (2023). Ethnobotanical, Phytochemical, and Pharmacological Properties of the Subfamily Nepetoideae (Lamiaceae) in Inflammatory Diseases. Plants.

[5] Sun Y., Yang H.-Y., Huang P.-Z., Zhang L.-J., Feng W.-J., Li Y., Gao K. (2023). Abietane Diterpenoids with Anti-Inflammatory Activities from *Callicarpa Bodinieri*. Phytochemistry.

[6] Kolsi L.E., Leal A.S., Yli-Kauhaluoma J., Liby K.T., Moreira V.M. (2018). Dehydroabietic Oximes Halt Pancreatic Cancer Cell Growth in the G1 Phase through Induction of P27 and Downregulation of Cyclin D1. Sci. Rep..

[7] Abdissa N., Frese M., Sewald N. (2017). Antimicrobial Abietane-Type Diterpenoids from *Plectranthus punctatus*. Molecules.

[8] Tabefam M., Farimani M.M., Danton O., Ramseyer J., Kaiser M., Ebrahimi S.N., Salehi P., Batooli H., Potterat O., Hamburger M. (2018). Antiprotozoal Diterpenes from *Perovskia abrotanoides*. Planta Med..

[9] Agra M.D.F., Silva K.N., Basílio I.J.L.D., Freitas P.F.D., Barbosa-Filho J.M. (2008). Survey of Medicinal Plants Used in the Region Northeast of Brazil. Rev. Bras. Farmacogn..

[10] Ranzato Filardi F.L., Barros F.D., Baumgratz J.F.A., Bicudo C.E.M., Cavalcanti T.B., Nadruz Coelho M.A., Costa A., Costa D., Goldenburg R., Labiak P.H. (2018). BFG Brazilian Flora 2020: Innovation and Collaboration to Meet Target 1 of the Global Strategy for Plant Conservation (GSPC). Rodriguésia.

[11] Araújo E.C.C., Lima M.A.S., Silveira E.R. (2004). Spectral Assignments of New Diterpenes from *Hyptis martiusii* Benth. Magn. Reson. Chem..

[12] Araújo E.C.C., Lima M.A.S., Montenegro R.C., Nogueira M.A.S., Costa-Lotufo L.V., Pessoa C., Moraes M.O., Silveira E.R. (2006). Cytotoxic Abietane Diterpenes from *Hyptis martiusii* Benth. Z. Für Naturforschung C.

[13] Cavalcanti B.C., Moura D.J., Rosa R.M., Moraes M.O., Araújo E.C.C., Lima M.A.S., Silveira E.R., Saffi J., Henriques J.A.P., Pessoa C. (2008). Genotoxic Effects of Tanshinones from *Hyptis martiusii* in V79 Cell Line. Food Chem. Toxicol..

[14] Barbosa A.G.R., Tintino C.D.M.O., Pessoa R.T., de Lacerda Neto L.J., Martins A.O.B.P.B., de Oliveira M.R.C., Coutinho H.D.M., Cruz-Martins N., Quintans L.J., Wilairatana P. (2022). Anti-Inflammatory and Antinociceptive Effect of *Hyptis martiusii* BENTH Leaves Essential Oil. Biotechnol. Rep..

[15] Levy G.C., Lichter R.L., Nelson G.L. (1980). Carbon-13 Nuclear Magnetic Resonance Spectroscopy.

[16] Han D., Li W., Hou Z., Lin C., Xie Y., Zhou X., Gao Y., Huang J., Lai J., Wang L. (2023). The Chromosome-Scale Assembly of the *Salvia rosmarinus* Genome Provides Insight into Carnosic Acid Biosynthesis. Plant J..

[17] Lima K.S.B.D., Pimenta A.T.A., Guedes M.L.S., Lima M.A.S., Silveira E.R. (2012). Abietane Diterpenes from *Hyptis carvalhoi* Harley. Biochem. Syst. Ecol..

[18] Costa-Lotufo L.V., Araújo E.C.C., Lima M.A.S., Moraes M.E.A., Pessoa C., Silviera E.R., Morais M.O. (2004). Antiproliferative Effects of Abietane Diterpenoids Isolated from *Hyptis martiusii* Benth (Labiatae). Pharmazie.

[19] González A.G., Herrera J.R., Luis J.G., Ravelo A.G., Ferro E.A. (1988). Terpenes and Flavones of *Salvia cardiophylla*. Phytochemistry.

[20] Zhao H., Li H., Huang G., Chen Y. (2017). A New Abietane Mono-Norditerpenoid from *Podocarpus nagi*. Nat. Prod. Res..

[21] Hirata A., Kim S.-Y., Kobayakawa N., Tanaka N., Kashiwada Y. (2015). Miltiorins A–D, Diterpenes from Radix *Salviae miltiorrhizae*. Fitoterapia.

[22] Lin S., Zhang Y., Liu M., Yang S., Gan M., Zi J., Song W., Fan X., Wang S., Liu Y. (2010). Abietane and C_20_-Norabietane Diterpenes from the Stem Bark of *Fraxinus sieboldiana* and Their Biological Activities. J. Nat. Prod..

[23] Zheng T.-L., Liu S.-Z., Huo C.-Y., Li J., Wang B.-W., Jin D.-P., Cheng F., Chen X.-M., Zhang X.-M., Xu X.-T. (2021). Au-Catalyzed 1,3-Acyloxy Migration/Cyclization Cascade: A Direct Strategy toward the Synthesis of Functionalized Abietane-Type Diterpenes. CCS Chem..

[24] Zhang W., Xiao D., Mao Q., Xia H. (2023). Role of Neuroinflammation in Neurodegeneration Development. Signal Transduct. Target. Ther..

[25] Oliveira M.R. (2016). The Dietary Components Carnosic Acid and Carnosol as Neuroprotective Agents: A Mechanistic View. Mol. Neurobiol..

[26] Oliveira M.R., Souza I.C.C., Fürstenau C.R. (2018). Carnosic Acid Induces Anti-Inflammatory Effects in Paraquat-Treated SH-SY5Y Cells Through a Mechanism Involving a Crosstalk Between the Nrf2/HO-1 Axis and NF-κB. Mol. Neurobiol..

[27] Justo A.F.O., Suemoto C.K. (2022). The Modulation of Neuroinflammation by Inducible Nitric Oxide Synthase. J. Cell Commun. Signal..

[28] Konsman J.P. (2022). Cytokines in the Brain and Neuroinflammation: We Didn’t Starve the Fire!. Pharmaceuticals.

[29] Wang H., Wang H., Wang J., Wang Q., Ma Q., Chen Y. (2015). Protocatechuic Acid Inhibits Inflammatory Responses in LPS-Stimulated BV2 Microglia via NF-κB and MAPKs Signaling Pathways. Neurochem. Res..

[30] (2022). ChemSketch.

[31] (2011). Spartan’ 10.

[32] Grimblat N., Zanardi M.M., Sarotti A.M. (2015). Beyond DP4: An Improved Probability for the Stereochemical Assignment of Isomeric Compounds Using Quantum Chemical Calculations of NMR Shifts. J. Org. Chem..

[33] (2023). Origin(Pro).

[34] Frisch M.J., Trucks G.W., Schlegel H.B., Scuseria G.E., Robb M.A., Cheeseman J.R., Scalmani G., Barone V., Petersson G.A., Nakatsuji H. (2016). Gaussian 09.

[35] Mosmann T. (1983). Rapid Colorimetric Assay for Cellular Growth and Survival: Application to Proliferation and Cytotoxicity Assays. J. Immunol. Methods.

[36] Griess P. (1879). Bemerkungen Zu Der Abhandlung Der HH. Weselsky Und Benedikt “Ueber Einige Azoverbindungen”. Berichte Dtsch. Chem. Ges..

