# Supplementary material for: Abietane Diterpenes from Medusantha martiusii and Their Anti-Neuroinflammatory Activity
Source: Molecules. 2024 Jun 7;29(12):2723. doi: 10.3390/molecules29122723 (PMC11207065; doi:10.3390/molecules29122723)
Supplement: Supplementary file 1 [file molecules-29-02723-s001.zip › molecules-3029621-supplementary.pdf]

SUPPLEMENTARY DATA

Abietane Diterpenes from Medusantha martiusii and Their Anti-Neuroinflammatory Activity

Edileuza B. de Assis [1], Rodrigo S. de Andrade 1, Joanda P. R. e Silva 1, Lucas H. Martorano 2, Geraldo M. W. Amorim 1, Paulo B. A. Loureiro 1, Lucas S. Abreu 2, Marianna V. Sobral 1, Marcus T. Scotti 1, Fernando M. dos Santos Junior 2, Maria de Fátima Agra 1, Josean F. Tavares 1,* and Marcelo S. da Silva [1,*]

1 Postgraduate Program in Natural and Synthetic Bioactive Products, Federal University of Paraiba, João Pessoa 58051-900, Brazil; edileuzabezerra@ltf.ufpb.br (E.B.d.A.); rodrigo@ltf.ufpb.br (R.S.d.A.); joandapaolla.1@gmail.com (J.P.R.e.S.); moiseswand@ltf.ufpb.br (G.M.W.A.); paulobrunoaloureiro@ltf.ufpb.br (P.B.A.L.); mariannavbs@gmail.com (M.V.S.); mtscotti@ccae.ufpb.br (M.T.S.); agramf@ltf.ufpb.br (M.d.F.A.)

[2] Department of Organic Chemistry, Fluminense Federal University, Niterói 24020-141, Brazil; lucashm@id.uff.br (L.H.M.); abreu_lucas@id.uff.br (L.S.A.); fernando_martins@id.uff.br (F.M.d.S.J.)

* Correspondence: josean@ltf.ufpb.br (J.F.T.); marcelosobral@ltf.ufpb.br (M.S.d.S.)

CONTENTS

Figure S1. IR spectrum of compound 1 1
Figure S2. HRESIMS Spectrum of **1** ([M - H_2O + H]$^+$ positive ion mode). 1
Figure S3. ^{1}H NMR (400 MHz, $CDCl_3$) spectrum of **1** 2
Figure S4. ^{1}H NMR (400 MHz, $CDCl_3$) spectrum of **1** (expansion). 2
Figure S5. ^{1}H NMR (400 MHz, $CDCl_3$) spectrum of **1** (expansion). 3
Figure S6. ^{1}H NMR (400 MHz, $CDCl_3$) spectrum of **1** (expansion). 3
Figure S7. ^{13}C NMR - BB (125 MHz, $CDCl_3$) spectrum of **1**. 4
Figure S8. ^{13}C NMR - BB (125 MHz, $CDCl_3$) spectrum of **1** (expansion). 4
Figure S9. ^{13}C NMR – DEPT 135 (125 MHz, $CDCl_3$) spectrum of **1** 5
Figure S10. HSQC NMR (400 X 125 MHz, $CDCl_3$) spectrum of **1** 5
Figure S11. HSQC NMR (400 X 125 MHz, $CDCl_3$) spectrum of **1** (expansion) 6
Figure S12. HMBC NMR (400 X 125 MHz, $CDCl_3$) spectrum of **1** 6
Figure S13. HMBC NMR (400 X 125 MHz, $CDCl_3$) spectrum of **1** (expansion). 7
Figure S14. HMBC NMR (400 X 125 MHz, $CDCl_3$) spectrum of **1** (expansion). 7
Figure S15. COSY NMR (400 MHz, $CDCl_3$) spectrum of **1.** 8
Figure S16. COSY NMR (400 MHz, $CDCl_3$) spectrum of **1** (expansion). 8
Figure S17. NOESY NMR (400 MHz, $CDCl_3$) spectrum of **1.** 9
Figure S18. NOESY NMR (400 MHz, $CDCl_3$) spectrum of **1** (expansion). 9
Figure S19. IR spectrum of compound **2.** 10
Figure S20. HRESIMS Spectrum of **2** ([2M + Na]$^+$ positive ion mode) 10
Figure S21. ^{1}H NMR (400 MHz, CD_3OD) spectrum of **2** 11
Figure S22. ^{1}H NMR (400 MHz, CD_3OD) spectrum of 2 (expansion). 11
Figure S23. ^{1}H NMR (400 MHz, CD_3OD) spectrum of **2** (expansion). 12
Figure S24. ^{13}C NMR - BB (100 MHz, CD_3OD) spectrum of **2.** 12
Figure S25. ^{13}C NMR - BB (100 MHz, CD_3OD) spectrum of **2** (expansion). 13
Figure S26. ^{13}C NMR – DEPT 135 (100 MHz, CD_3OD) spectrum of **2** 13
Figure S27. HSQC NMR (400 X 100 MHz, CD_3OD) spectrum of **2** 14
Figure S28. HMBC NMR (400 X 100 MHz, CD_3OD) spectrum of **2** 14
Figure S29. HMBC NMR (400 X 100 MHz, CD_3OD) spectrum of **2** (expansion). 14
Figure S30. HMBC NMR (400 X 100 MHz, CD_3OD) spectrum of **2** (expansion). 15
Figure S31. HMBC NMR (400 X 100 MHz, CD_3OD) spectrum of **2** (expansion). 15
Figure S32. HMBC NMR (400 X 100 MHz, CD_3OD) spectrum of **2** (expansion). 16
Figure S33. COSY NMR (400 MHz, CD_3OD) spectrum of **2** 16
Figure S34. COSY NMR (400 MHz, CD_3OD) spectrum of **2**(expansion). 17

Figure S35. NOESY NMR (400 MHz, CD_3OD) spectrum of **2**.................................. 18
Figure S36. NOESY NMR (400 MHz, CD_3OD) spectrum of **2** (expansion)............... 18
Figure S37. IR spectrum of compound **3**... 18
Figure S38. HRESIMS Spectrum of **3** ($[2M + Na]^+$ positive ion mode)...................... 19
Figure S39. ^{1}H NMR (400 MHz, CD_3OD) spectrum of **3**... 19
Figure S41. ^{1}H NMR (400 MHz, CD_3OD) spectrum of **3** (expansion)......................... 20
Figure S42. ^{1}H NMR (400 MHz, CD_3OD) spectrum of **3** (expansion)......................... 21
Figure S43. ^{13}C NMR - BB (100 MHz, CD_3OD) spectrum of **3**.................................. 21
Figure S44. ^{13}C NMR - BB (100 MHz, CD_3OD) spectrum of **3** (expansion).............. 22
Figure S45. ^{13}C NMR – DEPT 135 (100 MHz, CD_3OD) spectrum of **3.**...................... 22
Figure S46. HSQC NMR (400 X 100 MHz, CD_3OD) spectrum of **3** 23
Figure S47. HMBC NMR (400 X 100 MHz, CD_3OD) spectrum of **3.** 23
Figure S48. HMBC NMR (400 X 100 MHz, CD_3OD) spectrum of **3** (expansion). 24
Figure S49. HMBC NMR (400 X 100 MHz, CD_3OD) spectrum of **3** (expansion). 24
Figure S50. COSY NMR (400 MHz, CD_3OD) spectrum of **3**. 25
Figure S51. COSY NMR (400 MHz, CD_3OD) spectrum of **3** (expansion). 25
Figure S52. NOESY NMR (400 MHz, CD_3OD) spectrum of **3.**................................. 26
Figure S53. NOESY NMR (400 MHz, CD_3OD) spectrum of **3** (expansion)............... 26
Figure S54. IR spectrum of compound **4.**.. 27
Figure S55. HRESIMS Spectrum of **4** ($[M - H_2O + H]^+$ positive ion mode)............... 27
Figure S56. ^{1}H NMR (400 MHz, CD_3OD) spectrum of **4.**.. 28
Figure S57. ^{1}H NMR (400 MHz, CD_3OD) spectrum of **4** (expansion)......................... 28
Figure S58. ^{1}H NMR (400 MHz, CD_3OD) spectrum of **4** (expansion)......................... 29
Figure S59. ^{1}H NMR (400 MHz, CD_3OD) spectrum of **4** (expansion)......................... 29
Figure S60. ^{13}C NMR - BB (100 MHz, CD_3OD) spectrum of **4.**................................ 30
Figure S61. ^{13}C NMR - BB (100 MHz, CD_3OD) spectrum of **4** (expansion).............. 30
Figure S62. ^{13}C NMR - DEPT 135 (100 MHz, CD_3OD) spectrum of **4**....................... 31
Figure S63. HSQC NMR (400 X 100 MHz, CD_3OD) spectrum of **4** 31
Figure S64. HSQC NMR (400 X 100 MHz, CD_3OD) spectrum of **4** (expansion)........ 32
Figure S65. HMBC NMR (400 X 100 MHz, CD_3OD) spectrum of **4.** 32
Figure S66. HMBC NMR (400 X 100 MHz, CD_3OD) spectrum of **4** (expansion). 33
Figure S67. IR spectrum of compound **5**... 33
Figure S68. HRESIMS Spectrum of **5** ($[M + Na]^+$ positive ion mode)......................... 34
Figure S69. ^{1}H NMR (400 MHz, CD_3OD) spectrum of **5**... 34
Figure S70. ^{1}H NMR (400 MHz, CD_3OD) spectrum of **5** (expansion)......................... 35

Figure S71. ^{1}H NMR (400 MHz, CD_3OD) spectrum of **5** (expansion). 36
Figure S72. ^{1}H NMR (400 MHz, CD_3OD) spectrum of **5** (expansion). 36
Figure S73. ^{13}C NMR - BB (100 MHz, CD_3OD) spectrum of **5** 37
Figure S74. ^{13}C NMR - BB (100 MHz, CD_3OD) spectrum of **5** (expansion). 37
Figure S75. ^{13}C NMR - BB (100 MHz, CD_3COCD_3) spectrum of **5** 38
Figure S76. ^{13}C NMR – DEPT 135 (100 MHz, CD_3OD) spectrum of **5**....................... 38
Figure S77. HSQC NMR (400 X 100 MHz, CD_3OD) spectrum of **5** 39
Figure S78. HSQC NMR (400 X 100 MHz, CD_3OD) spectrum of **5** (expansion)........ 39
Figure S79. HMBC NMR (400 X 100 MHz, CD_3OD) spectrum of **5** 40
Figure S80. HMBC NMR (400 X 100 MHz, CD_3OD) spectrum of **5** (expansion). 40
Figure S81. HMBC NMR (400 X 100 MHz, CD_3OD) spectrum of **5** (expansion). 41
Figure S82. COSY NMR (400 MHz, CD_3OD) spectrum of **5** 41
Figure S83. COSY NMR (400 MHz, CD_3OD) spectrum of **5** (expansion). 42
Figure S84. COSY NMR (400 MHz, CD_3OD) spectrum of **5** (expansion). 42
Figure S85. NOESY NMR (400 MHz, CD_3COCD_3) spectrum of **5.**............................ 43
Figure S86. IR spectrum of compound **6**.. 43
Figure S87. HRESIMS Spectrum of **6** ($[2M + Na]^+$ positive ion mode)....................... 44
Figure S88. ^{1}H NMR (400 MHz, $CDCl_3$) spectrum of **6.**... 44
Figure S89. ^{1}H NMR (400 MHz, $CDCl_3$) spectrum of **6** (expansion). 45
Figure S90. ^{1}H NMR (400 MHz, $CDCl_3$) spectrum of **6** (expansion). 45
Figure S91. ^{13}C NMR - BB (125 MHz, $CDCl_3$) spectrum of **6.**.................................. 46
Figure S92. ^{13}C NMR – DEPT 135 (125 MHz, $CDCl_3$) spectrum of **6**......................... 46
Figure S93. IR spectrum of compound **7**.. 47
Figure S94. HRESIMS Spectrum of **7** ($[2M + Na]^+$ positive ion mode)**.**...................... 47
Figure S95. ^{1}H NMR (500 MHz, $CDCl_3$) spectrum of **7**... 47
Figure S96. ^{1}H NMR (500 MHz, $CDCl_3$) spectrum of **7** (expansion). 48
Figure S97. ^{1}H NMR (500 MHz, $CDCl_3$) spectrum of **7** (expansion). 48
Figure S98. ^{1}H NMR (500 MHz, $CDCl_3$) spectrum of **7** (expansion). 49
Figure S99. ^{13}C NMR - BB (125 MHz, $CDCl_3$) spectrum of **7**.................................... 49
Figure S100. ^{13}C NMR - BB (125 MHz, $CDCl_3$) spectrum of **7** (expansion). 50
Figure S101. ^{13}C NMR – DEPT 135 (125 MHz, $CDCl_3$) spectrum of **7**...................... 50
Figure S102. ^{13}C NMR – DEPT 135 (125 MHz, $CDCl_3$) spectrum of **7** (expansion)... 51
Figure S103. HSQC NMR (500 X 125 MHz, $CDCl_3$) spectrum of **7** 51
Figure S104. HMBC NMR (500 X 125 MHz, $CDCl_3$) spectrum of **7** 52
Figure S105. HMBC NMR (500 X 125 MHz, $CDCl_3$) spectrum of **7** (expansion). 52

Figure S106. HMBC NMR (500 X 125 MHz, $CDCl_3$) spectrum of **7** (expansion). 53
Figure S107. HMBC NMR (500 X 125 MHz, $CDCl_3$) spectrum of **7** (expansion). 53
Figure S108. COSY NMR (500 MHz, $CDCl_3$) spectrum of **7** 54
Figure S109. COSY NMR (500 MHz, $CDCl_3$) spectrum of **7** (expansion). 54
Figure S110. COSY NMR (500 MHz, $CDCl_3$) spectrum of **7** (expansion). 55
Figure S111. NOESY NMR (500 MHz, $CDCl_3$) spectrum of **7.**.................................. 55
Figure S112. NOESY NMR (500 MHz, $CDCl_3$) spectrum of **7** (expansion)................ 56
Figure S113. NOESY NMR (500 MHz, $CDCl_3$) spectrum of **7** (expansion)................ 56
Figure S114. IR spectrum of compound **8.**... 57
Figure S115. HRESIMS Spectrum of **8** ($[M + Na]^+$ positive ion mode)....................... 57
Figure S116. ^{1}H NMR (500 MHz, $CDCl_3$) spectrum of **8.**... 58
Figure S117. ^{1}H NMR (500 MHz, $CDCl_3$) spectrum of **8** (expansion)........................ 59
Figure S118. ^{1}H NMR (500 MHz, $CDCl_3$) spectrum of **8** (expansion)........................ 59
Figure S119. ^{1}H NMR (500 MHz, $CDCl_3$) spectrum of **8** (expansion)........................ 60
Figure S120. ^{13}C NMR - BB (125 MHz, $CDCl_3$) spectrum of **8.**................................. 60
Figure S121. ^{13}C NMR – DEPT 135 (125 MHz, $CDCl_3$) spectrum of **8**....................... 61
Figure S122. ^{13}C NMR – DEPT 135 (125 MHz, $CDCl_3$) spectrum of **8** (expansion)... 61
Figure S123. HSQC NMR (500 X 125 MHz, $CDCl_3$) spectrum of **8** 62
Figure S124. HSQC NMR (500 X 125 MHz, $CDCl_3$) spectrum of **8** (expansion)........ 62
Figure S125. HMBC NMR (500 X 125 MHz, $CDCl_3$) spectrum of **8** 63
Figure S126. HMBC NMR (500 X 125 MHz, $CDCl_3$) spectrum of **8** (expansion). 63
Figure S127. HMBC NMR (500 X 125 MHz, $CDCl_3$) spectrum of **8** (expansion). 64
Figure S128. HMBC NMR (500 X 125 MHz, $CDCl_3$) spectrum of **8** (expansion). 64
Figure S129. COSY NMR (500 MHz, $CDCl_3$) spectrum of **8** 65
Figure S130. COSY NMR (500 MHz, $CDCl_3$) spectrum of **8** (expansion). 65
Figure S131. NOESY NMR (500 MHz, $CDCl_3$) spectrum of **8**.................................. 66
Figure S132. IR spectrum of compound **9**.. 66
Figure S133. HRESIMS Spectrum of **9** ($[2M + Na]^+$ positive ion mode).................... 67
Figure S134. ^{1}H NMR (500 MHz, $CDCl_3$) spectrum of 9... 67
Figure S135. ^{1}H NMR (500 MHz, $CDCl_3$) spectrum of **9** (expansion)........................ 68
Figure S136. ^{1}H NMR (500 MHz, $CDCl_3$) spectrum of **9** (expansion)........................ 68
Figure S137. ^{1}H NMR (500 MHz, $CDCl_3$) spectrum **9** (expansion)............................. 69
Figure S138. ^{1}H NMR (500 MHz, $CDCl_3$) spectrum of **9** (expansion)........................ 69
Figure S139. ^{13}C NMR - BB (125 MHz, $CDCl_3$) spectrum of **9**. 70
Figure S140. ^{13}C NMR – DEPTQ 135 (125 MHz, $CDCl_3$) spectrum of **9.**.................. 70

Figure S141. HSQC NMR (500 X 125 MHz, $CDCl_3$) spectrum of **9.** 71

Figure S142. HSQC NMR (500 X 125 MHz, $CDCl_3$) spectrum of **9** (expansion)........ 71

Figure S143. HMBC NMR (500 X 125 MHz, $CDCl_3$) spectrum of **9.** 72

Figure S144. HMBC NMR (500 X 125 MHz, $CDCl_3$) spectrum of **9** (expansion). 72

Figure S145. HMBC NMR (500 X 125 MHz, $CDCl_3$) spectrum of **9** (expansion). 73

Figure S146. HMBC NMR (500 X 125 MHz, $CDCl_3$) spectrum of **9** (expansion). 73

Figure S147. COSY NMR (500 MHz, $CDCl_3$) spectrum of **9.** 74

Figure S148. COSY NMR (500 MHz, $CDCl_3$) spectrum of **9** (expansion). 74

Figure S149. NOESY NMR (500 MHz, $CDCl_3$) spectrum of **9.**................................. 75

Figure S150. Results of DP4+ analysis of **1**. 76

Table S1. Comparison of ^{13}C NMR (δ_{calc}) chemical shifts simulated for all candidates of 1 (GIAO-mPW1PW91/6-31+G(d,p)//B3LYP/6-31G(d)) with the experimental values of the isolated natural product... 76

Table S2. Comparison of ^{1}H NMR (δ_{calc}) chemical shifts simulated for all candidates of 1 (GIAO-mPW1PW91/6-31+G(d,p)//B3LYP/6-31G(d)) with the experimental values of the isolated natural product... 77

Figure S151. Comparison of the observed ECD spectrum of compound **1** in ACN (black solid line) with the calculated [CAM-B3LYP/PCM(ACN)/TZVP] ECD spectra of the Boltzmann average of the 8 lowest-energy conformers identified candidate 1h (2R,3R,5R,7S,10S) (blue solid line) and its enantiomer (2S,3S,5S,7R,10R) (red dashed line) of 1... 77

Figure S152. The 8 lowest energy conformers of candidate 1h at B3LYP/6-31G(d) level. 78

Figure S153. Results of DP4+ analysis of **2**. 79

Table S3. Comparison of ^{13}C NMR (δ_{calc}) chemical shifts simulated for all candidates of 2 (GIAO-mPW1PW91/6-31+G(d,p)//B3LYP/6-31G(d)) with the experimental values of the isolated natural product... 79

Table S4. Comparison of ^{1}H NMR (δ_{calc}) chemical shifts simulated for all candidates of 2 (GIAO-mPW1PW91/6-31+G(d,p)//B3LYP/6-31G(d)) with the experimental values of the isolated natural product... 80

Figure S154. Comparison of the observed ECD spectrum of compound 2 in ACN (black solid line) with the calculated [CAM-B3LYP/PCM(ACN)/TZVP] ECD spectra of the Boltzmann average of the 15 lowest-energy conformers identified candidate 2d (2R,3R,5R,10S) (blue solid line) and its enantiomer (2S,3S,5S,10R) (red dashed line) of 2. 80

Figure S155. The 15 lowest energy conformers of candidate 2d at B3LYP/6-31G(d) level. 81

Figure S156. Results of DP4+ analysis of 3. 82

Table S5. Comparison of ^{13}C NMR (δ_{calc}) chemical shifts simulated for all candidates of 3 (GIAO-mPW1PW91/6-31+G(d,p)//B3LYP/6-31G(d)) with the experimental values of the isolated natural product.. 82

Table S6. Comparison of ^{1}H NMR (δ_{calc}) chemical shifts simulated for all candidates of 3 (GIAO-mPW1PW91/6-31+G(d,p)//B3LYP/6-31G(d)) with the experimental values of the isolated natural product.. 83

Figure S157. Comparison of the observed ECD spectrum of **3** in ACN (black trace) with the calculated [CAM-B3LYP/PCM(ACN)/TZVP] ECD spectrum of the Boltzmann average of the 14 lowest-energy identified candidate 3d (2R,3R,5R,10S) (blue solid line) and its enantiomer (2S,3S,5S,10R) (red dashed line) of **3**. 83

Figure S158. The 14 lowest energy conformers of candidate 3d at B3LYP/6-31G(d) level. 84

Figure S159. Results of DP4+ analysis of **4**. 85

Table S7. Comparison of ^{13}C NMR (δ_{calc}) and ^{1}H NMR chemical shifts simulated for all candidates of 4 (GIAO-mPW1PW91/6-31+G(d,p)//B3LYP/6-31G(d)) with the experimental values of the isolated natural product. 85

Figure S160. Comparison of the observed ECD spectrum of 4 in ACN (black solid line) with the calculated [CAM-B3LYP/PCM(ACN)/TZVP] ECD spectrum of the Boltzmann average of the 12 lowest-energy conformers identified candidate 4b (2S,5S,10R) (blue solid line) and its enantiomer (2R,5R,10S) (red line) of 4. 86

Figure S161. The 12 lowest energy conformers of candidate 4b at B3LYP/6-31G(d) level. 86

Figure S162. Results of DP4+ analysis of **5**. 87

Table S8. Comparison of ^{13}C NMR (δ_{calc}) and ^{1}H NMR chemical shifts simulated for all candidates of 5 (GIAO-mPW1PW91/6-31+G(d,p)//B3LYP/6-31G(d)) with the experimental values of the isolated natural product. 87

Figure S163. Comparison of the observed ECD spectrum of 5 in ACN (black trace) with the calculated [CAM-B3LYP/PCM(ACN)/TZVP] ECD spectrum of the Boltzmann average of the 12 lowest-energy conformers identified for candidate 5b (2S,5S,10R) (blue solid line) and its enantiomer (2R,5R,10S) (red dashed line) of 5. 88

Figure S164. The 12 lowest energy conformers of candidate 5b at B3LYP/6-31G(d) level. 88

Figure S165. Results of DP4+ analysis of 6. 89

Table S9. Comparison of ^{13}C NMR (δ_{calc}) and ^{1}H NMR chemical shifts simulated for all candidates of 6 (GIAO-mPW1PW91/6-31+G(d,p)//B3LYP/6-31G(d)) with the experimental values of the isolated natural product. 89

Figure S166. Comparison of the observed ECD spectrum of 6 in ACN (black trace) with the calculated [CAM-B3LYP/PCM(ACN)/TZVP] ECD spectrum of the Boltzmann average of the 12 lowest-energy conformers identified for candidate 6b (2S,5S,10R) (blue solid line) and its enantiomer (2R,5R,10S) (red dashed line) of 6. 90

Figure S167. The 12 lowest energy conformers of candidate 6b at B3LYP/6-31G(d) level. 90

Figure S168. Results of DP4+ analysis of 7. 91

Table S10. Comparison of ^{13}C NMR (δ_{calc}) and ^{1}H NMR chemical shifts simulated for all candidates of 7 (GIAO-mPW1PW91/6-31+G(d,p)//B3LYP/6-31G(d)) with the experimental values of the isolated natural product. 91

Figure S169. Comparison of the observed ECD spectrum of 7 in ACN (black trace) with the calculated [CAM-B3LYP/PCM(ACN)/TZVP] ECD spectrum of the Boltzmann average of the 16 lowest-energy conformers identified for candidate 7b (2S,5S,7S,10R) (blue solid line) and its enantiomer (2R,5R,7R,10S) (red dashed line) of 7. 92

Figure S170. The 16 lowest energy conformers of candidate 7b at B3LYP/6-31G(d) level. 92

Figure S171. Results of DP4+ analysis of 8. 93

Table S11. Comparison of ^{13}C NMR (δ_{calc}) and ^{1}H NMR chemical shifts simulated for all candidates of 8 (GIAO-mPW1PW91/6-31+G(d,p)//B3LYP/6-31G(d)) with the experimental values of the isolated natural product. 93

Figure S172. Comparison of the observed ECD spectrum of 8 in ACN (black trace) with the calculated [CAM-B3LYP/PCM(ACN)/TZVP] ECD spectrum of the Boltzmann average of the 19 lowest-energy conformers identified for candidate 8b (2S,5S,6S,10R) (blue solid line) and its enantiomer (2R,5R,6R,10S) (red dashed line) of 8. 94

Figure S173. The 19 lowest energy conformers of candidate 8b at B3LYP/6-31G(d) level. 95

Figure S174. Results of DP4+ analysis of 9. 97

Table S12. Comparison of ^{13}C NMR (δ_{calc}) and ^{1}H NMR chemical shifts simulated for all candidates of 9 (GIAO-mPW1PW91/6-31+G(d,p)//B3LYP/6-31G(d)) with the experimental values of the isolated natural product. 97

Figure S175. Comparison of the observed ECD spectrum of 9 in ACN (black trace) with the calculated [CAM-B3LYP/PCM(ACN)/TZVP] ECD spectrum of the Boltzmann average of the 18 lowest-energy conformers identified for candidate 9b (2S,5S,10R) (blue solid line) and its enantiomer (2R,5R, 10S) (red dashed line) of 9. 98

Figure S176. The 18 lowest energy conformers of candidate 9b at B3LYP/6-31G(d) level. 98

Figure S1. IR spectrum of compound 1

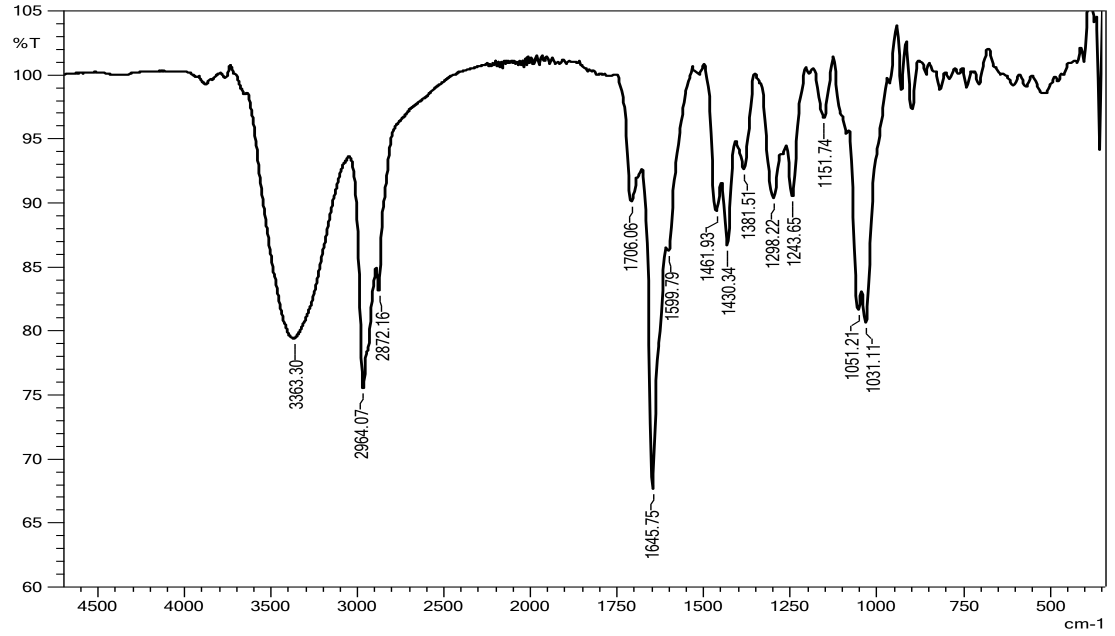

Figure S2. HRESIMS Spectrum of **1** ([M - H_2O + H]$^+$ positive ion mode).

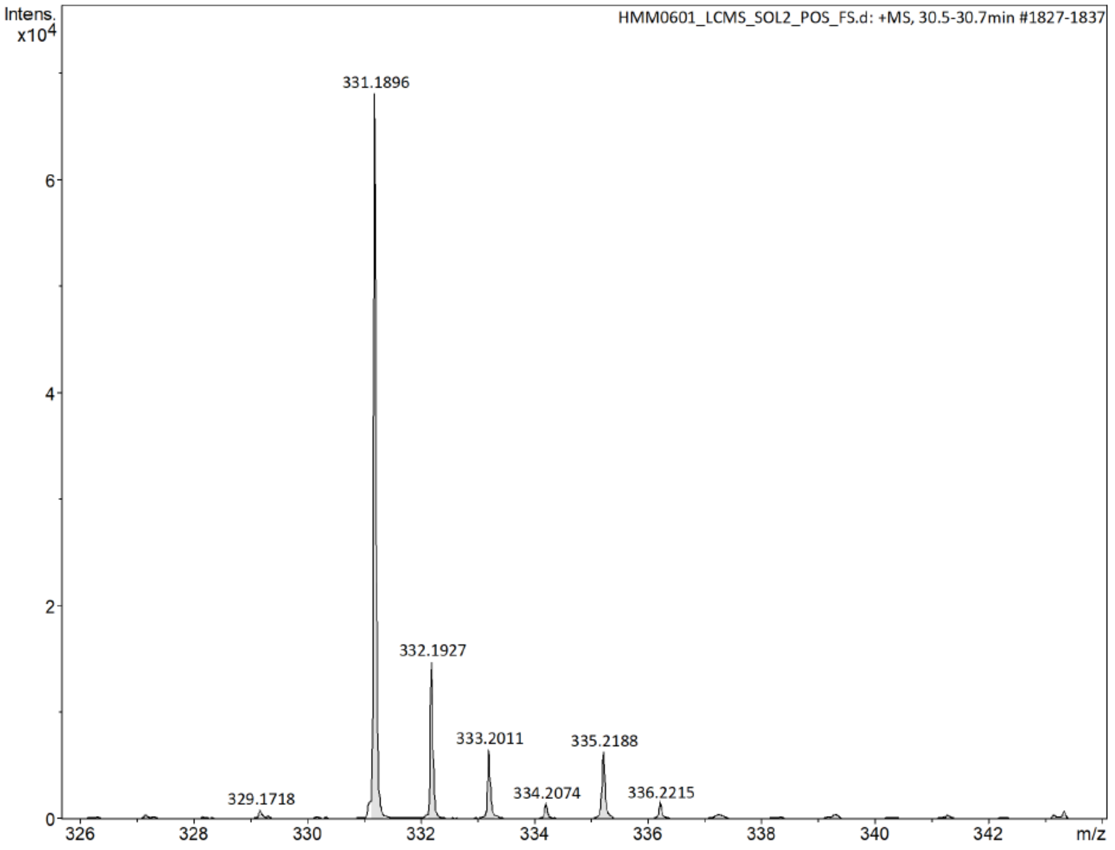

Figure S3. ^{1}H NMR (400 MHz, $CDCl_3$) spectrum of **1**

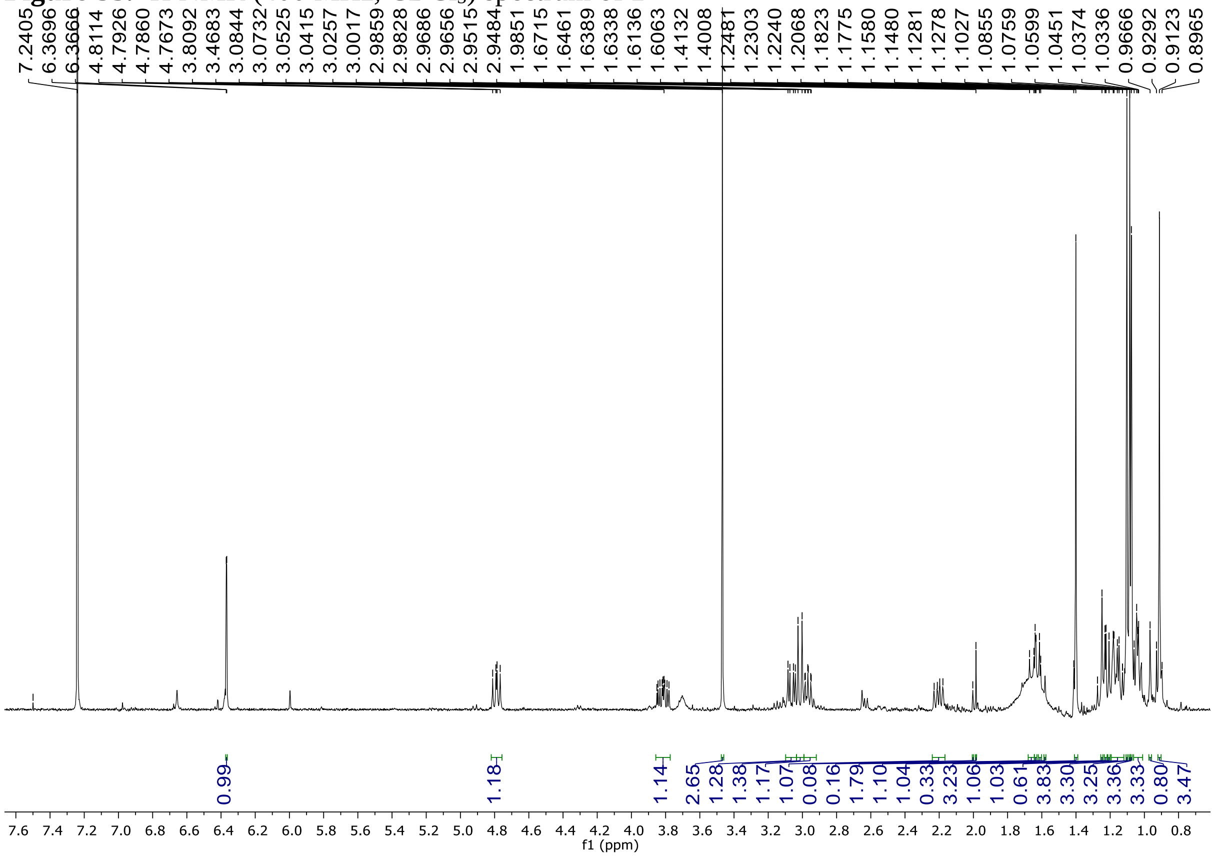

Figure S4. ^{1}H NMR (400 MHz, $CDCl_3$) spectrum of **1** (expansion).

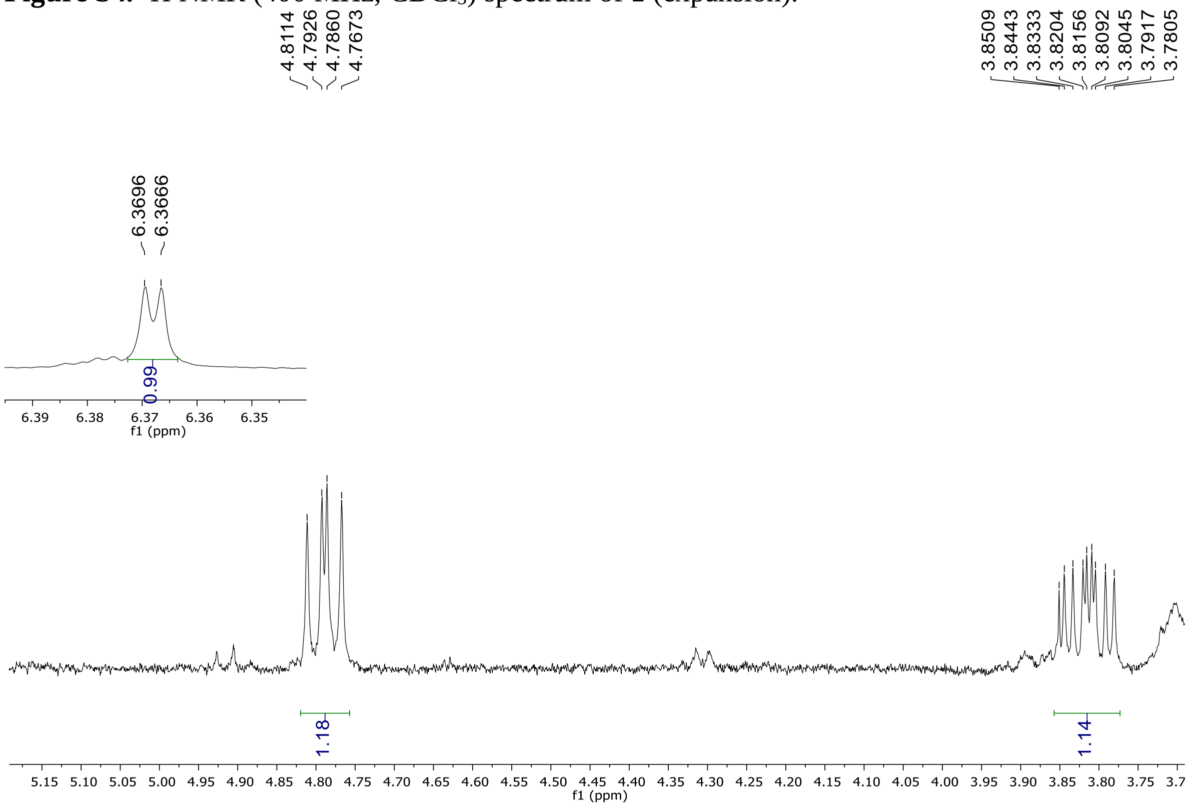

Figure S5. ^1H NMR (400 MHz, $CDCl_3$) spectrum of **1** (expansion).

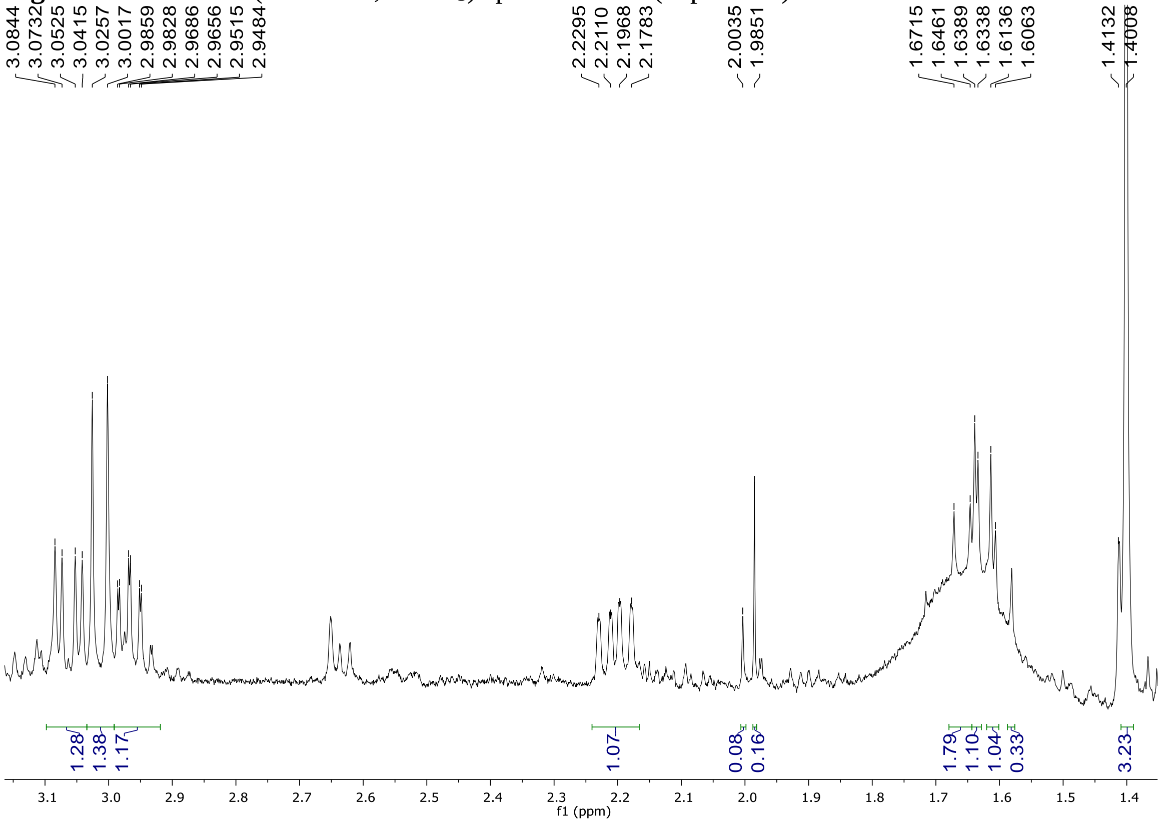

Figure S6. ^1H NMR (400 MHz, $CDCl_3$) spectrum of **1** (expansion).

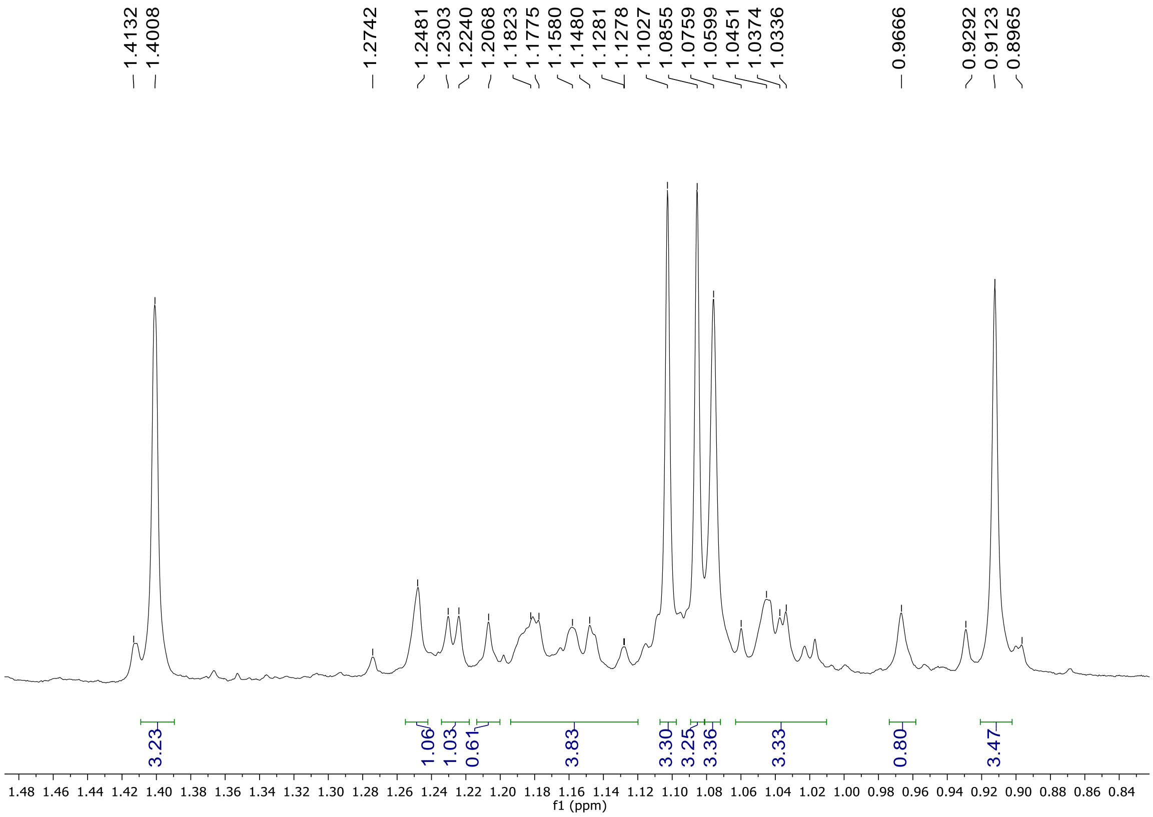

Figure S7. ^{13}C NMR - BB (125 MHz, $CDCl_3$) spectrum of **1**.

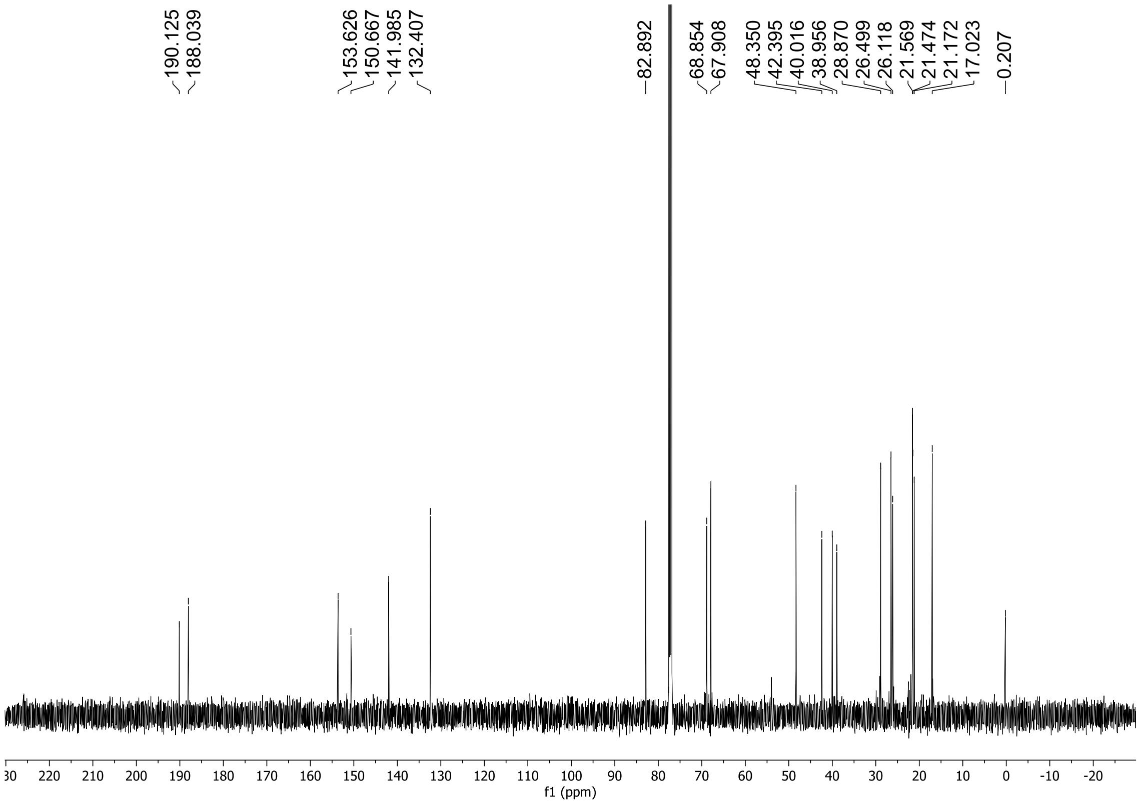

Figure S8. ^{13}C NMR - BB (125 MHz, $CDCl_3$) spectrum of **1** (expansion).

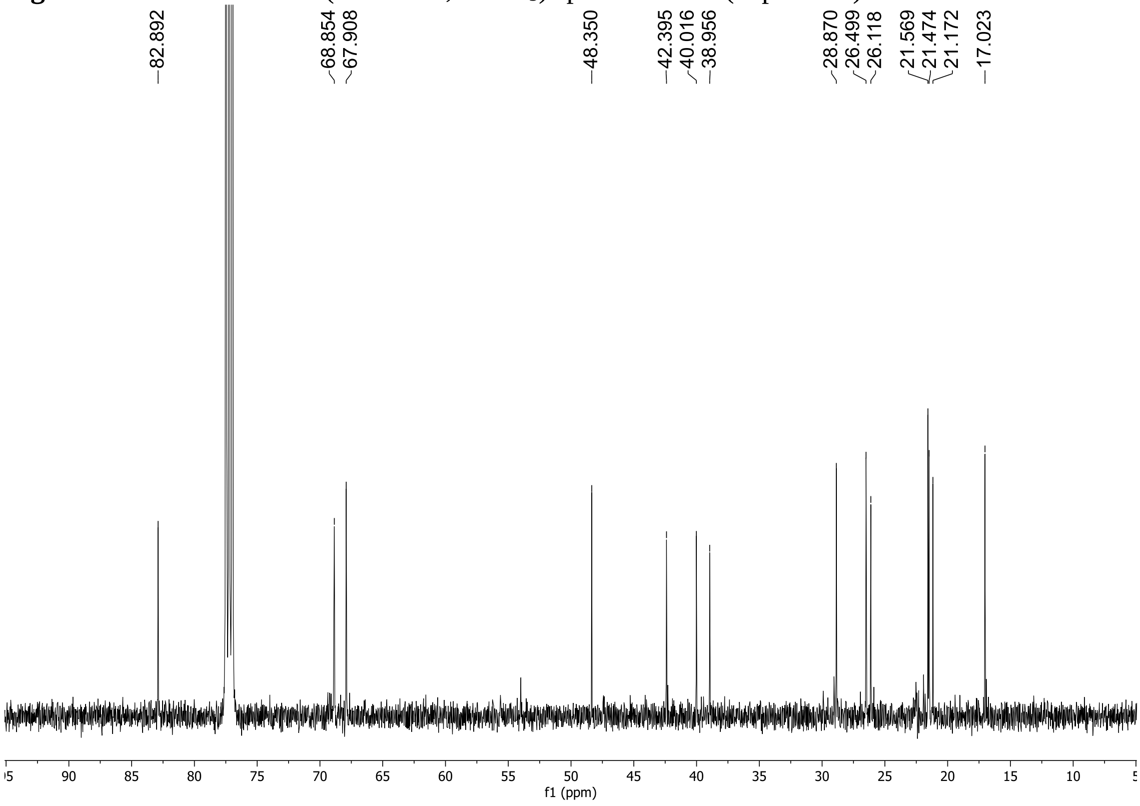

Figure S9. ^{13}C NMR – DEPT 135 (125 MHz, $CDCl_3$) spectrum of **1**

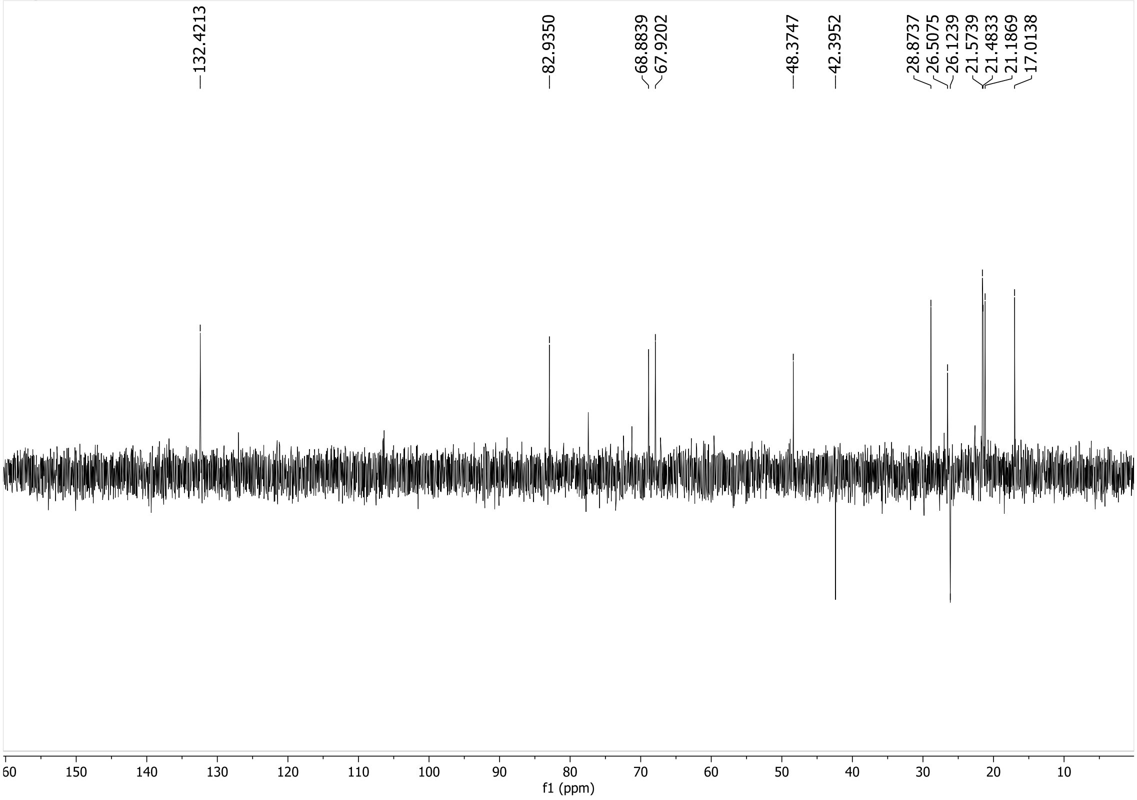

Figure S10. HSQC NMR (400 X 125 MHz, $CDCl_3$) spectrum of **1**

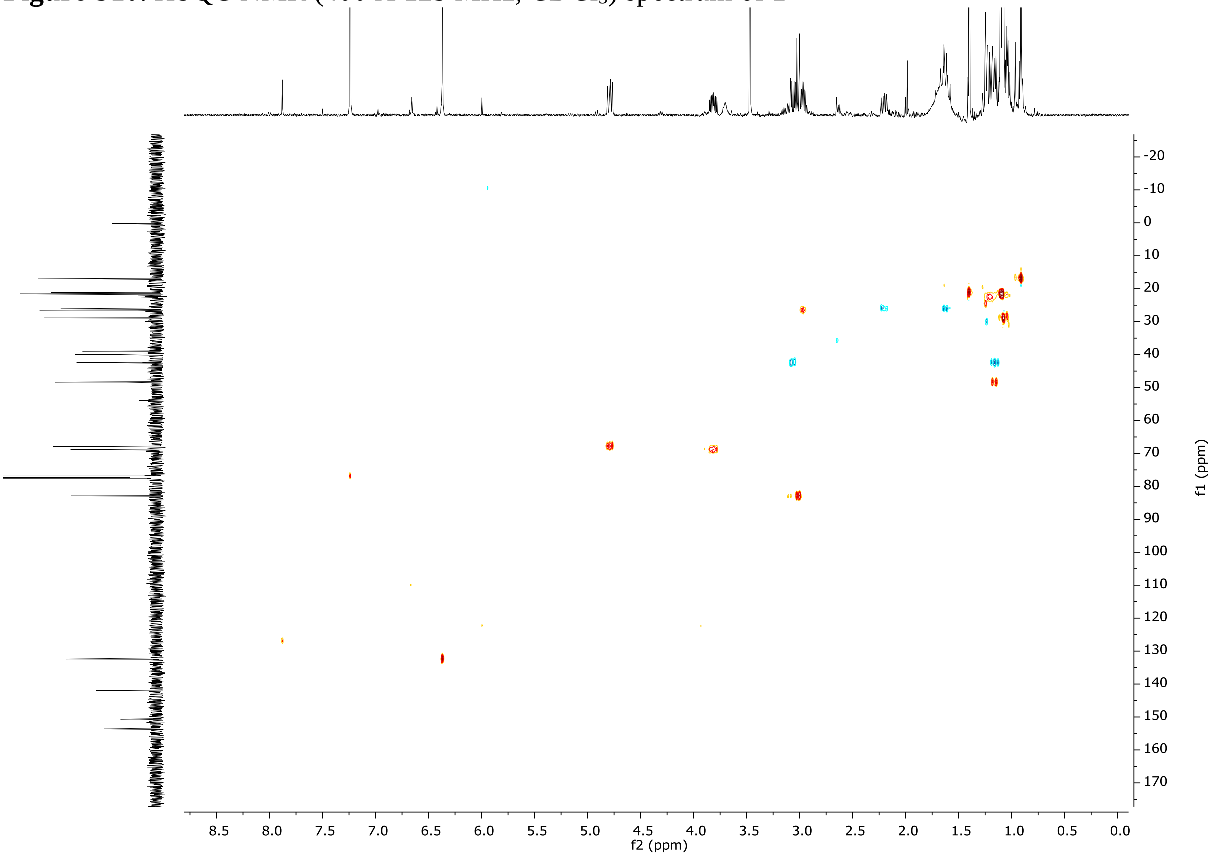

Figure S11. HSQC NMR (400 X 125 MHz, $CDCl_3$) spectrum of **1** (expansion).

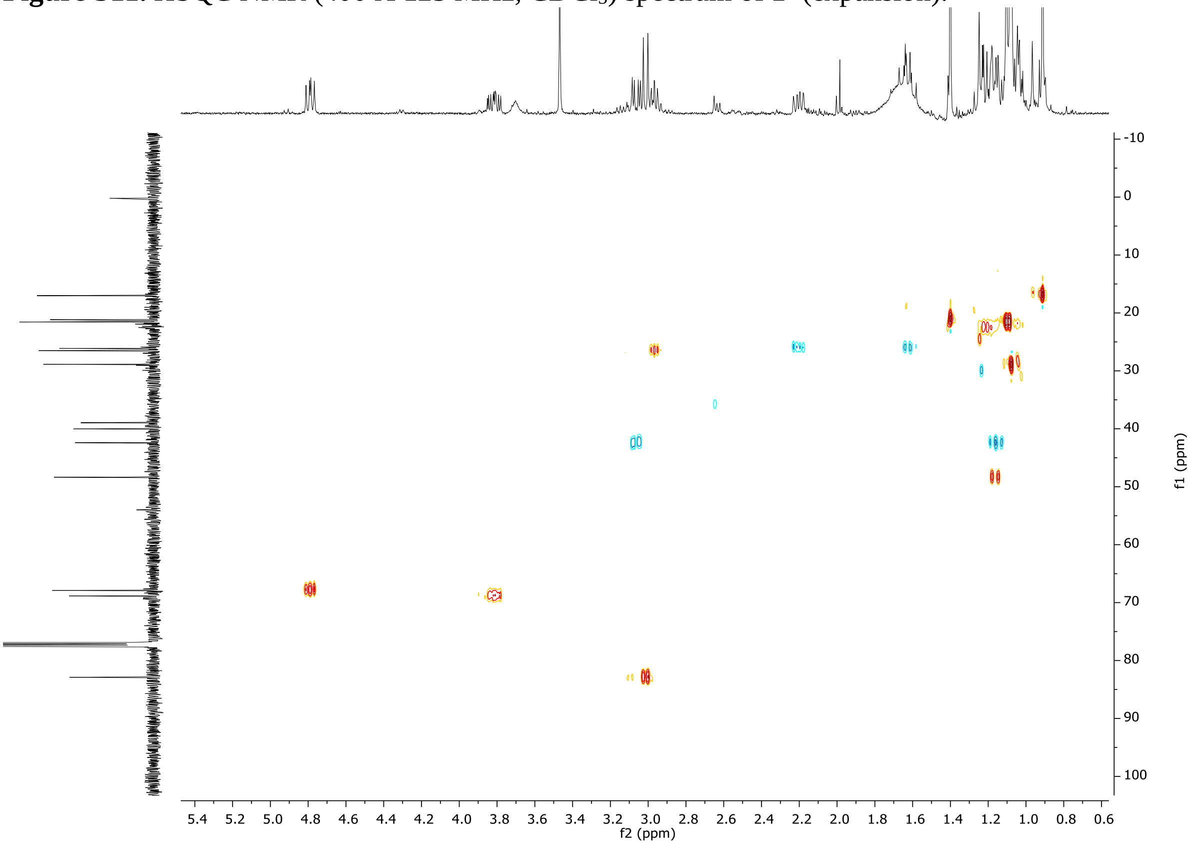

Figure S12. HMBC NMR (400 X 125 MHz, $CDCl_3$) spectrum of **1**

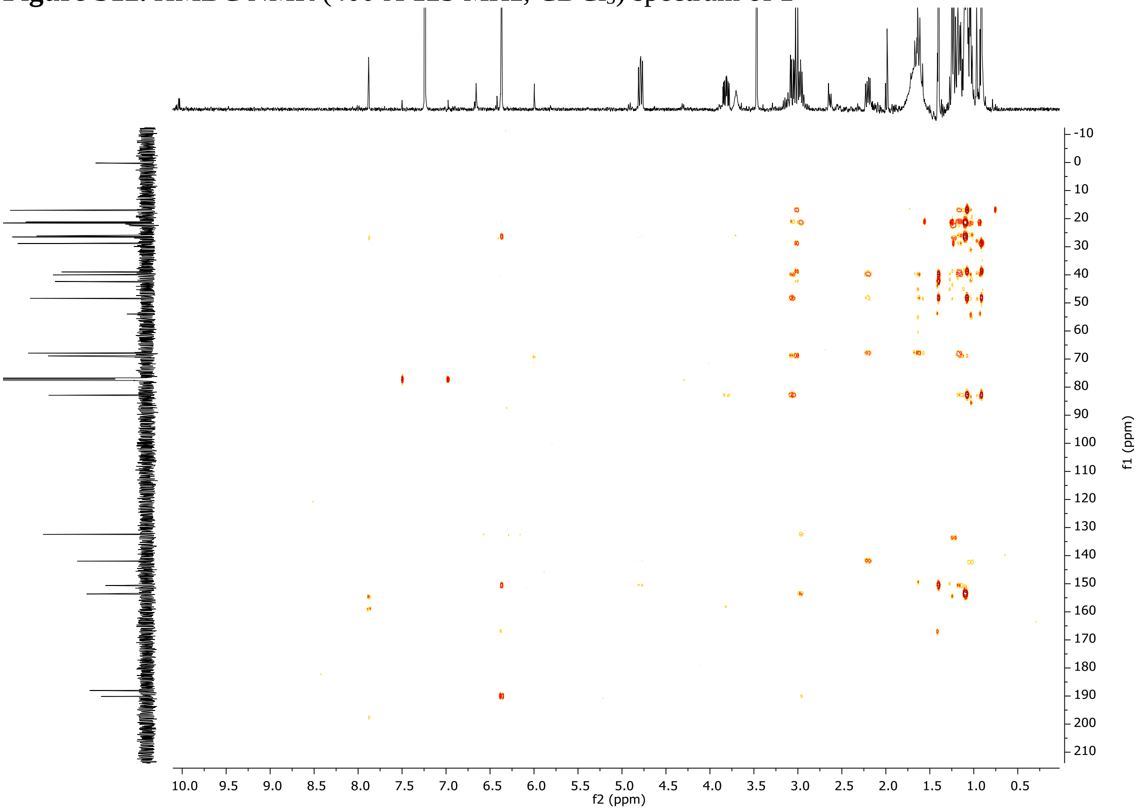

Figure S13. HMBC NMR (400 X 125 MHz, $CDCl_3$) spectrum of **1** (expansion).

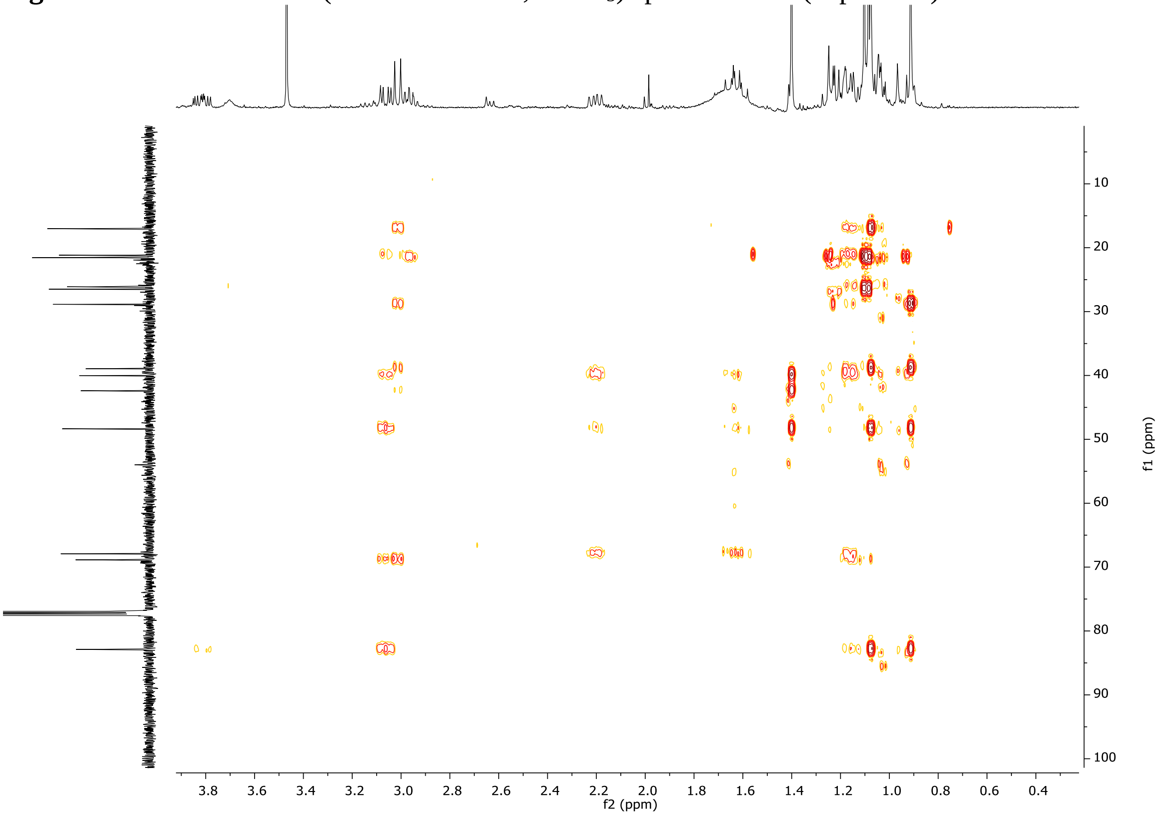

Figure S14. HMBC NMR (400 X 125 MHz, $CDCl_3$) spectrum of **1** (expansion).

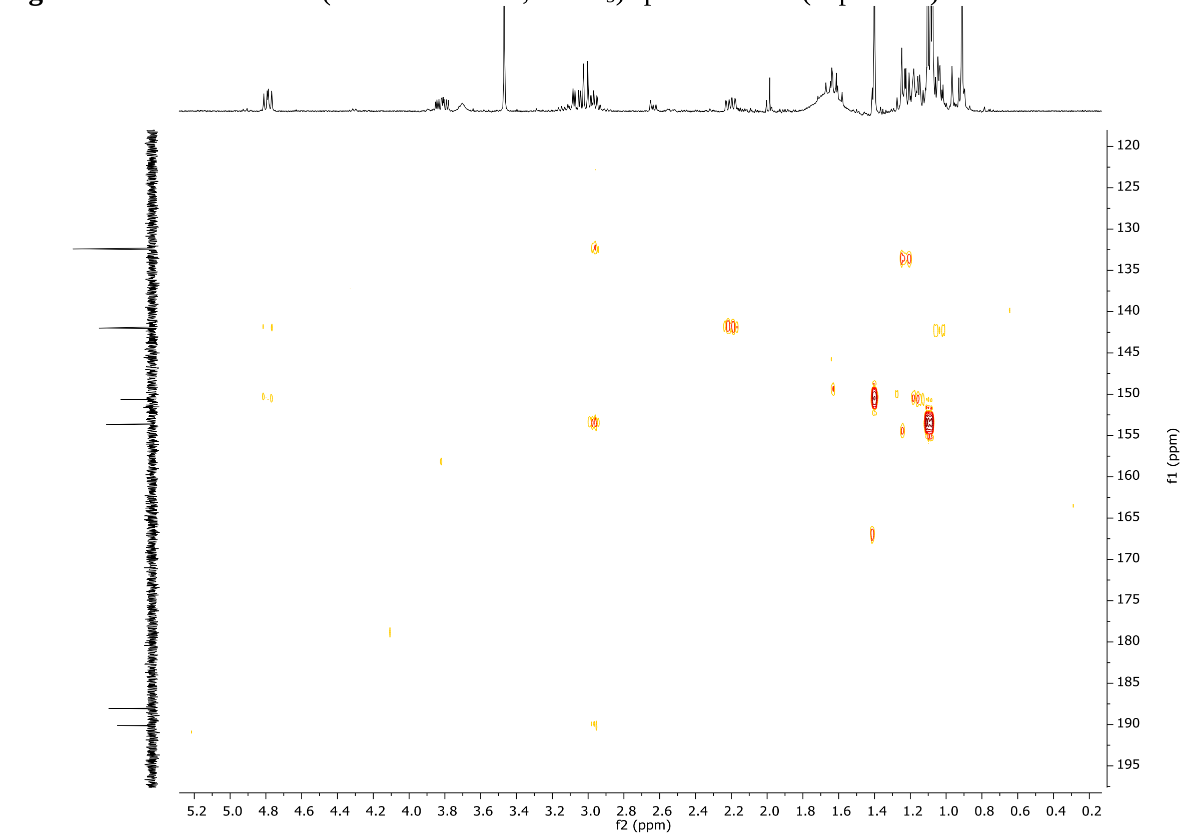

Figure S15. COSY NMR (400 MHz, $CDCl_3$) spectrum of **1.**

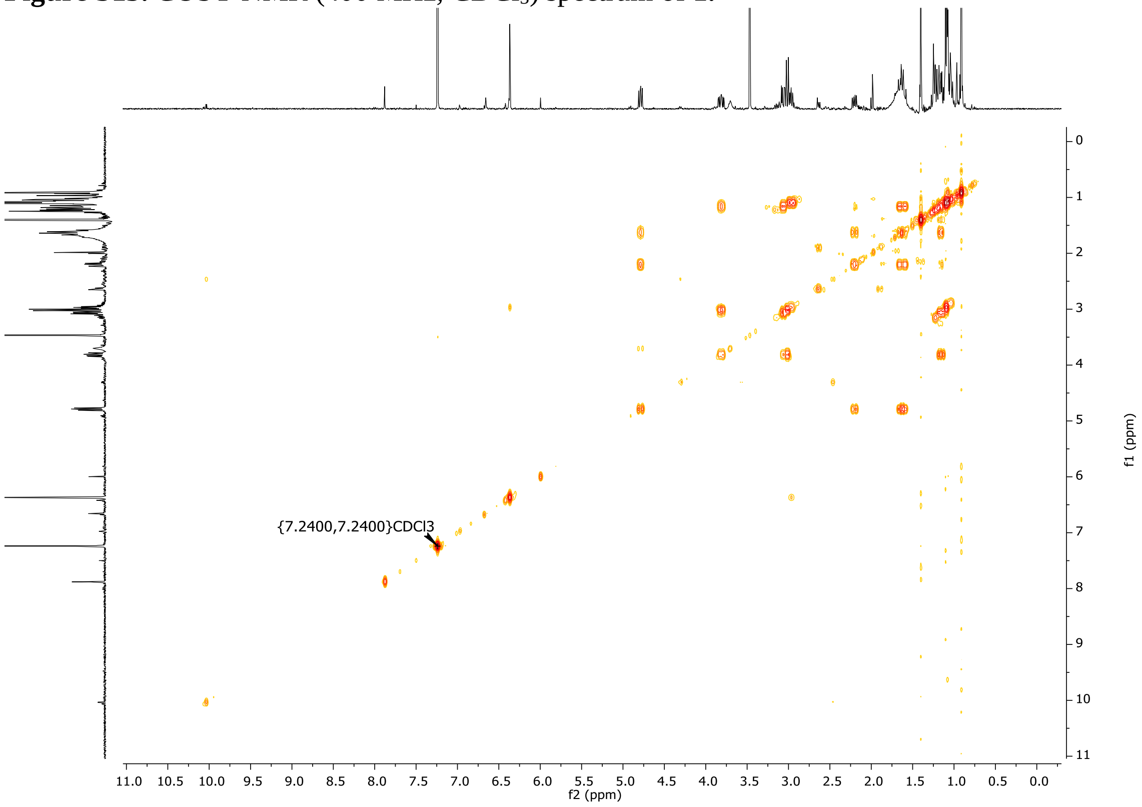

Figure S16. COSY NMR (400 MHz, $CDCl_3$) spectrum of **1** (expansion).

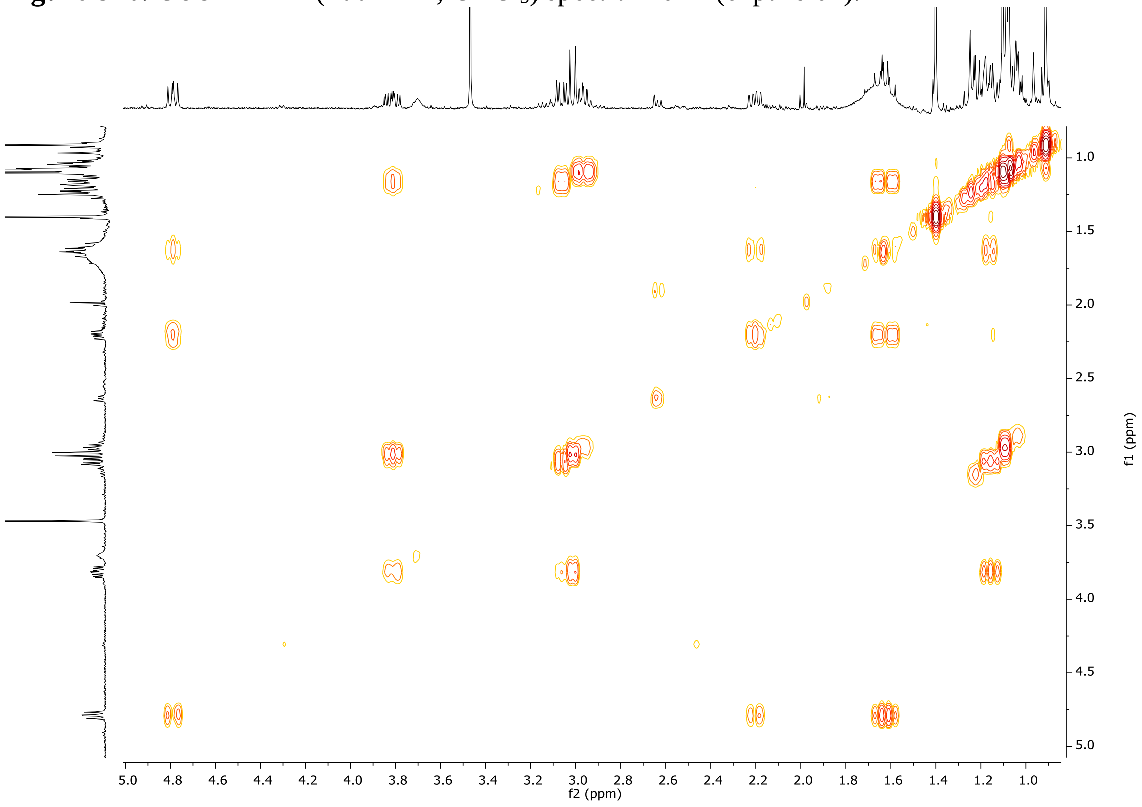

Figure S17. NOESY NMR (400 MHz, $CDCl_3$) spectrum of **1.**

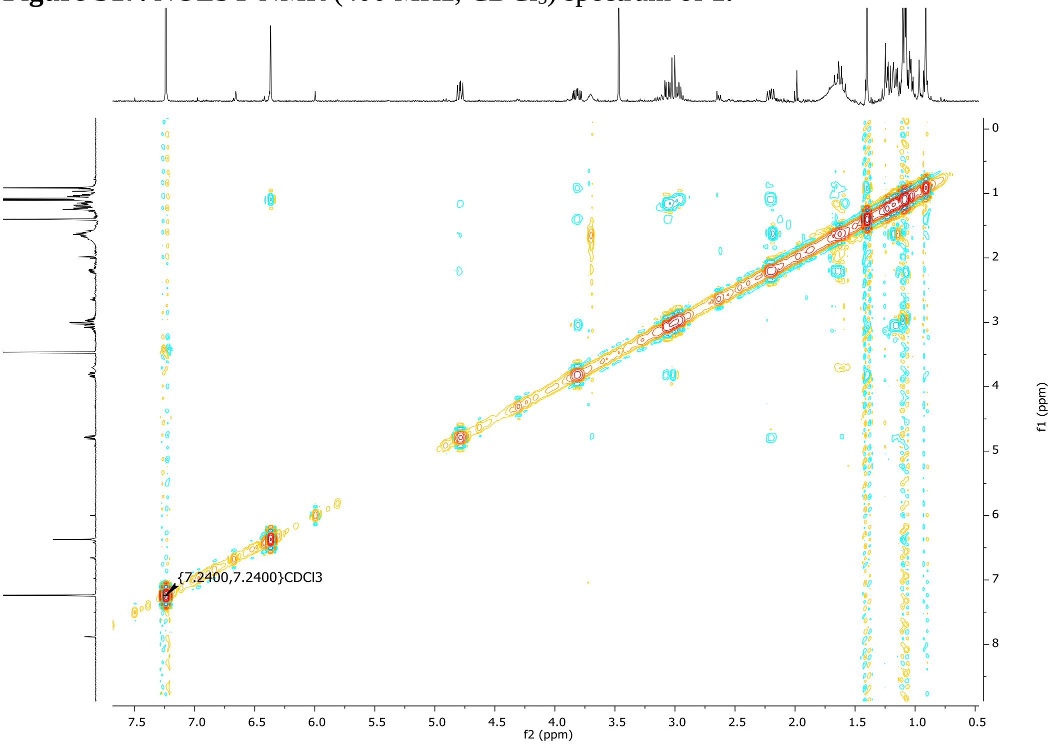

Figure S18. NOESY NMR (400 MHz, $CDCl_3$) spectrum of **1** (expansion).

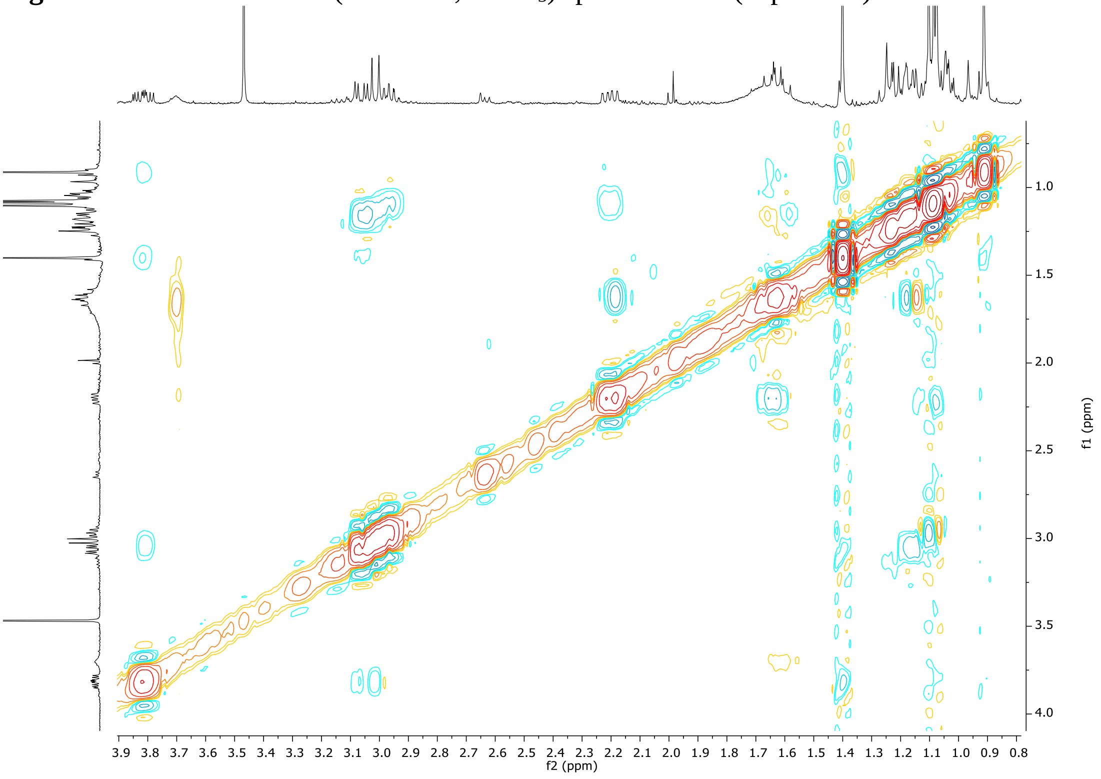

Figure S19. IR spectrum of compound **2.**

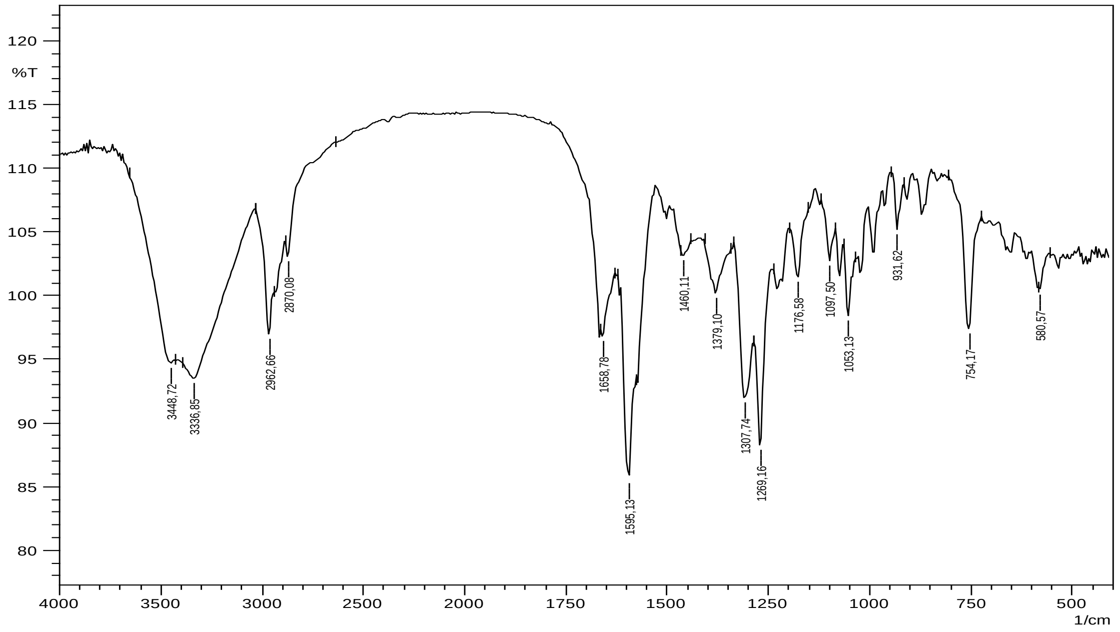

Figure S20. HRESIMS Spectrum of **2** ([2M + Na]$^+$ positive ion mode)

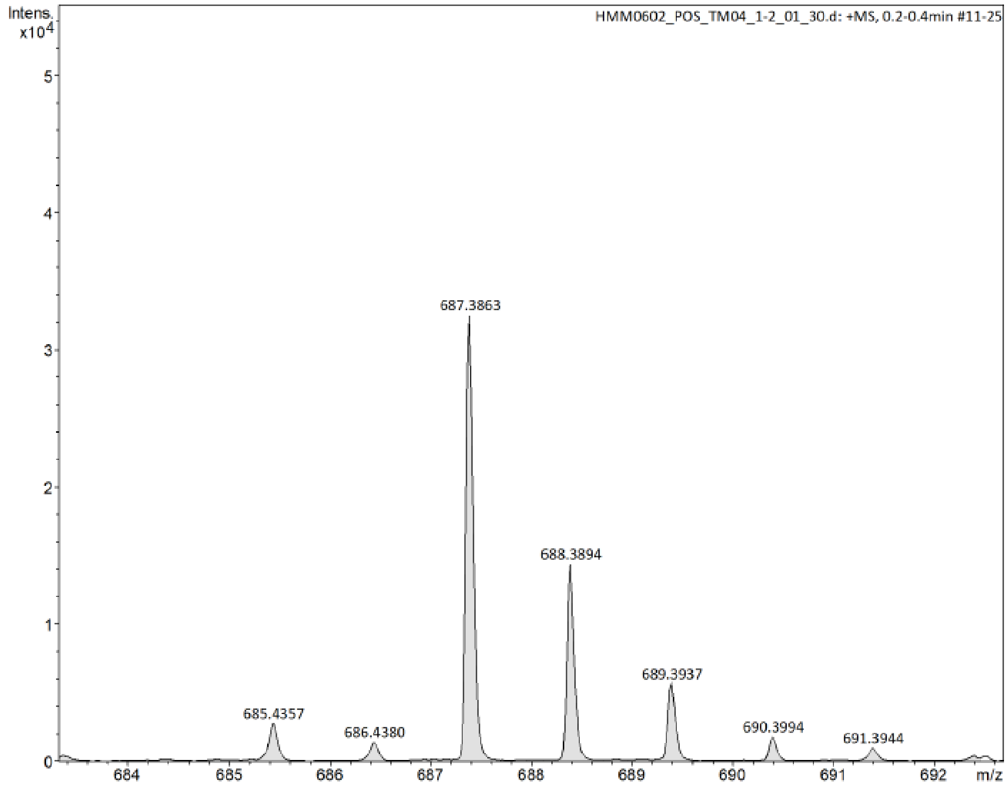

Figure S21. ^{1}H NMR (400 MHz, CD_3OD) spectrum of **2**

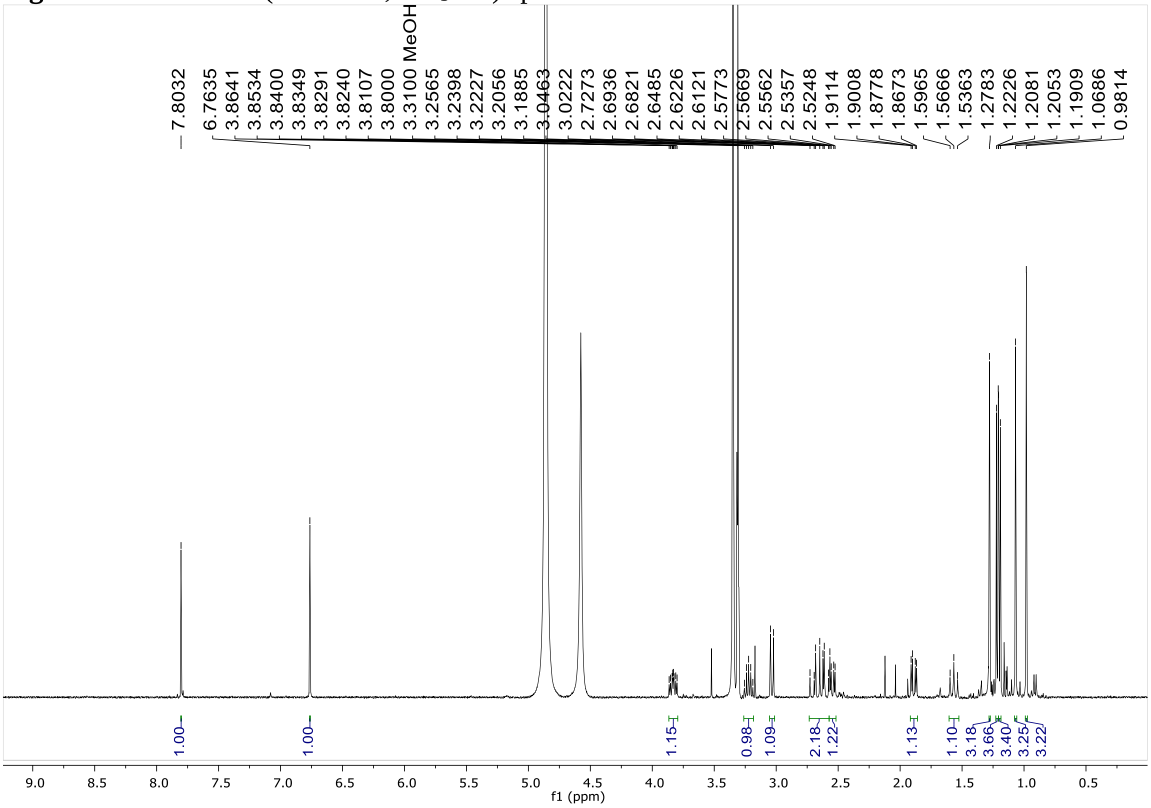

Figure S22. ^{1}H NMR (400 MHz, CD_3OD) spectrum of 2 (expansion).

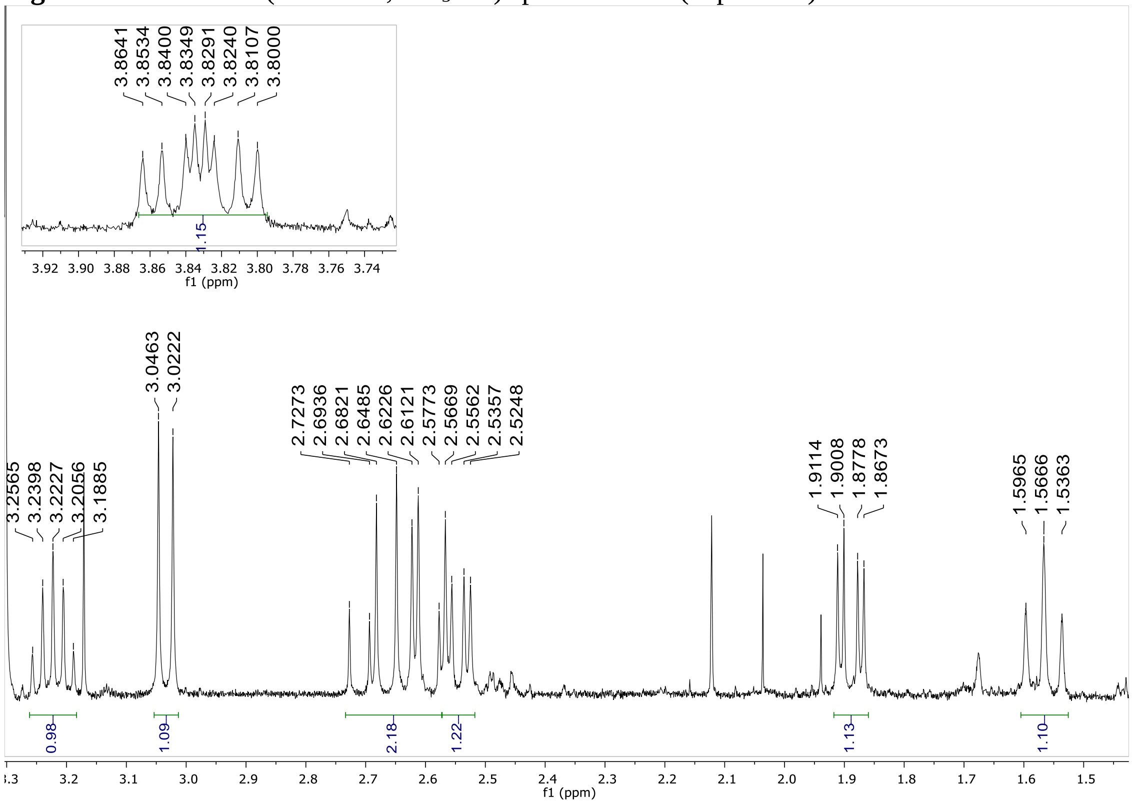

Figure S23. ^{1}H NMR (400 MHz, CD_3OD) spectrum of **2** (expansion).

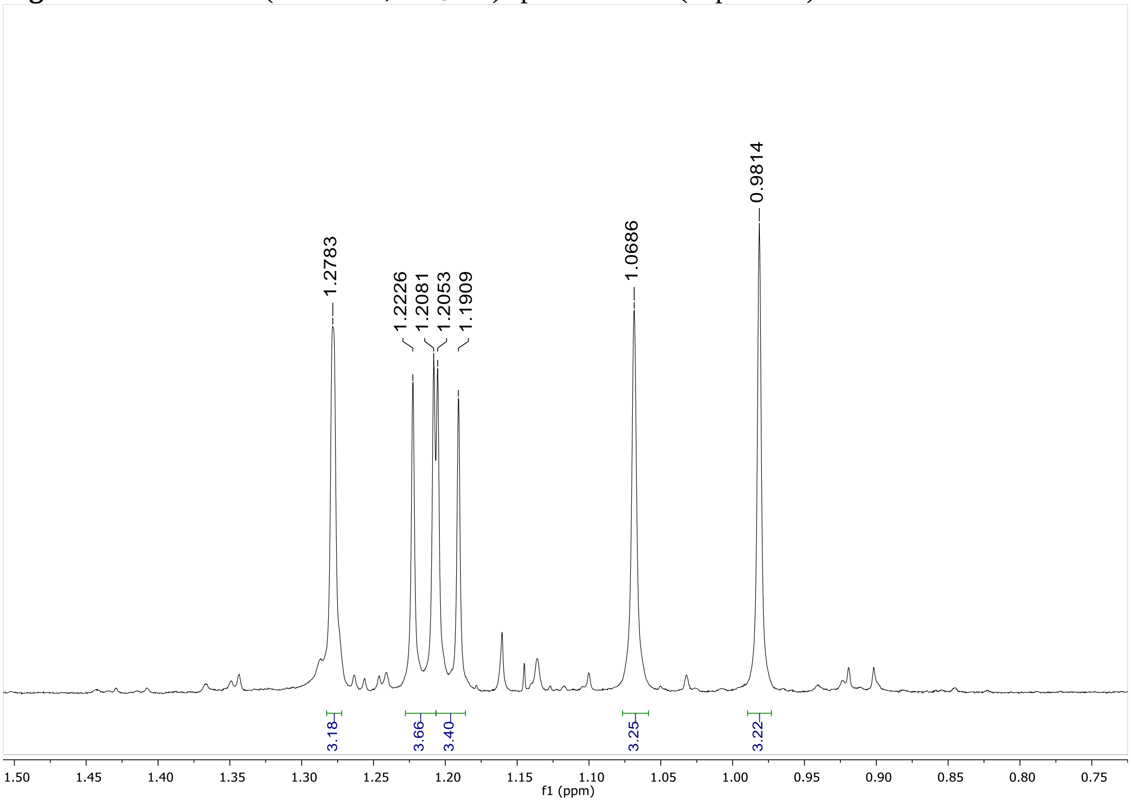

Figure S24. ^{13}C NMR - BB (100 MHz, CD_3OD) spectrum of **2.**

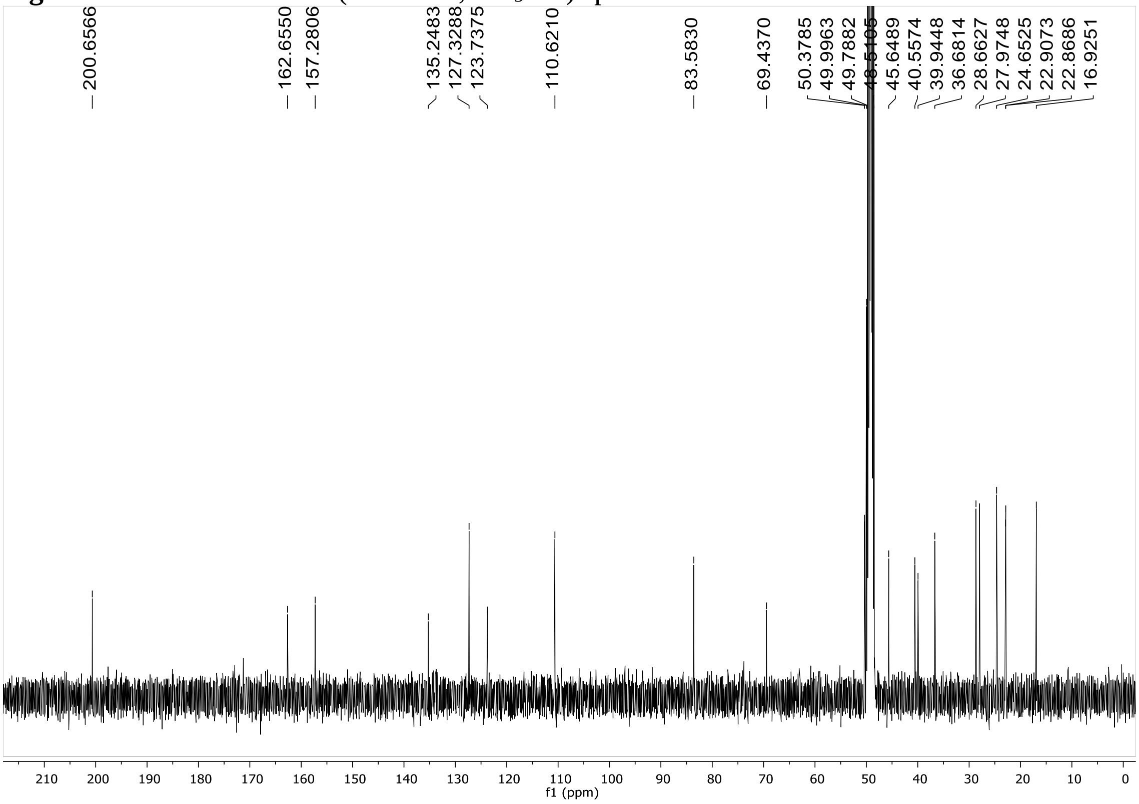

Figure S25. ^{13}C NMR - BB (100 MHz, CD_3OD) spectrum of **2** (expansion).

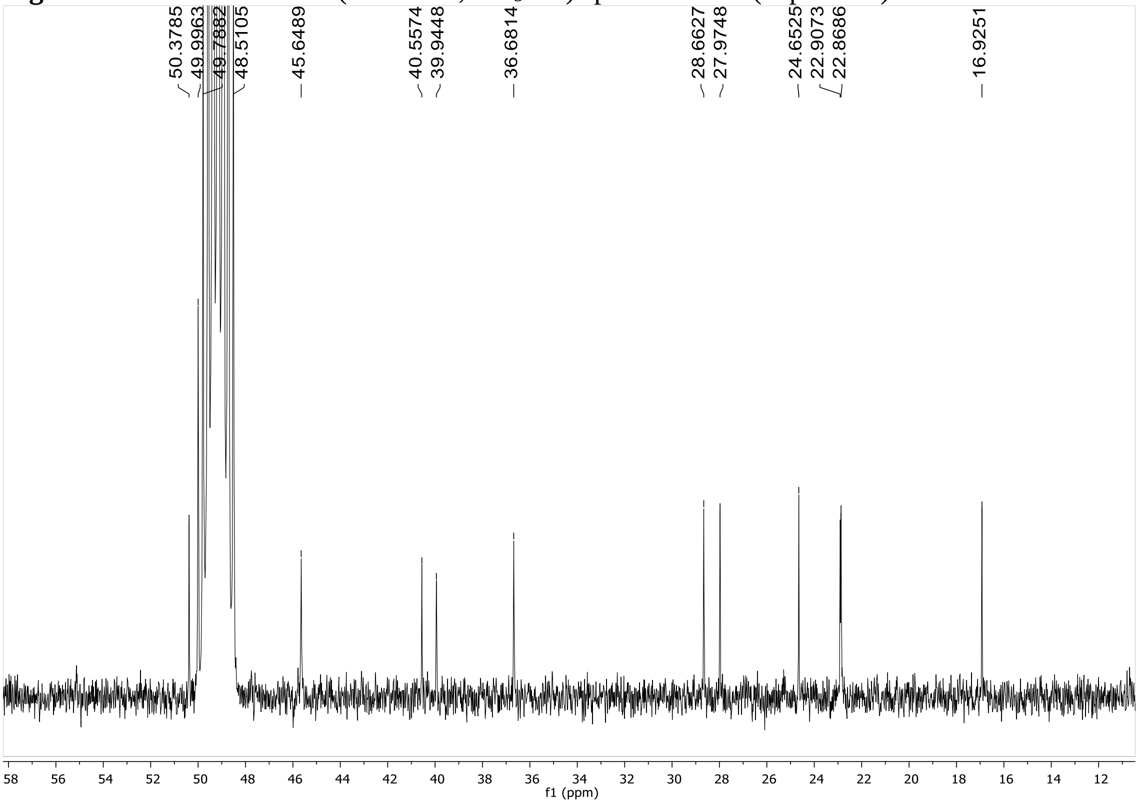

Figure S26. ^{13}C NMR – DEPT 135 (100 MHz, CD_3OD) spectrum of **2**

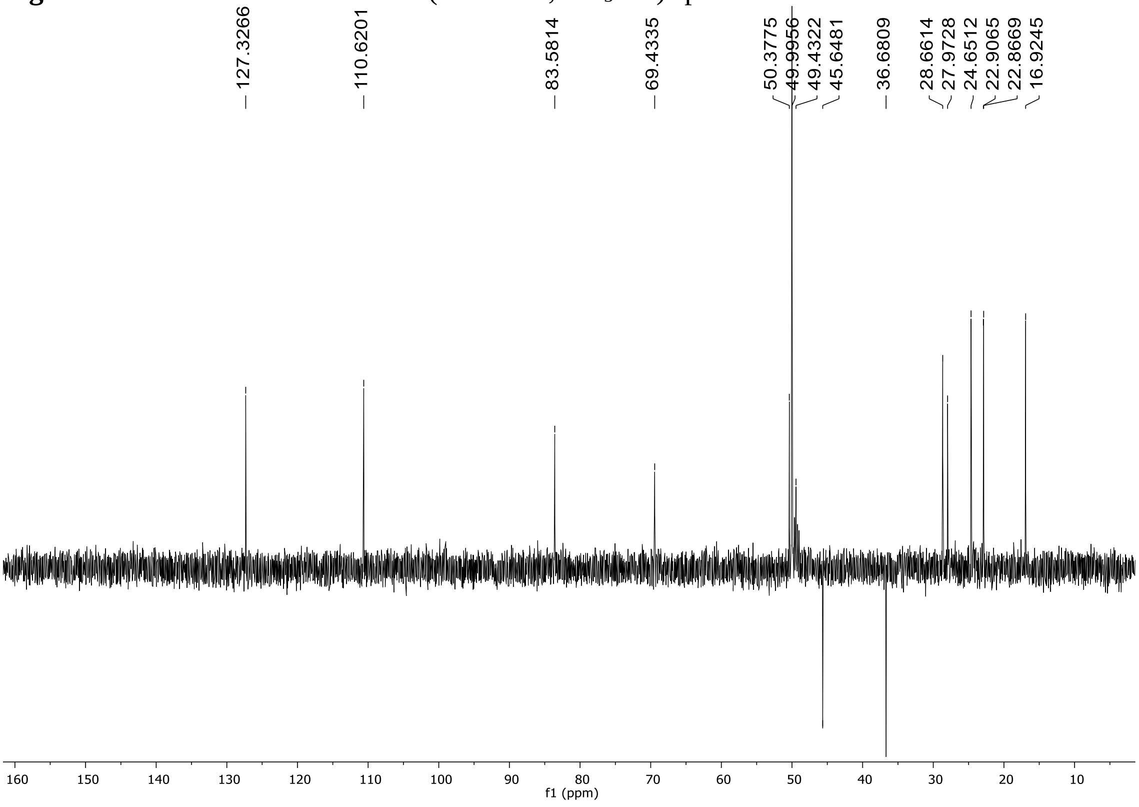

Figure S27. HSQC NMR (400 X 100 MHz, CD_3OD) spectrum of **2**

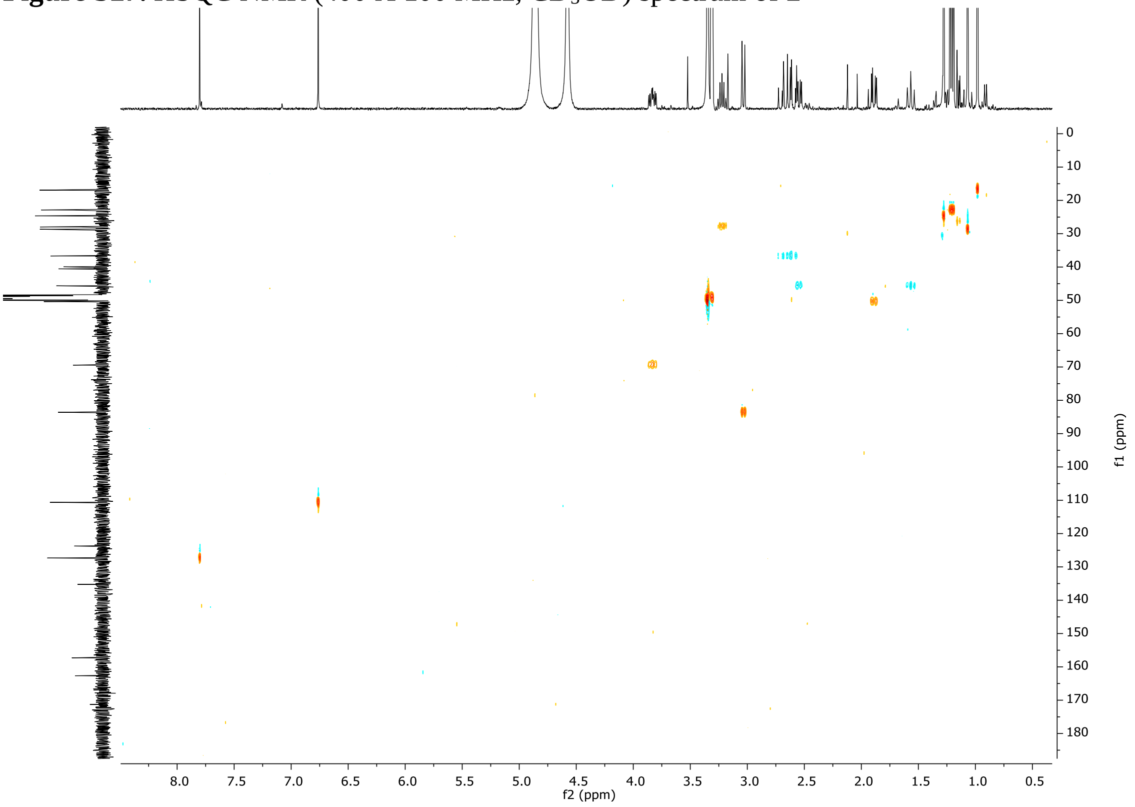

Figure S28. HMBC NMR (400 X 100 MHz, CD_3OD) spectrum of **2**

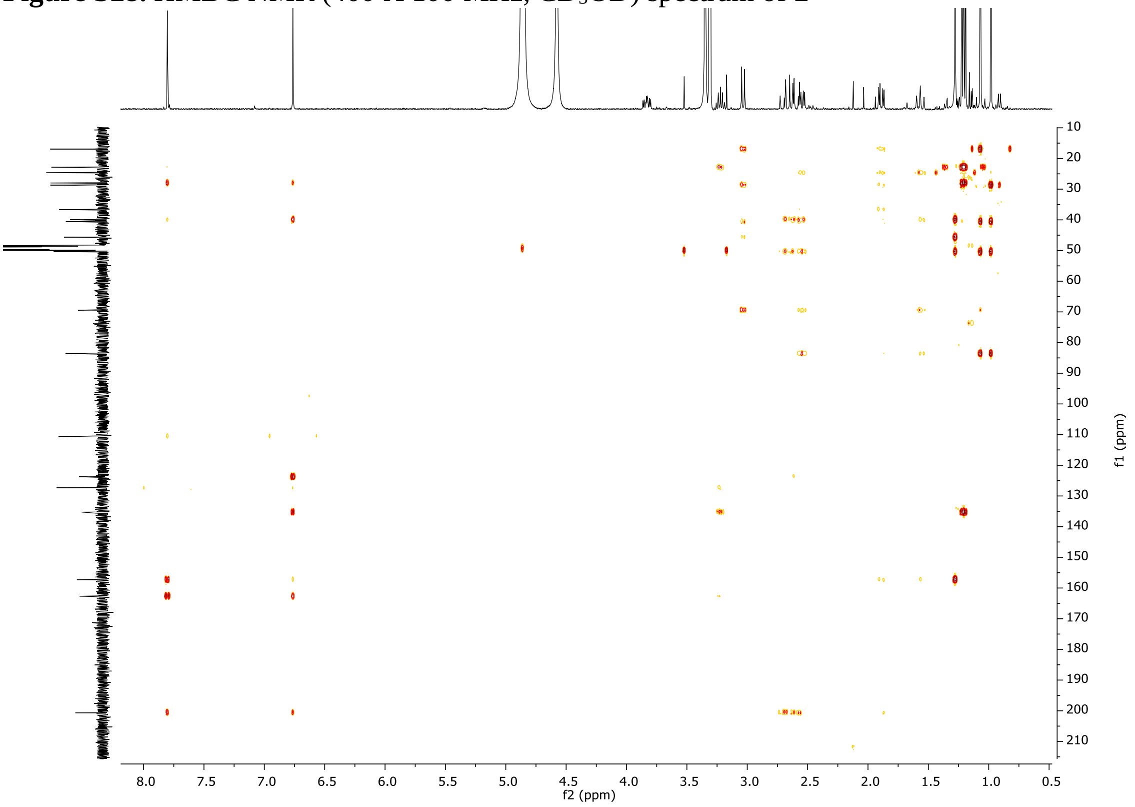

Figure S29. HMBC NMR (400 X 100 MHz, CD_3OD) spectrum of **2** (expansion).

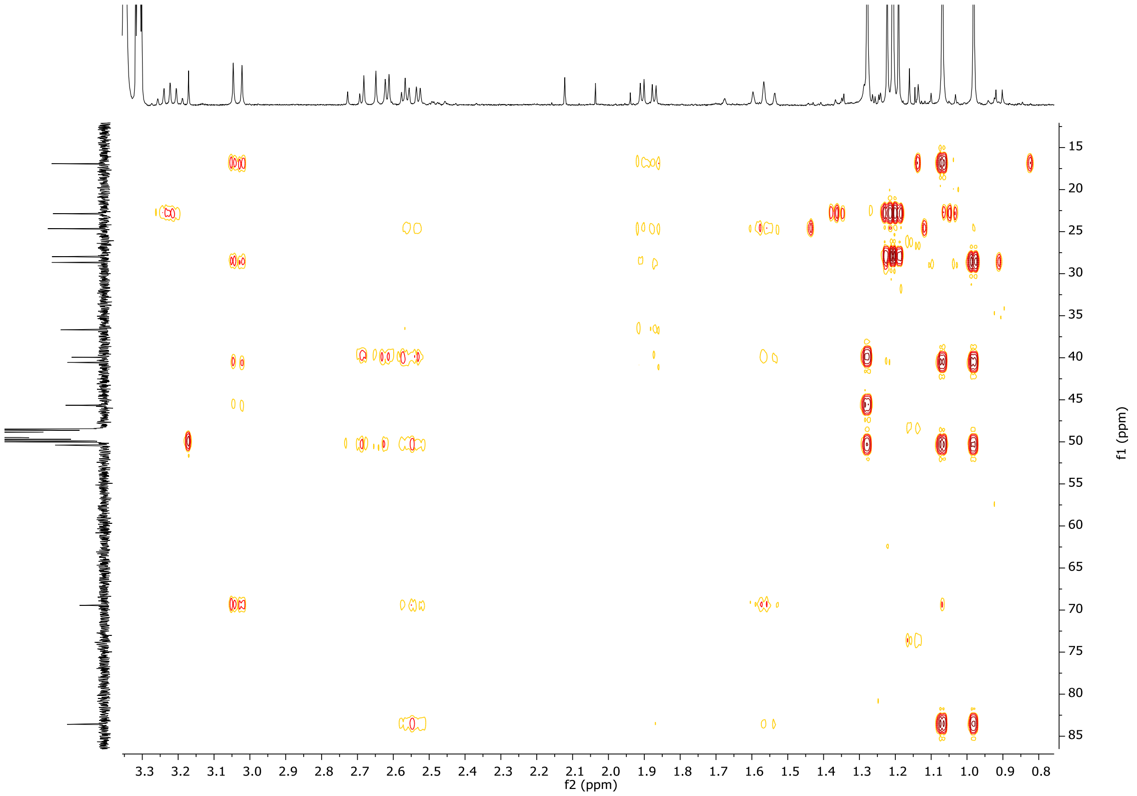

Figure S30. HMBC NMR (400 X 100 MHz, CD_3OD) spectrum of **2** (expansion).

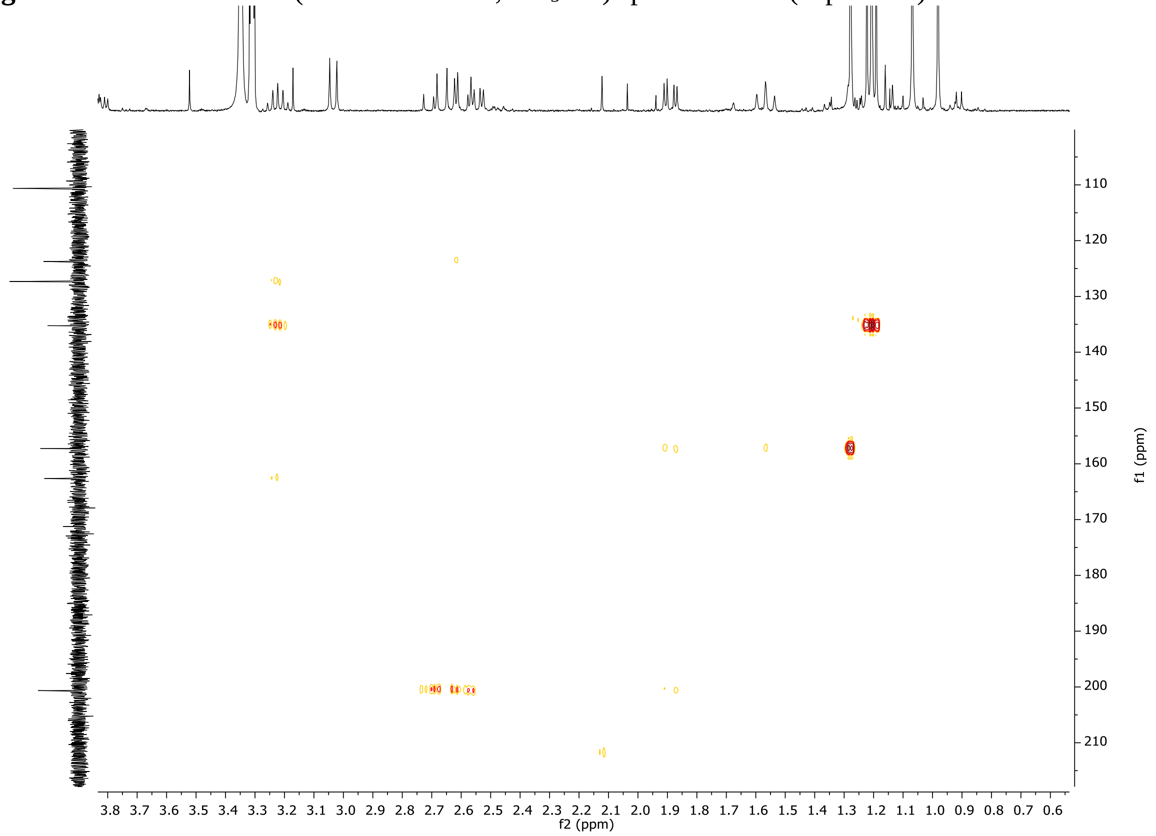

Figure S31. HMBC NMR (400 X 100 MHz, CD_3OD) spectrum of **2** (expansion).

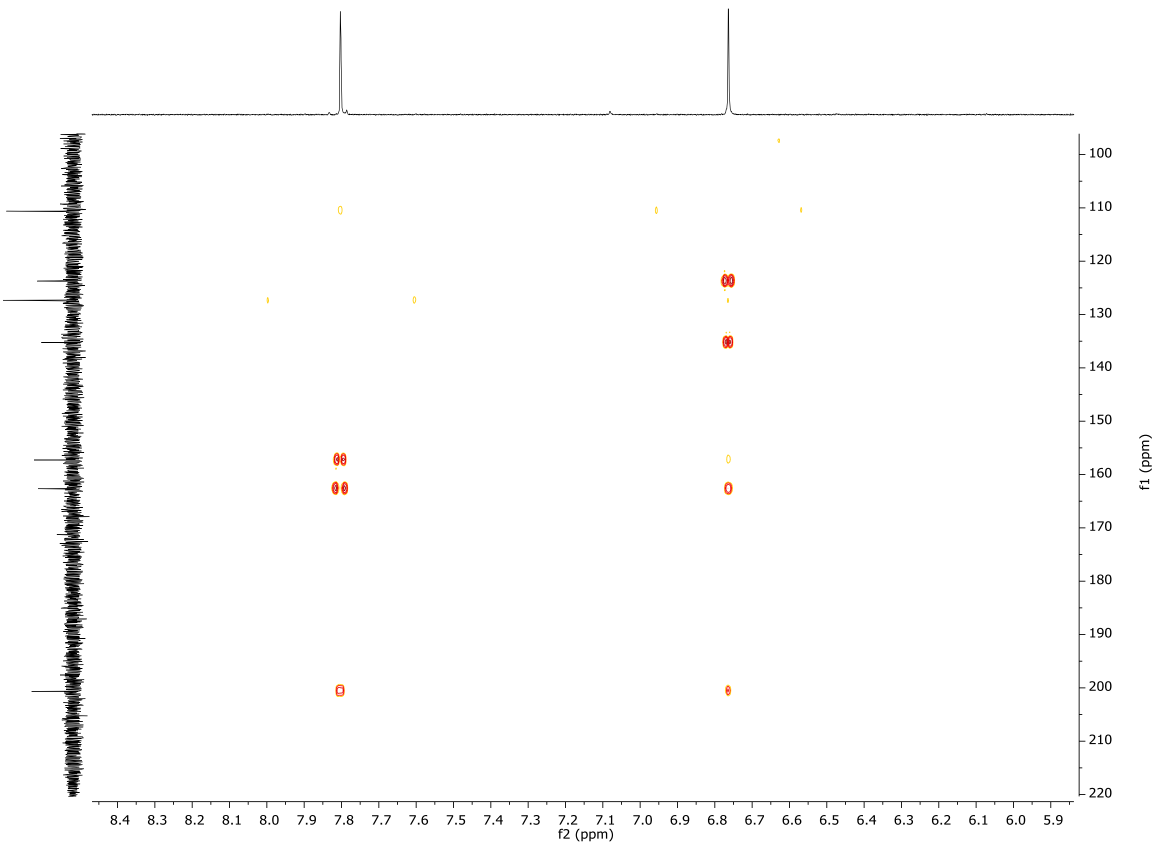

Figure S32. HMBC NMR (400 X 100 MHz, CD_3OD) spectrum of **2** (expansion).

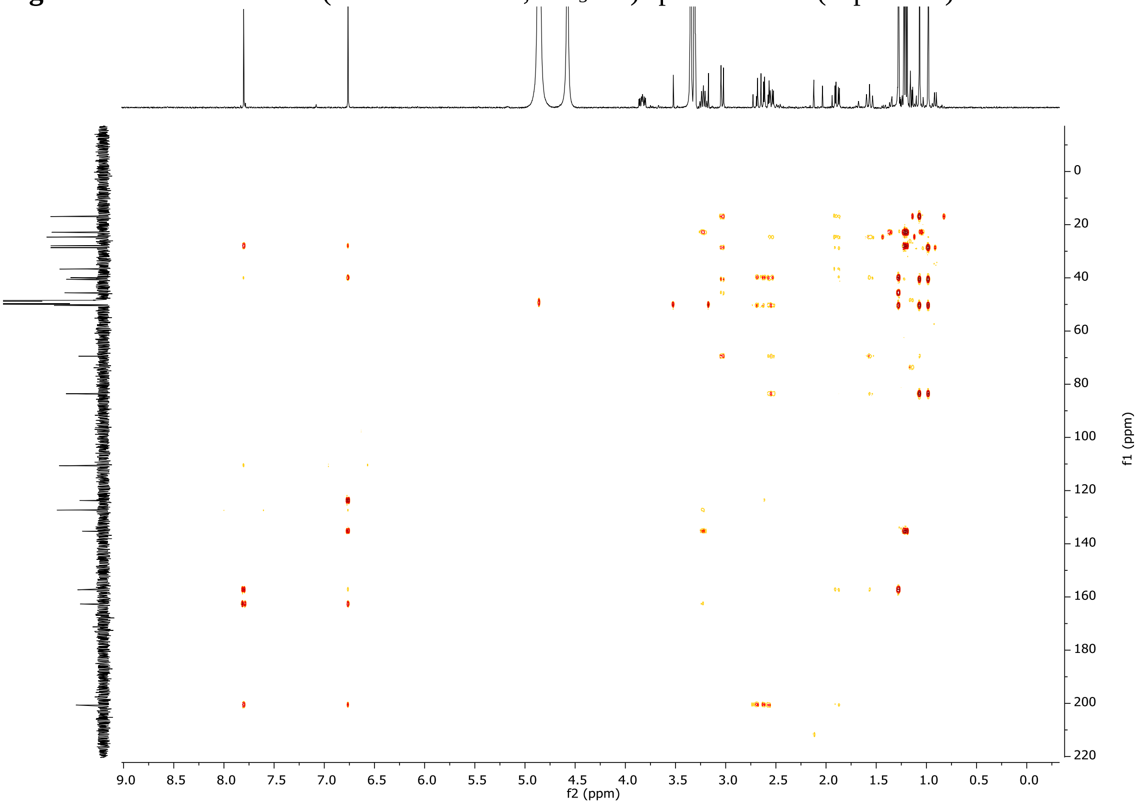

Figure S33. COSY NMR (400 MHz, CD_3OD) spectrum of **2**

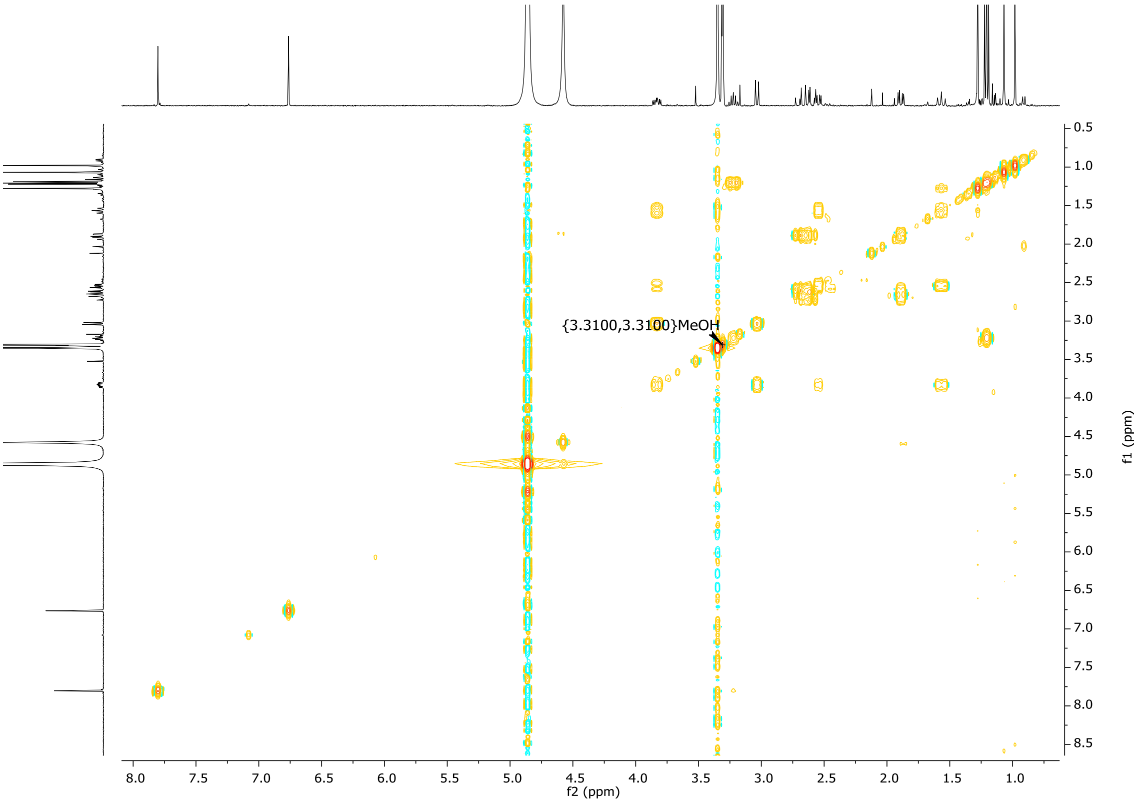

Figure S34. COSY NMR (400 MHz, CD_3OD) spectrum of **2**(expansion).

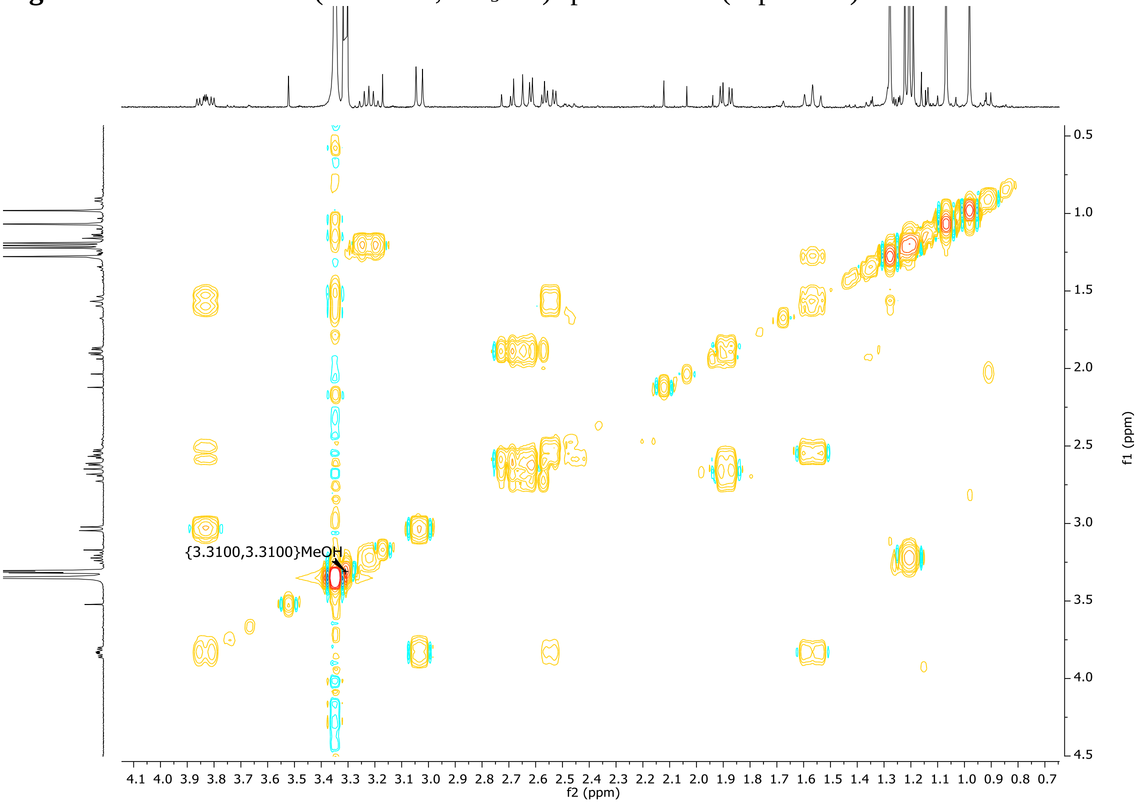

Figure S35. NOESY NMR (400 MHz, CD_3OD) spectrum of **2**

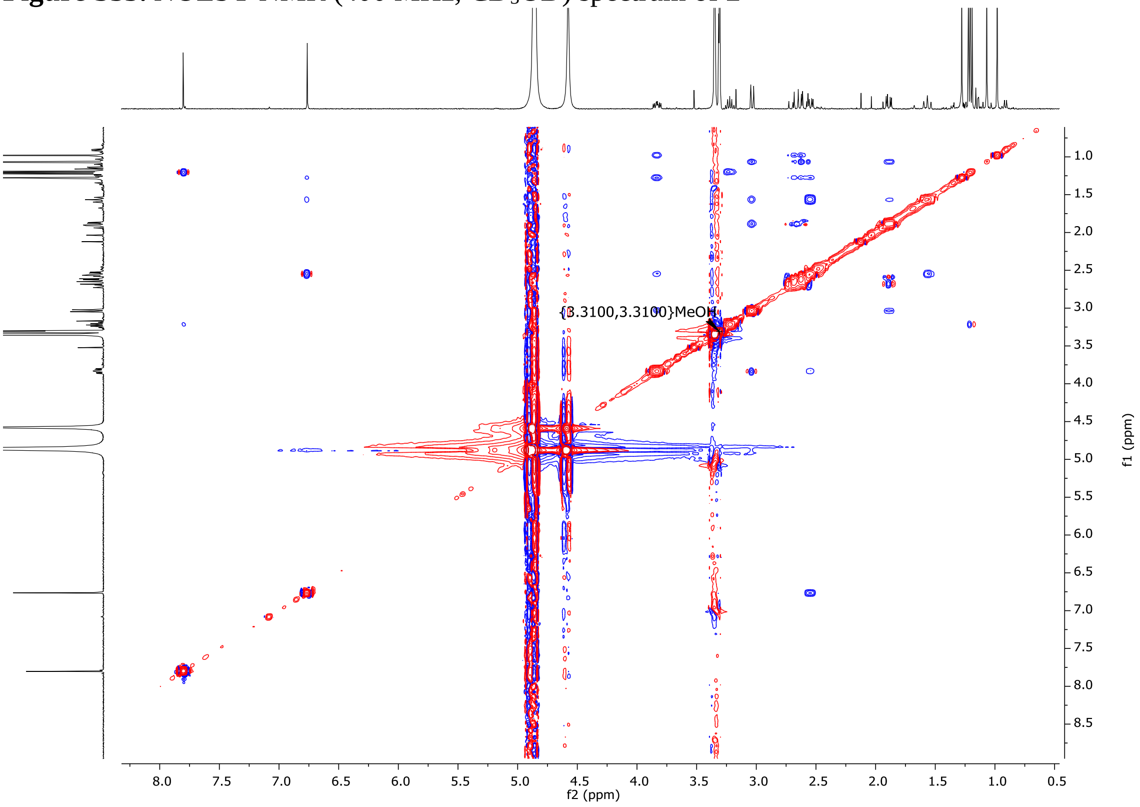

Figure S36. NOESY NMR (400 MHz, CD_3OD) spectrum of **2** (expansion).

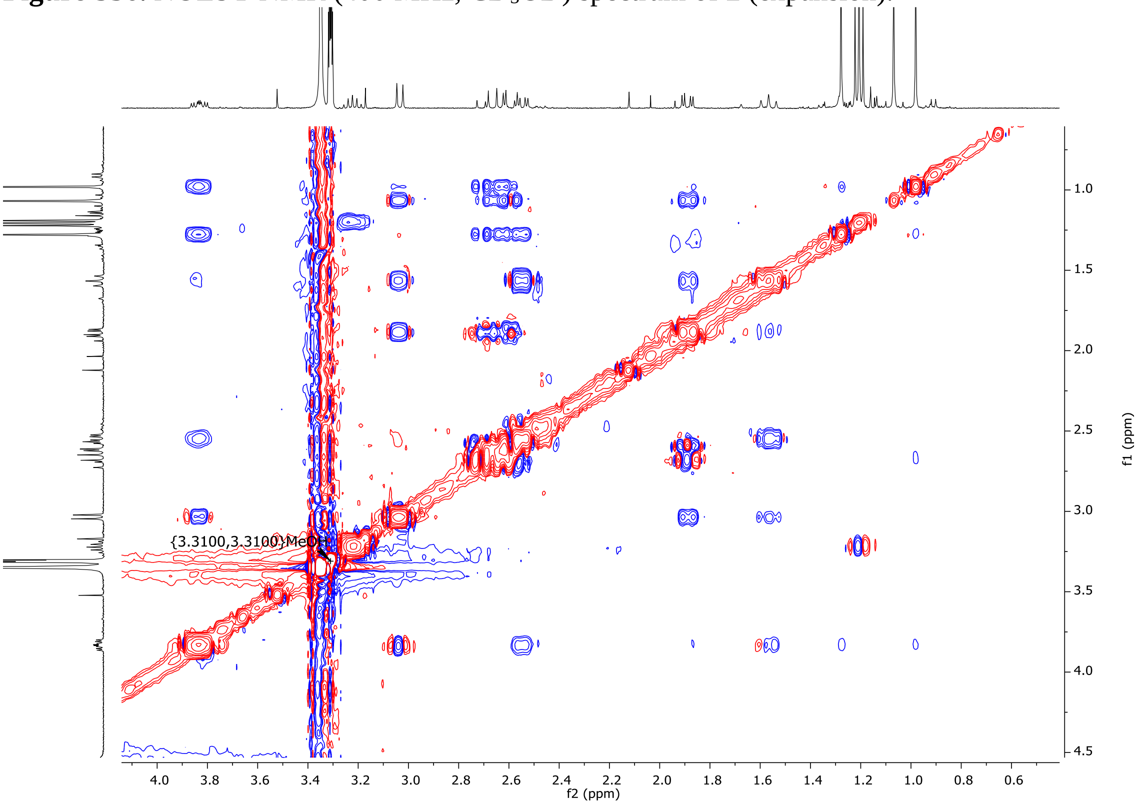

Figure S37. IR spectrum of compound **3**

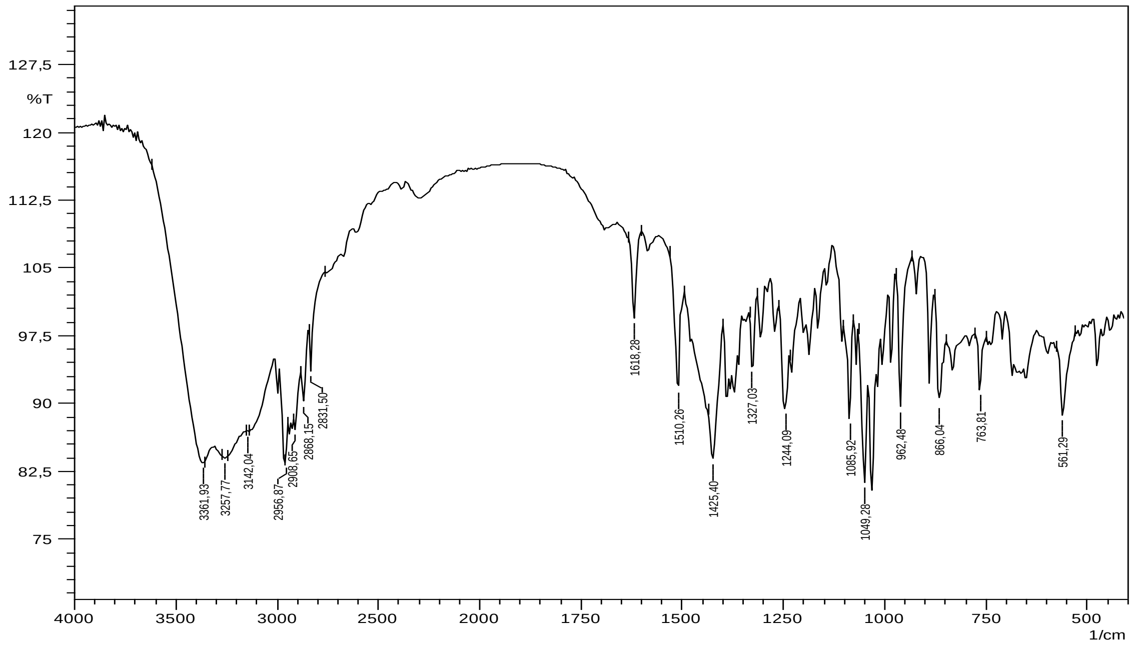

Figure S38. HRESIMS Spectrum of **3** ($[2M + Na]^+$ positive ion mode)

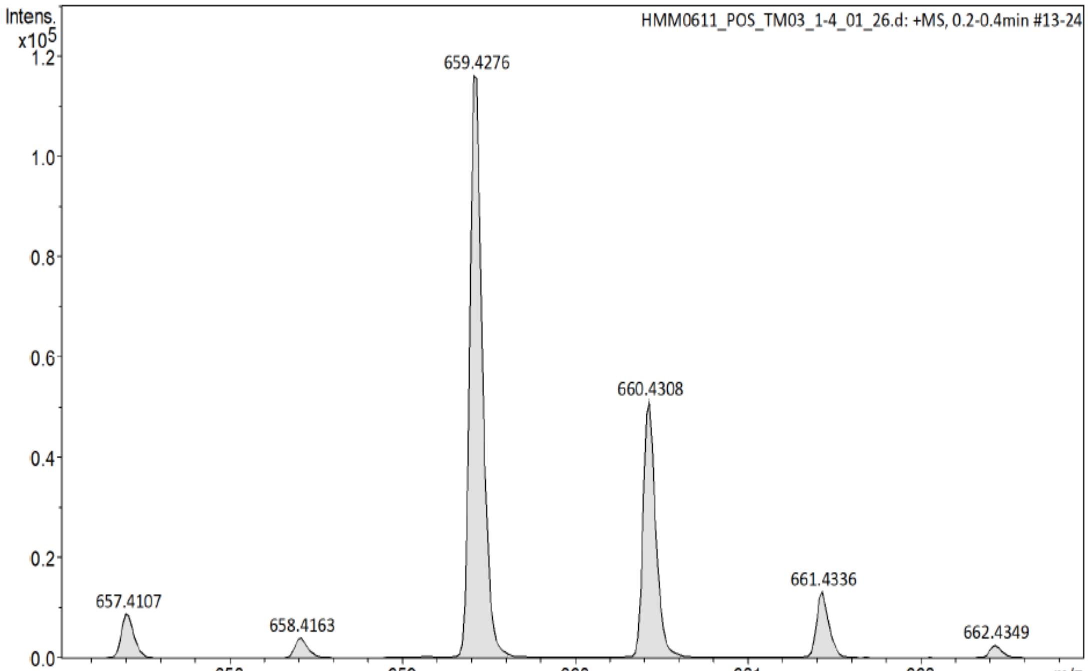

Figure S39. ^{1}H NMR (400 MHz, CD_3OD) spectrum of **3**

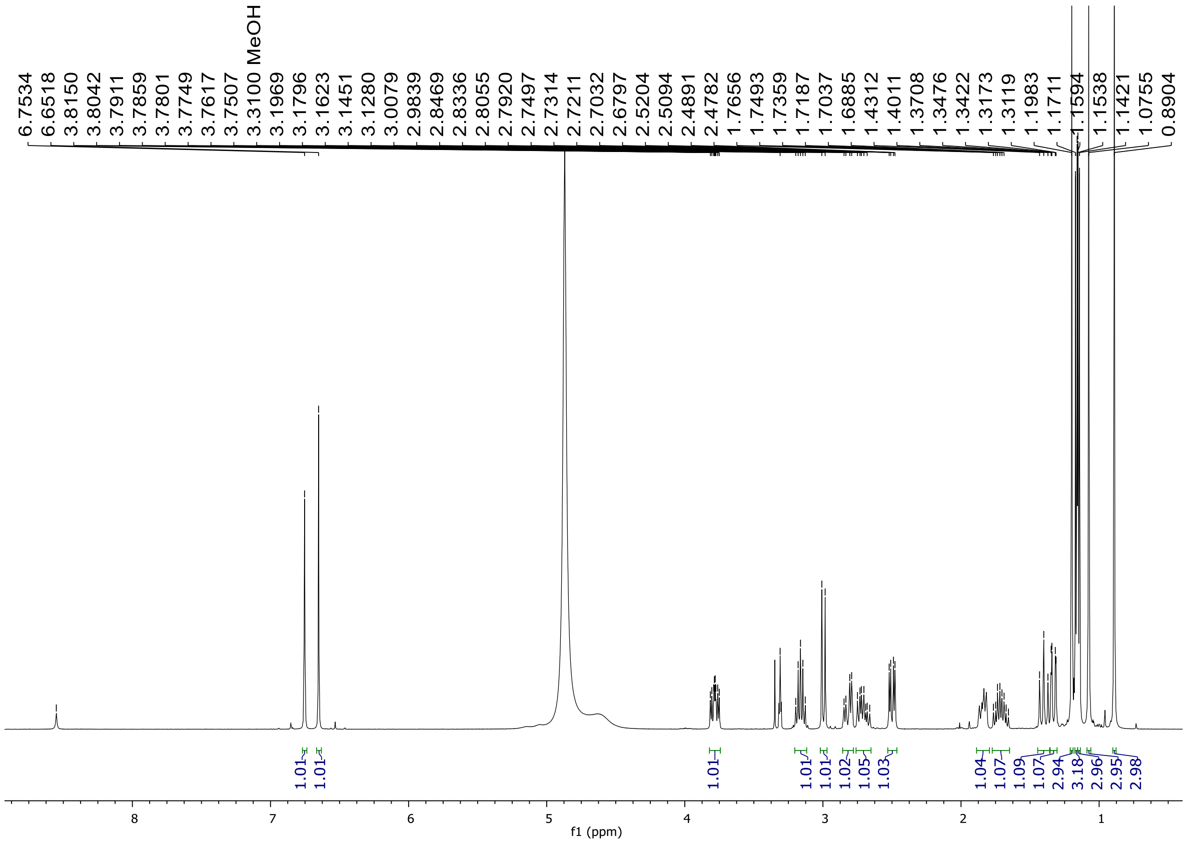

Figure S40. ^{1}H NMR (400 MHz, CD_3OD) spectrum of **3** (expansion).

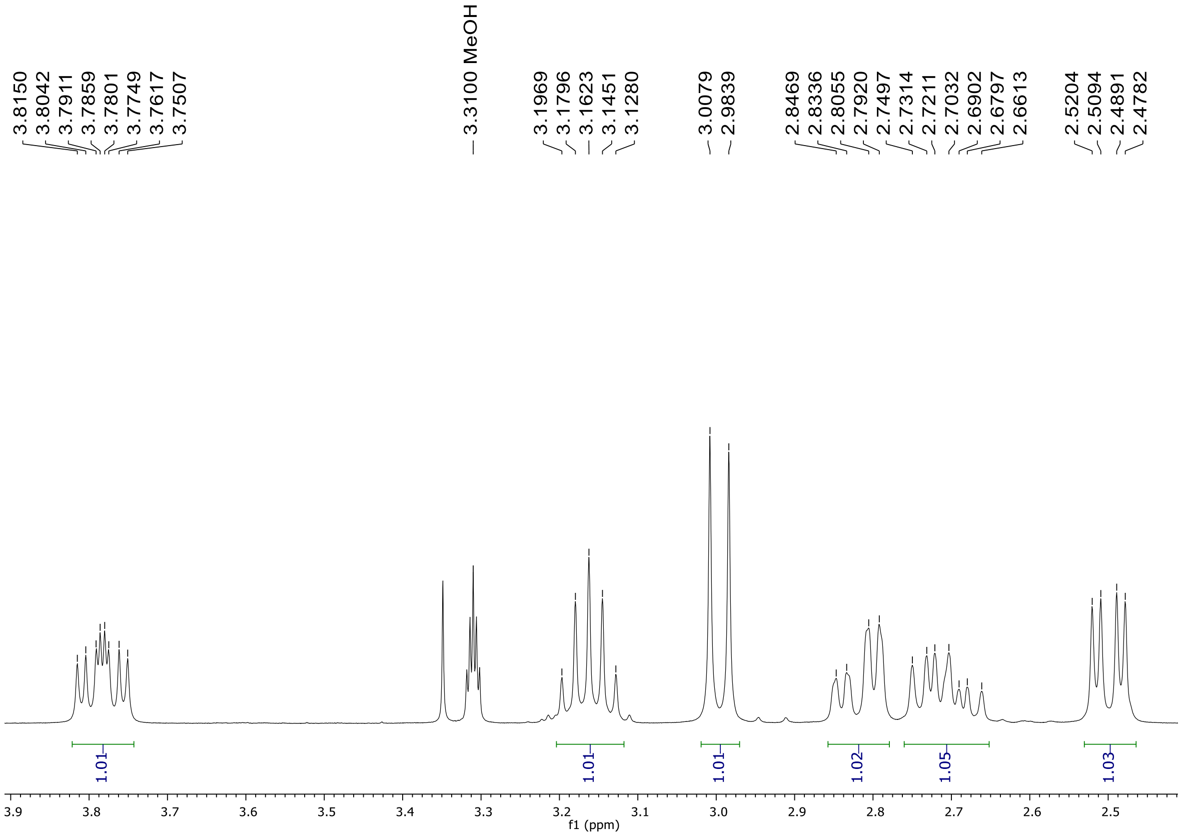

Figure S41. ^{1}H NMR (400 MHz, CD_3OD) spectrum of **3** (expansion).

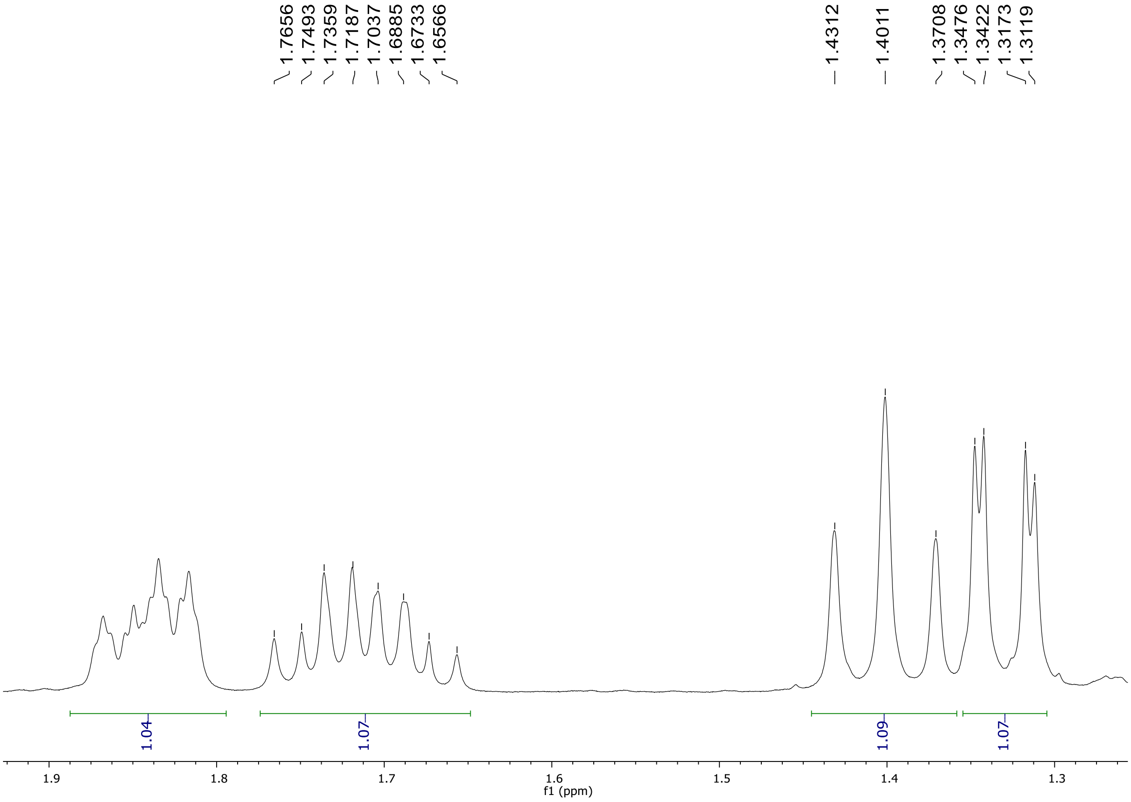

Figure S42. ^{1}H NMR (400 MHz, CD_3OD) spectrum of **3** (expansion).

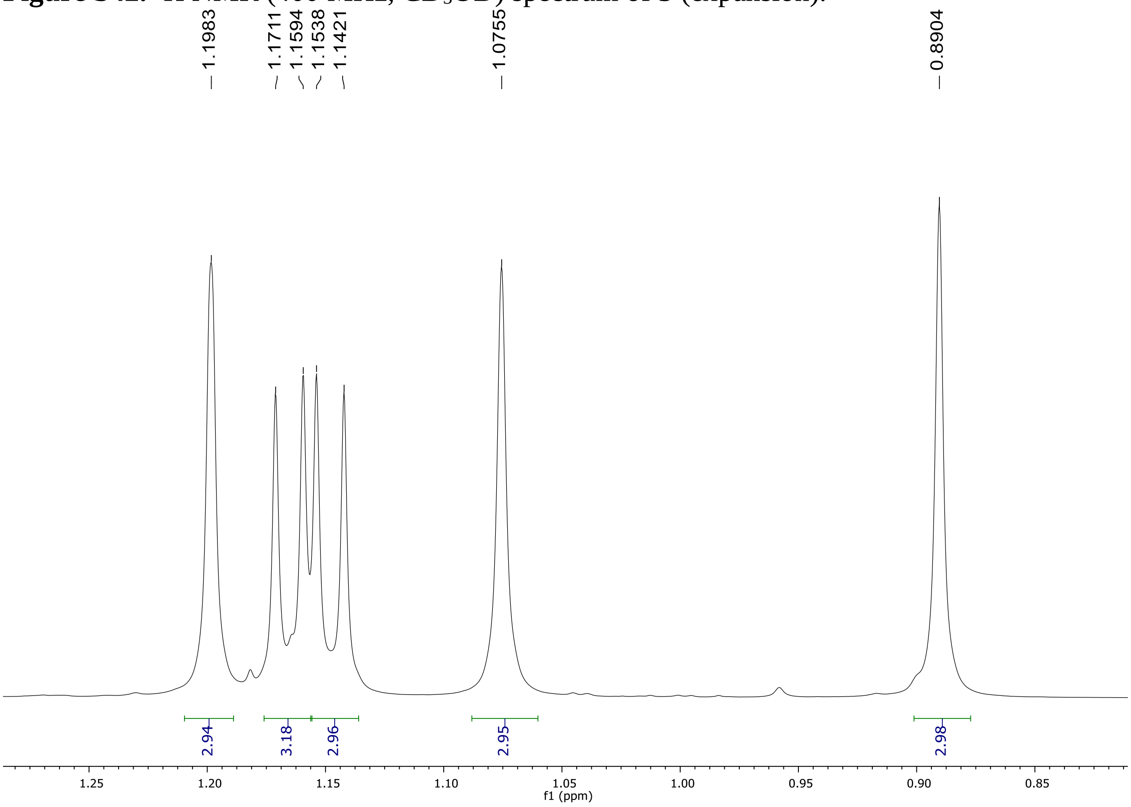

Figure S43. ^{13}C NMR - BB (100 MHz, CD_3OD) spectrum of **3**

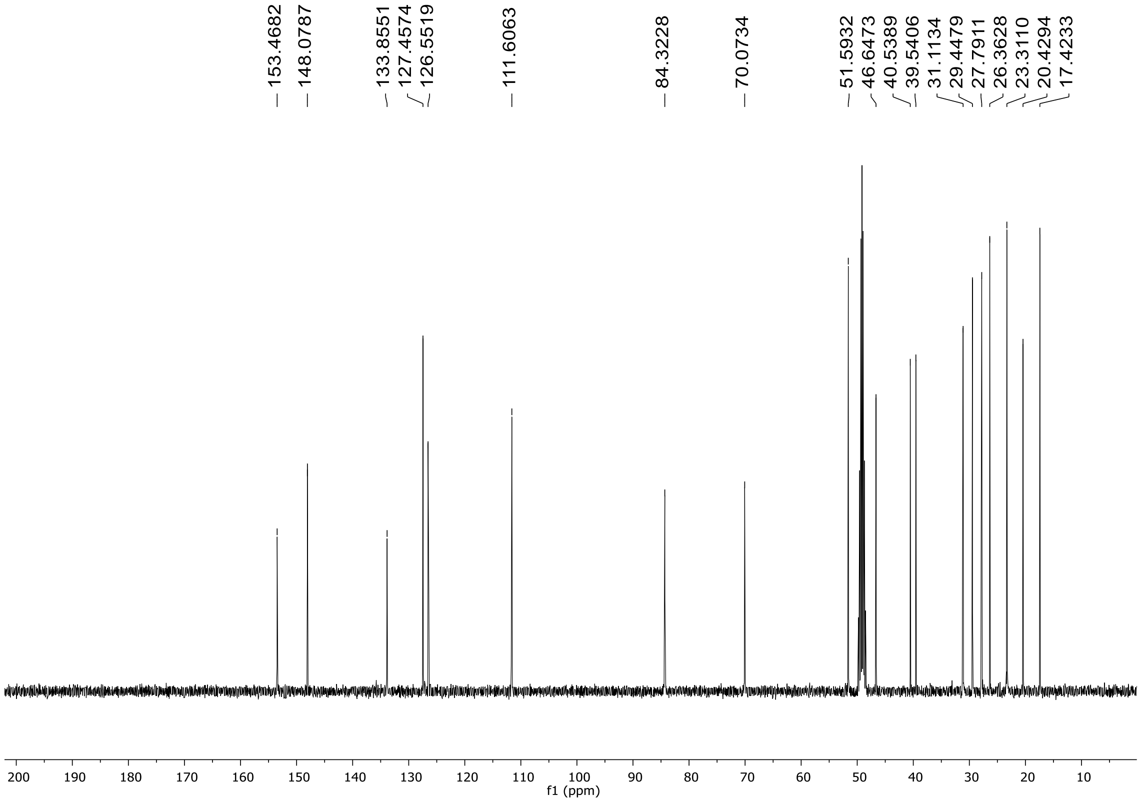

Figure S44. ^{13}C NMR - BB (100 MHz, CD_3OD) spectrum of **3** (expansion).

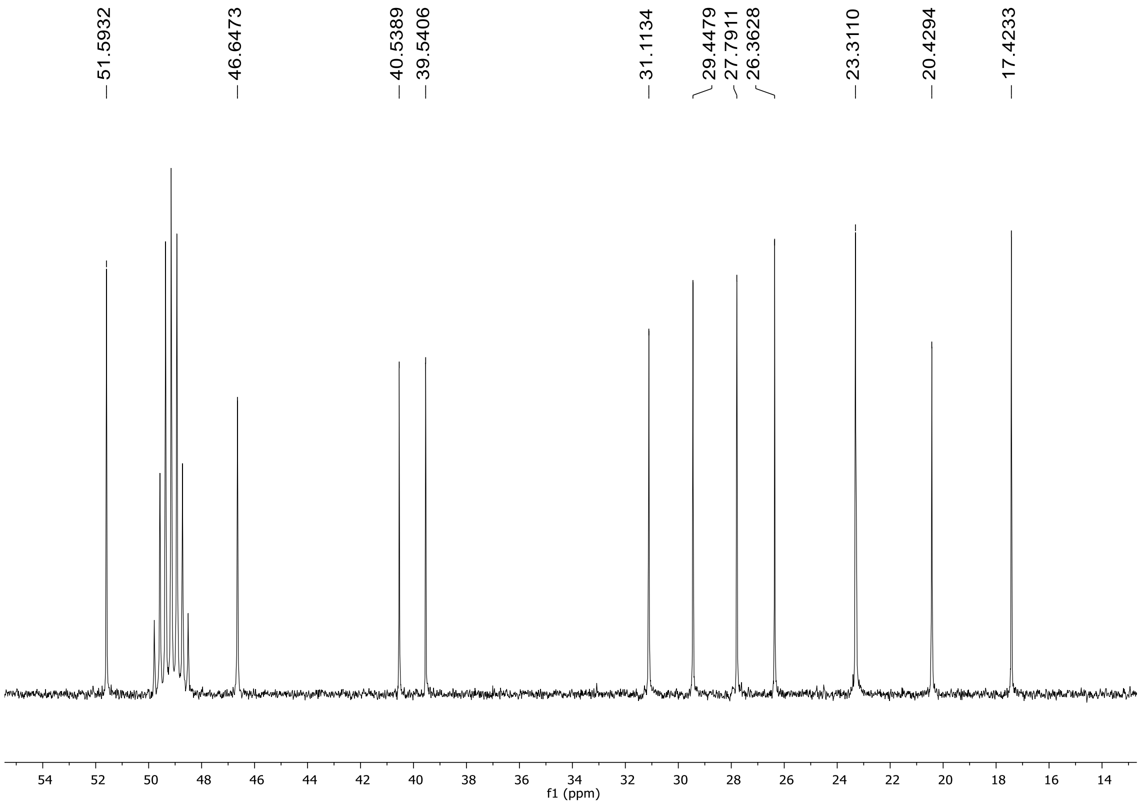

Figure S45. ^{13}C NMR – DEPT 135 (100 MHz, CD_3OD) spectrum of **3.**

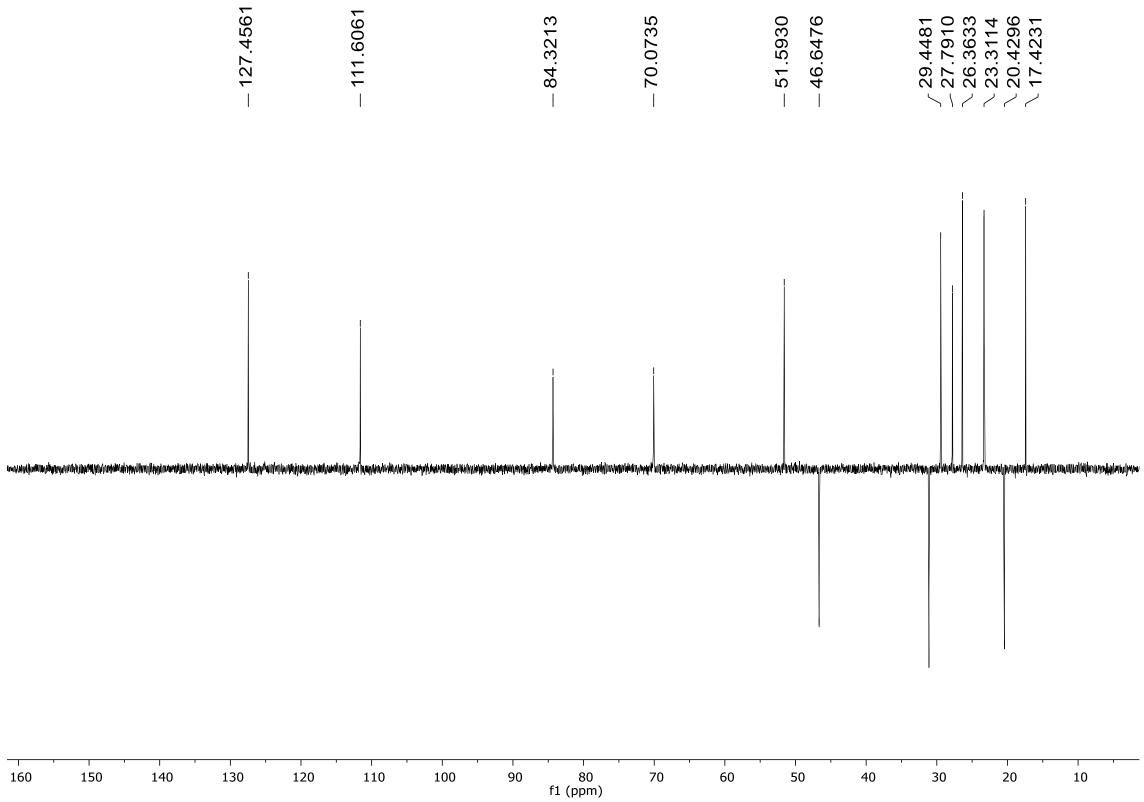

Figure S46. HSQC NMR (400 X 100 MHz, CD_3OD) spectrum of **3**

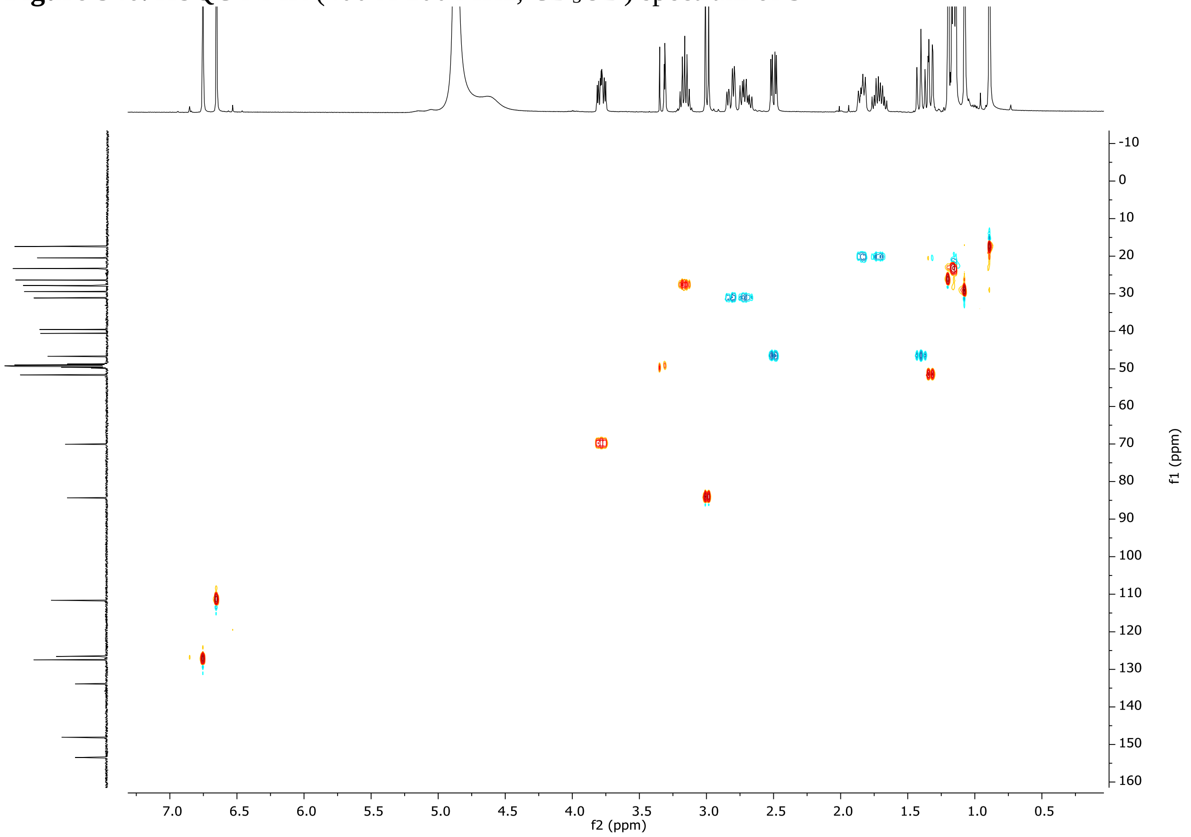

Figure S47. HMBC NMR (400 X 100 MHz, CD_3OD) spectrum of **3.**

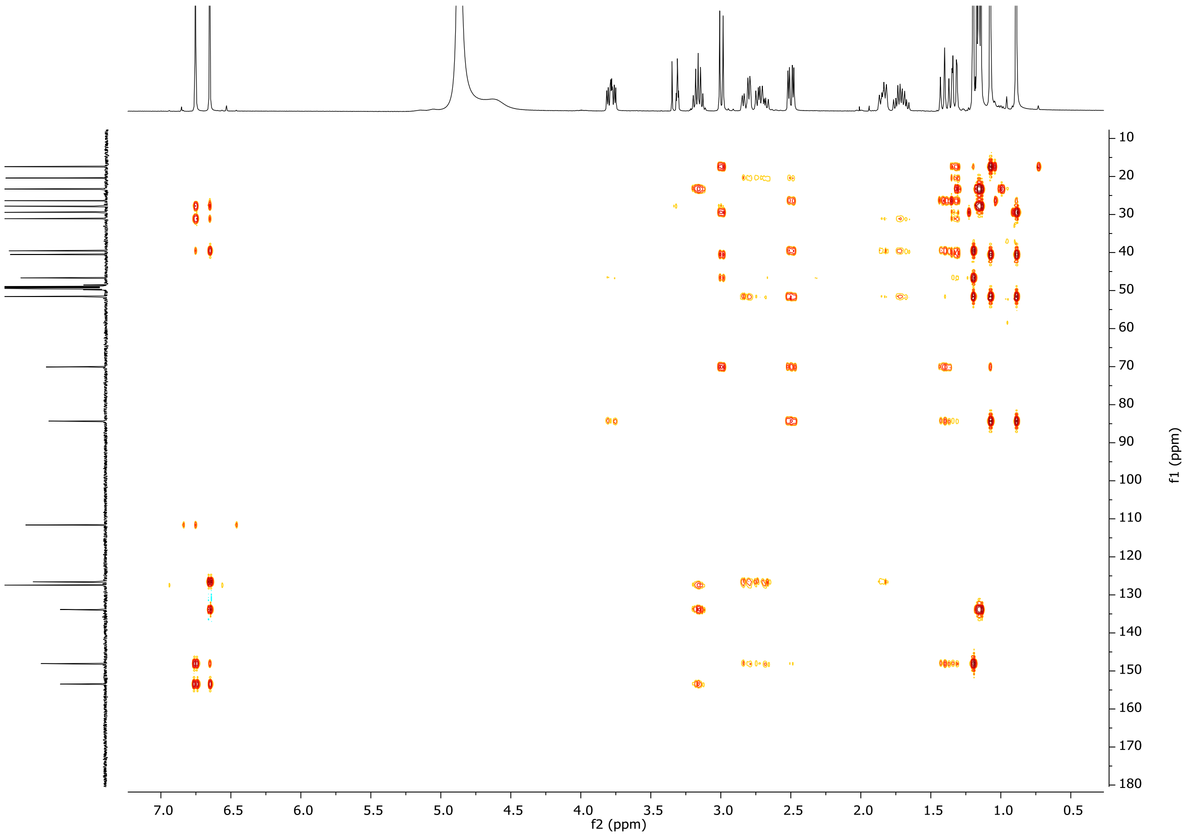

Figure S48. HMBC NMR (400 X 100 MHz, CD_3OD) spectrum of **3** (expansion).

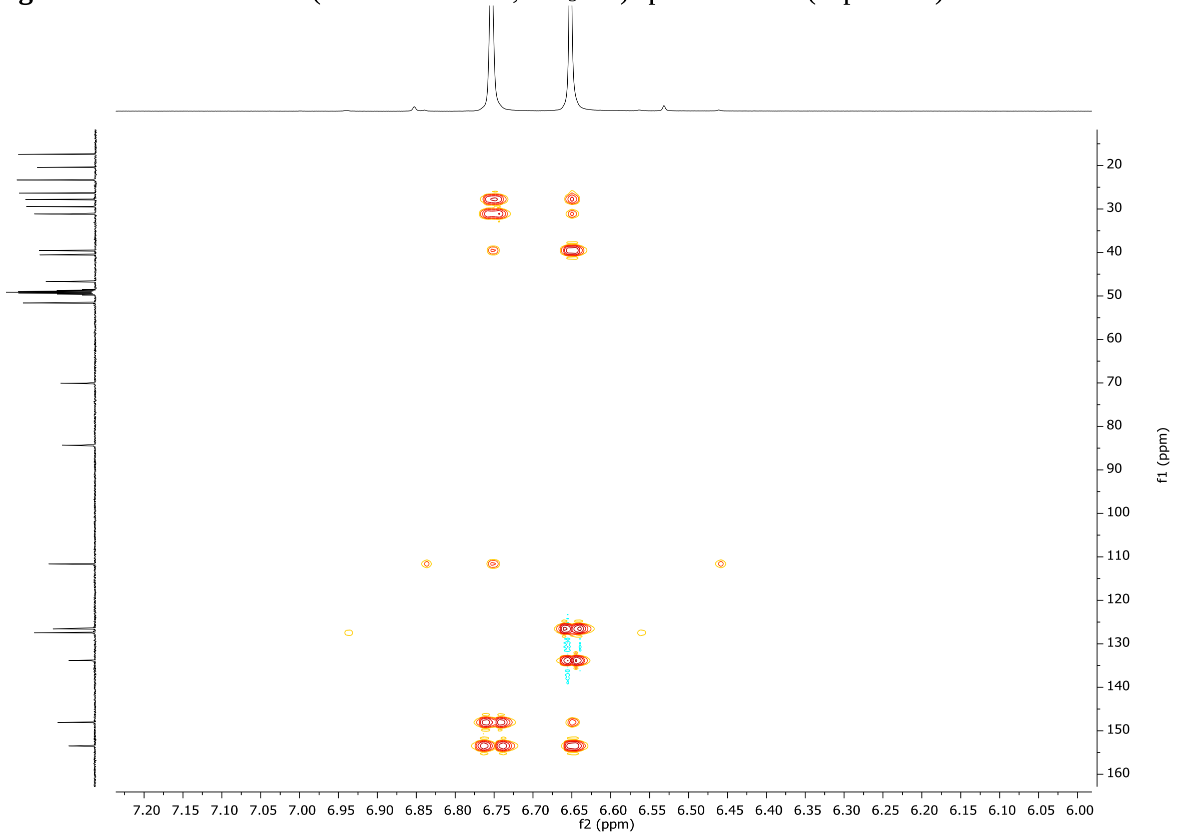

Figure S49. HMBC NMR (400 X 100 MHz, CD_3OD) spectrum of **3** (expansion).

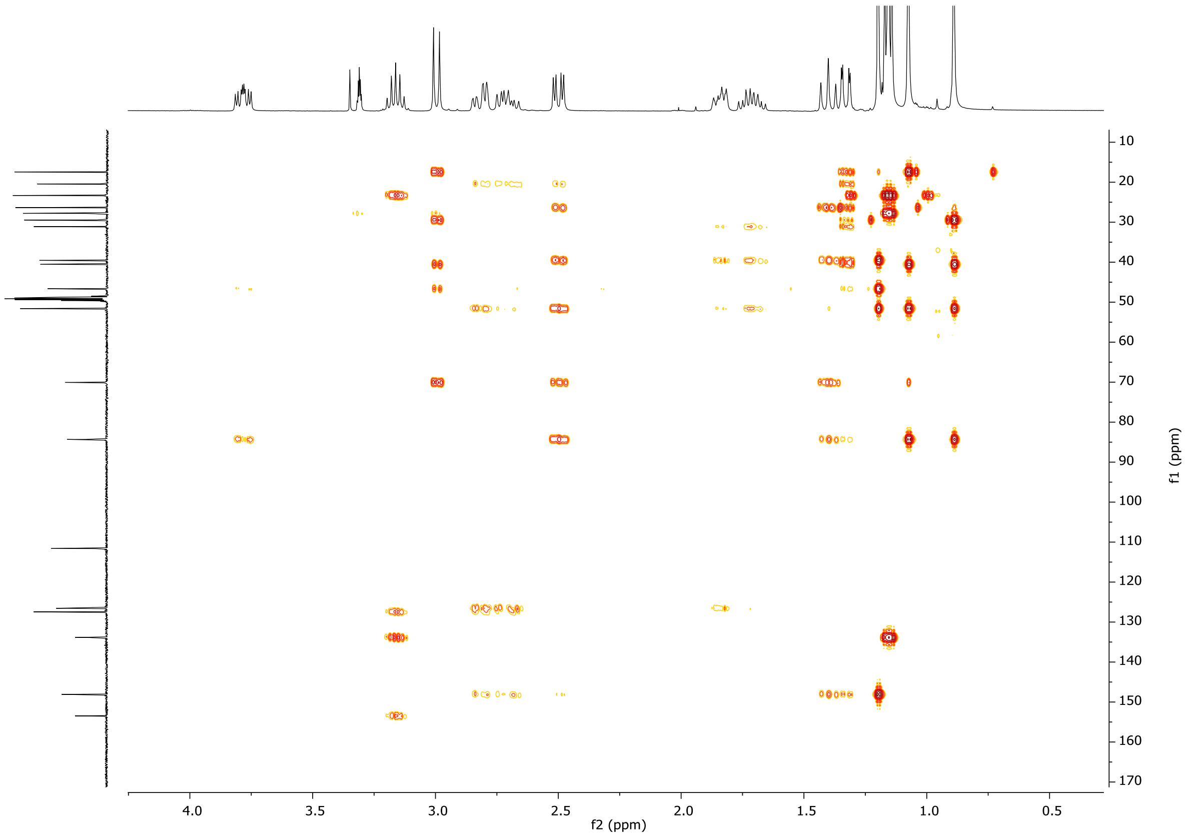

Figure S50. COSY NMR (400 MHz, CD_3OD) spectrum of **3**.

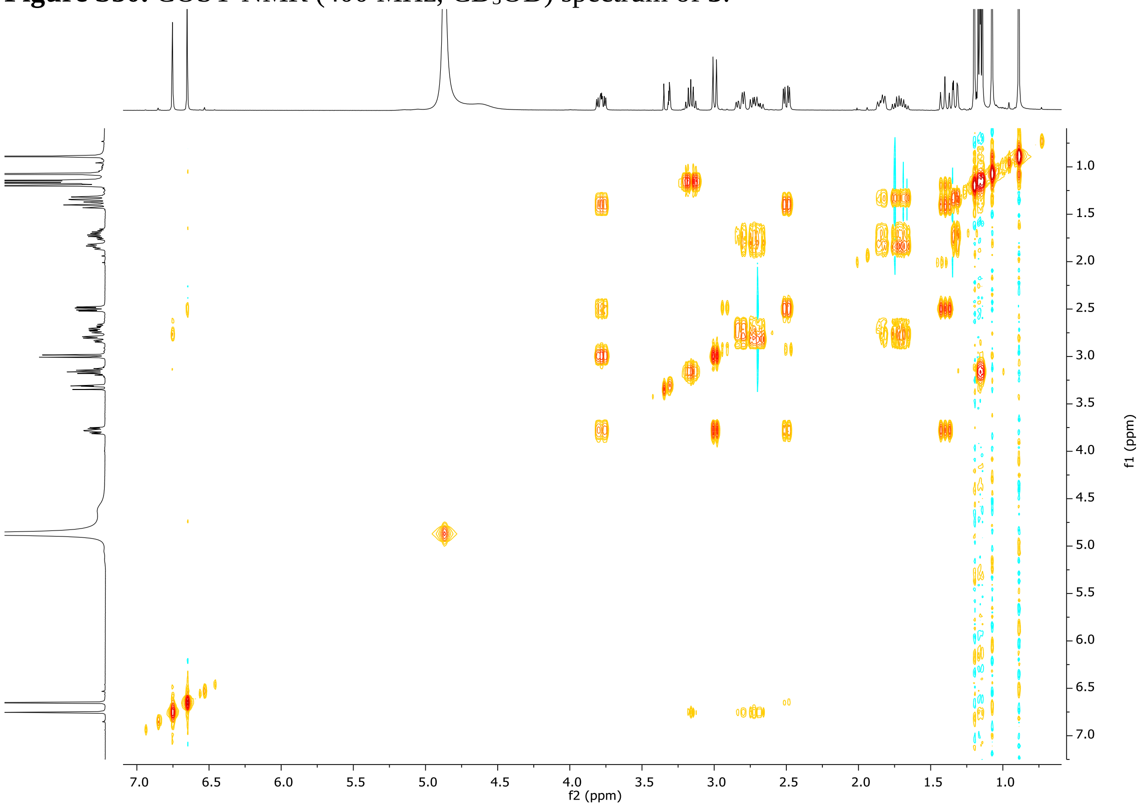

Figure S51. COSY NMR (400 MHz, CD_3OD) spectrum of **3** (expansion).

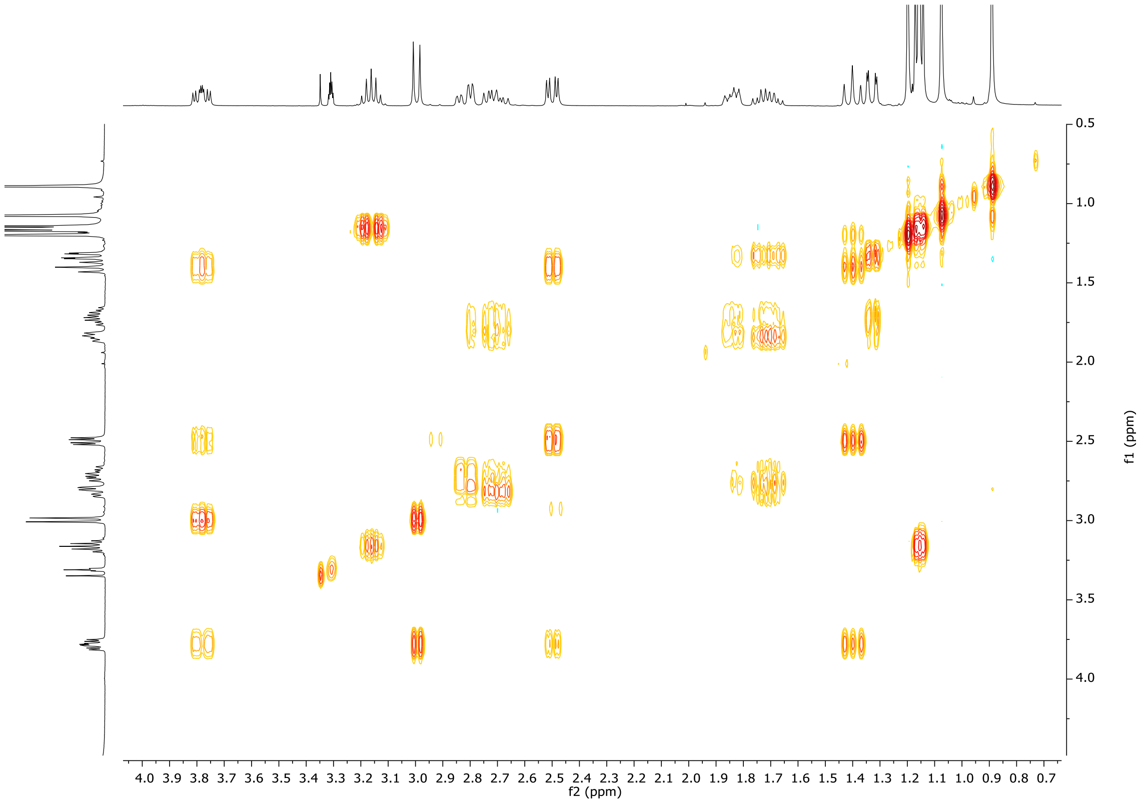

Figure S52. NOESY NMR (400 MHz, CD_3OD) spectrum of **3.**

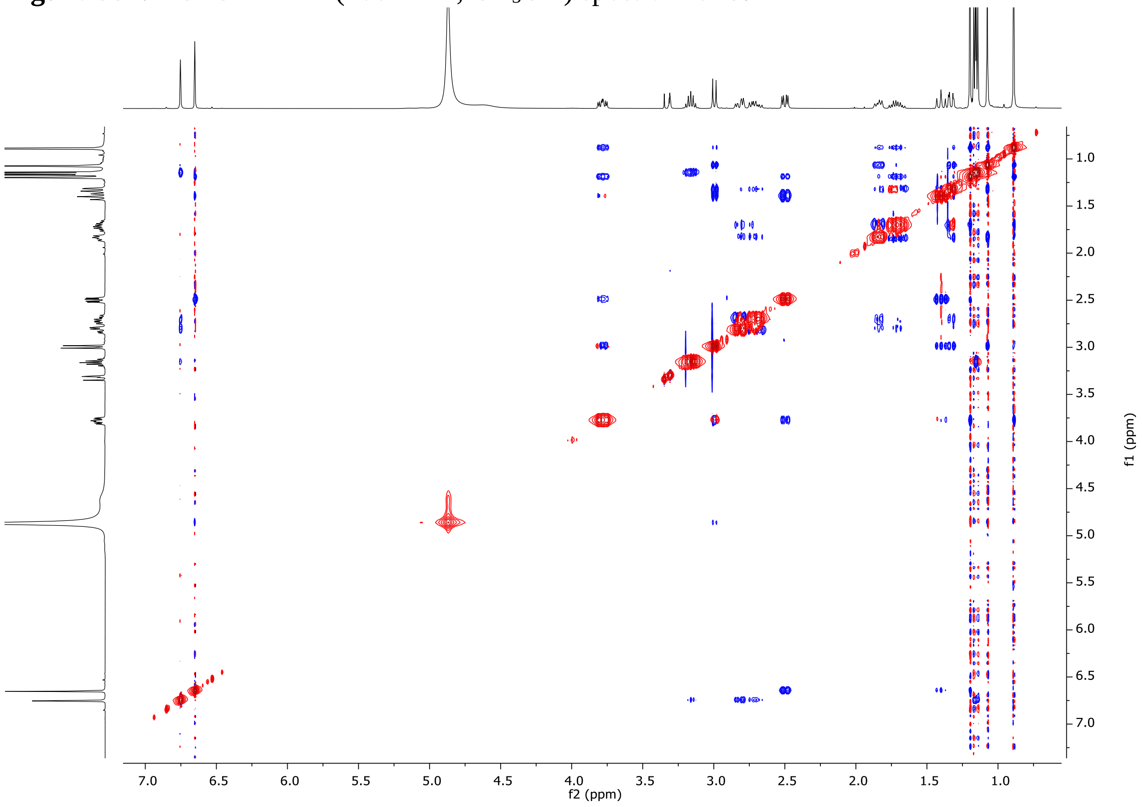

Figure S53. NOESY NMR (400 MHz, CD_3OD) spectrum of **3** (expansion).

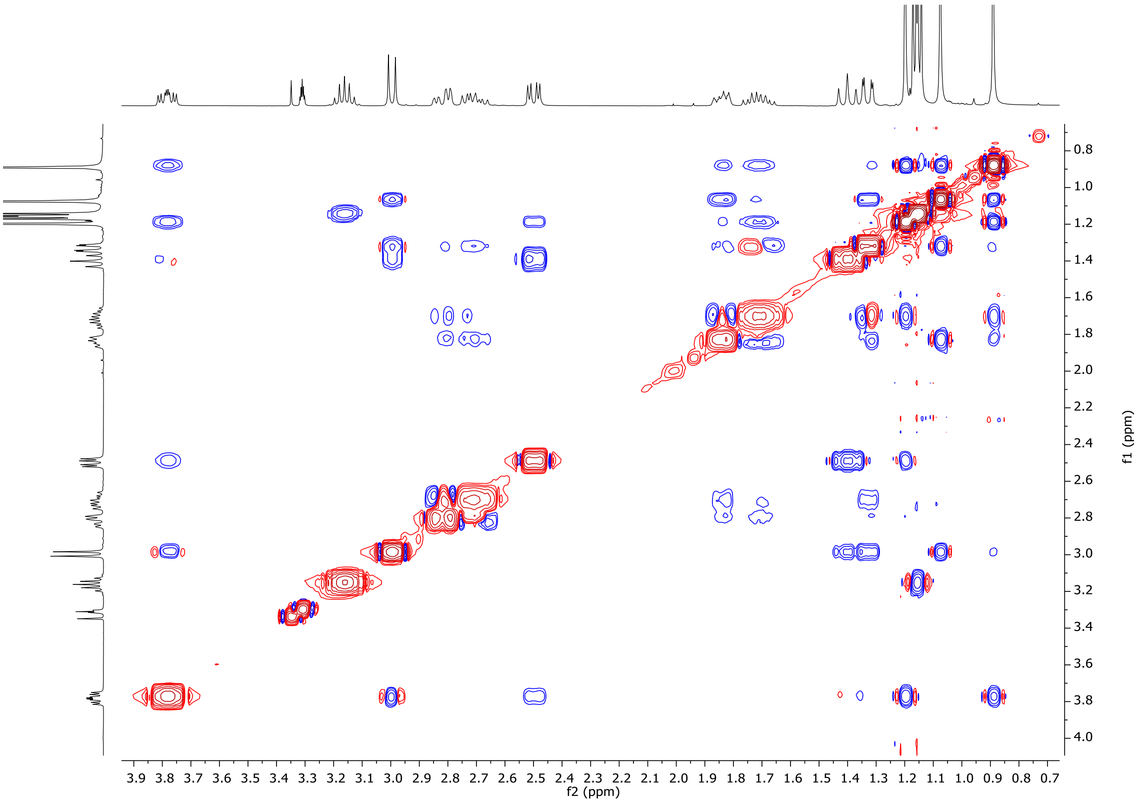

Figure S54. IR spectrum of compound **4.**

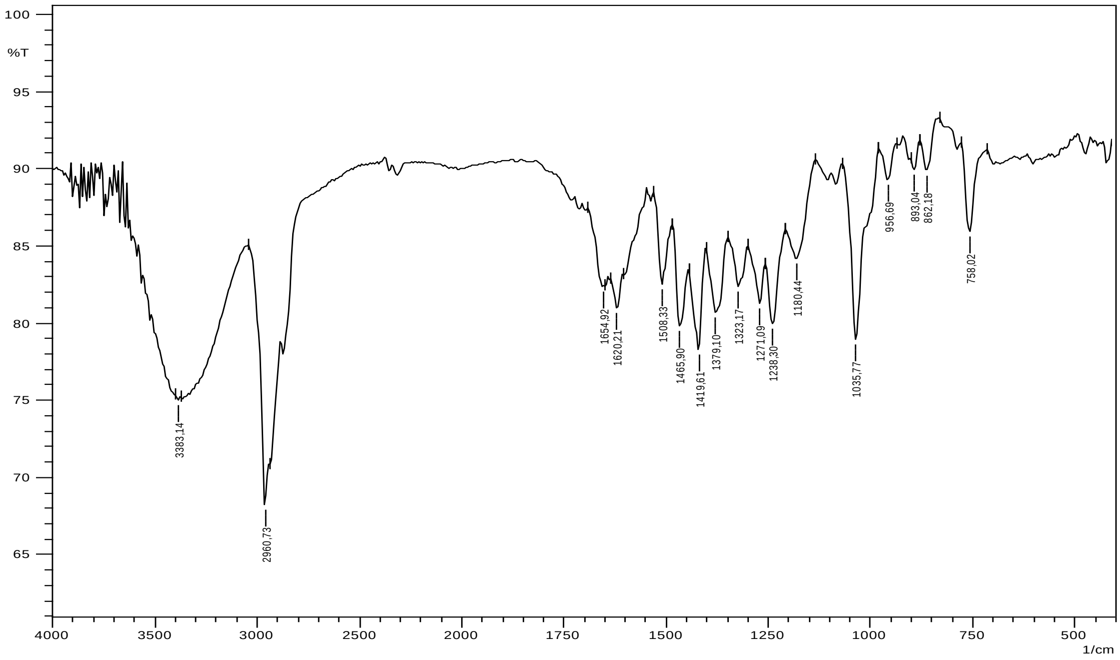

Figure S55. HRESIMS Spectrum of **4** ([M - H_2O + H]$^+$ positive ion mode)

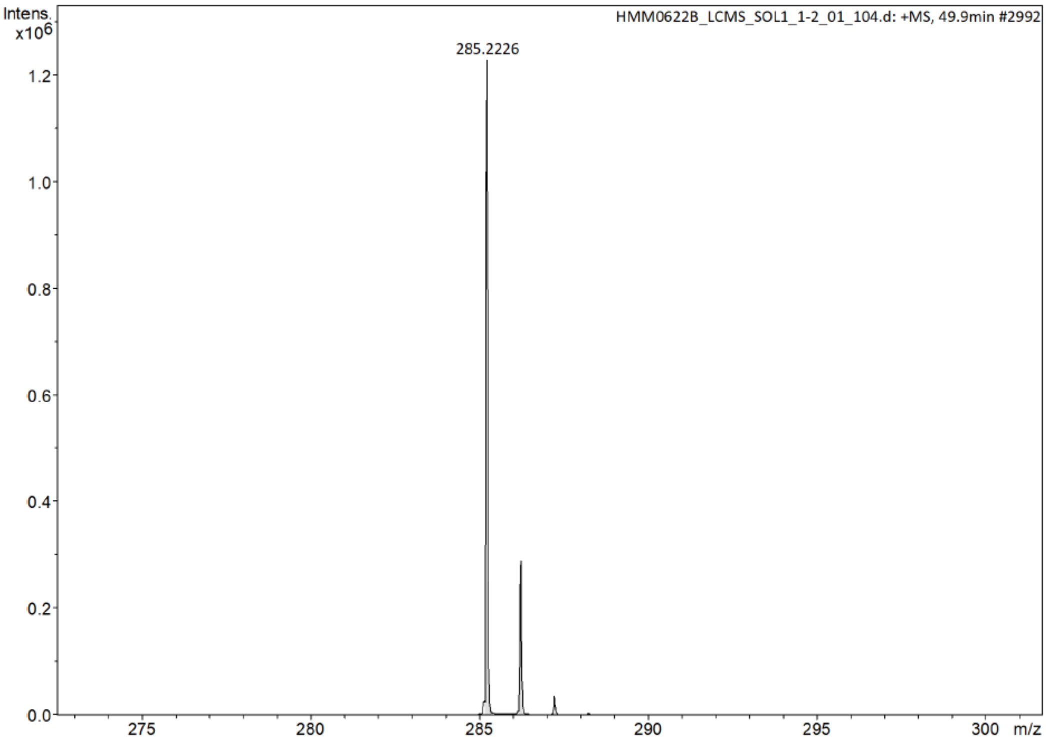

Figure S56. ^{1}H NMR (400 MHz, CD_3OD) spectrum of **4.**

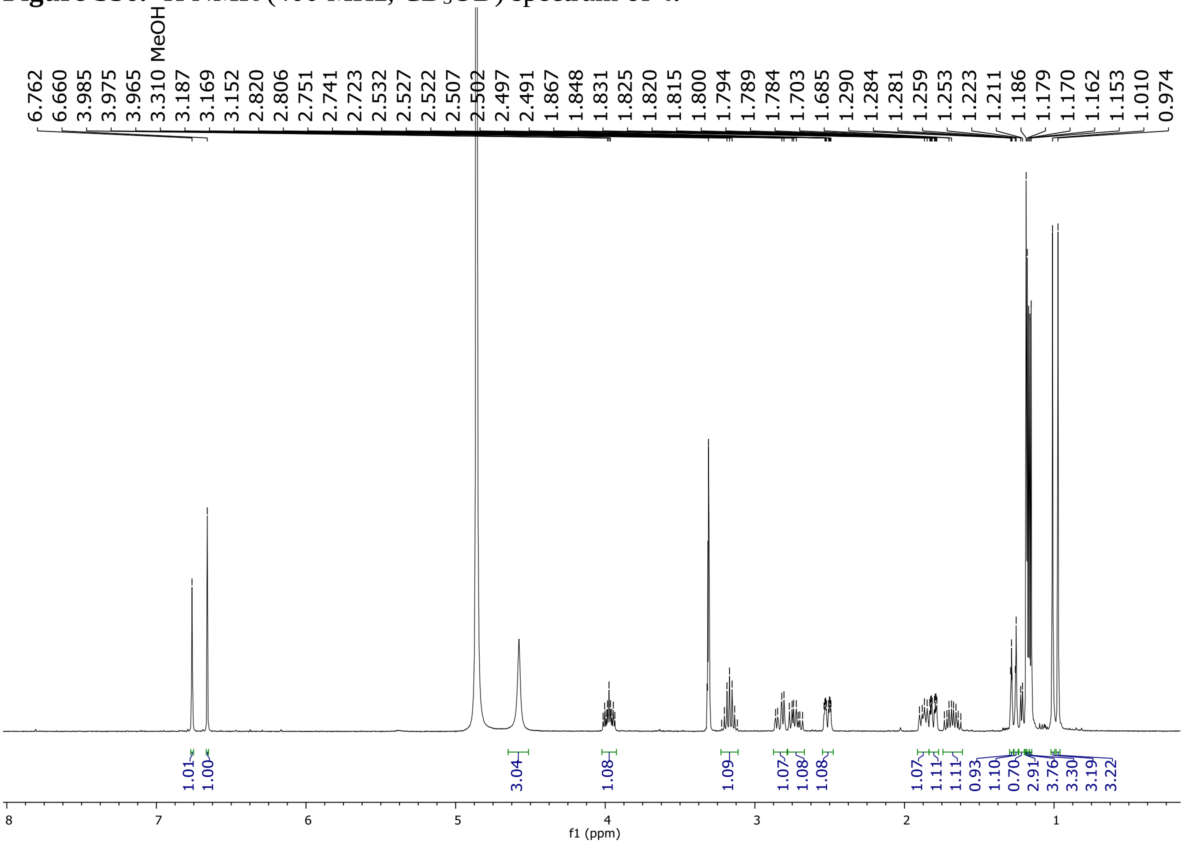

Figure S57. ^{1}H NMR (400 MHz, CD_3OD) spectrum of **4** (expansion).

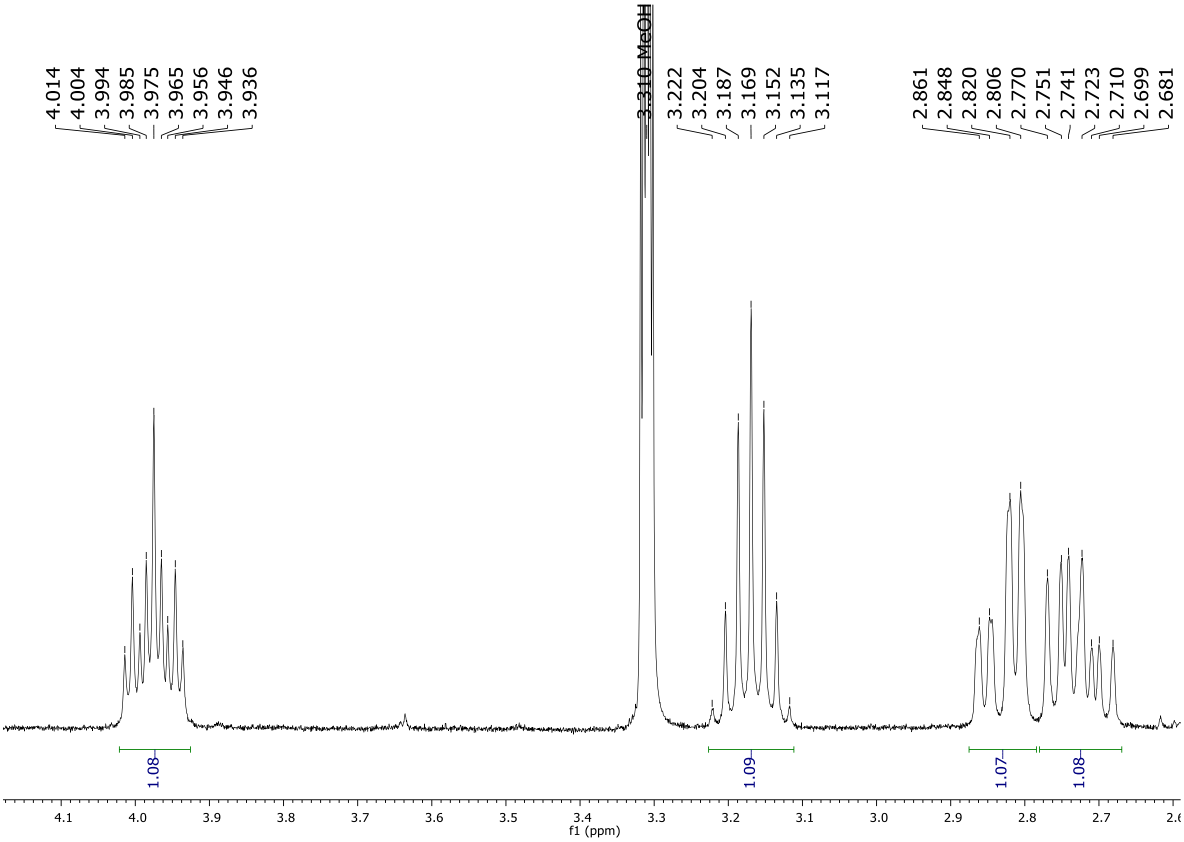

Figure S58. ^{1}H NMR (400 MHz, CD_3OD) spectrum of **4** (expansion).

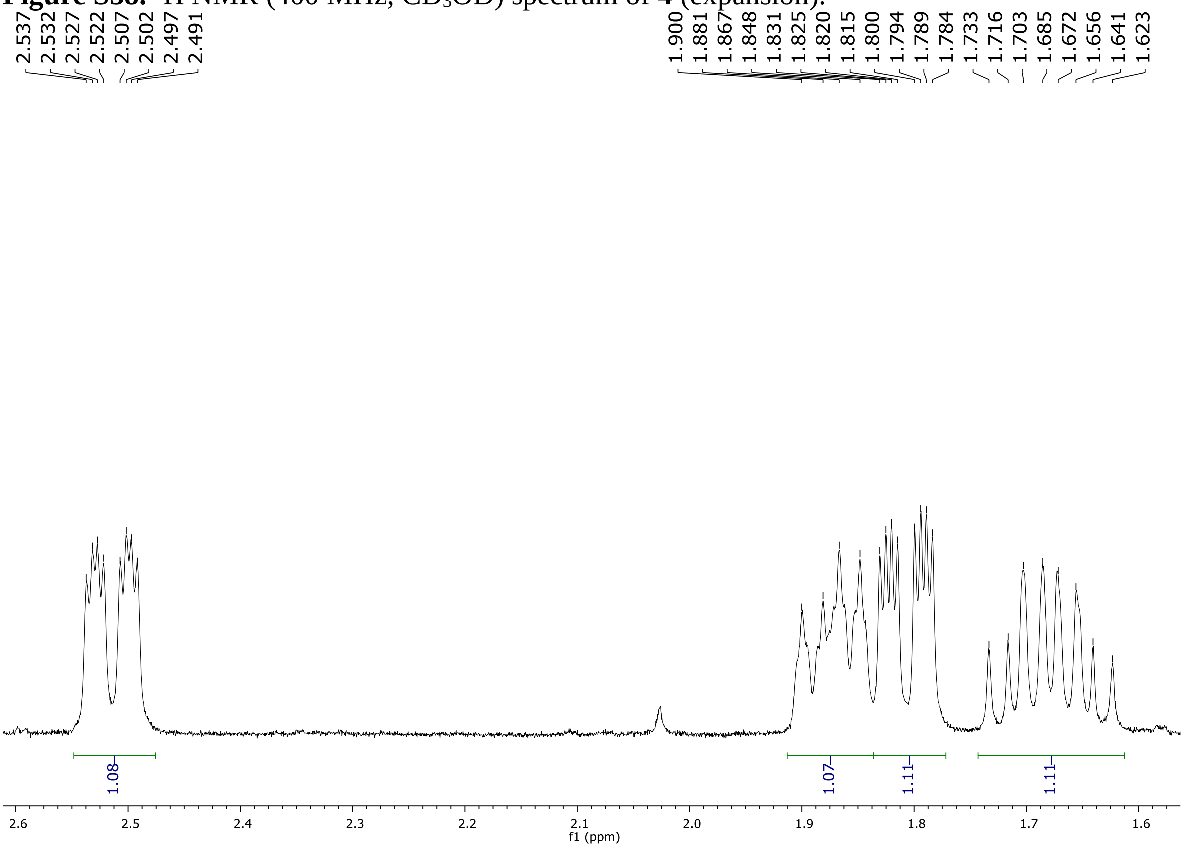

Figure S59. ^{1}H NMR (400 MHz, CD_3OD) spectrum of **4** (expansion).

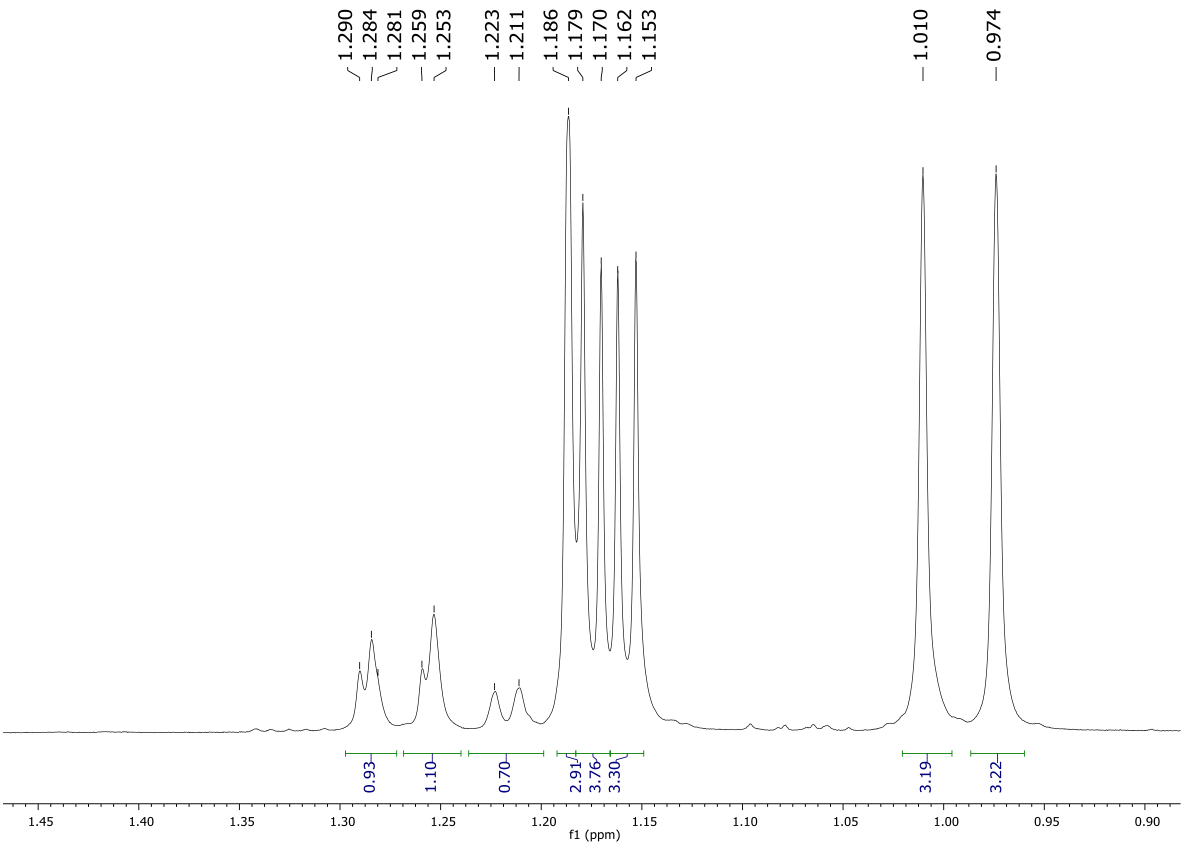

Figure S60. ^{13}C NMR - BB (100 MHz, CD_3OD) spectrum of **4.**

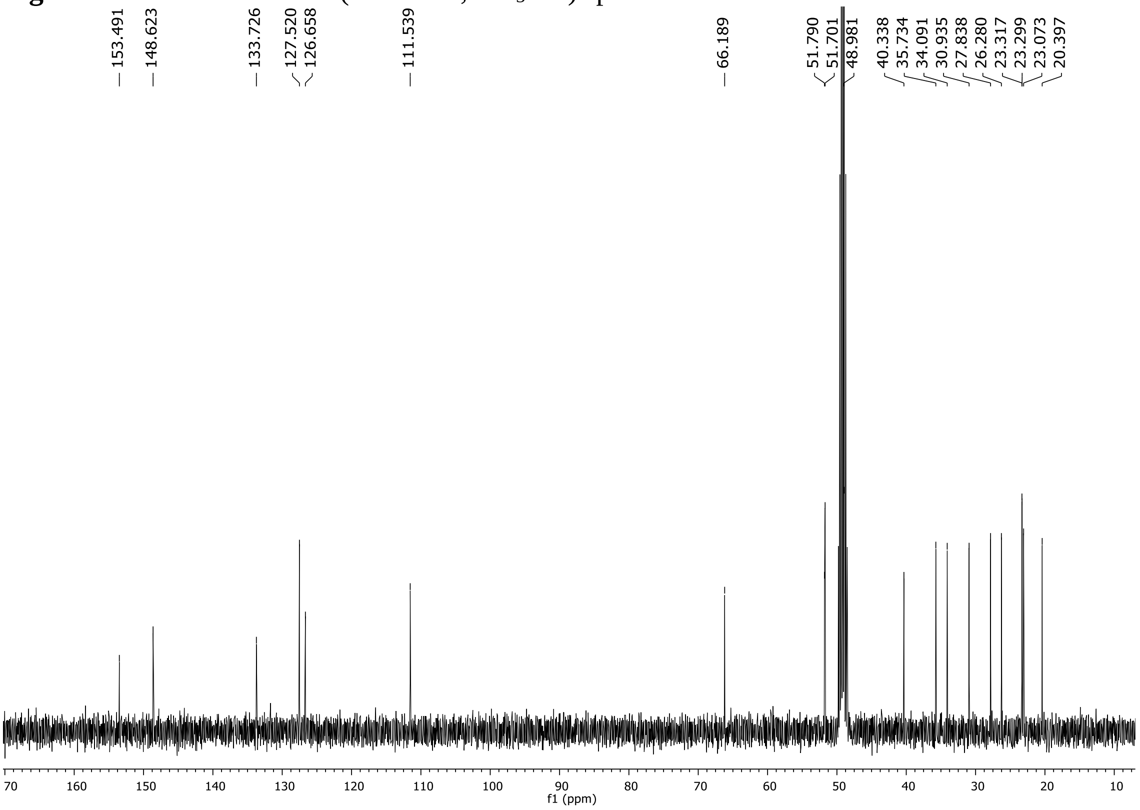

Figure S61. ^{13}C NMR - BB (100 MHz, CD_3OD) spectrum of **4** (expansion).

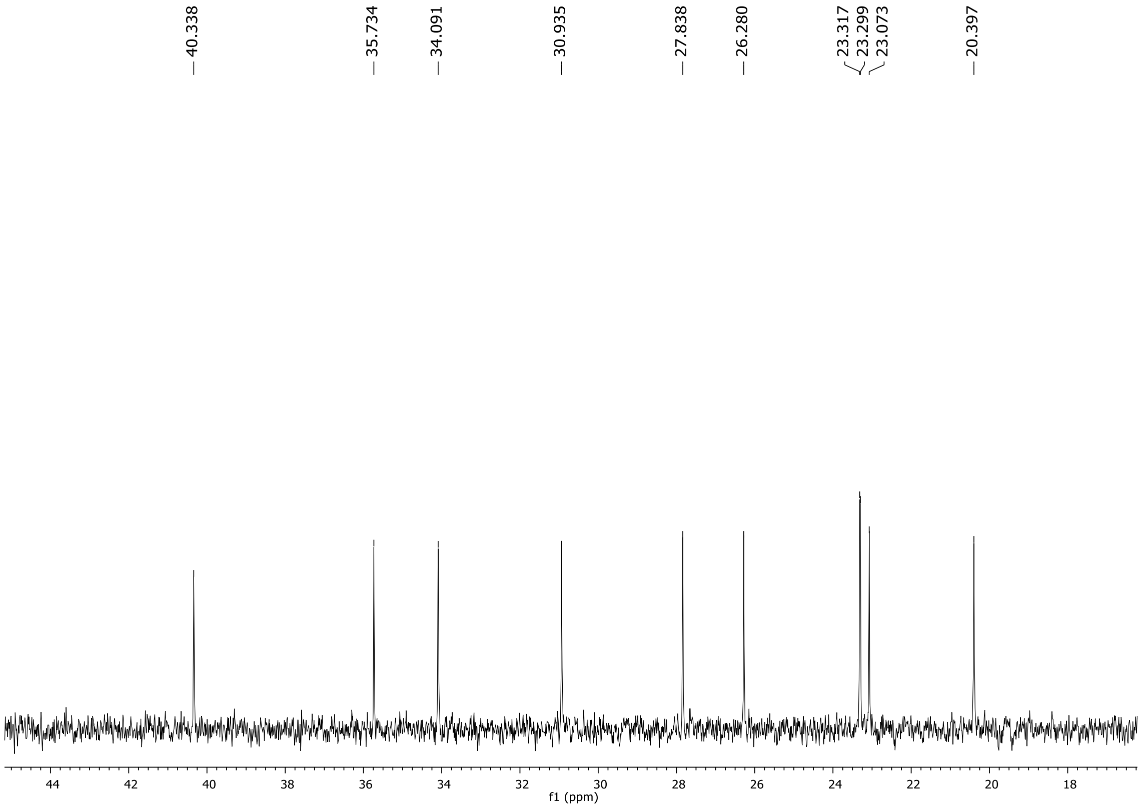

Figure S62. ^{13}C NMR - DEPT 135 (100 MHz, CD_3OD) spectrum of **4**.

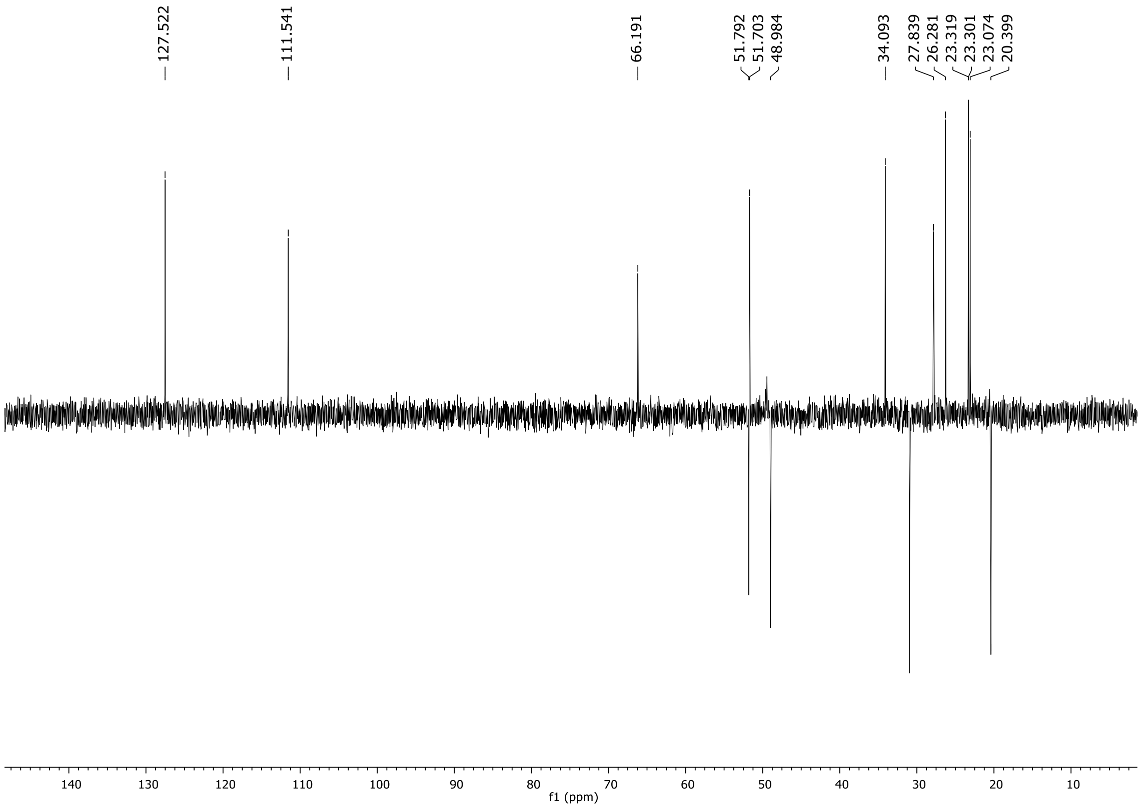

Figure S63. HSQC NMR (400 X 100 MHz, CD_3OD) spectrum of **4**

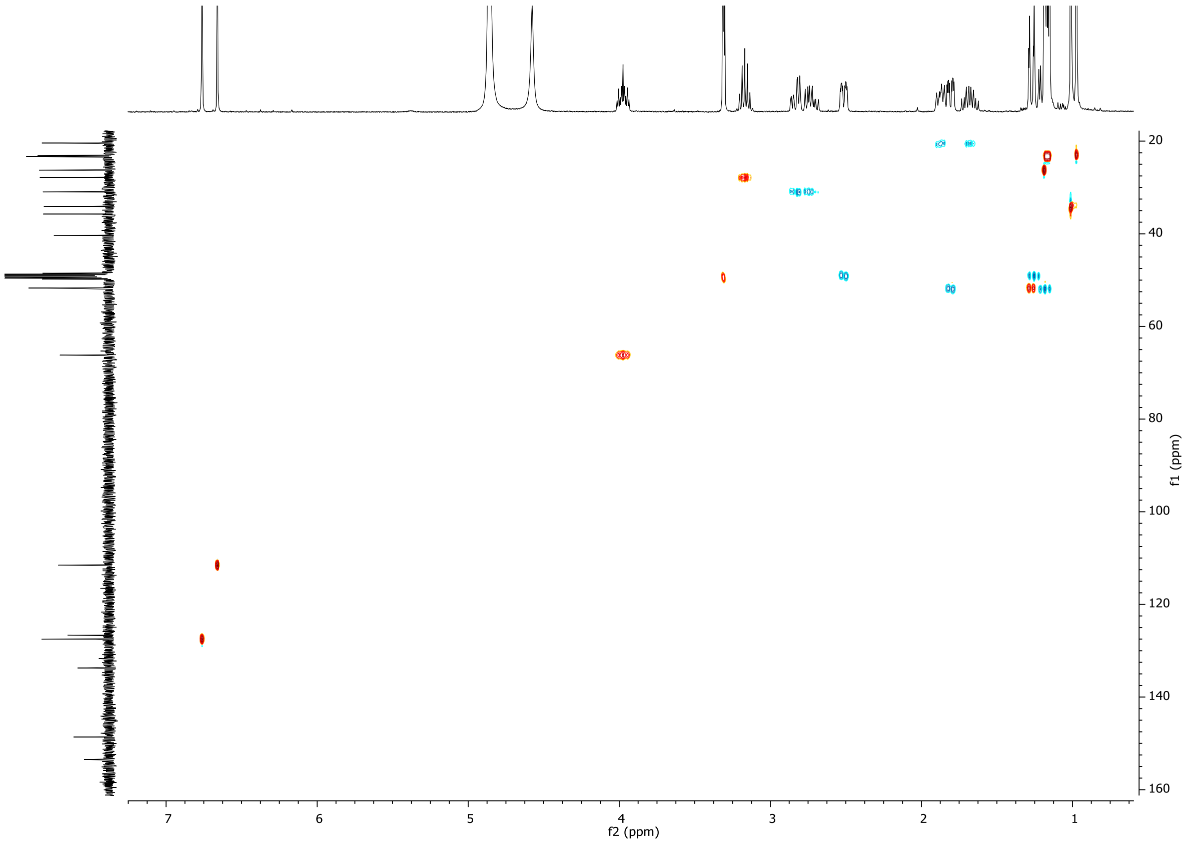

Figure S64. HSQC NMR (400 X 100 MHz, CD_3OD) spectrum of **4** (expansion).

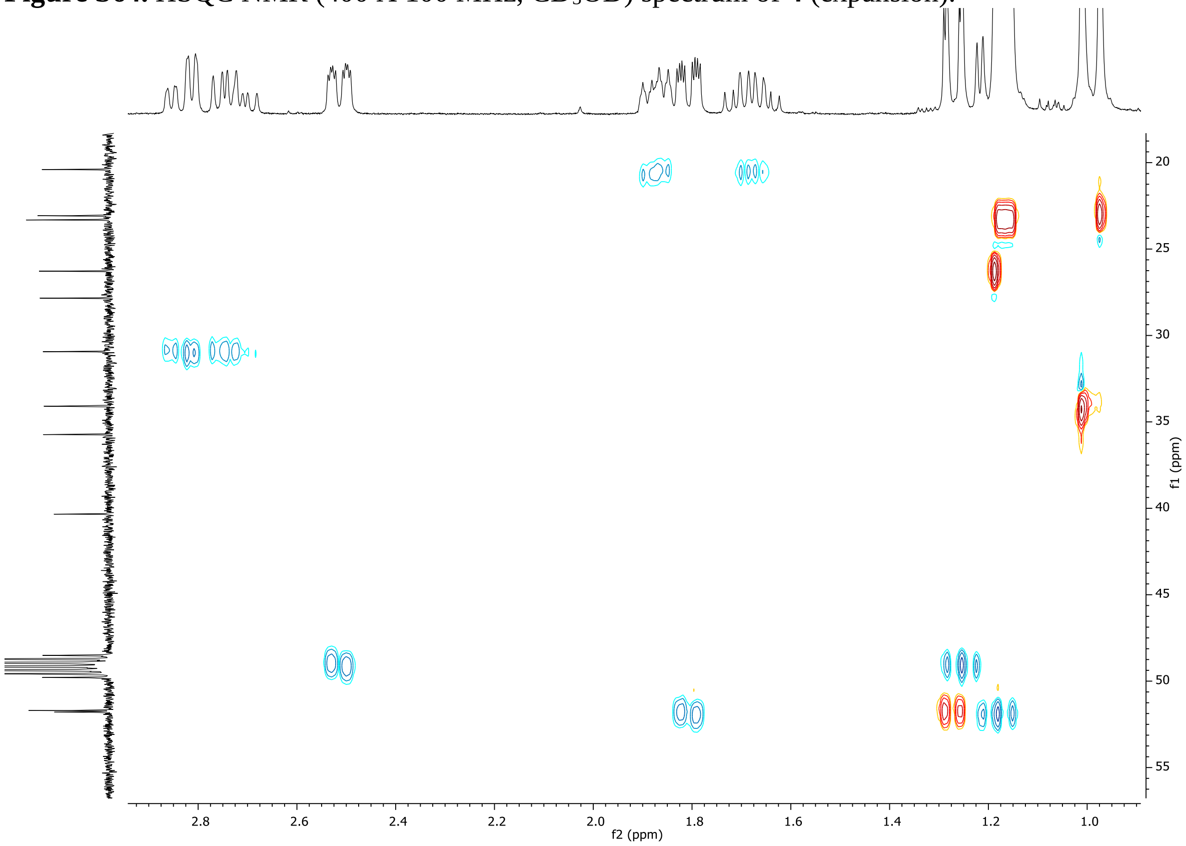

Figure S65. HMBC NMR (400 X 100 MHz, CD_3OD) spectrum of **4.**

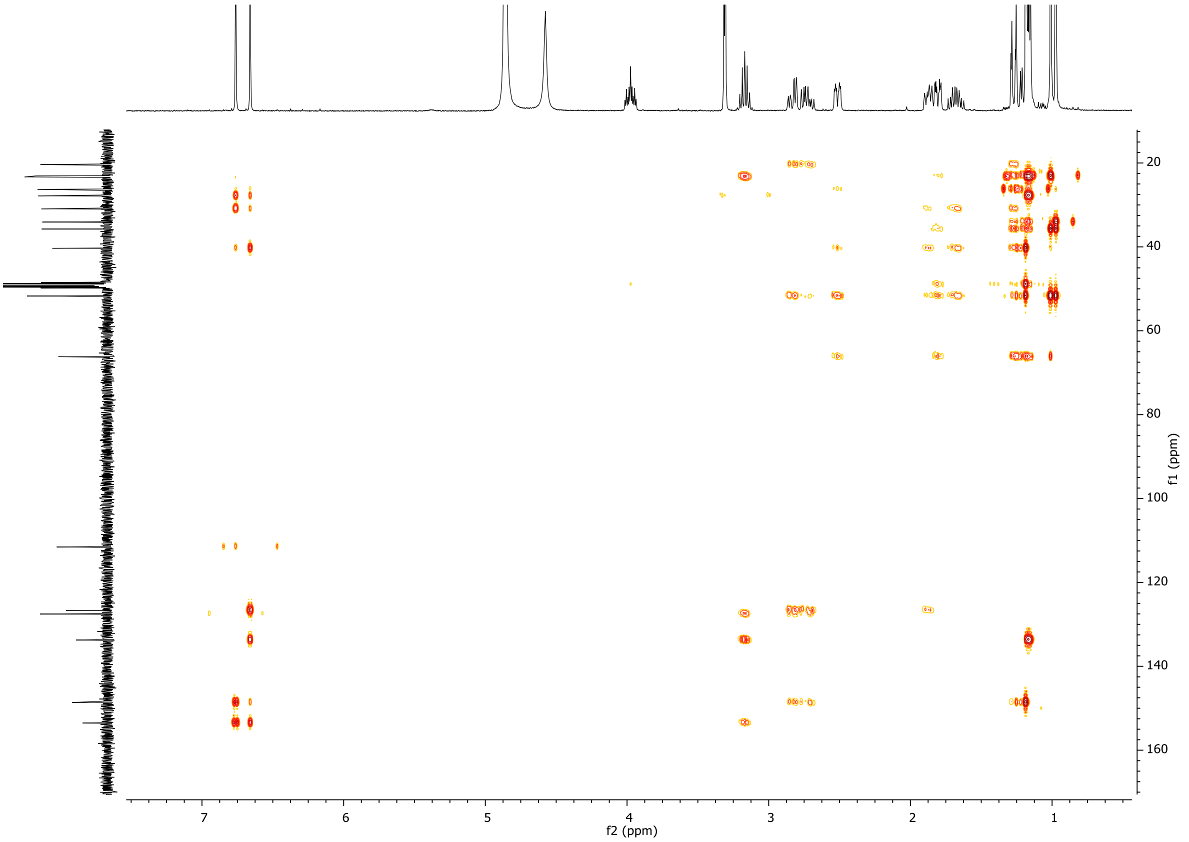

Figure S66. HMBC NMR (400 X 100 MHz, CD_3OD) spectrum of **4** (expansion).

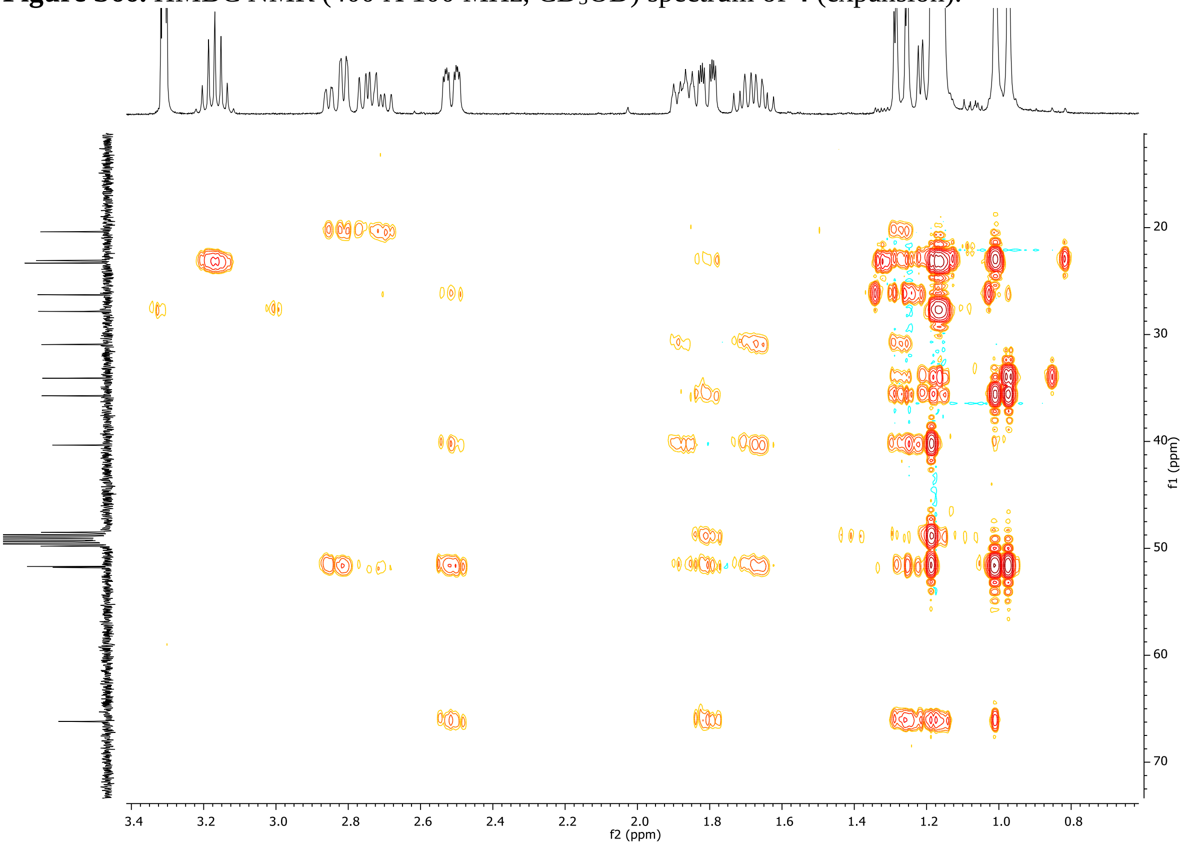

Figure S67. IR spectrum of compound **5**

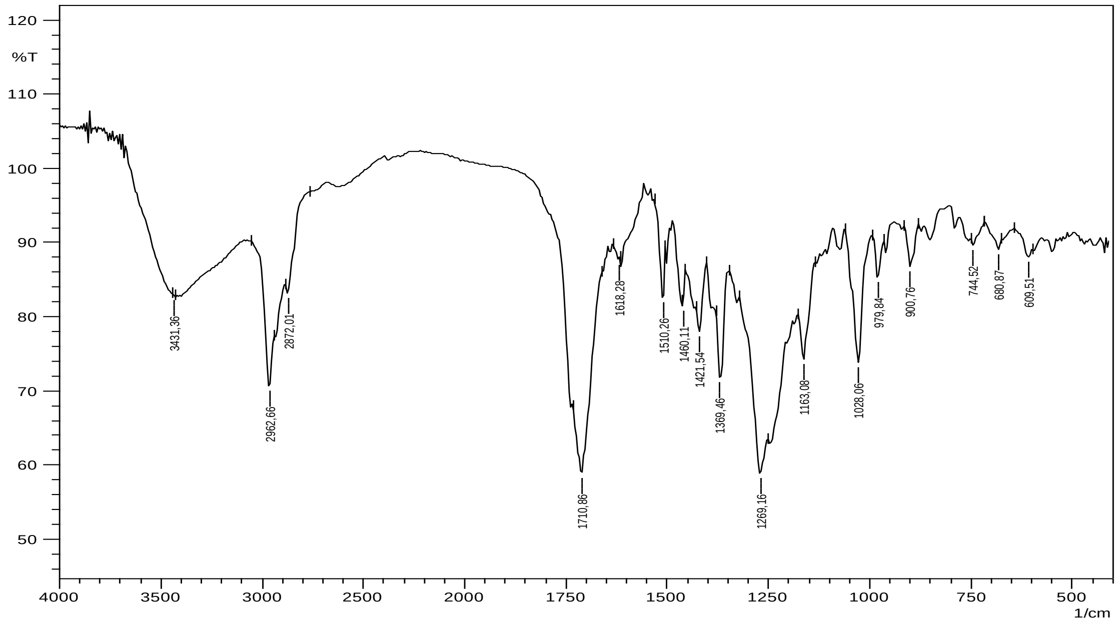

Figure S68. HRESIMS Spectrum of **5** ([M + Na]$^+$ positive ion mode)

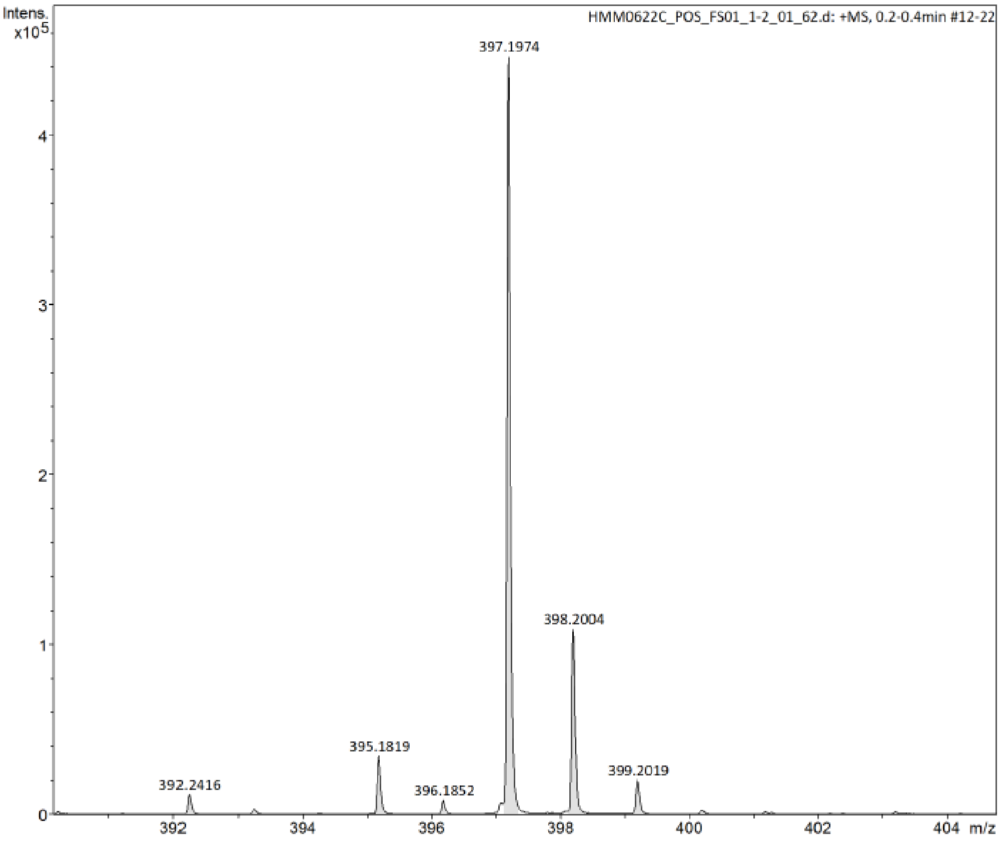

Figure S69. ^{1}H NMR (400 MHz, CD_3OD) spectrum of **5**.

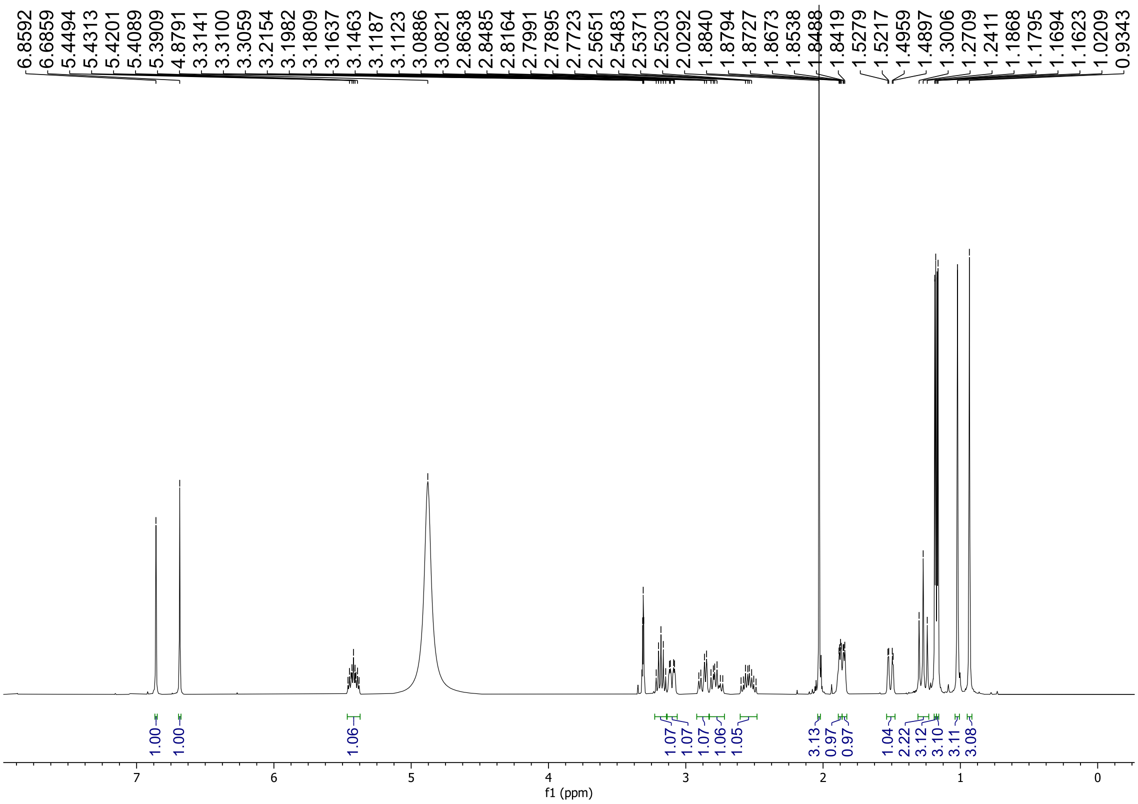

Figure S70. ^{1}H NMR (400 MHz, CD_3OD) spectrum of **5** (expansion).

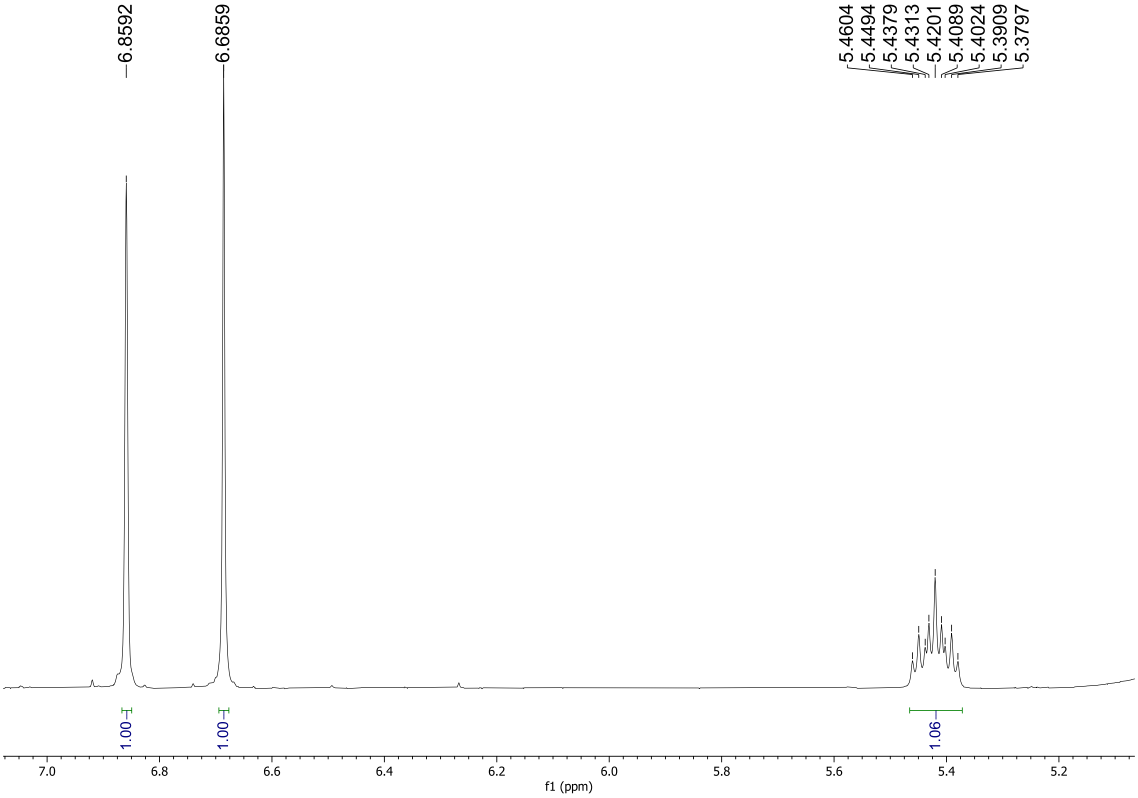

Figure S71. ^{1}H NMR (400 MHz, CD_3OD) spectrum of **5** (expansion).

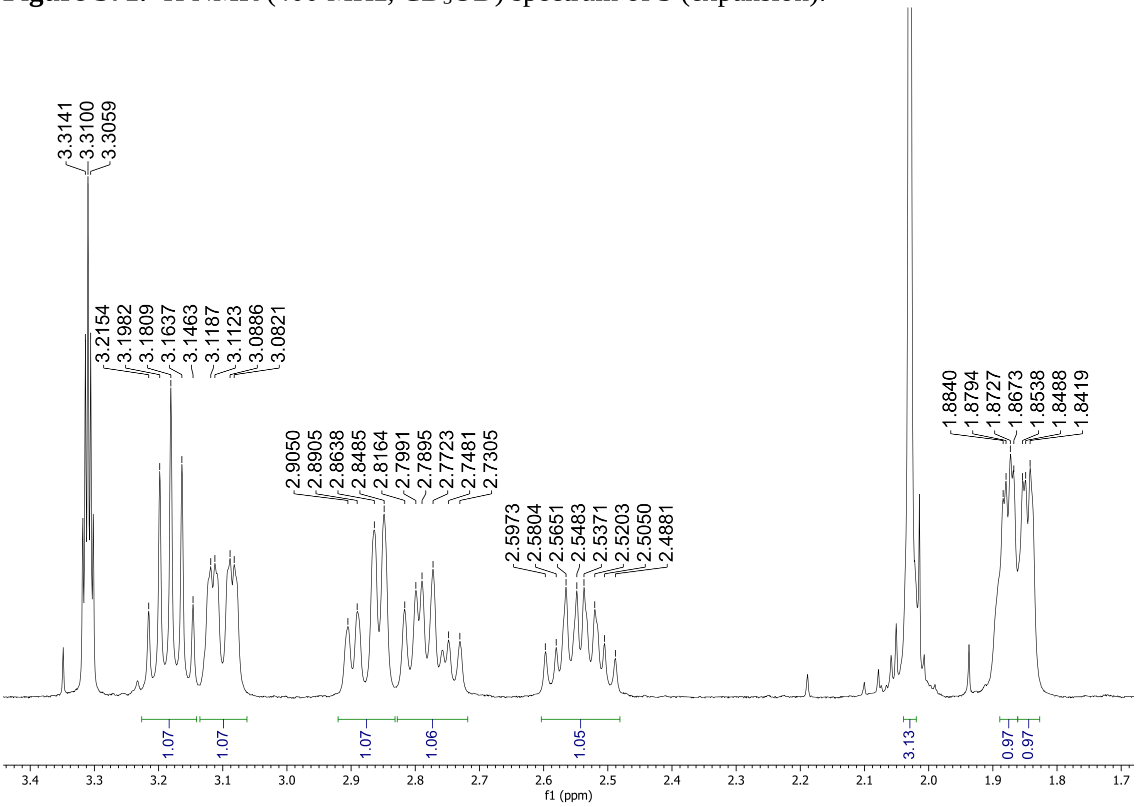

Figure S72. ^{1}H NMR (400 MHz, CD_3OD) spectrum of **5** (expansion).

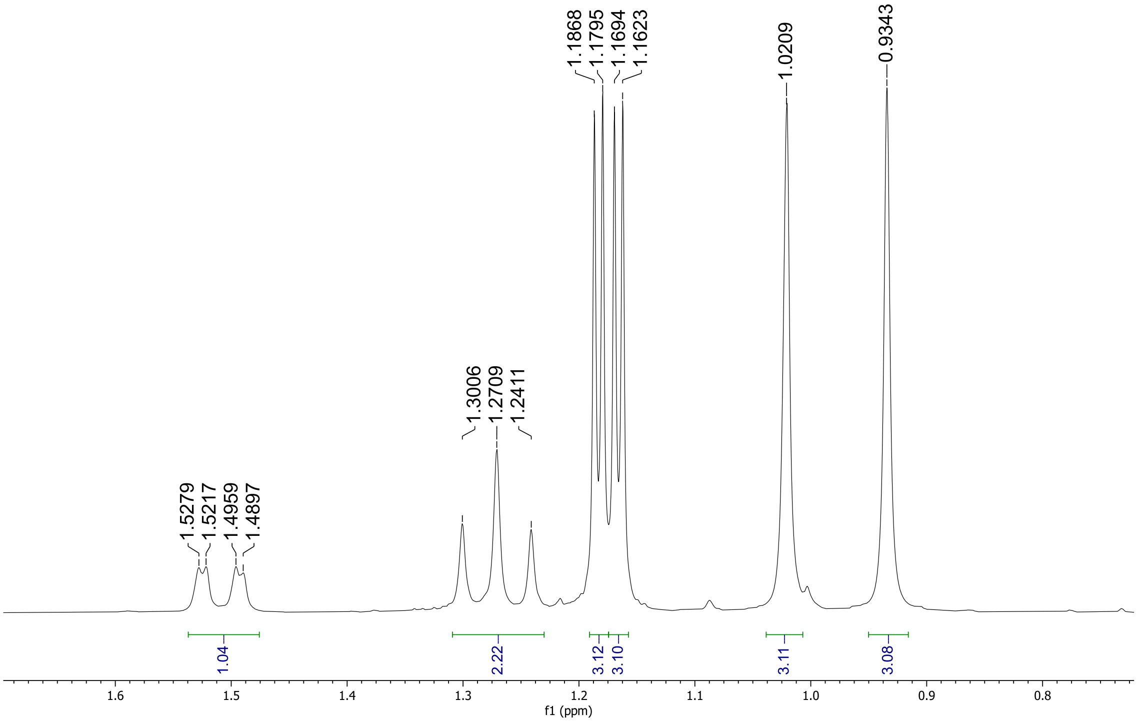

Figure S73. ^{13}C NMR - BB (100 MHz, CD_3OD) spectrum of **5**

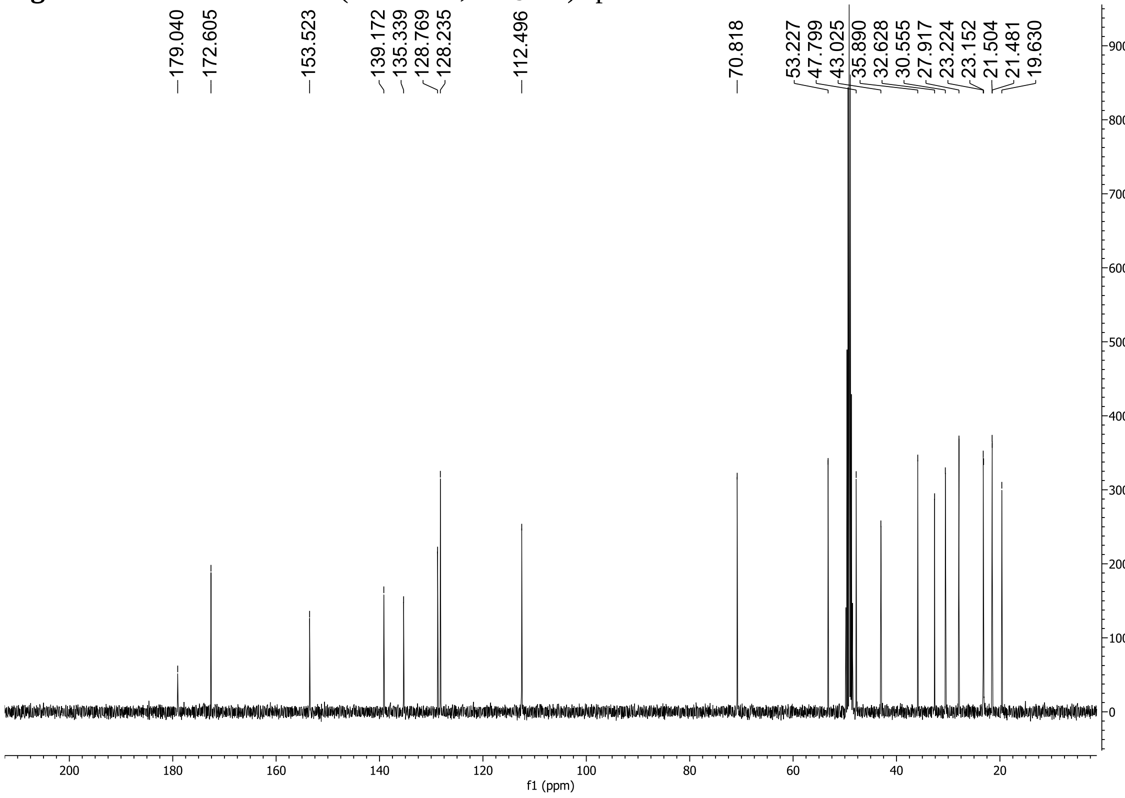

Figure S74. ^{13}C NMR - BB (100 MHz, CD_3OD) spectrum of **5** (expansion).

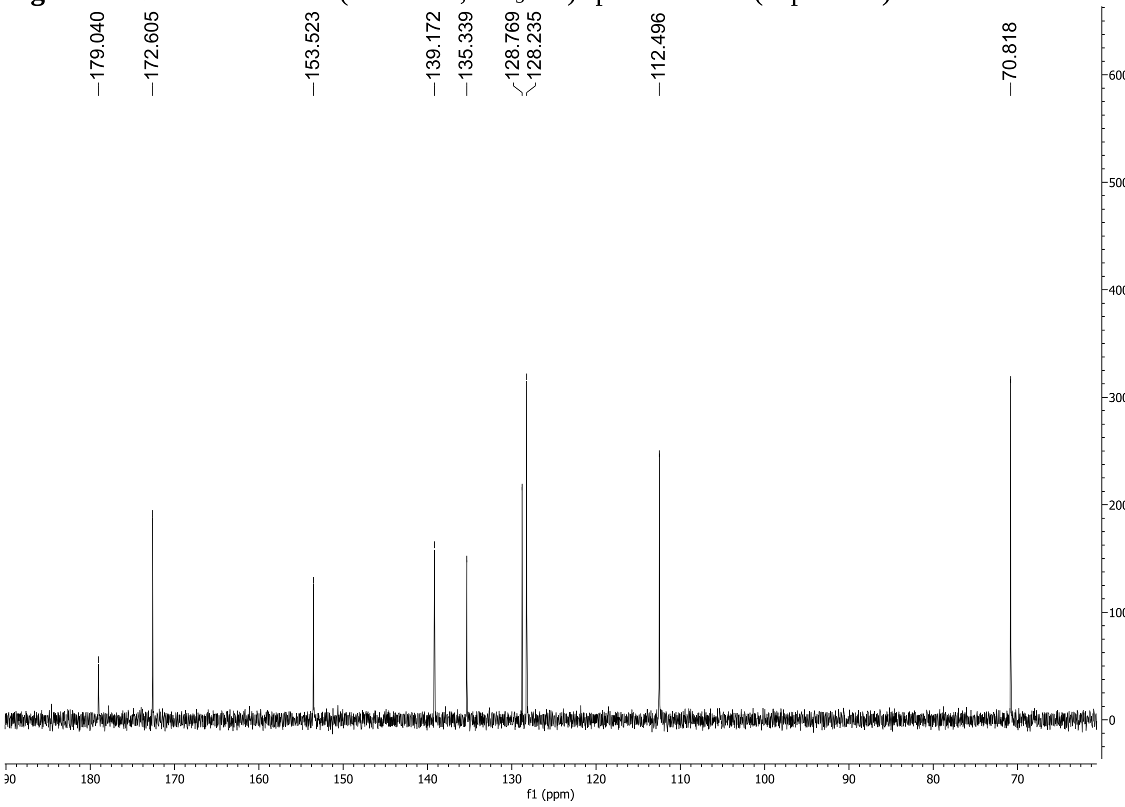

Figure S75. ^{13}C NMR - BB (100 MHz, CD_3COCD_3) spectrum of **5**

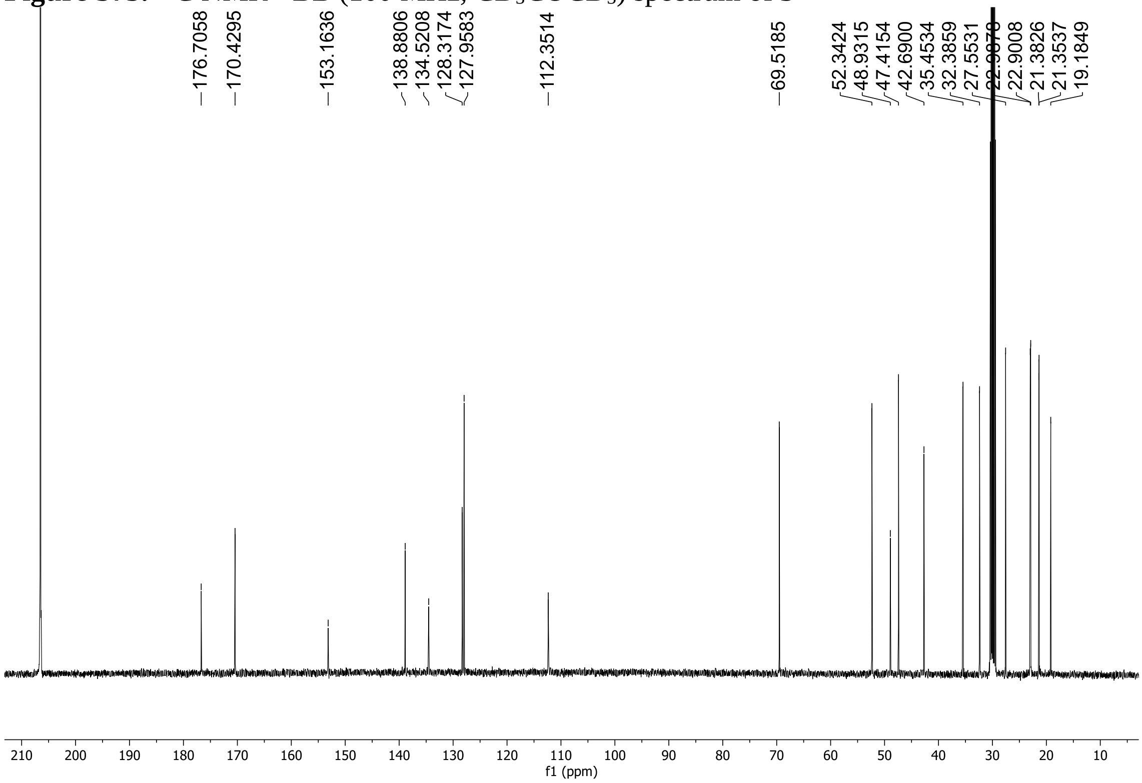

Figure S76. ^{13}C NMR – DEPT 135 (100 MHz, CD_3OD) spectrum of **5**

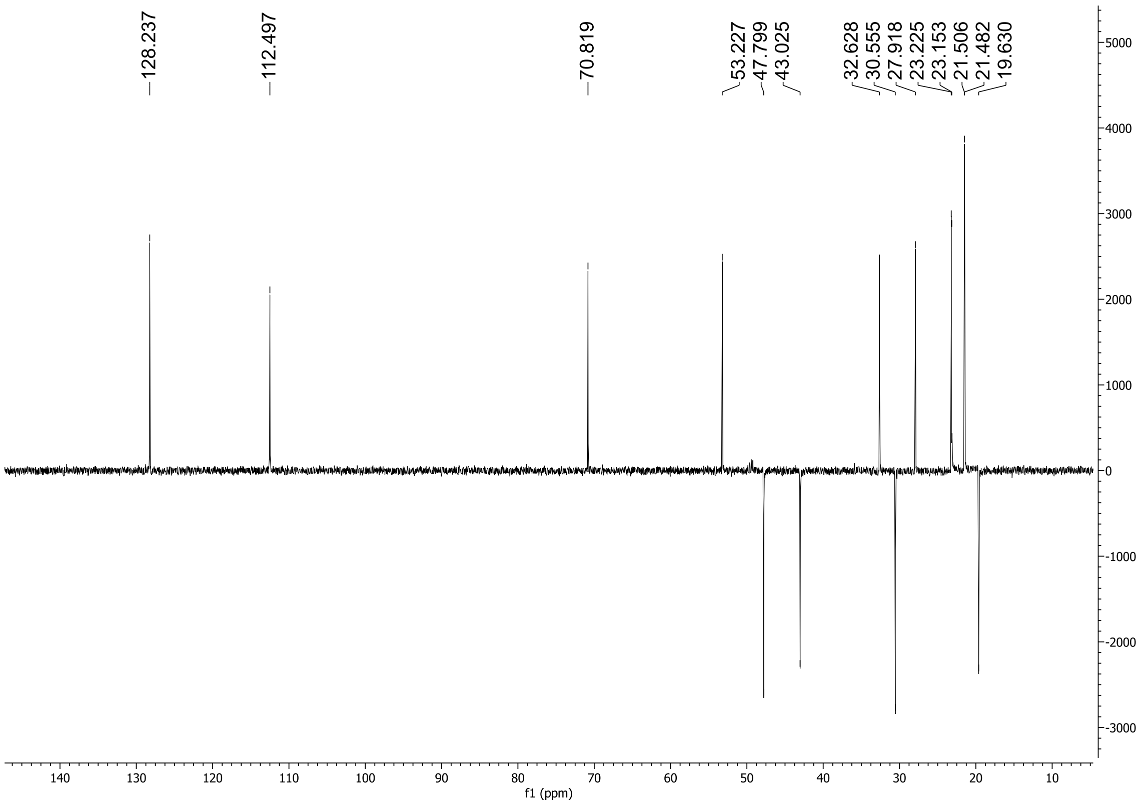

Figure S77. HSQC NMR (400 X 100 MHz, CD_3OD) spectrum of **5**

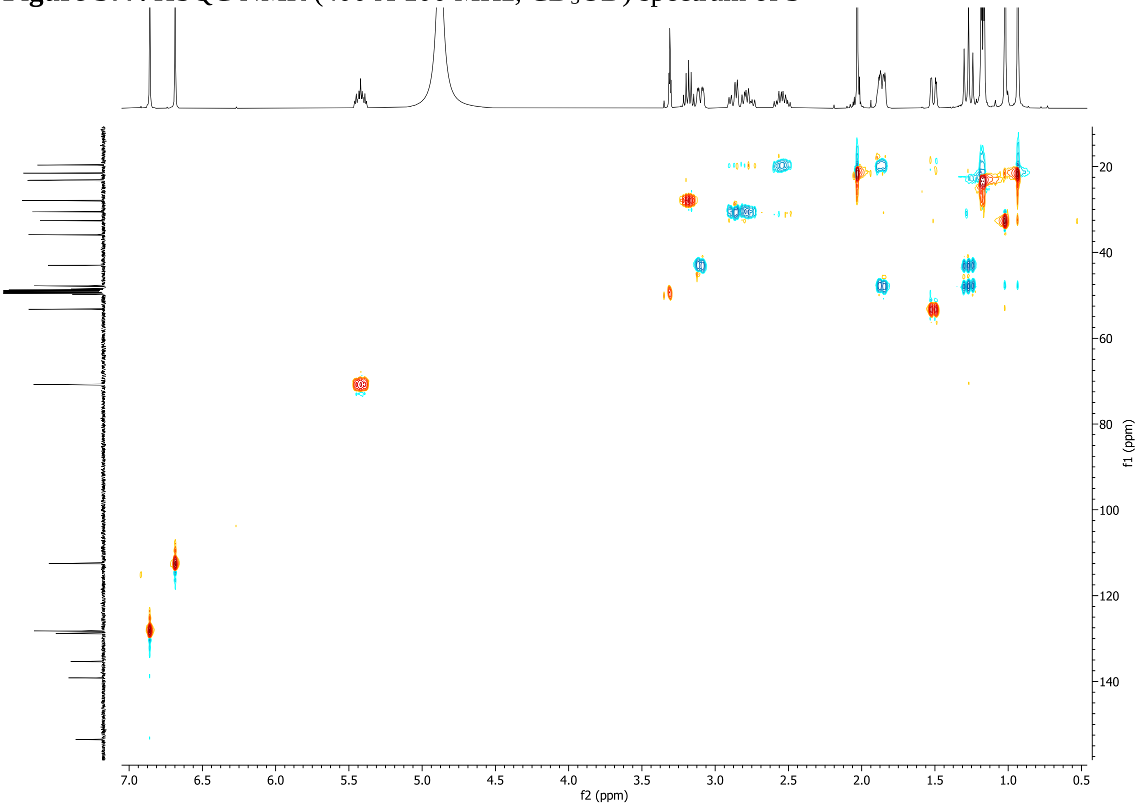

Figure S78. HSQC NMR (400 X 100 MHz, CD_3OD) spectrum of **5** (expansion).

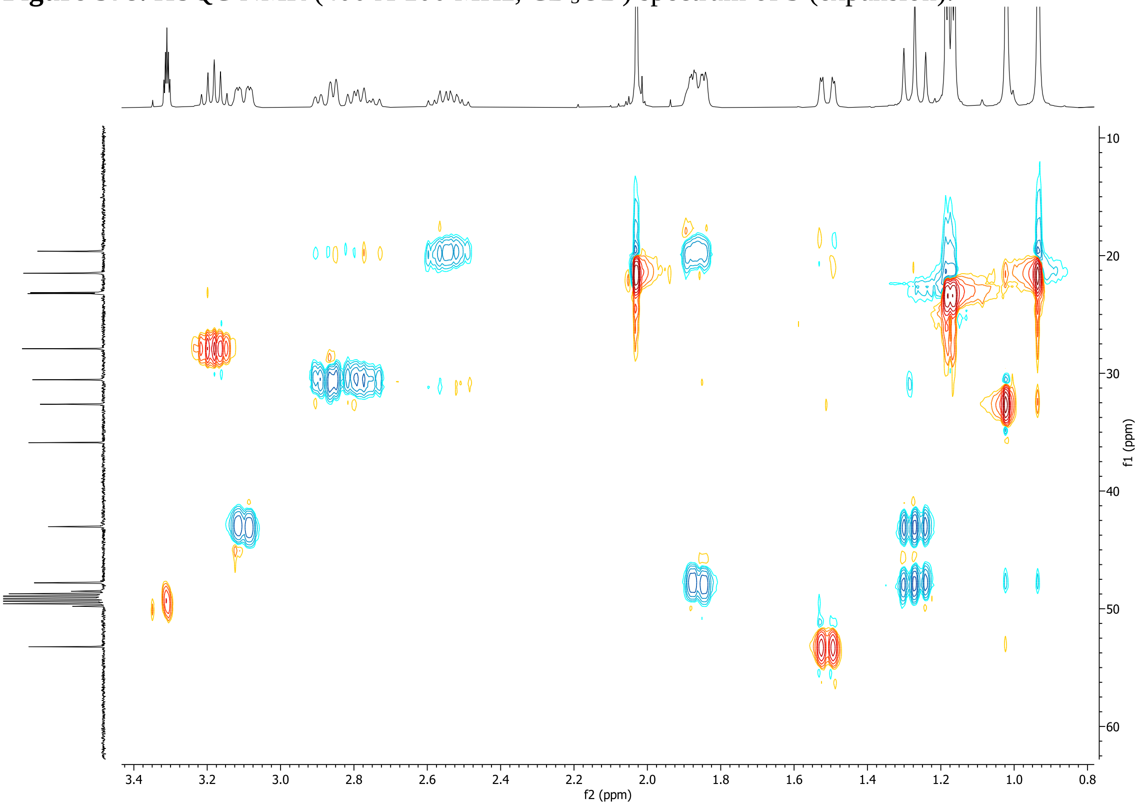

Figure S79. HMBC NMR (400 X 100 MHz, CD_3OD) spectrum of **5**

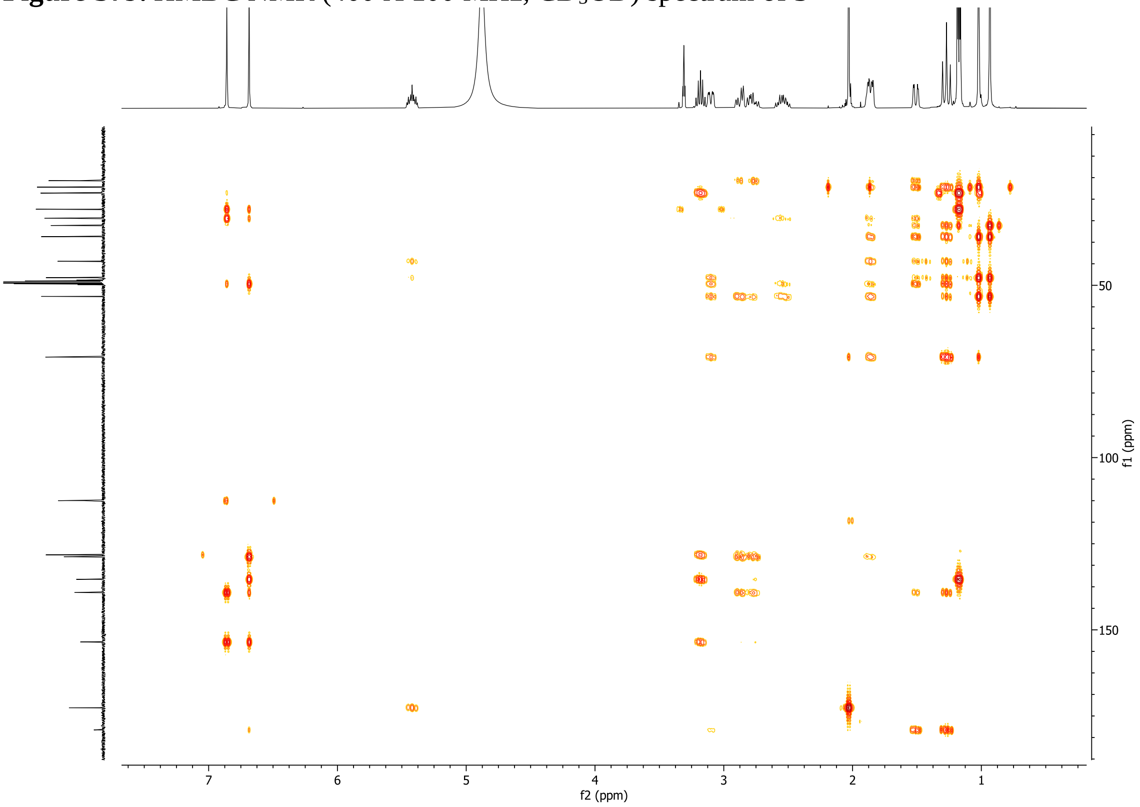

Figure S80. HMBC NMR (400 X 100 MHz, CD_3OD) spectrum of **5** (expansion).

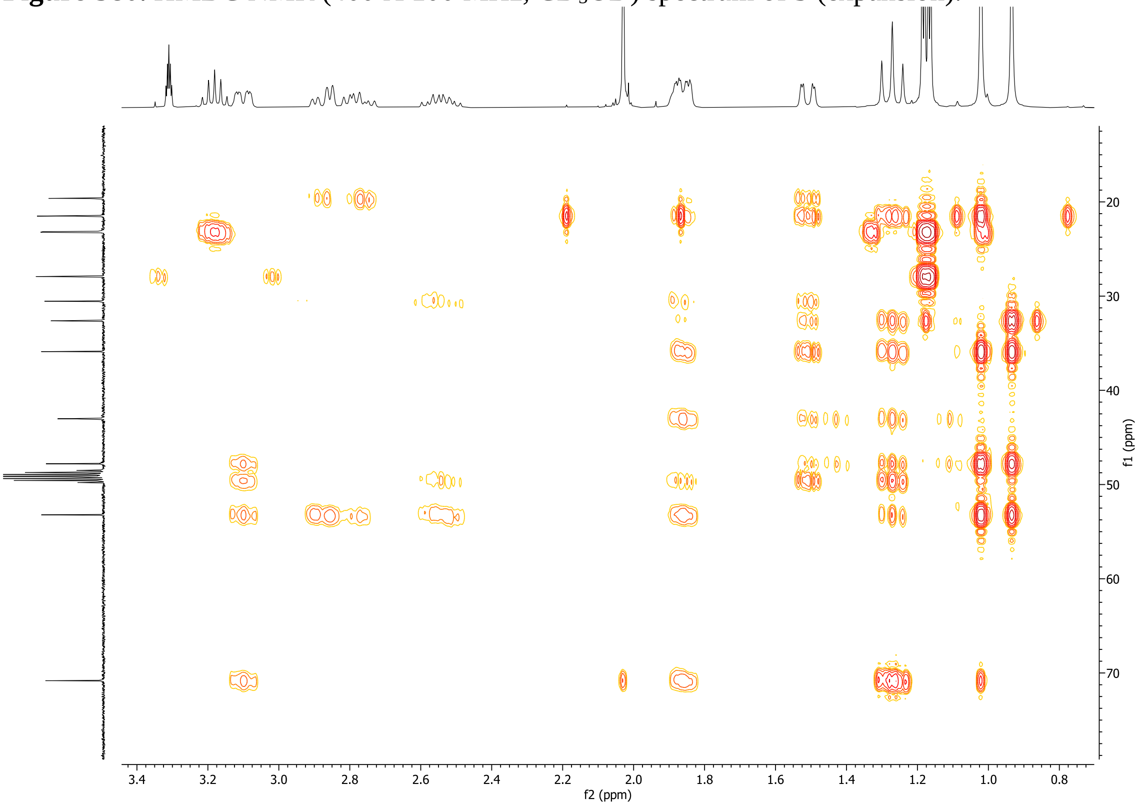

Figure S81. HMBC NMR (400 X 100 MHz, CD_3OD) spectrum of **5** (expansion).

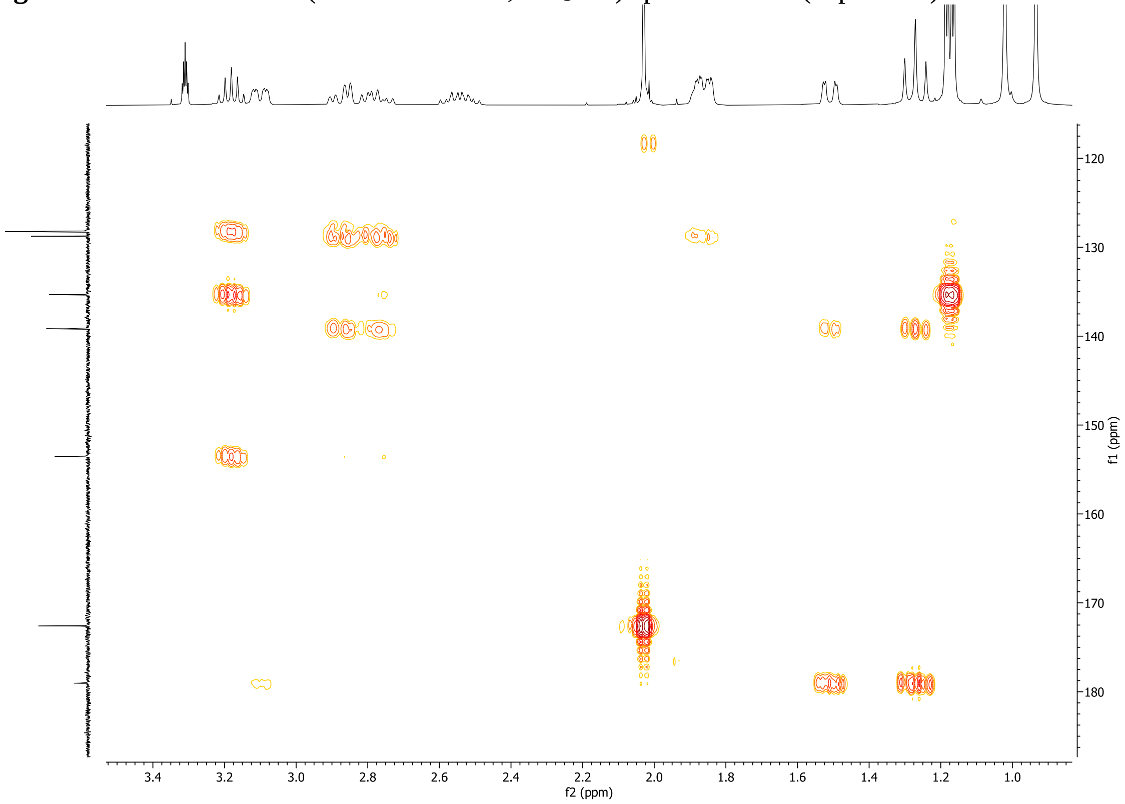

Figure S82. COSY NMR (400 MHz, CD_3OD) spectrum of **5**

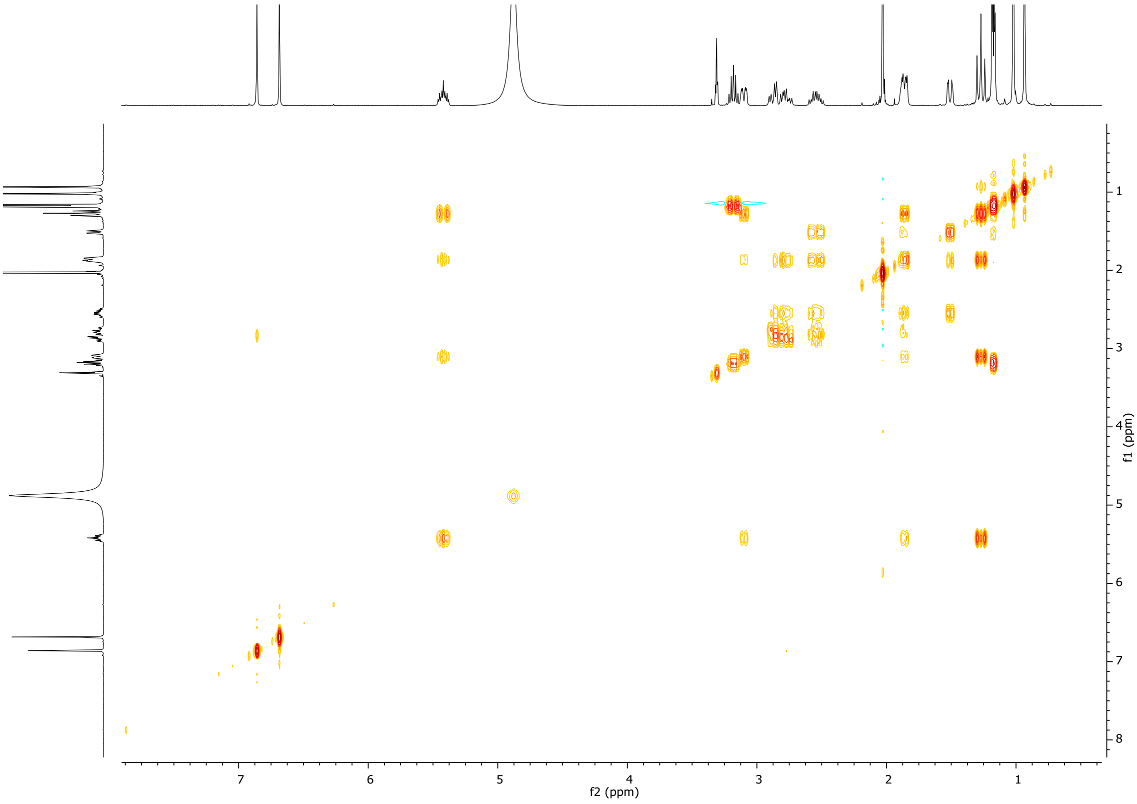

Figure S83. COSY NMR (400 MHz, CD_3OD) spectrum of **5** (expansion).

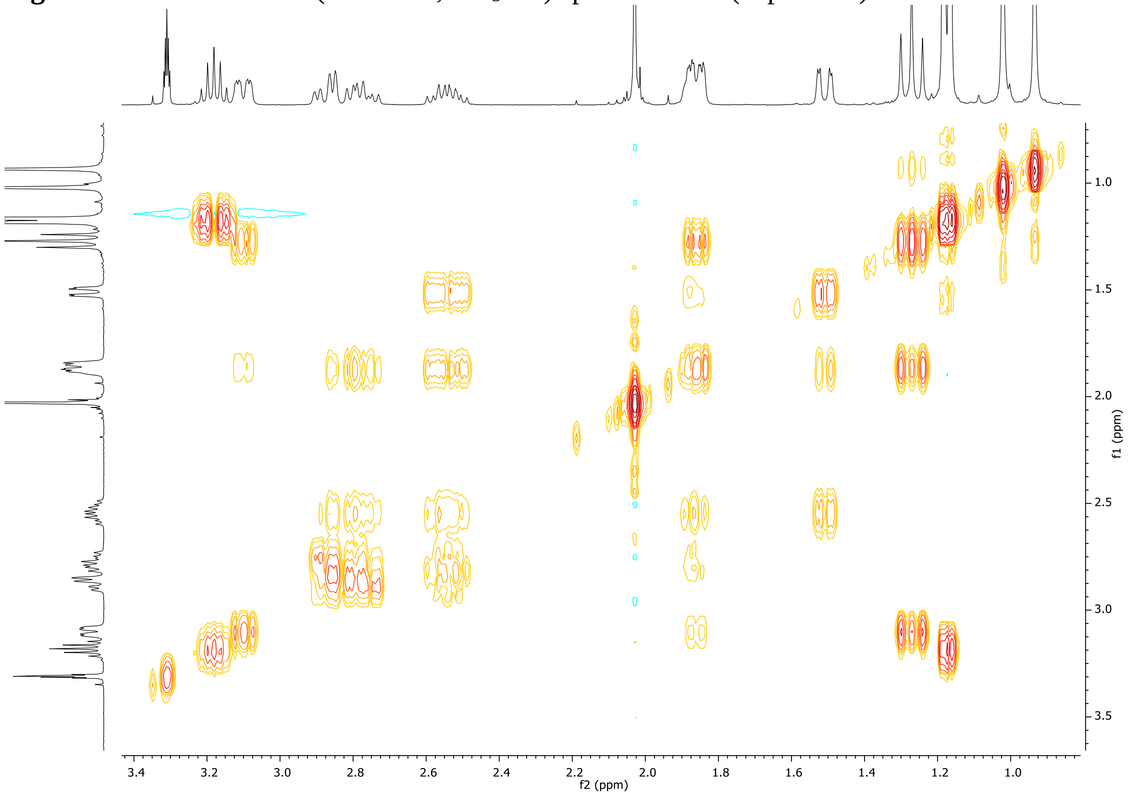

Figure S84. COSY NMR (400 MHz, CD_3OD) spectrum of **5** (expansion).

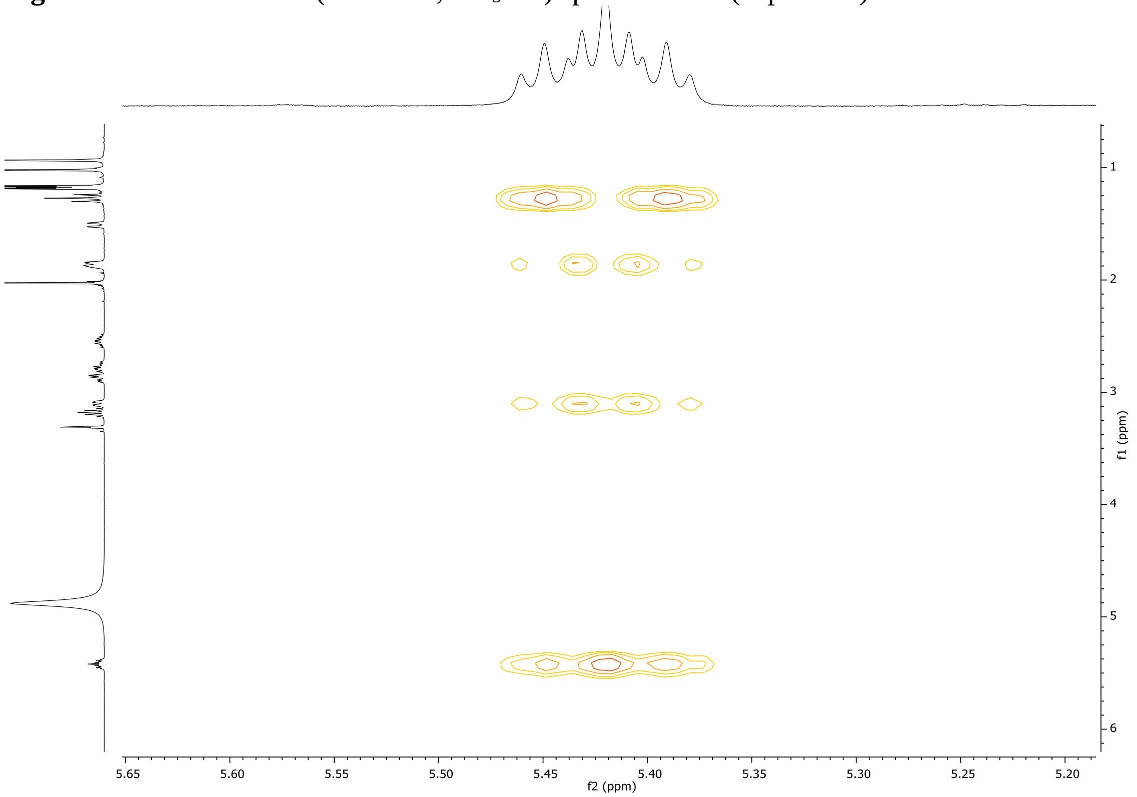

Figure S85. NOESY NMR (400 MHz, CD_3COCD_3) spectrum of **5.**

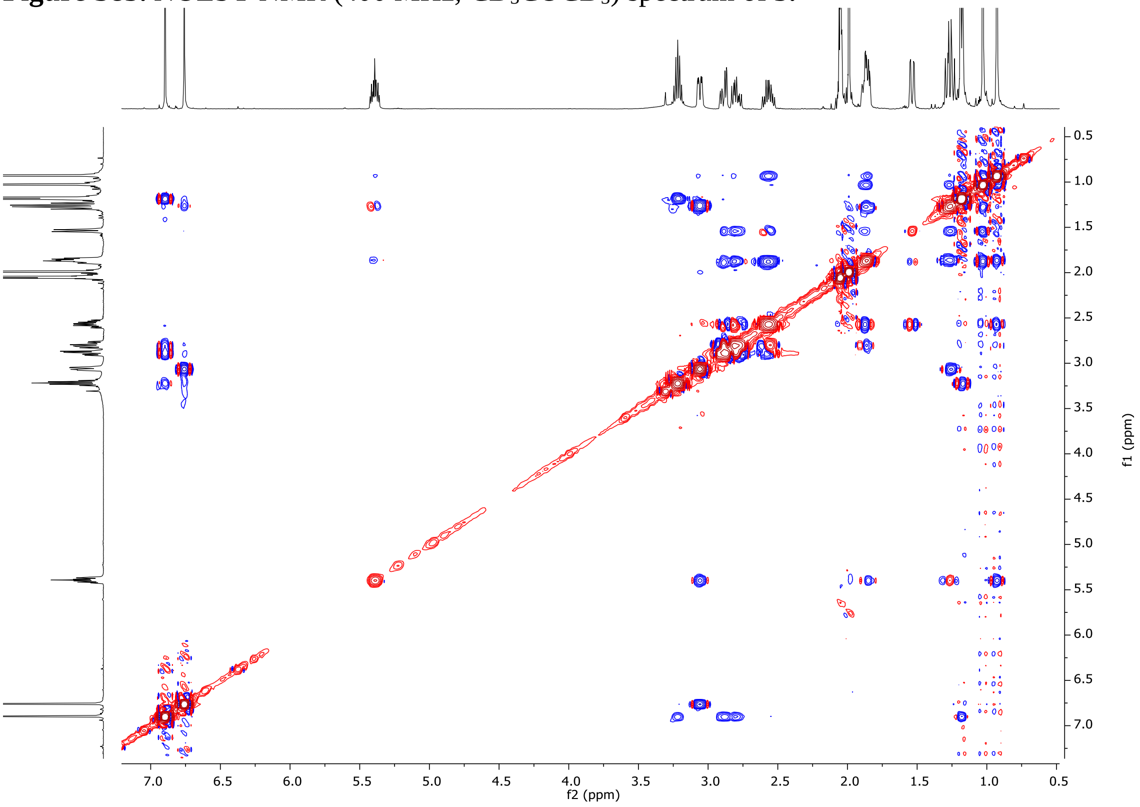

Figure S86. IR spectrum of compound **6**.

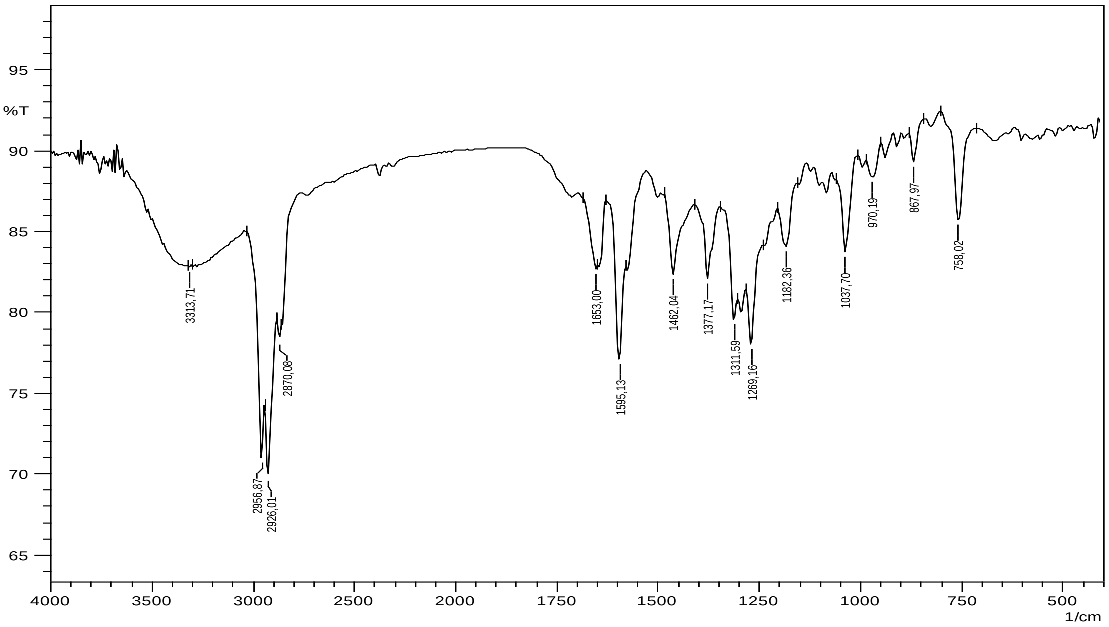

Figure S87. HRESIMS Spectrum of **6** ([2M + Na]$^+$ positive ion mode)

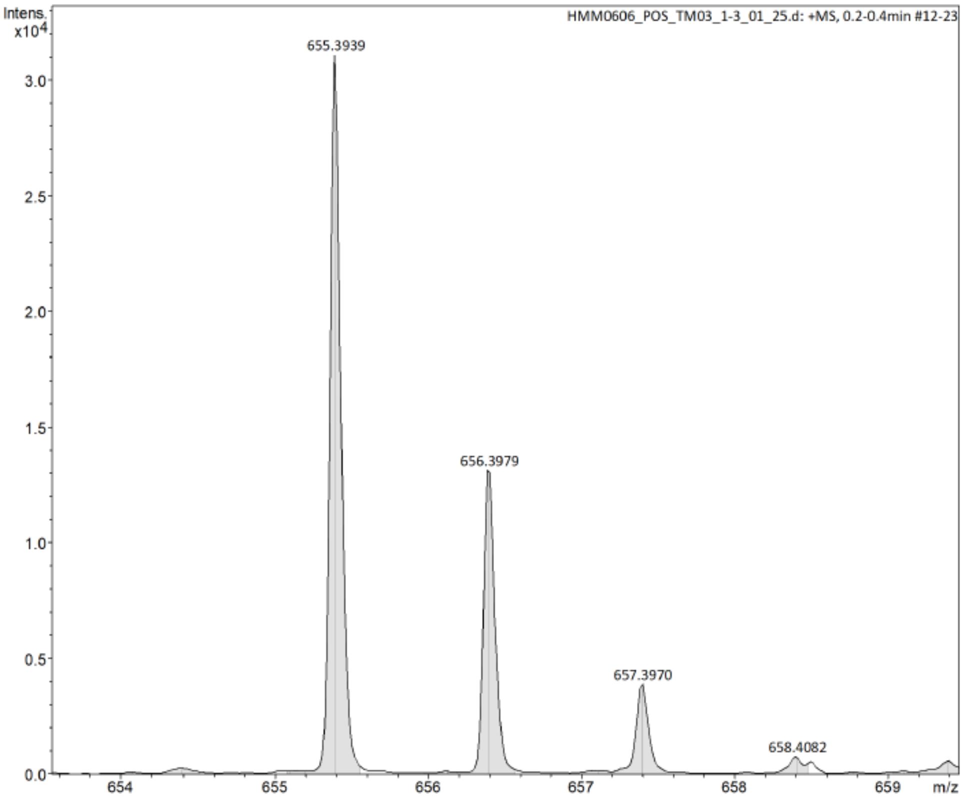

Figure S88. ^{1}H NMR (400 MHz, $CDCl_3$) spectrum of **6.**

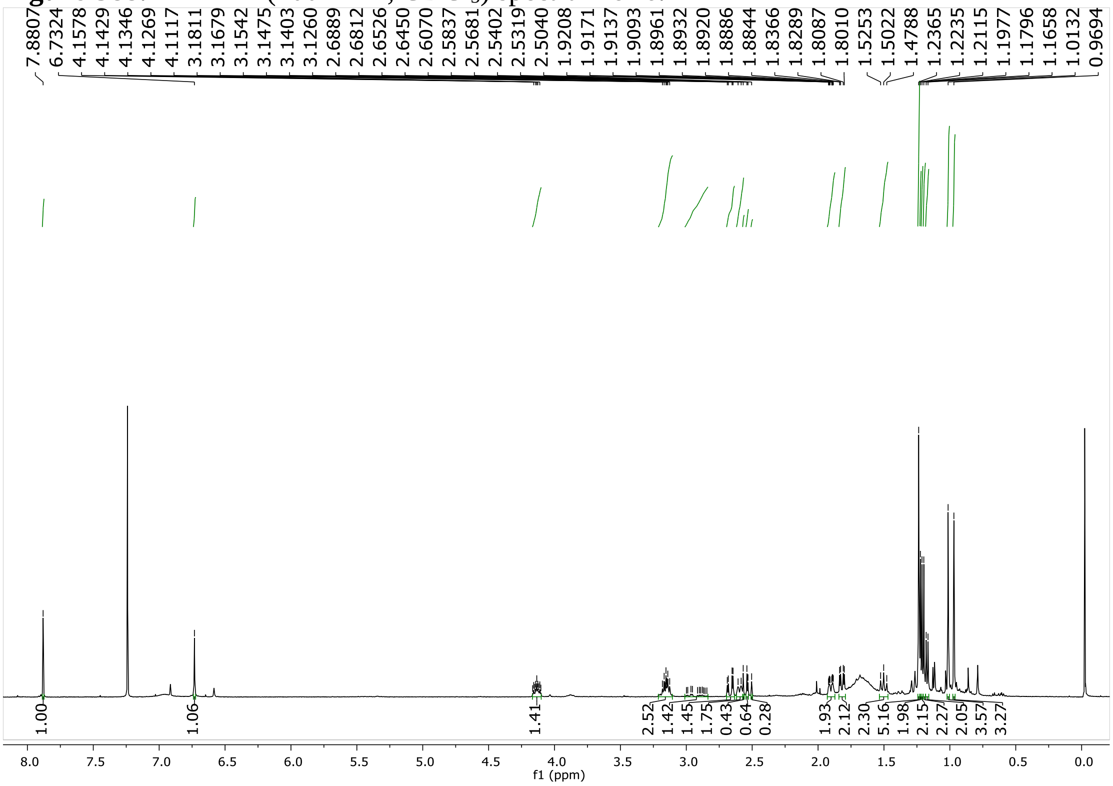

Figure S89. ^{1}H NMR (400 MHz, $CDCl_3$) spectrum of **6** (expansion).

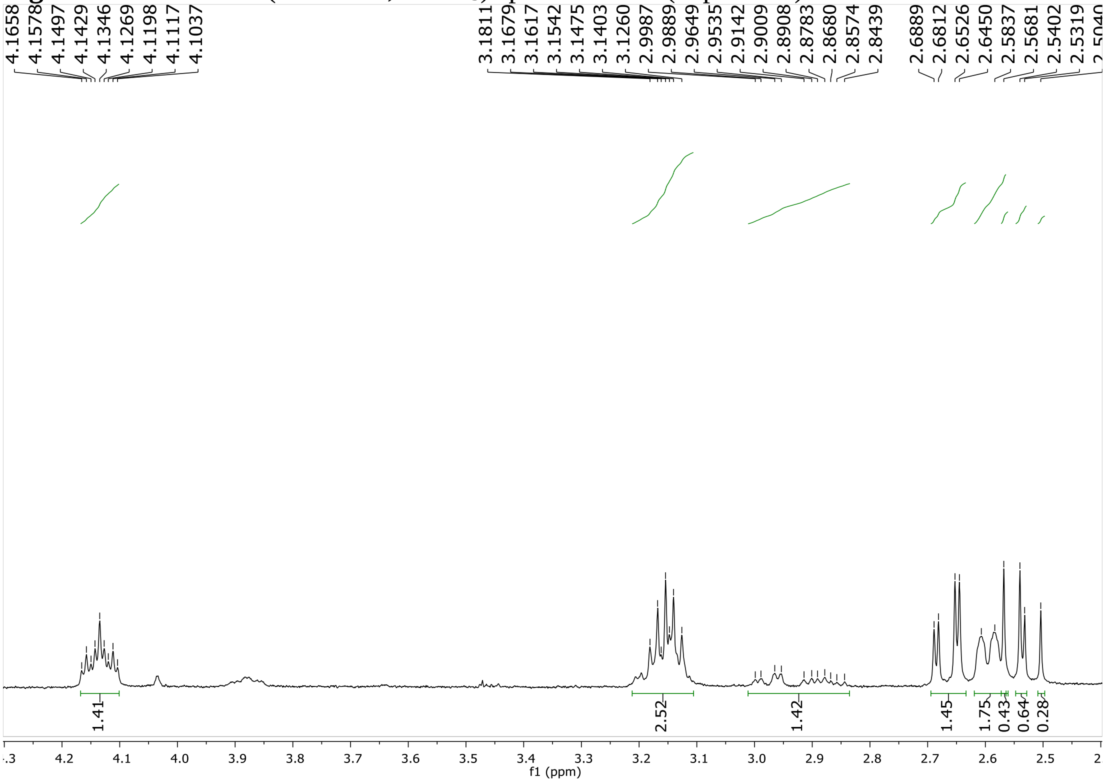

Figure S90. ^{1}H NMR (400 MHz, $CDCl_3$) spectrum of **6** (expansion).

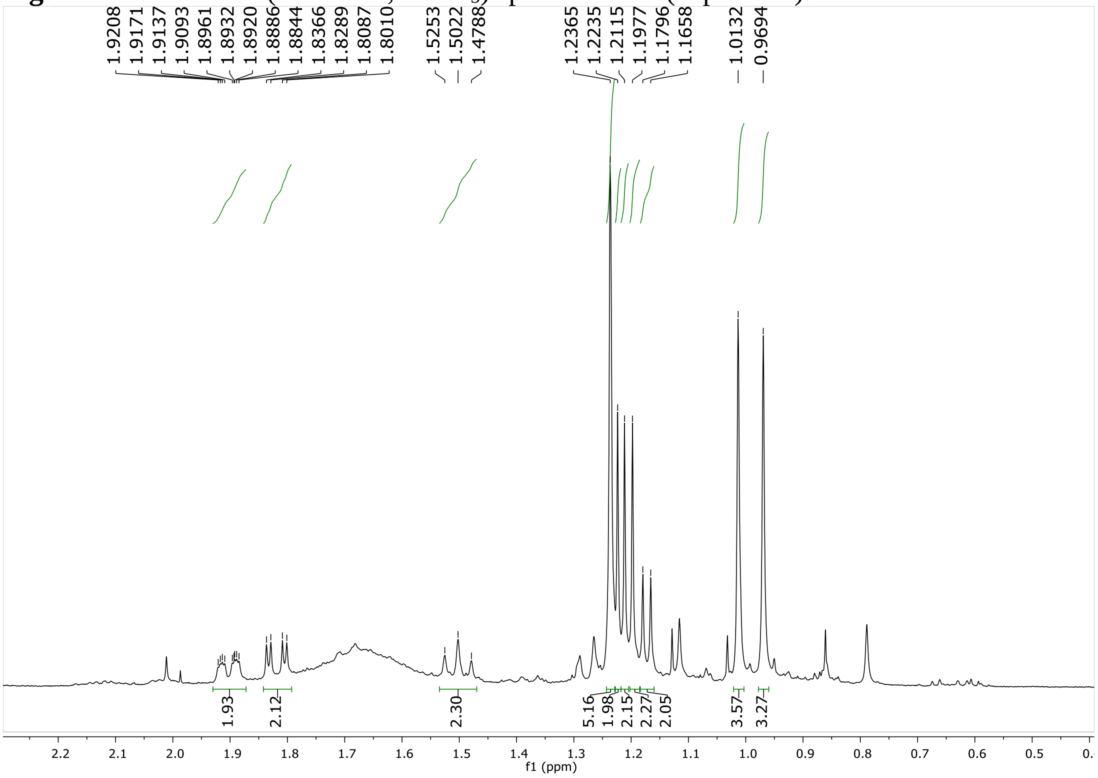

Figure S91. ^{13}C NMR - BB (125 MHz, $CDCl_3$) spectrum of **6.**

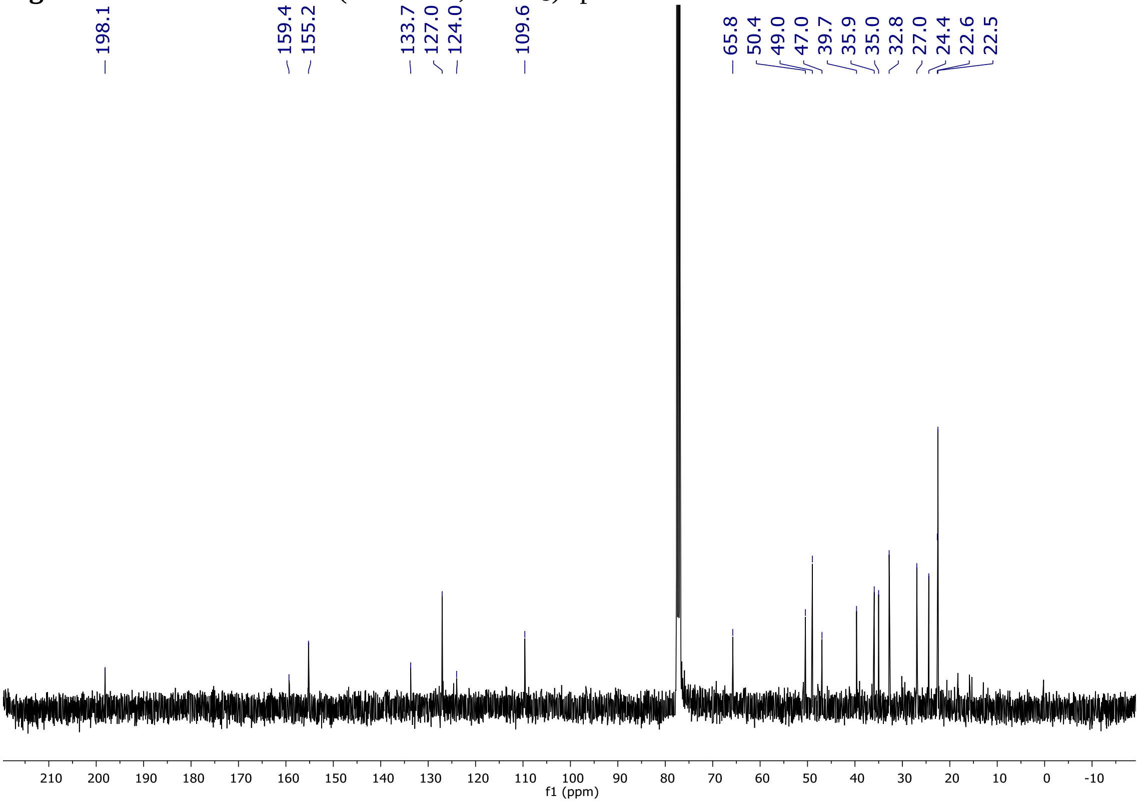

Figure S92. ^{13}C NMR – DEPT 135 (125 MHz, $CDCl_3$) spectrum of **6**.

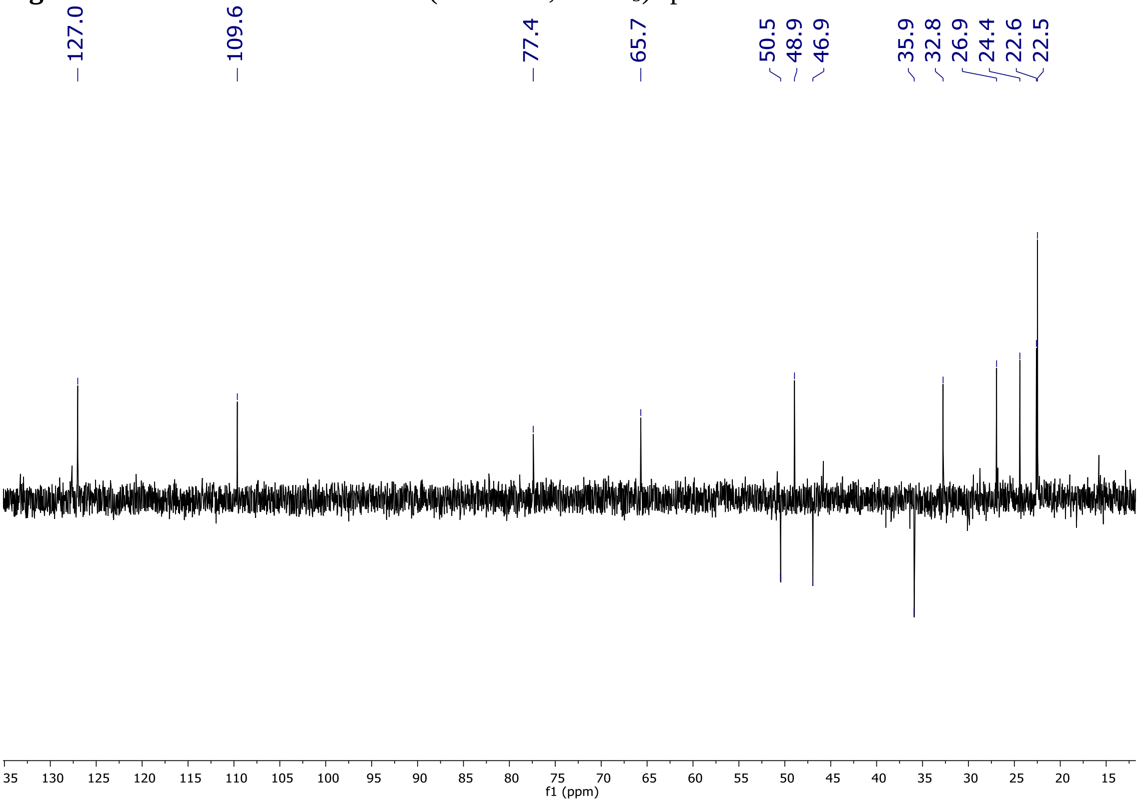

Figure S93. IR spectrum of compound **7**

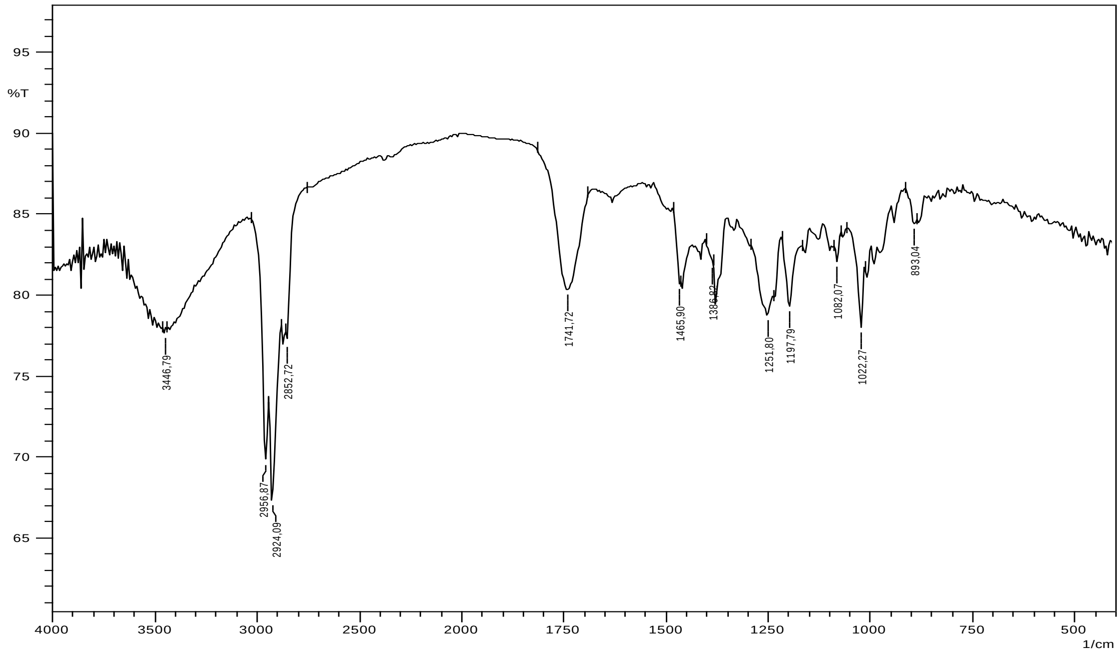

Figure S94. HRESIMS Spectrum of **7** ([2M + Na]$^+$ positive ion mode)**.**

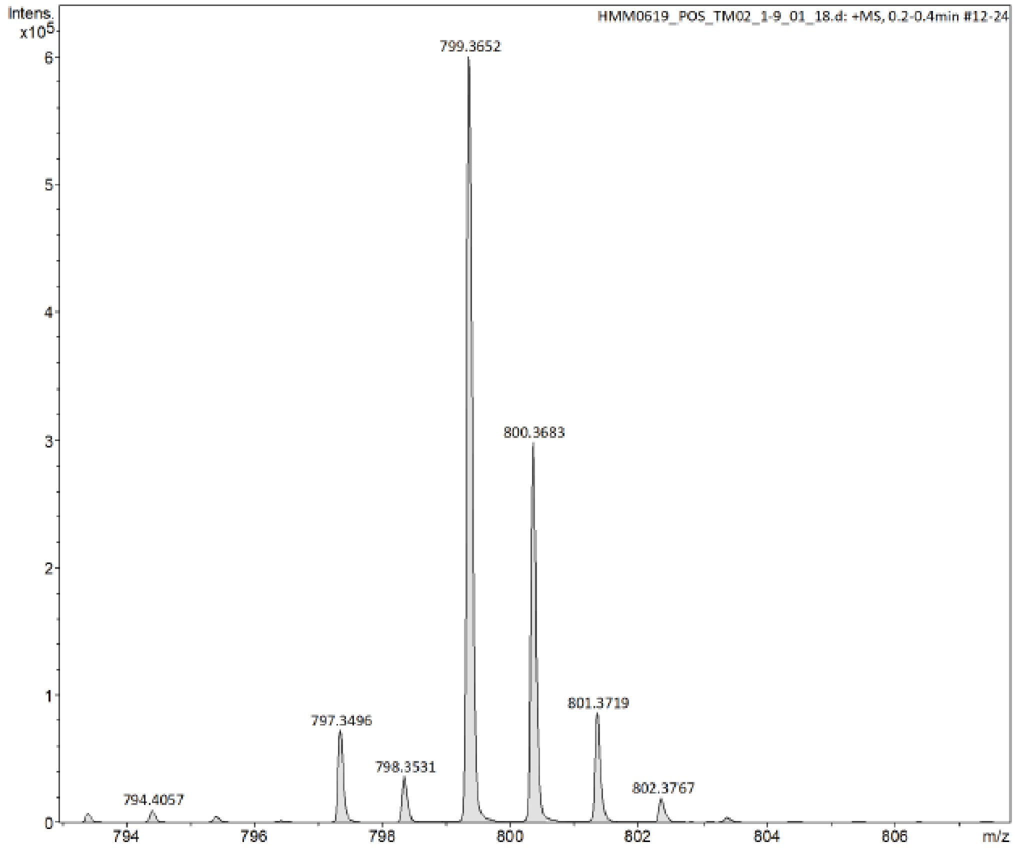

Figure S95. ^{1}H NMR (500 MHz, $CDCl_3$) spectrum of **7**

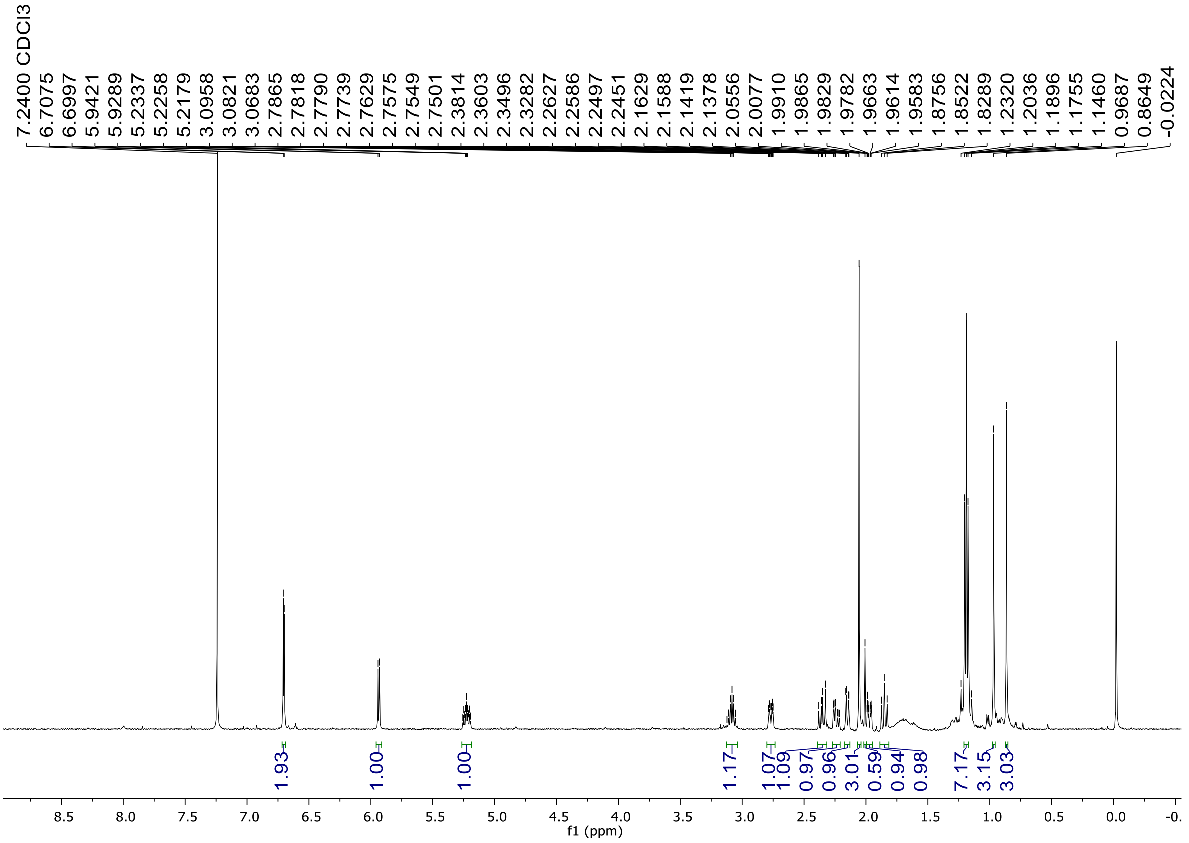

Figure S96. ^{1}H NMR (500 MHz, $CDCl_3$) spectrum of **7** (expansion).

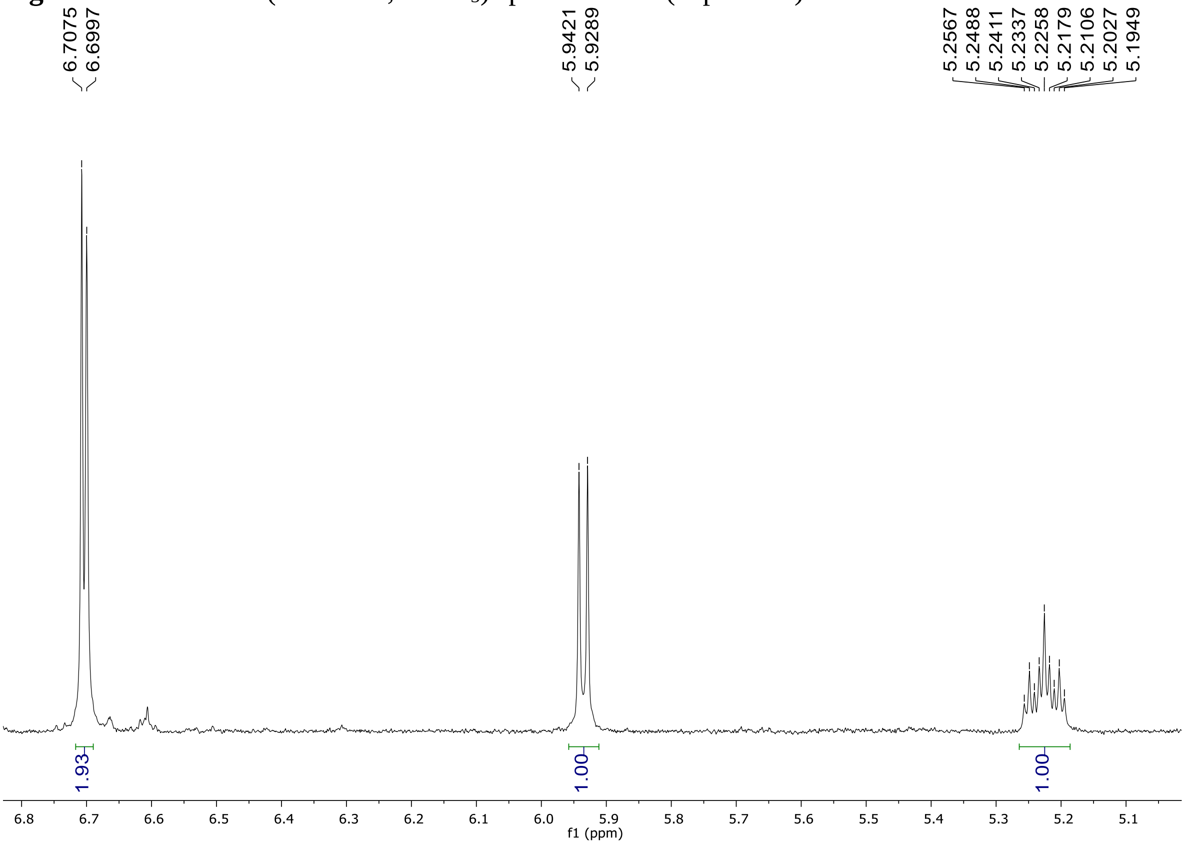

Figure S97. ^{1}H NMR (500 MHz, $CDCl_3$) spectrum of **7** (expansion).

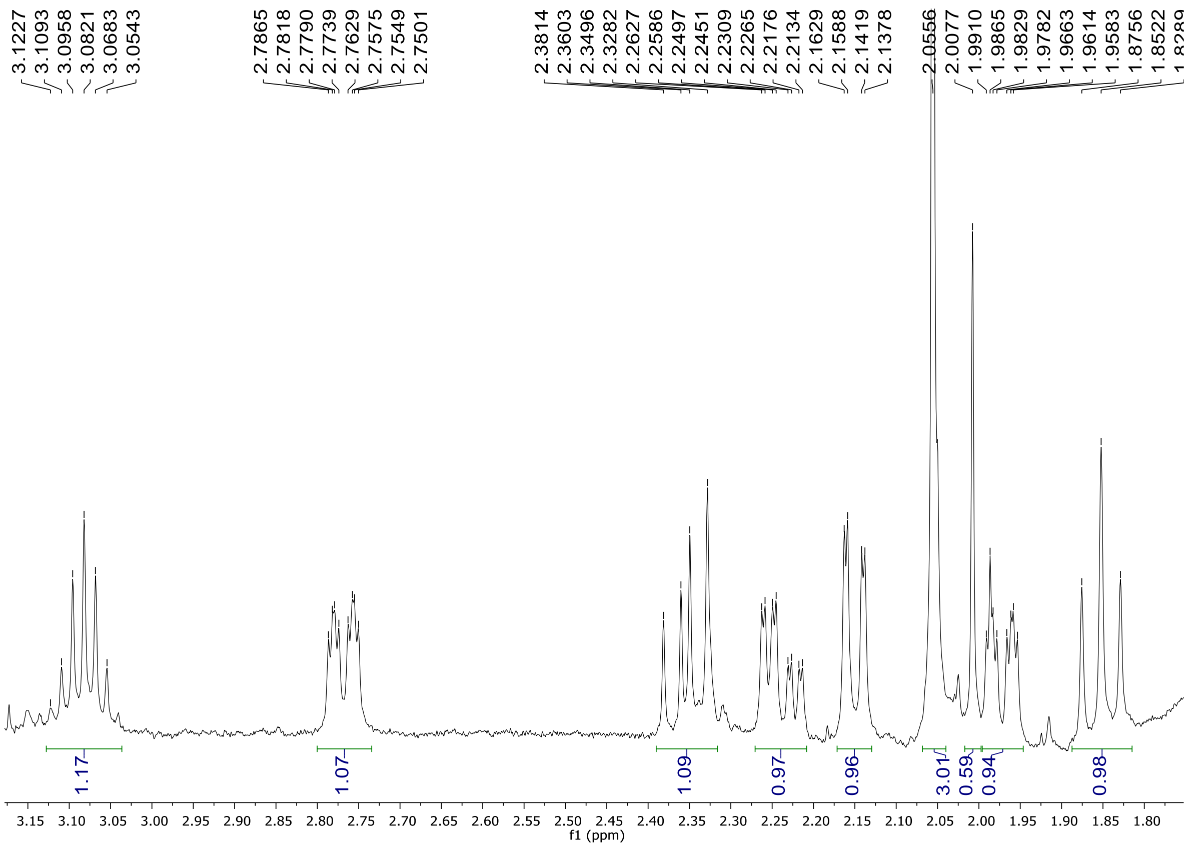

Figure S98. ^{1}H NMR (500 MHz, $CDCl_3$) spectrum of **7** (expansion).

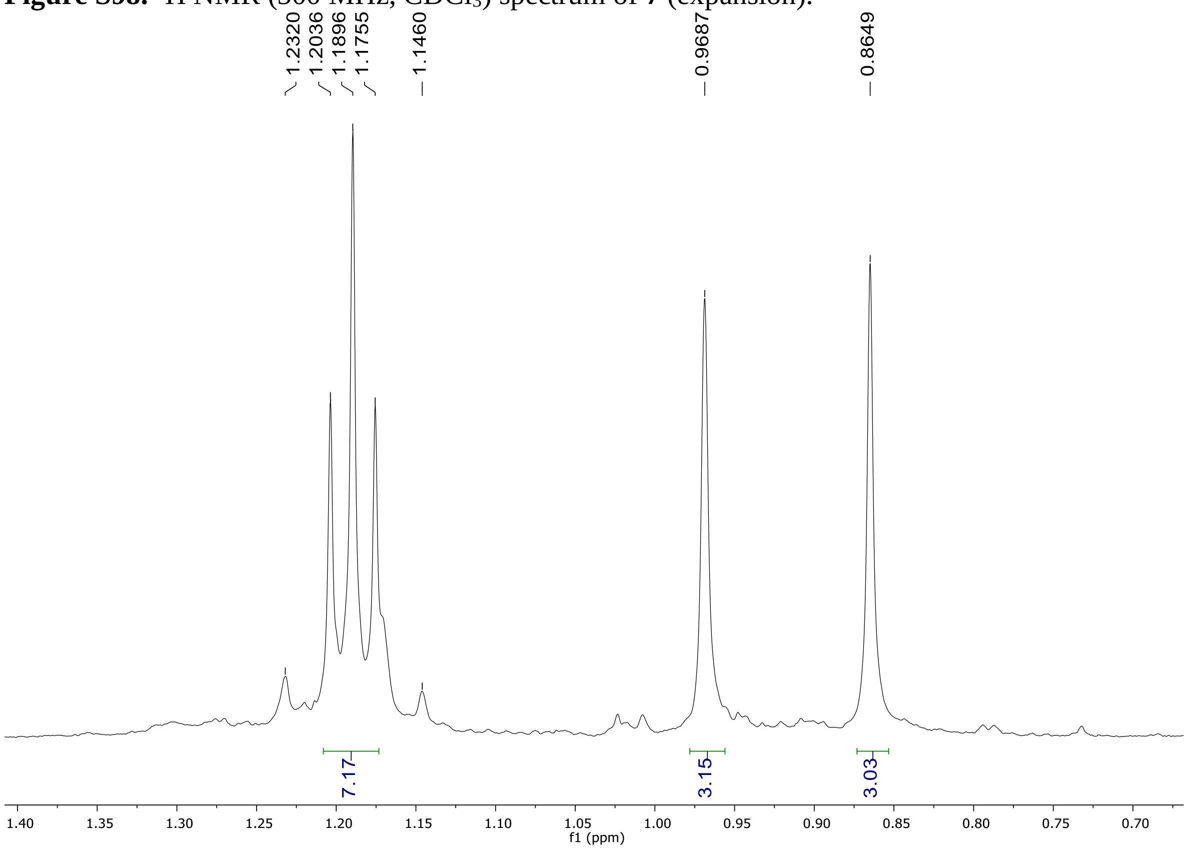

Figure S99. ^{13}C NMR - BB (125 MHz, $CDCl_3$) spectrum of **7**

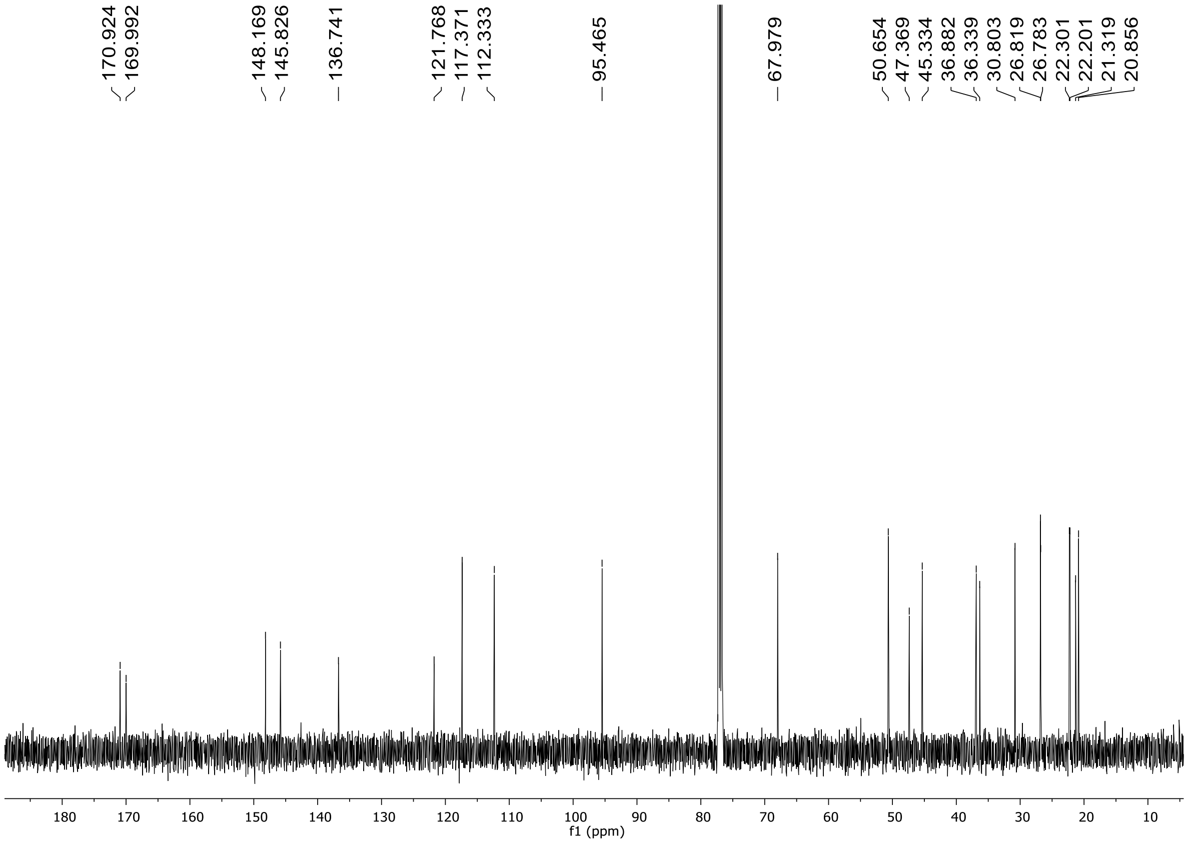

Figure S100. ^{13}C NMR - BB (125 MHz, $CDCl_3$) spectrum of **7** (expansion).

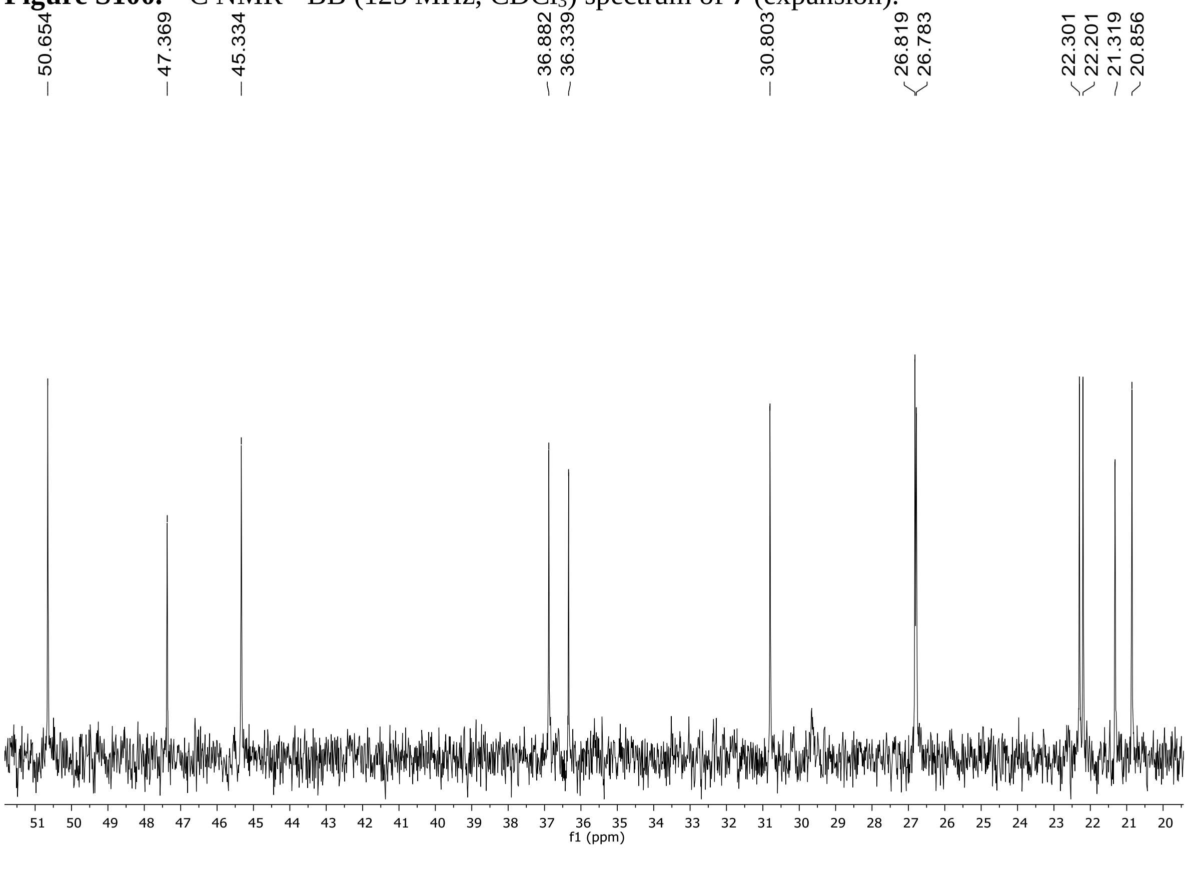

Figure S101. ^{13}C NMR – DEPT 135 (125 MHz, $CDCl_3$) spectrum of **7**.

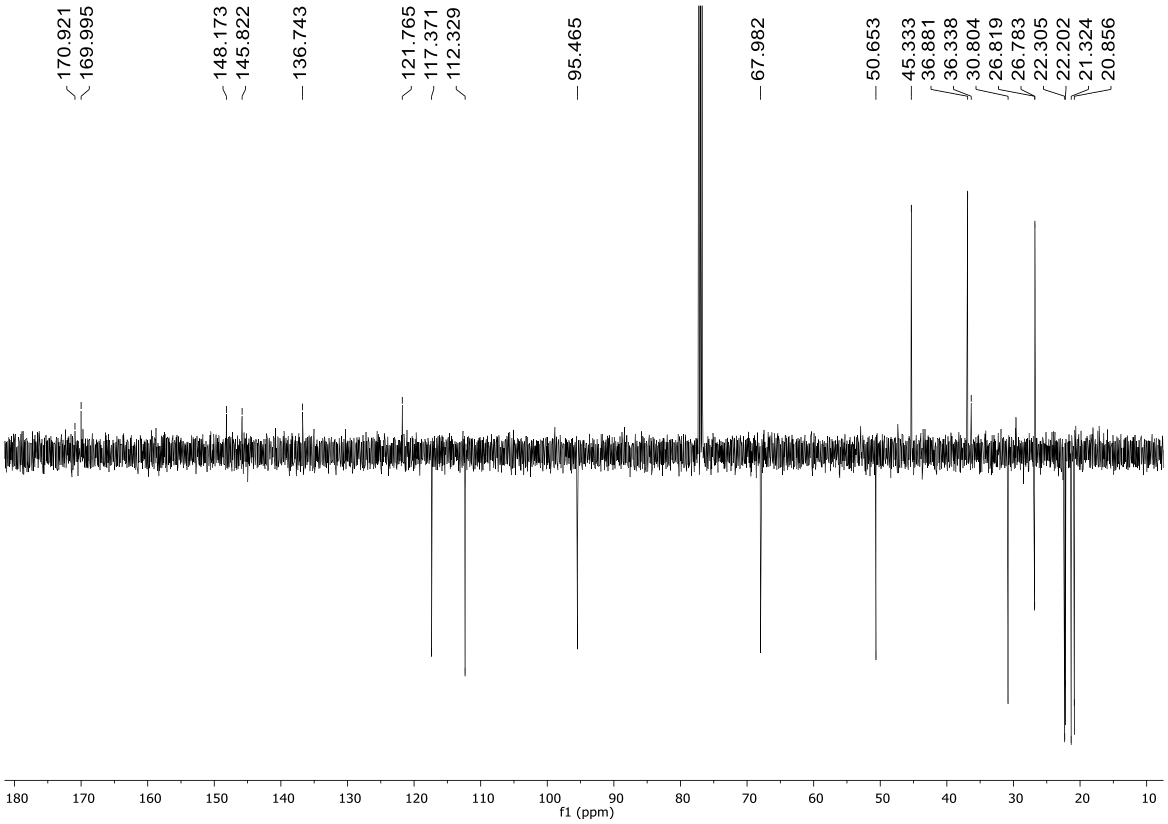

Figure S102. ^{13}C NMR – DEPT 135 (125 MHz, $CDCl_3$) spectrum of **7** (expansion)

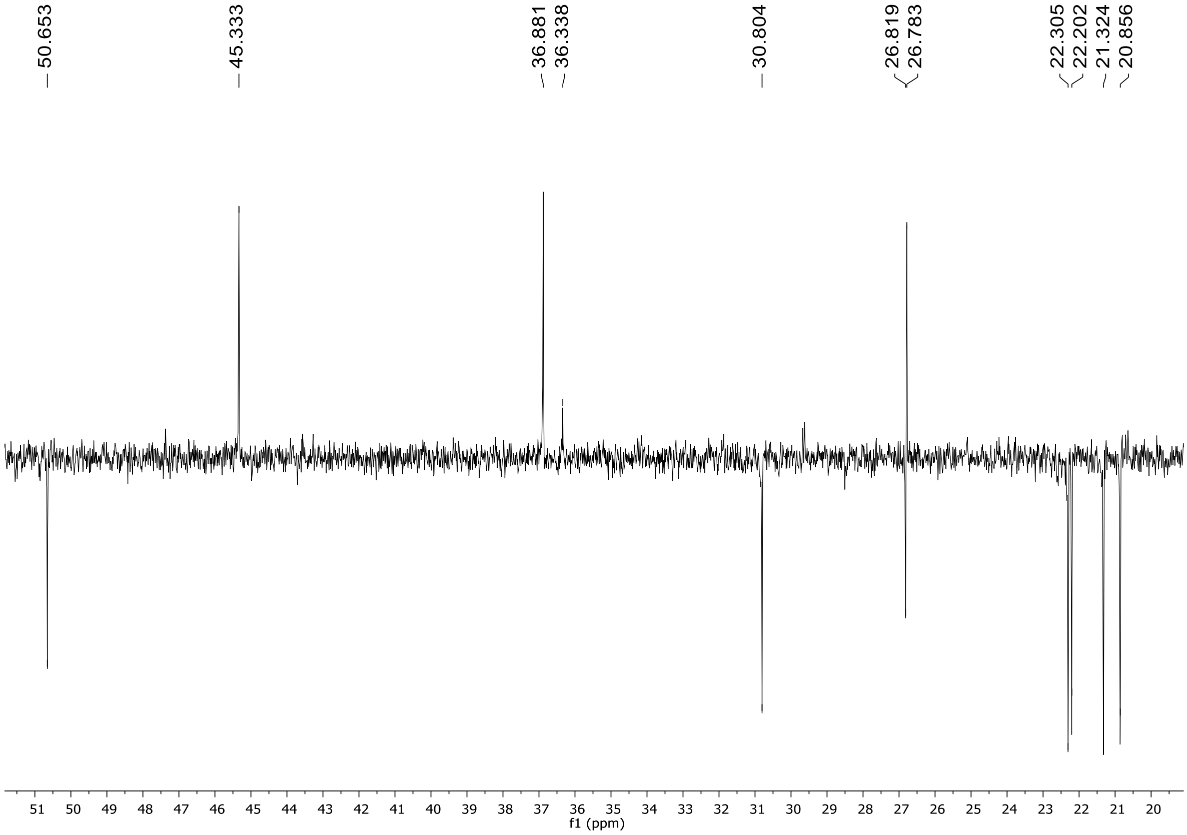

Figure S103. HSQC NMR (500 X 125 MHz, $CDCl_3$) spectrum of **7**

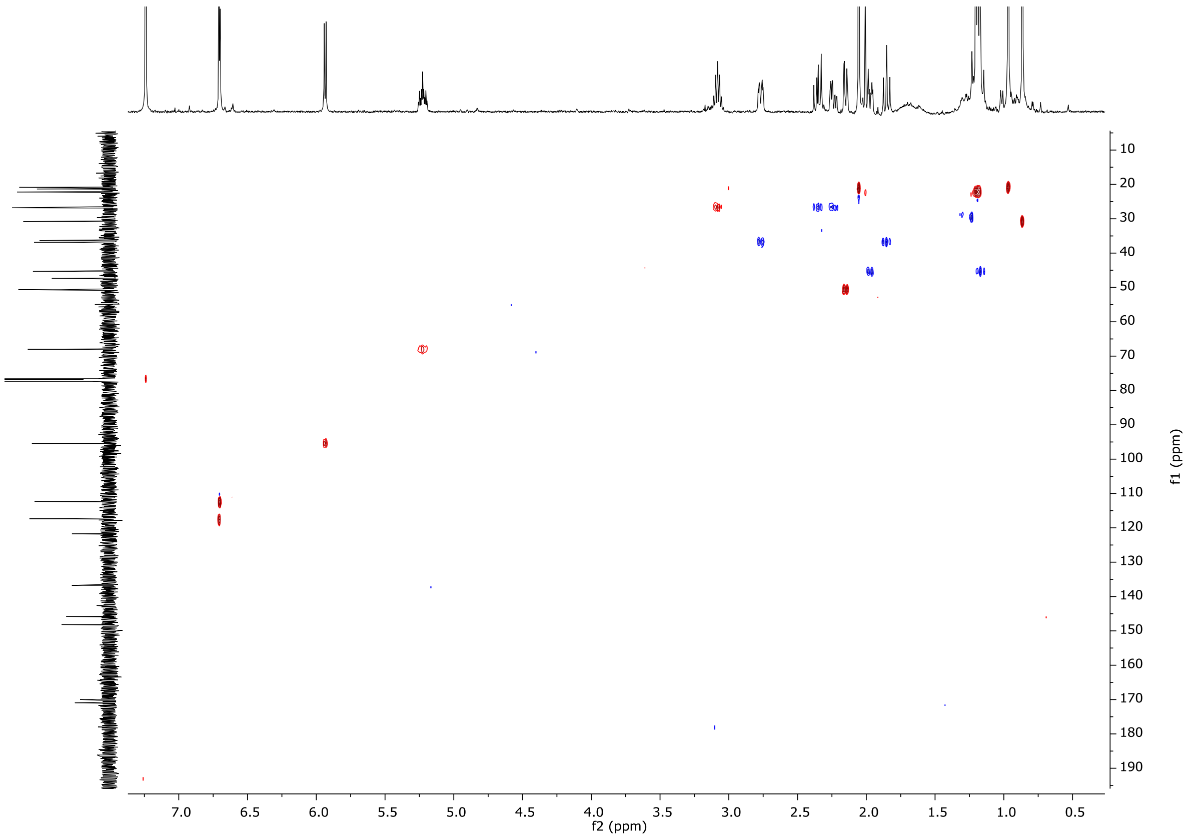

Figure S104. HMBC NMR (500 X 125 MHz, $CDCl_3$) spectrum of **7**

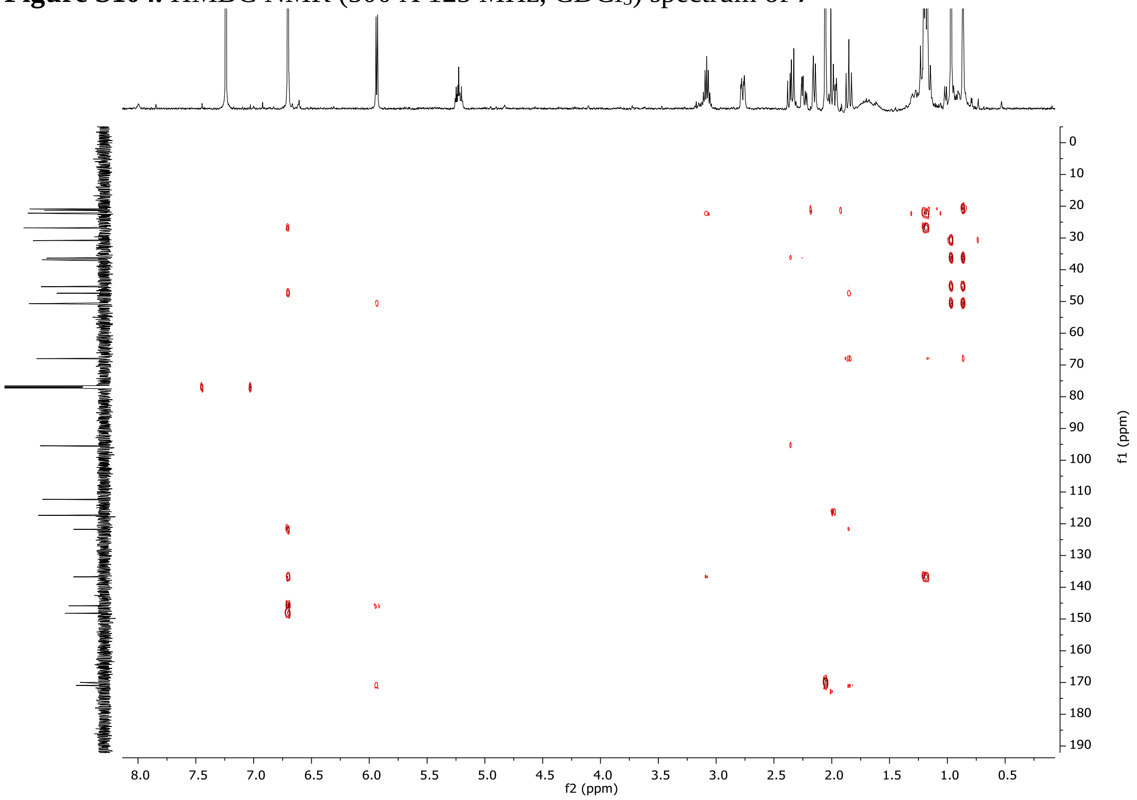

Figure S105. HMBC NMR (500 X 125 MHz, $CDCl_3$) spectrum of **7** (expansion).

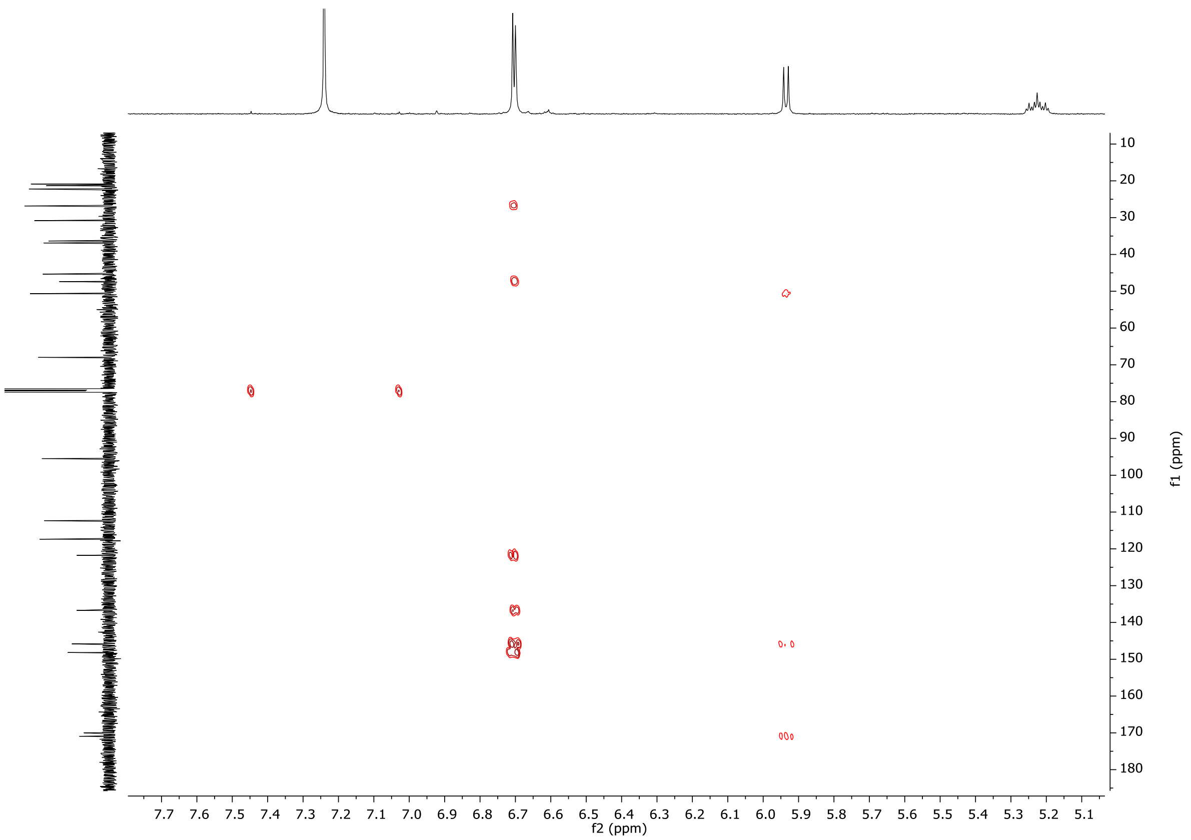

Figure S106. HMBC NMR (500 X 125 MHz, $CDCl_3$) spectrum of **7** (expansion).

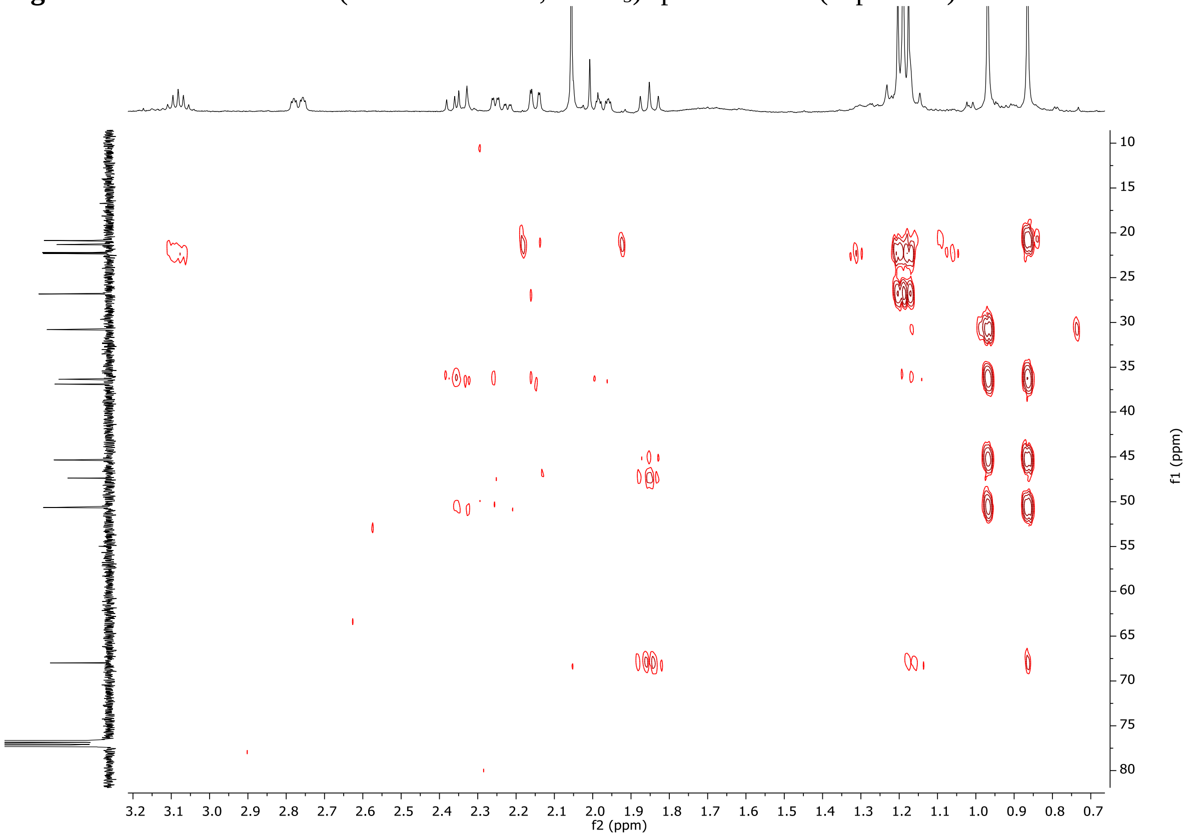

Figure S107. HMBC NMR (500 X 125 MHz, $CDCl_3$) spectrum of **7** (expansion).

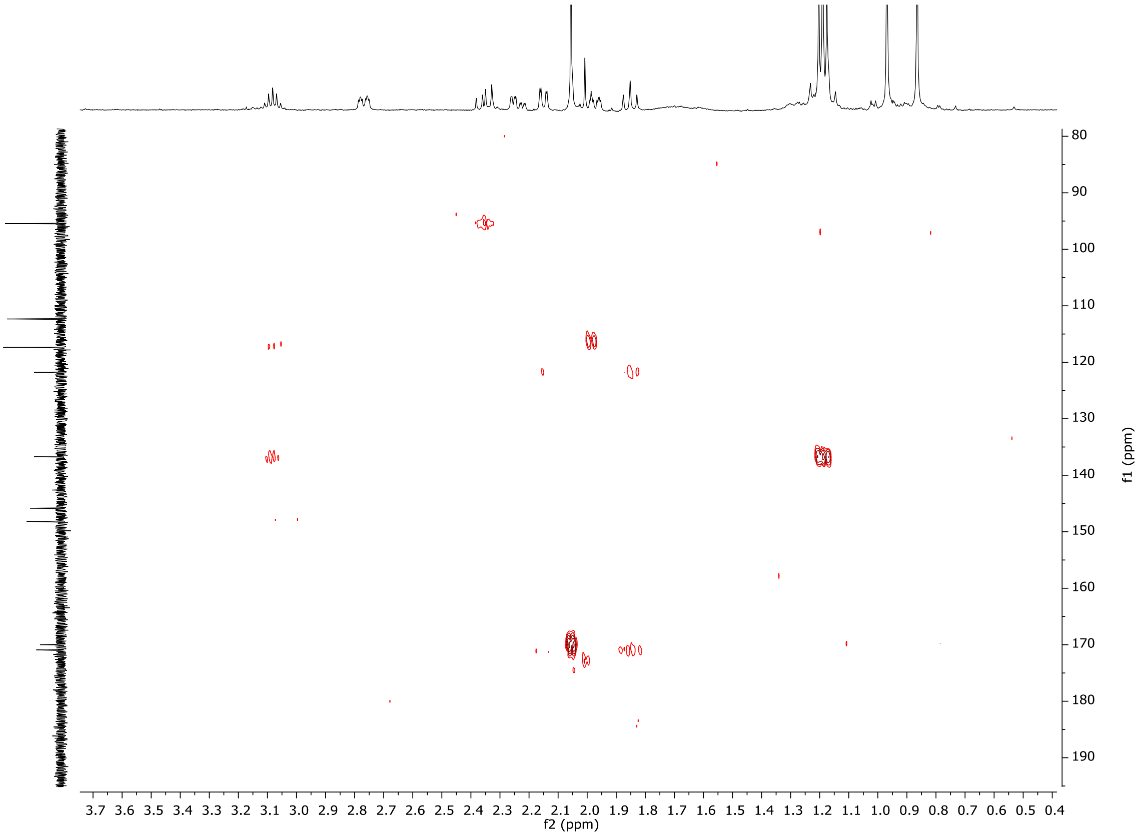

Figure S108. COSY NMR (500 MHz, $CDCl_3$) spectrum of **7**

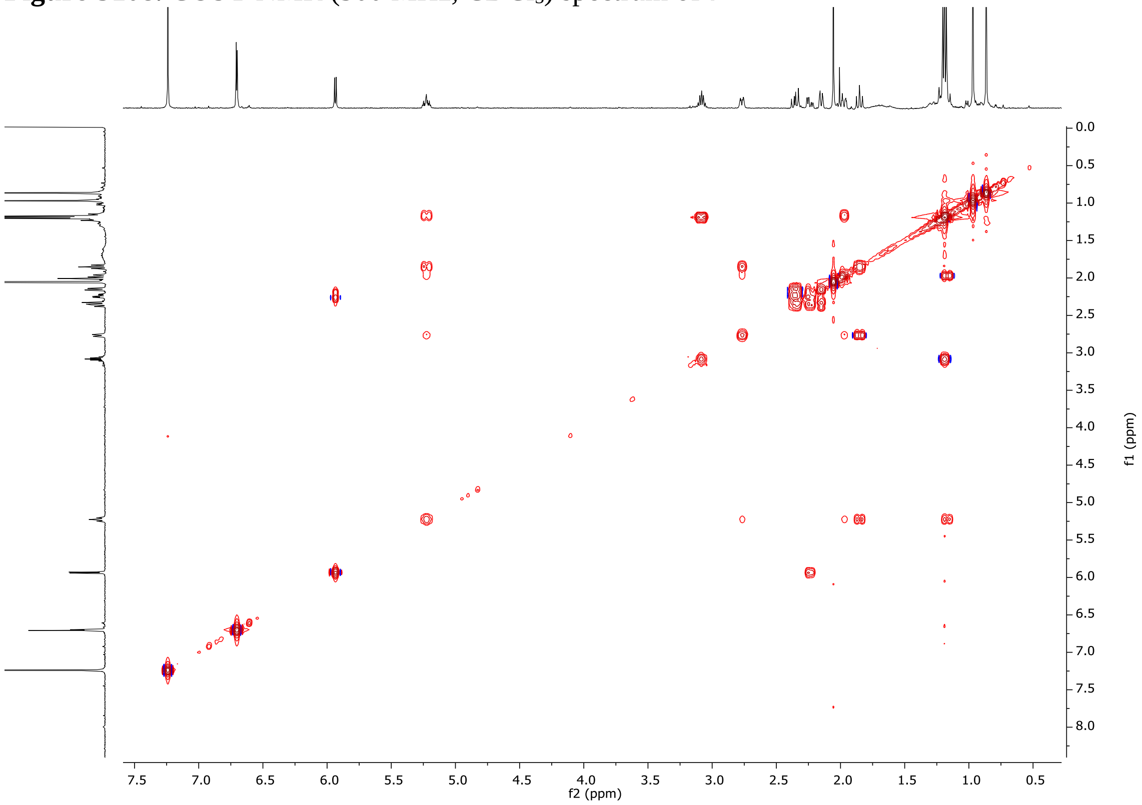

Figure S109. COSY NMR (500 MHz, $CDCl_3$) spectrum of **7** (expansion).

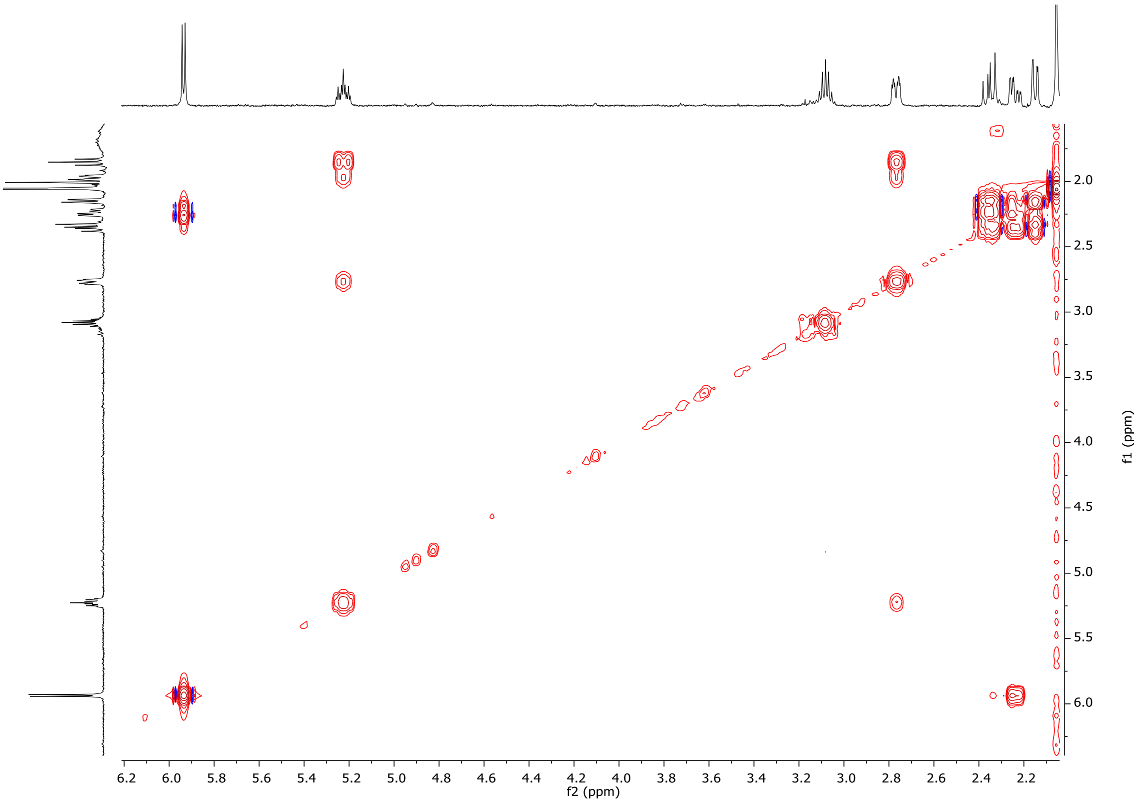

Figure S110. COSY NMR (500 MHz, $CDCl_3$) spectrum of **7** (expansion).

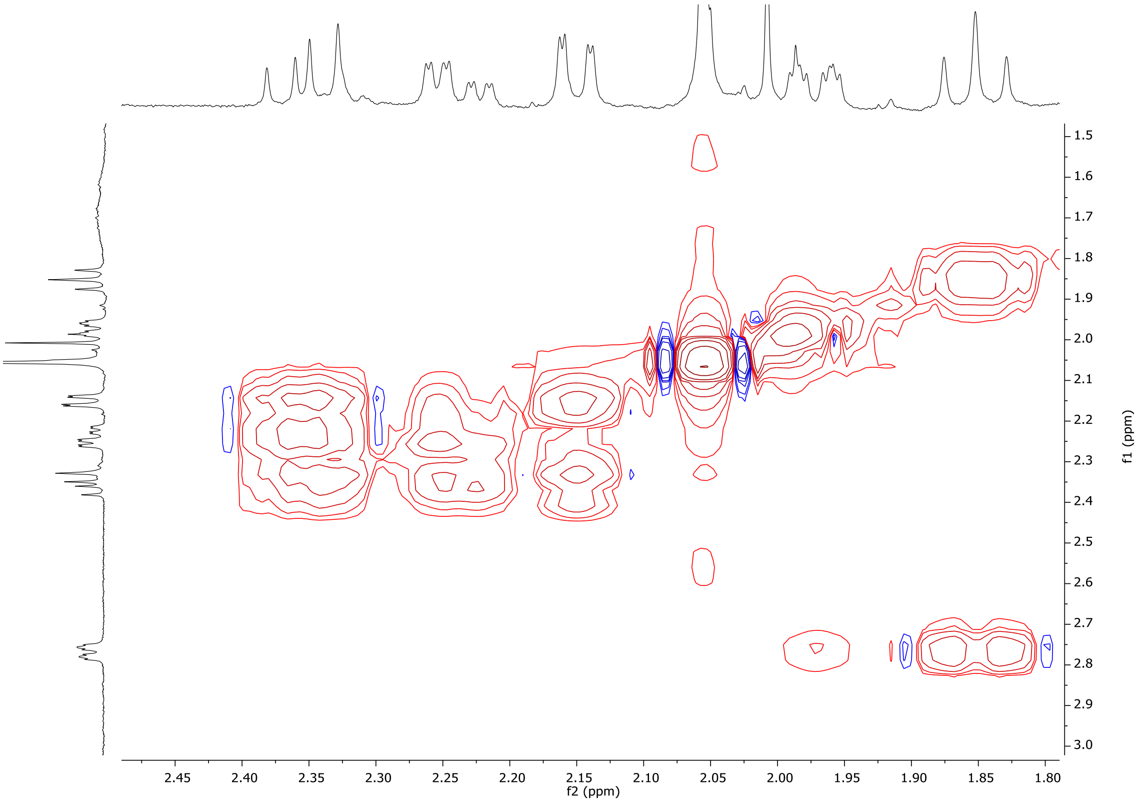

Figure S111. NOESY NMR (500 MHz, $CDCl_3$) spectrum of **7.**

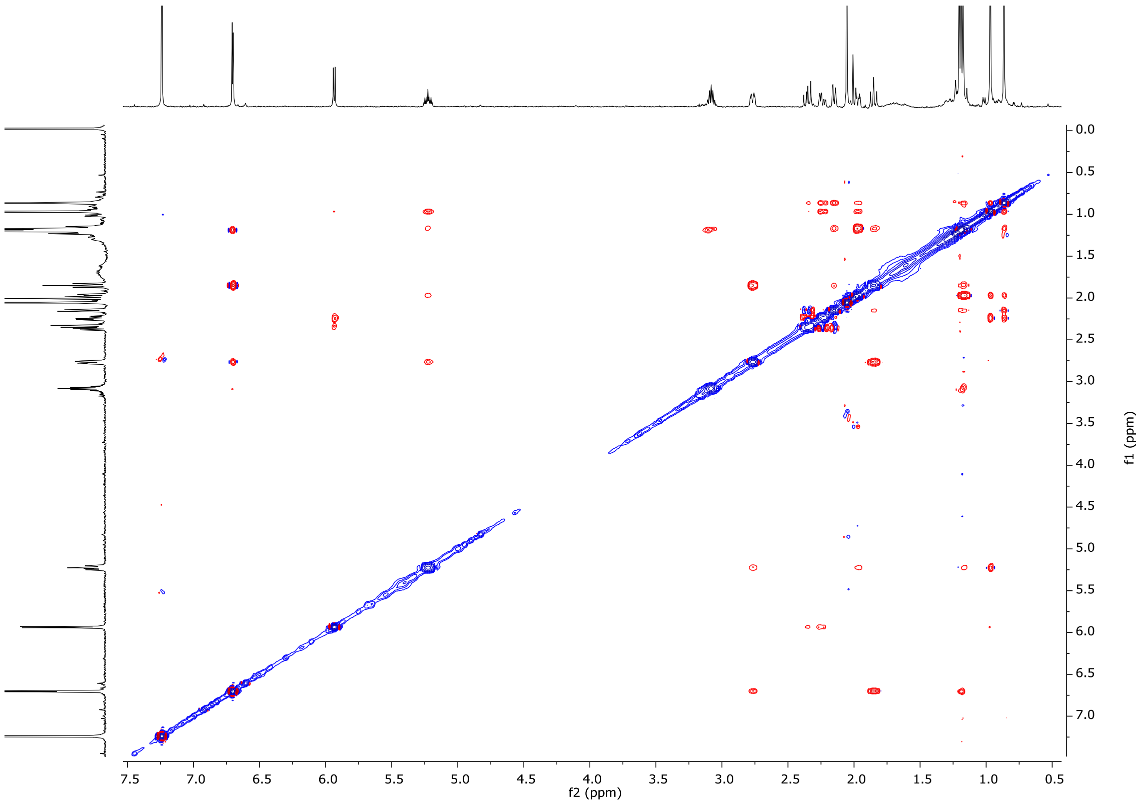

Figure S112. NOESY NMR (500 MHz, $CDCl_3$) spectrum of **7** (expansion).

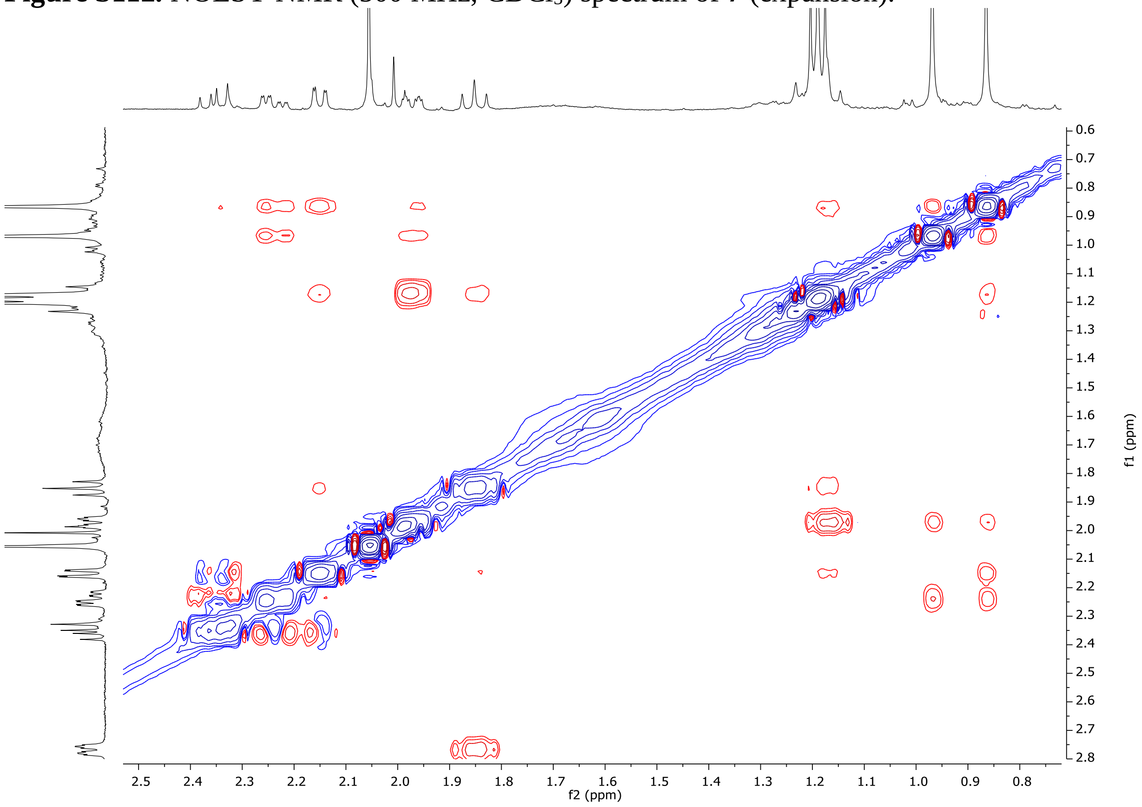

Figure S113. NOESY NMR (500 MHz, $CDCl_3$) spectrum of **7** (expansion).

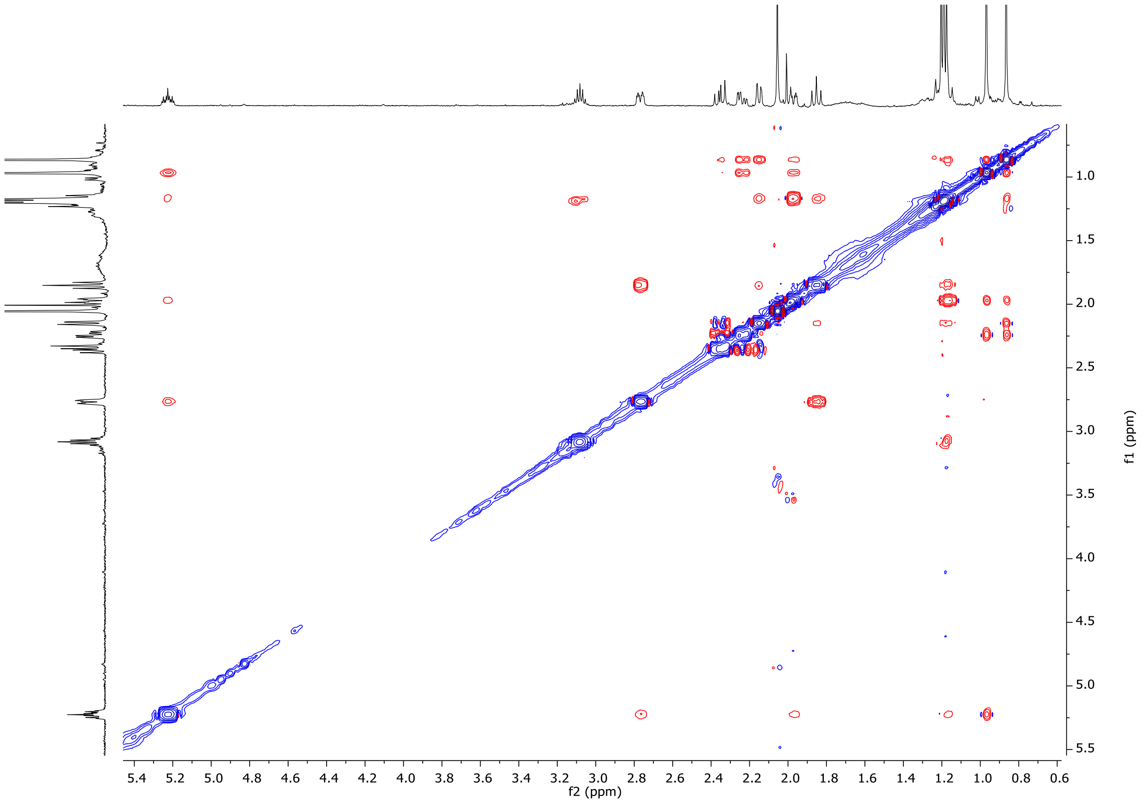

Figure S114. IR spectrum of compound **8.**

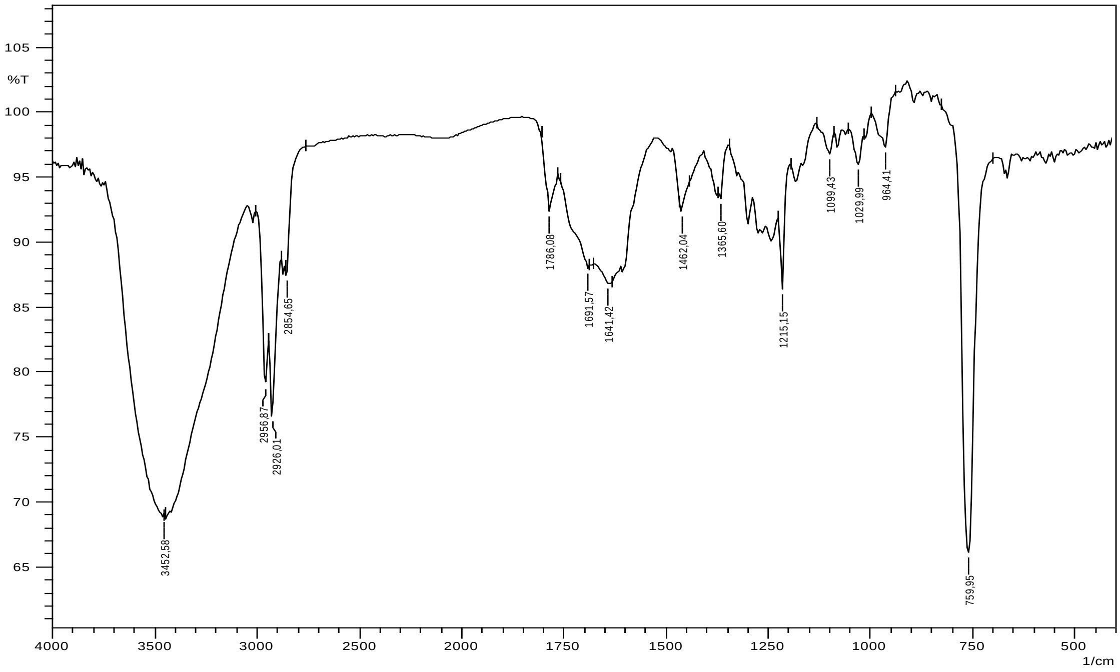

Figure S115. HRESIMS Spectrum of **8** ([M + Na]$^{+}$ positive ion mode).

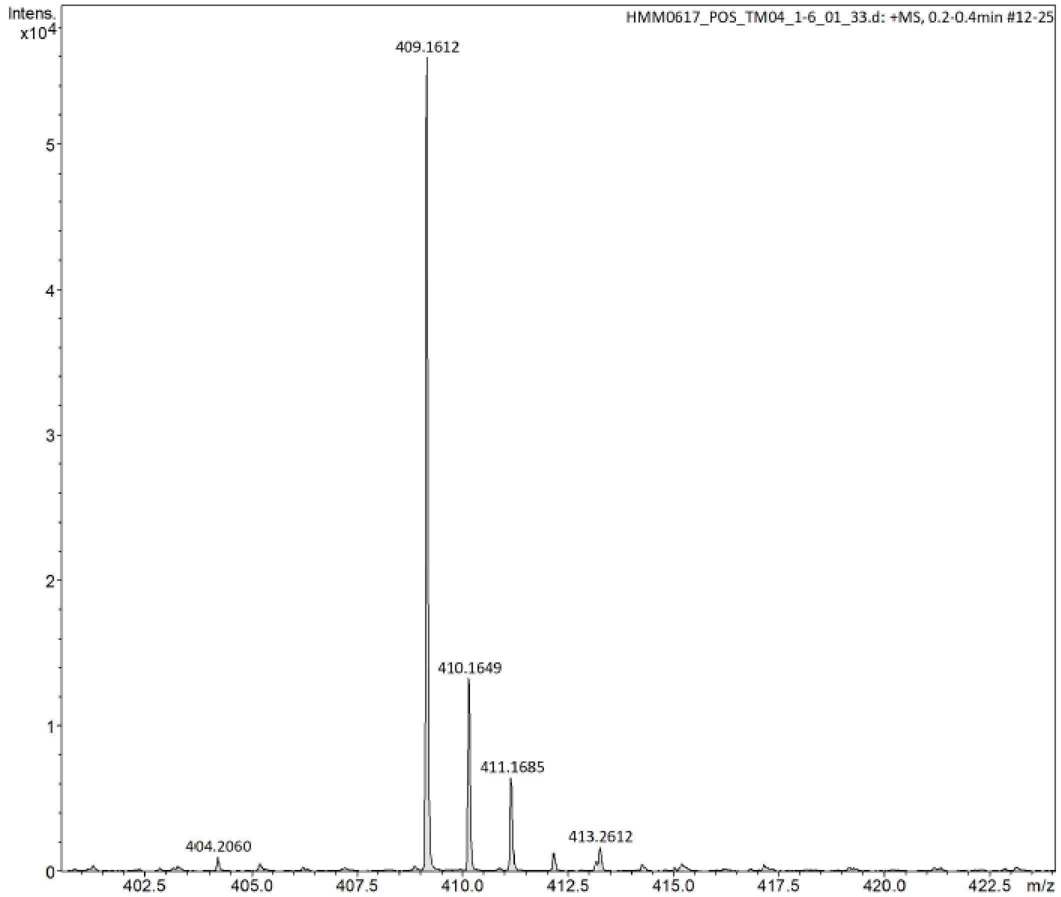

Figure S116. ^{1}H NMR (500 MHz, $CDCl_3$) spectrum of **8.**

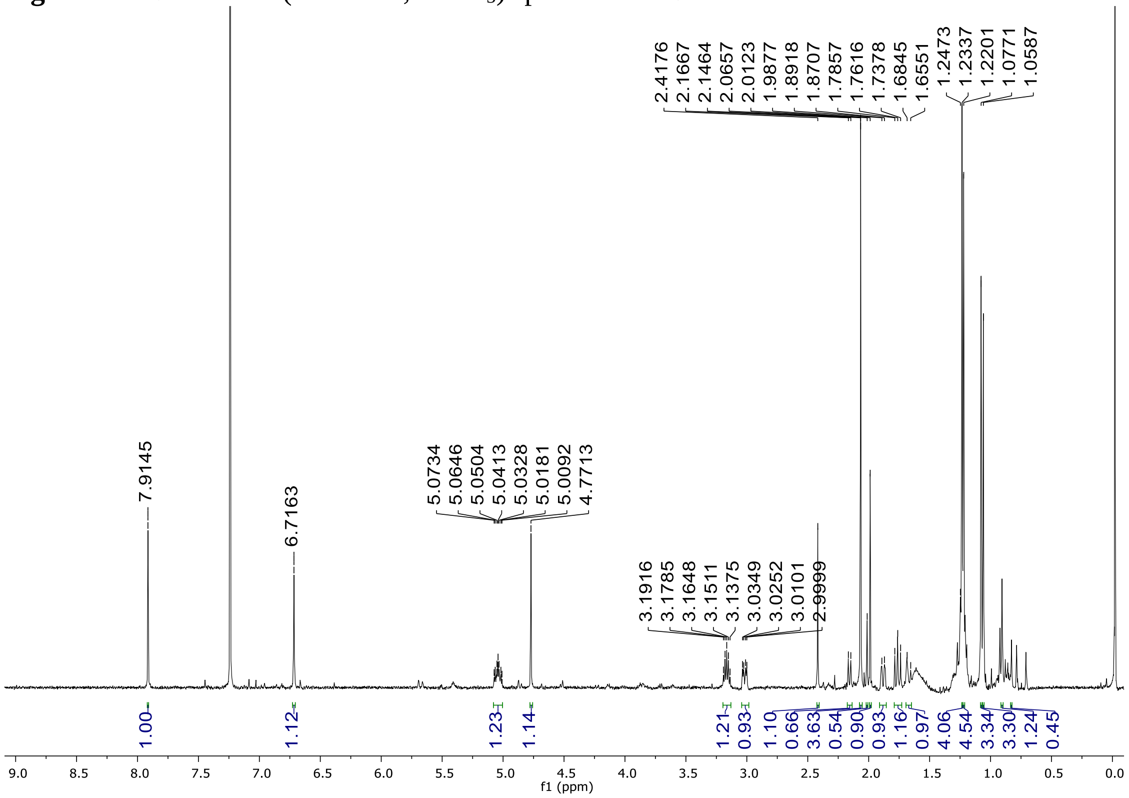

Figure S117. ^{1}H NMR (500 MHz, $CDCl_3$) spectrum of **8** (expansion).

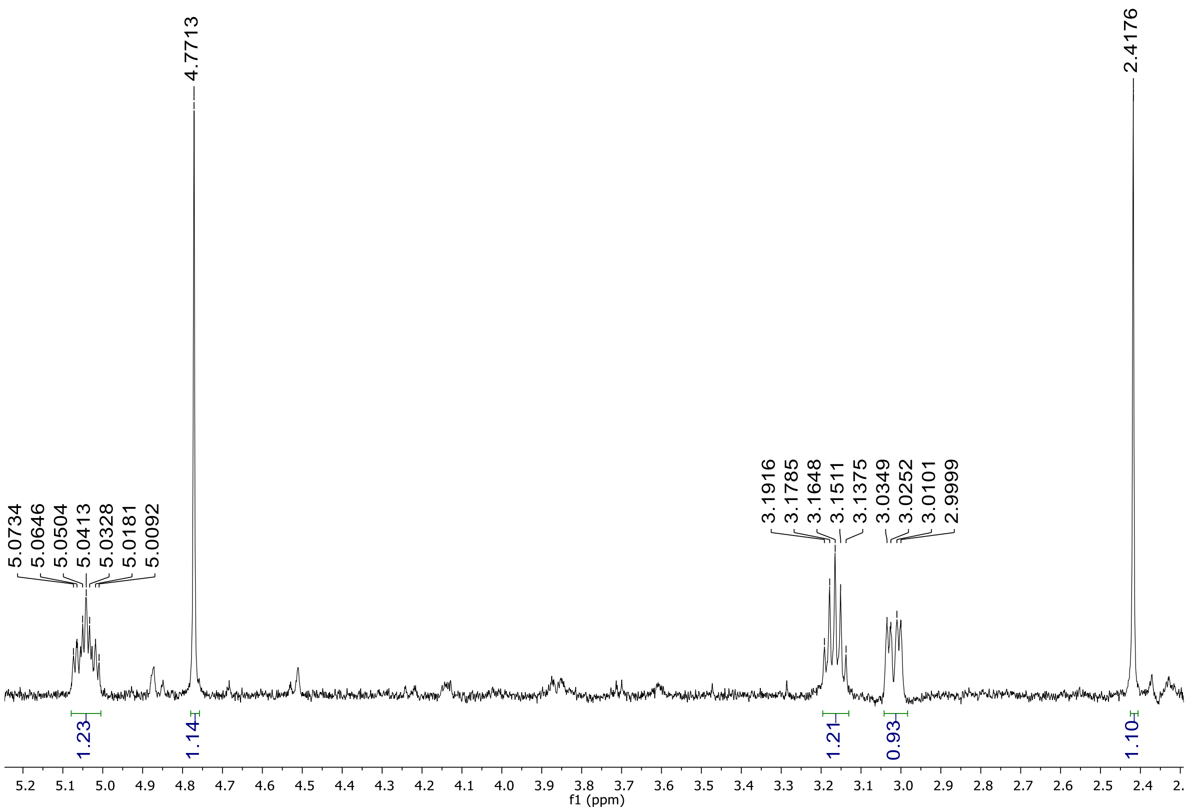

Figure S118. ^{1}H NMR (500 MHz, $CDCl_3$) spectrum of **8** (expansion).

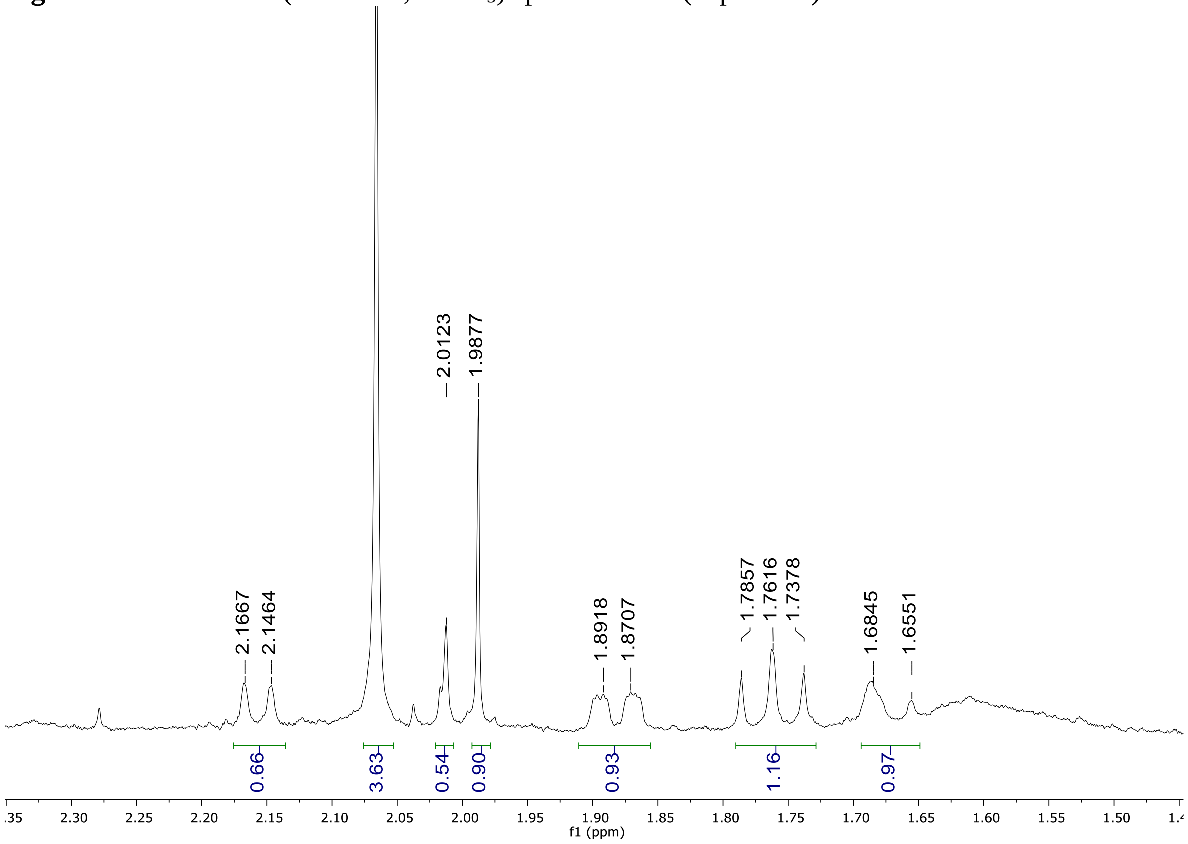

Figure S119. ^{1}H NMR (500 MHz, $CDCl_3$) spectrum of **8** (expansion).

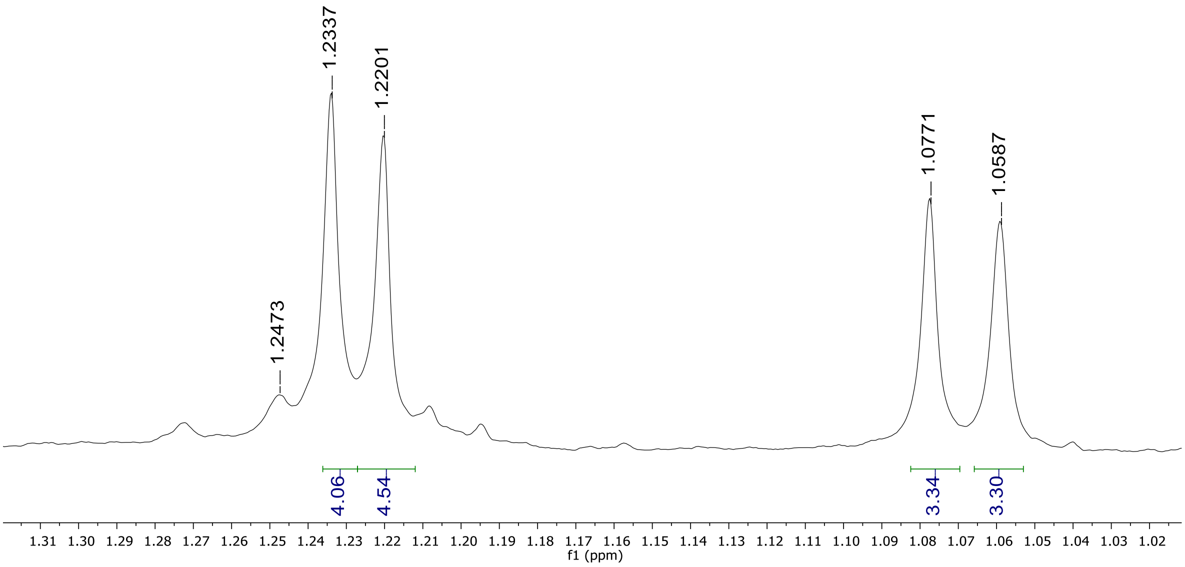

Figure S120. ^{13}C NMR - BB (125 MHz, $CDCl_3$) spectrum of **8.**

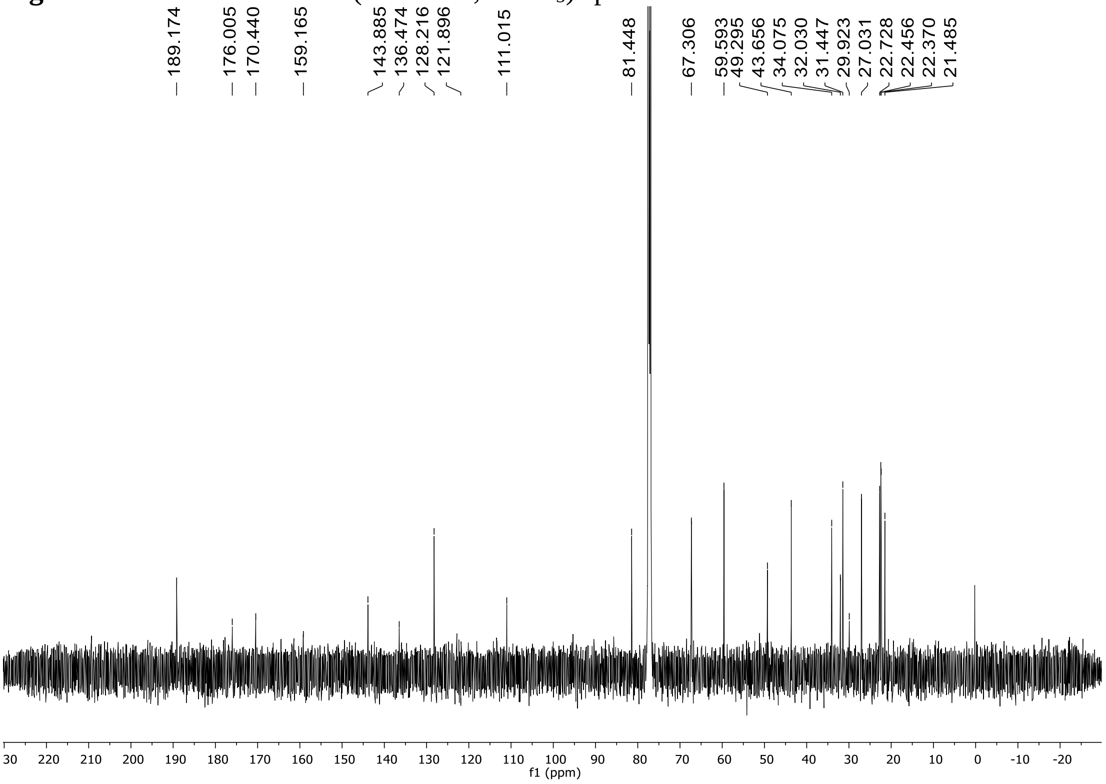

Figure S121. ^{13}C NMR – DEPT 135 (125 MHz, $CDCl_3$) spectrum of **8**.

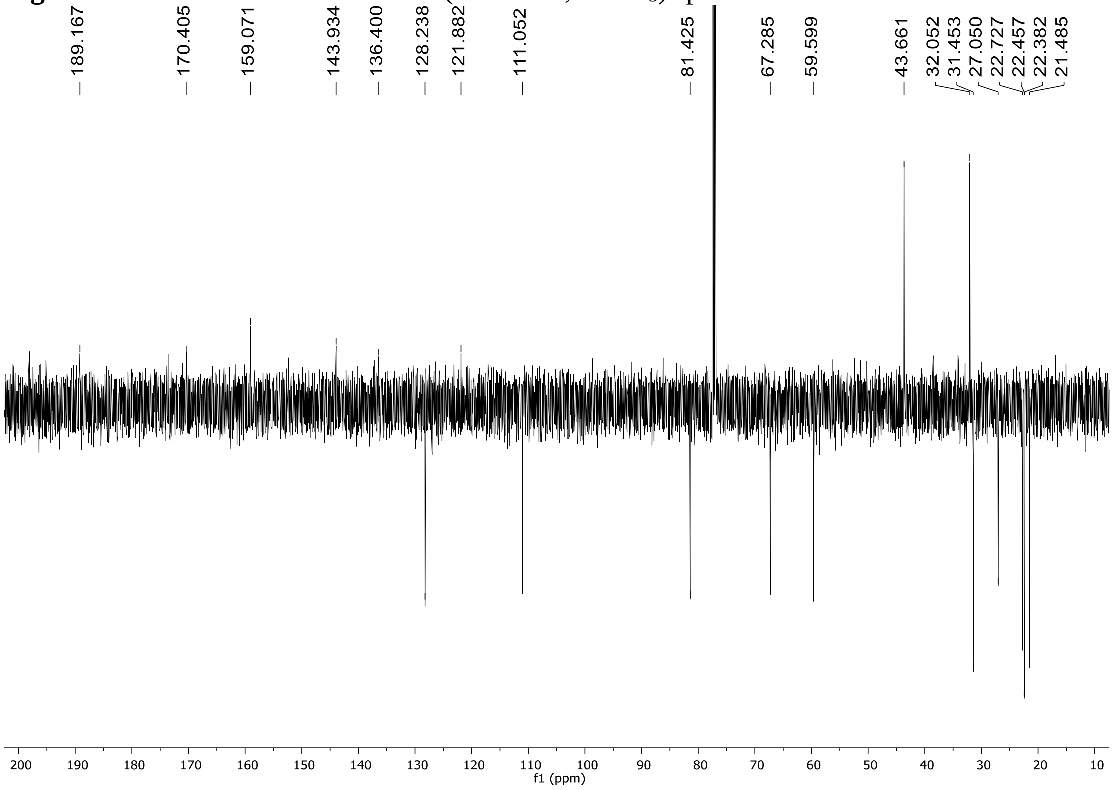

Figure S122. ^{13}C NMR – DEPT 135 (125 MHz, $CDCl_3$) spectrum of **8** (expansion).

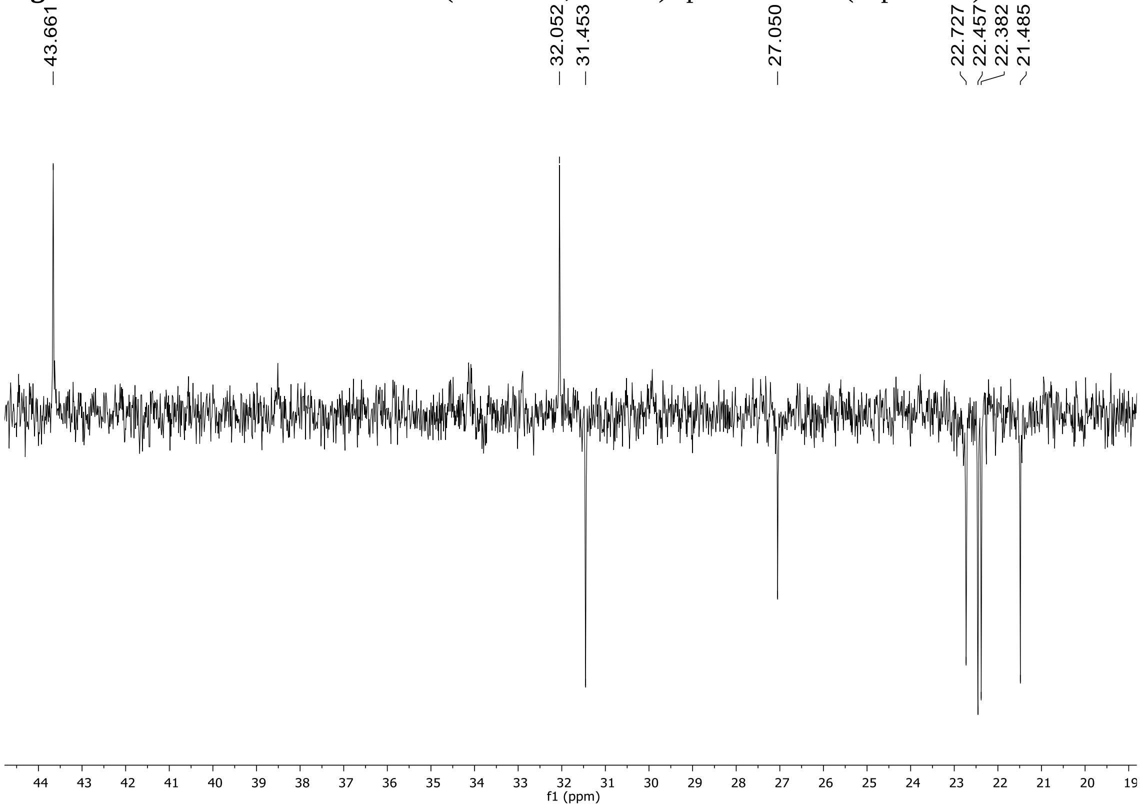

Figure S123. HSQC NMR (500 X 125 MHz, CDCl$_3$) spectrum of **8**

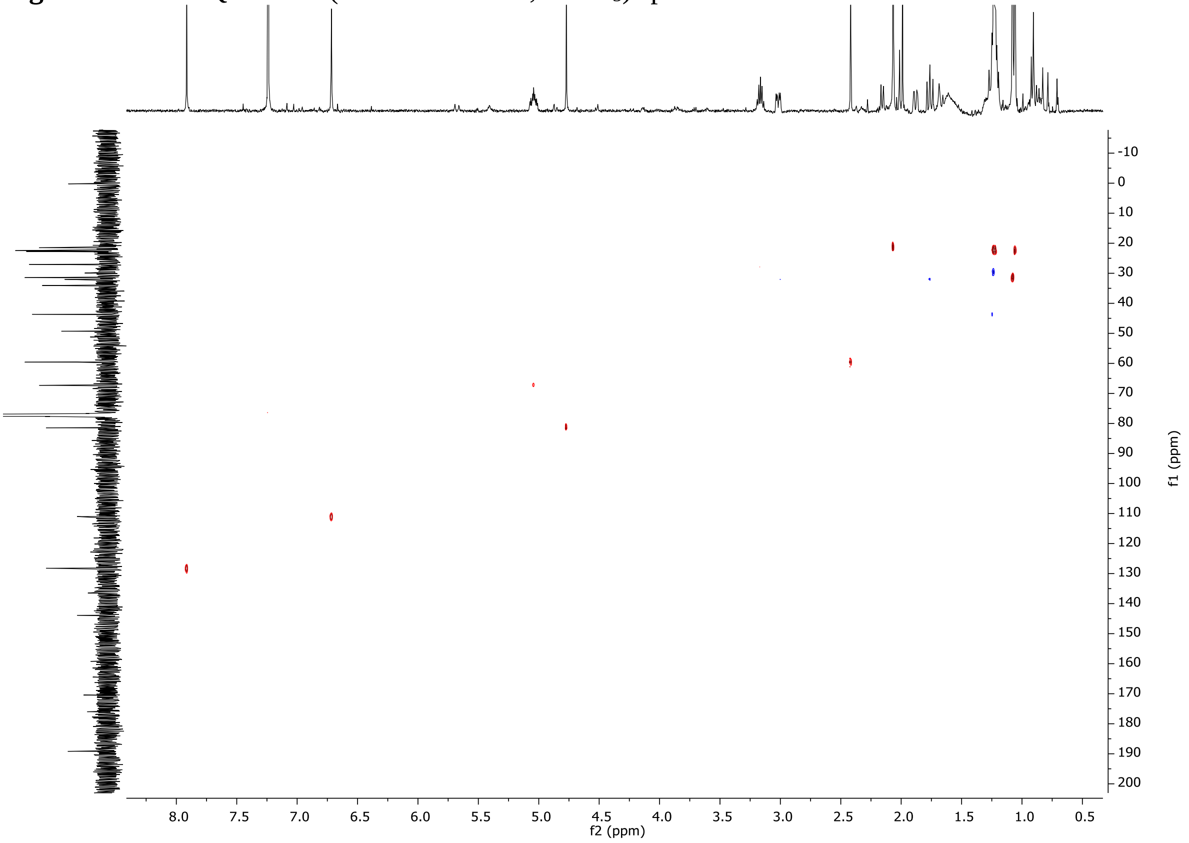

Figure S124. HSQC NMR (500 X 125 MHz, CDCl$_3$) spectrum of **8** (expansion).

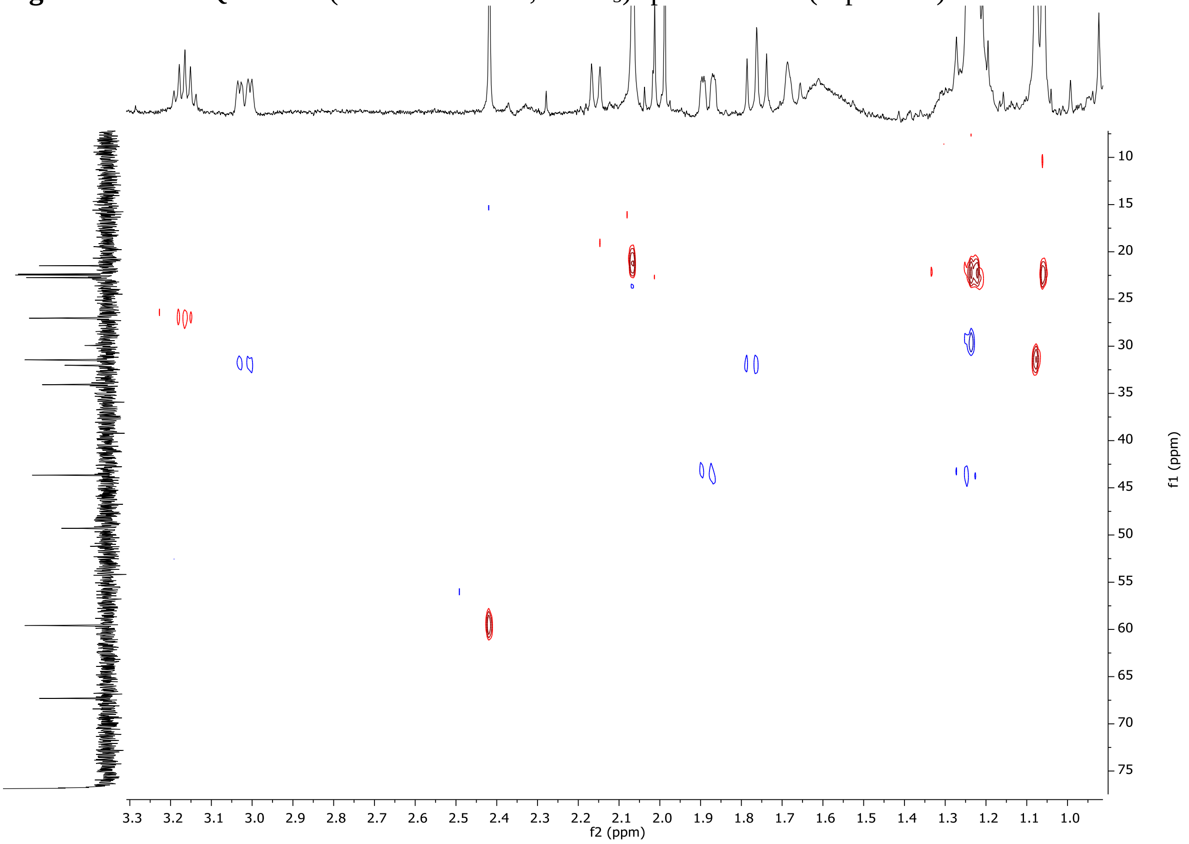

Figure S125. HMBC NMR (500 X 125 MHz, $CDCl_3$) spectrum of **8**

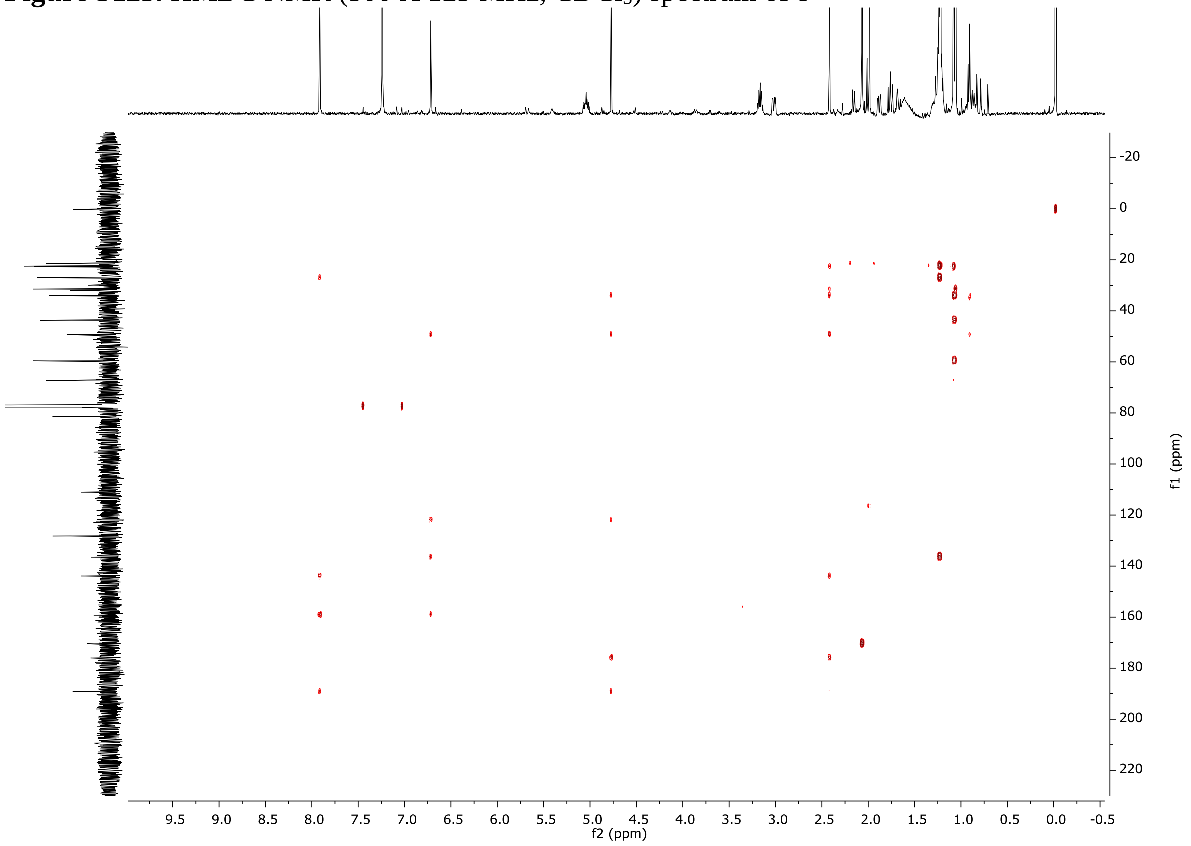

Figure S126. HMBC NMR (500 X 125 MHz, $CDCl_3$) spectrum of **8** (expansion).

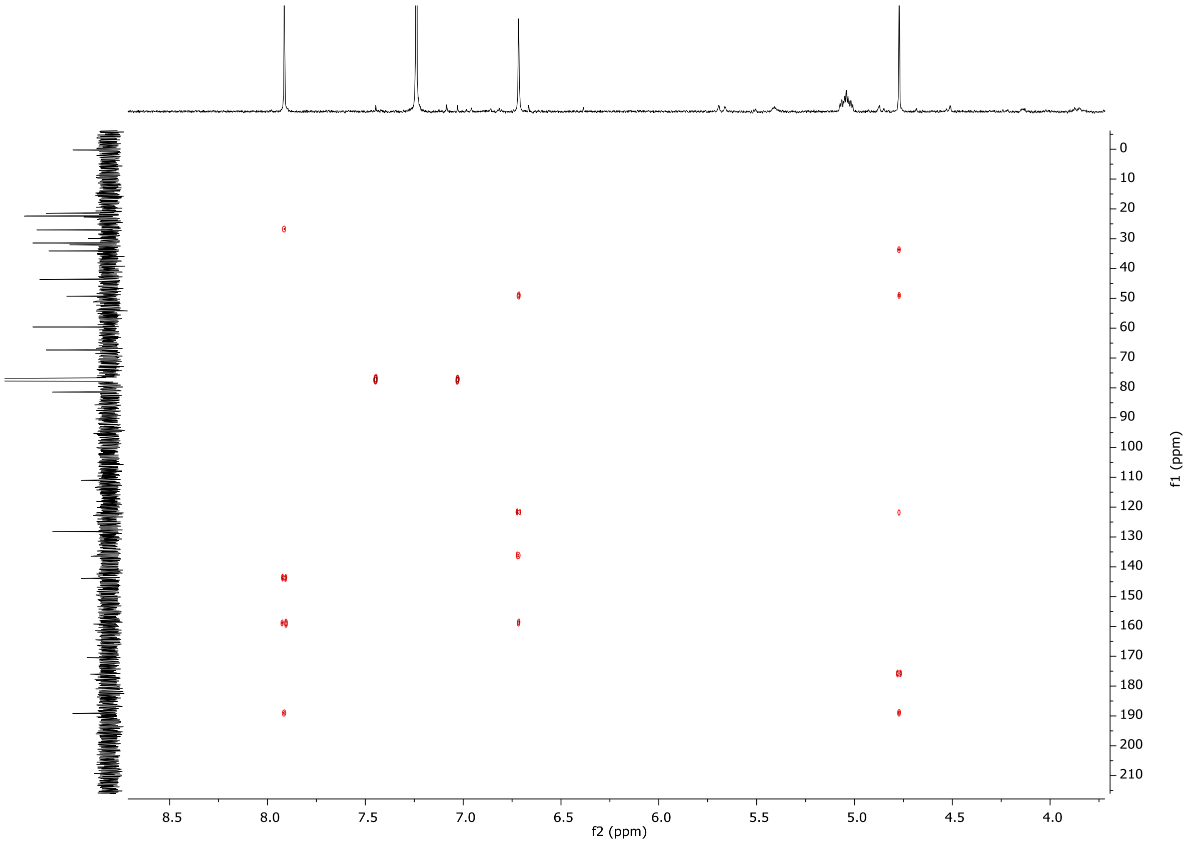

Figure S127. HMBC NMR (500 X 125 MHz, $CDCl_3$) spectrum of **8** (expansion).

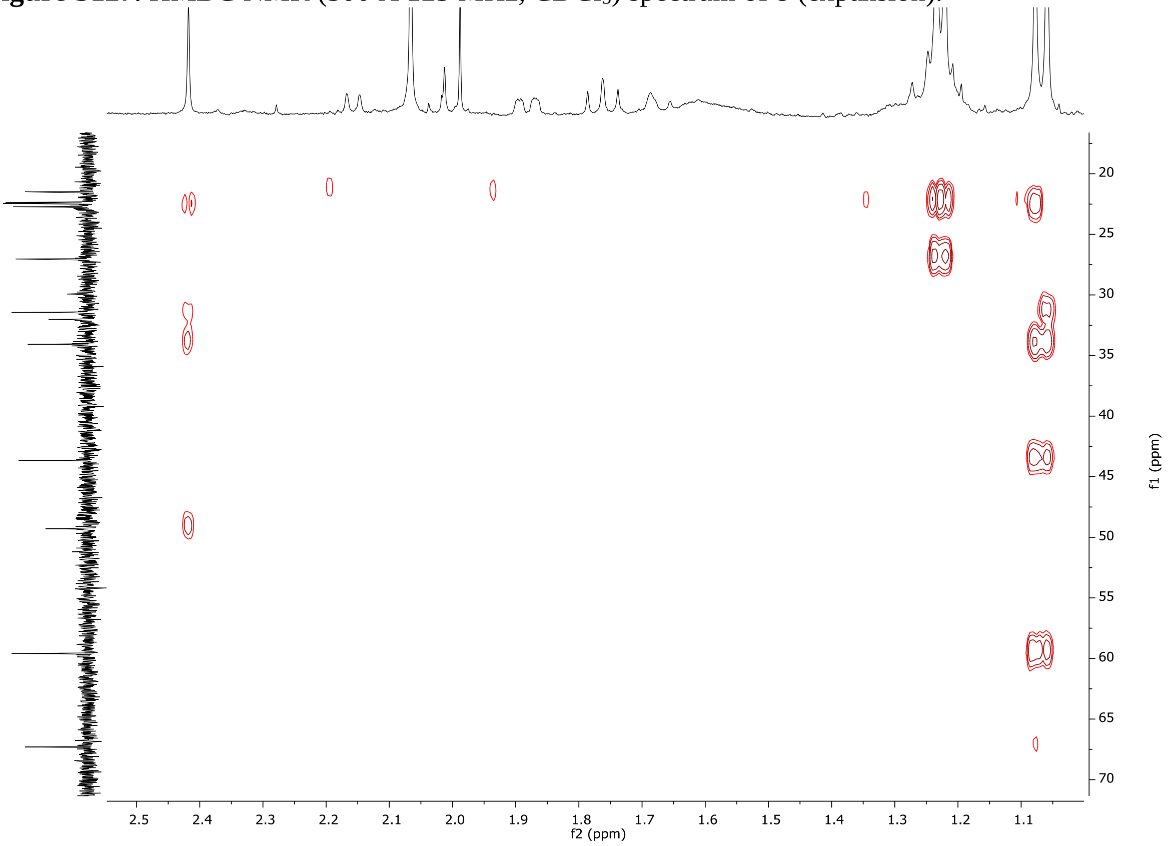

Figure S128. HMBC NMR (500 X 125 MHz, $CDCl_3$) spectrum of **8** (expansion).

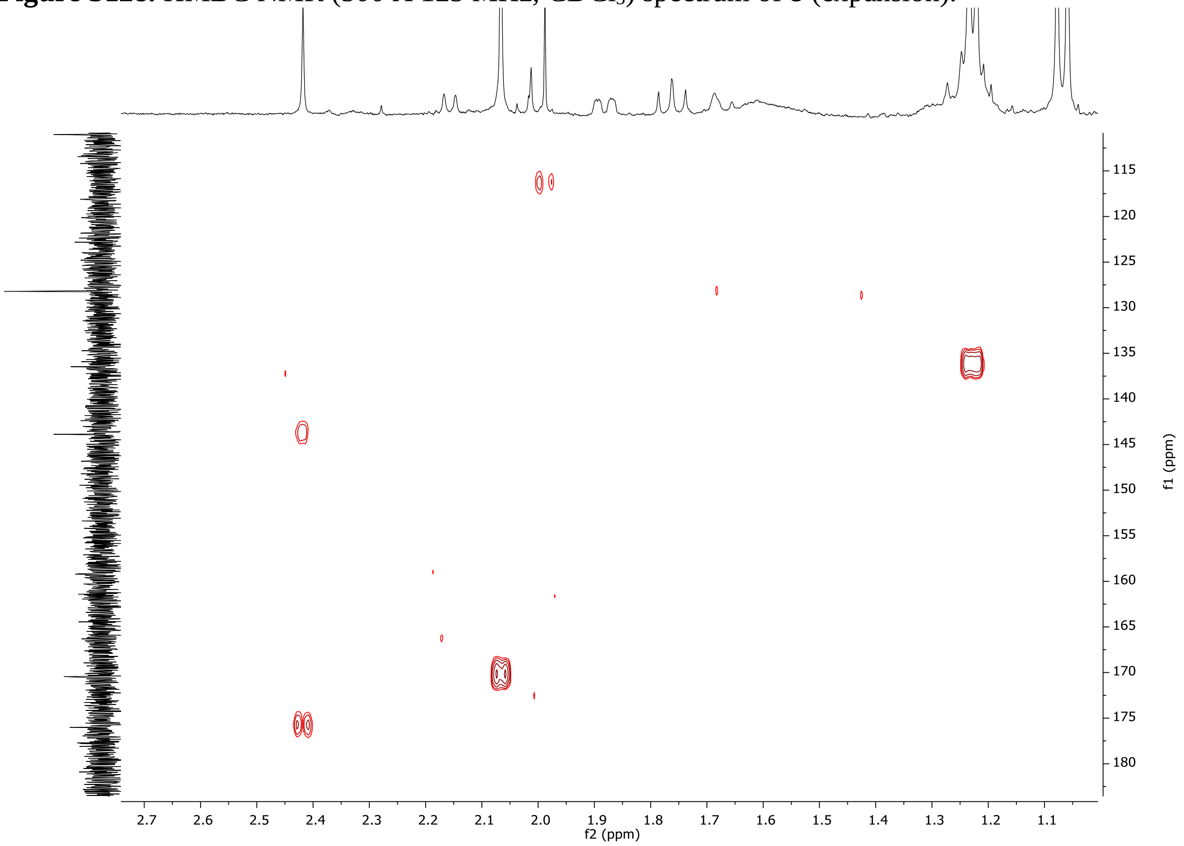

Figure S129. COSY NMR (500 MHz, $CDCl_3$) spectrum of **8**

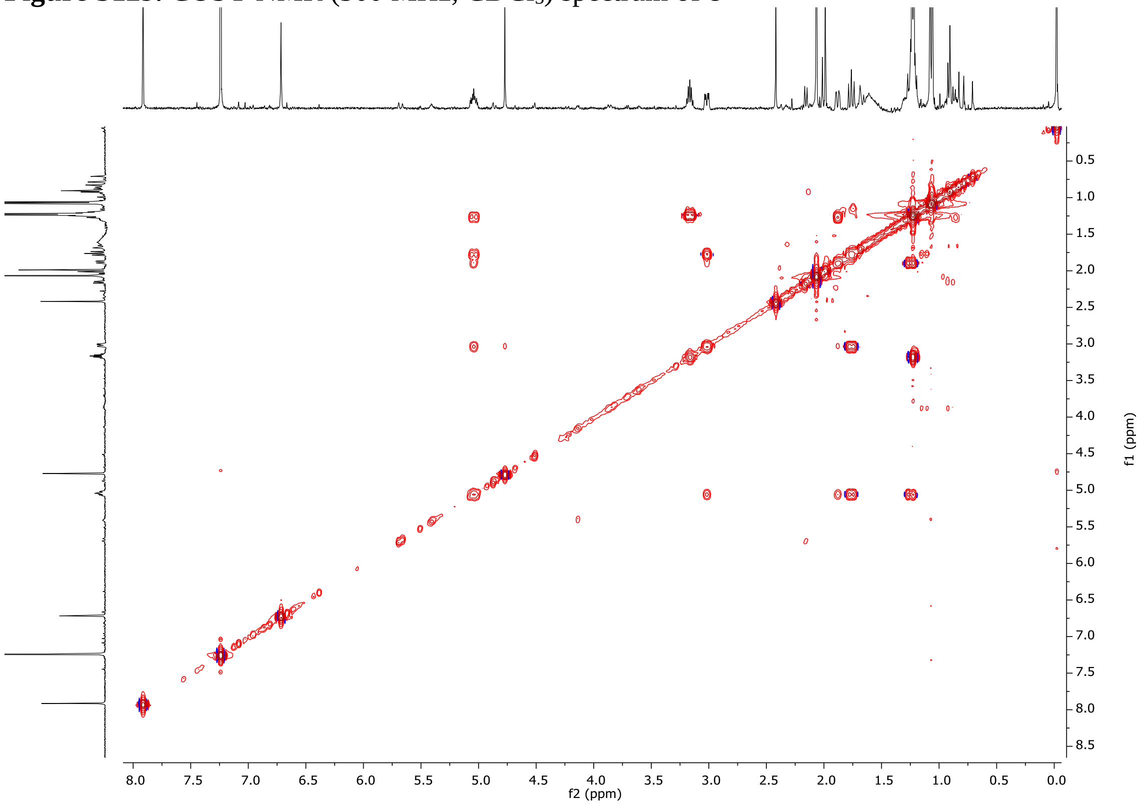

Figure S130. COSY NMR (500 MHz, $CDCl_3$) spectrum of **8** (expansion).

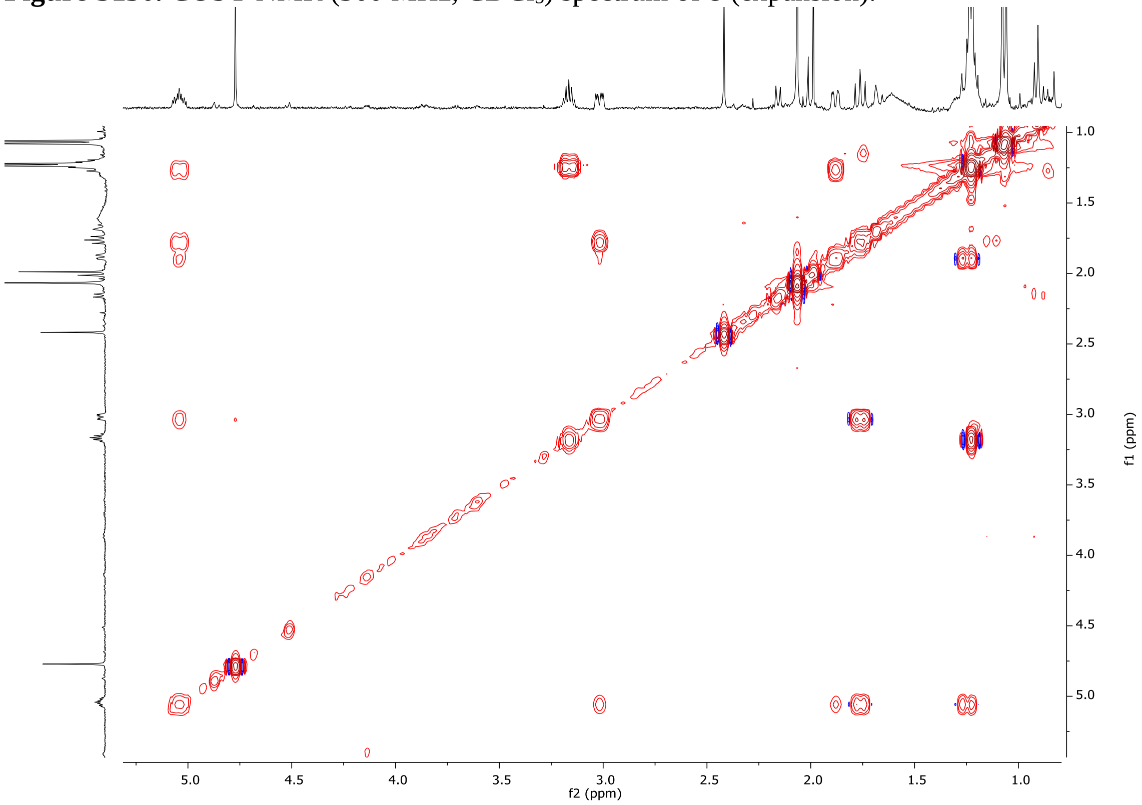

Figure S131. NOESY NMR (500 MHz, $CDCl_3$) spectrum of **8**.

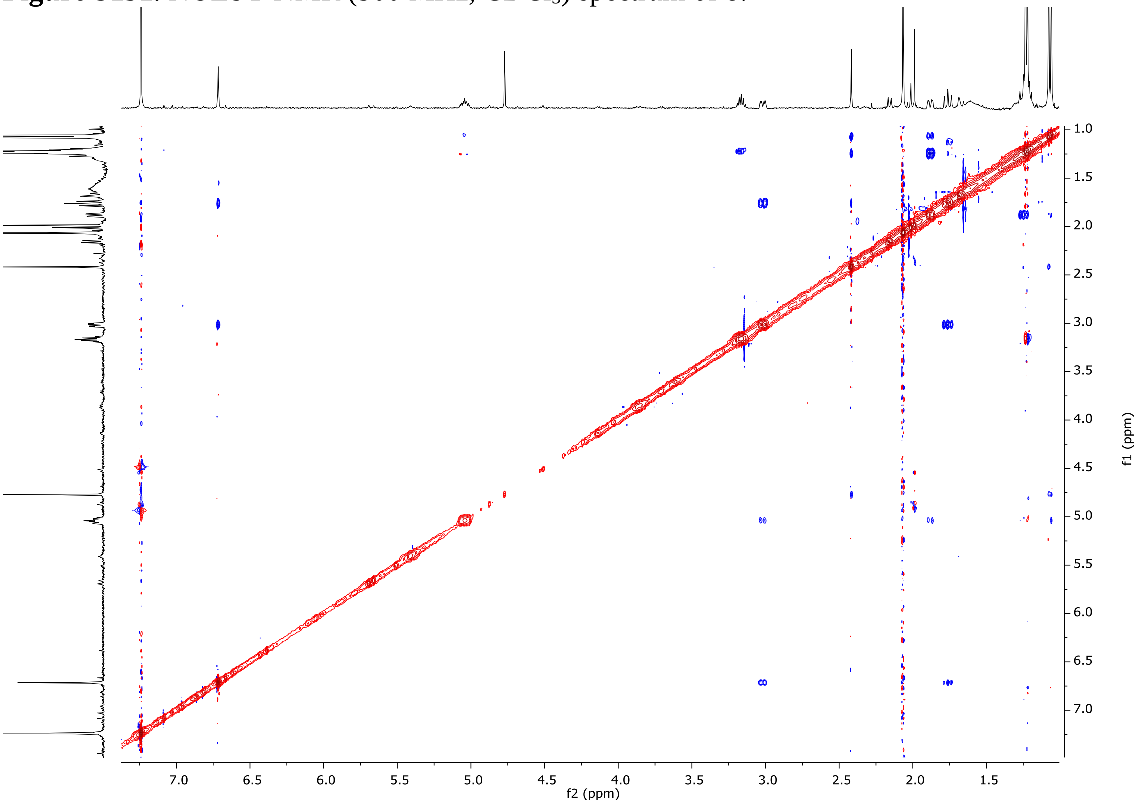

Figure S132. IR spectrum of compound **9**.

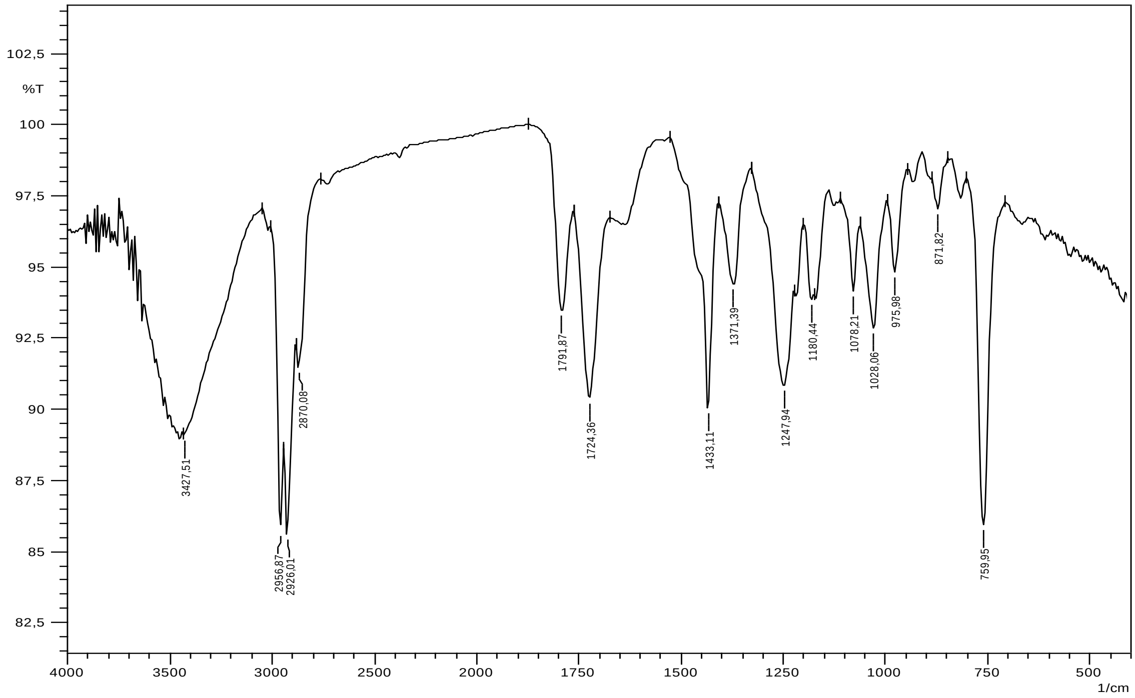

Figure S133. HRESIMS Spectrum of **9** ([2M + Na]$^+$ positive ion mode)

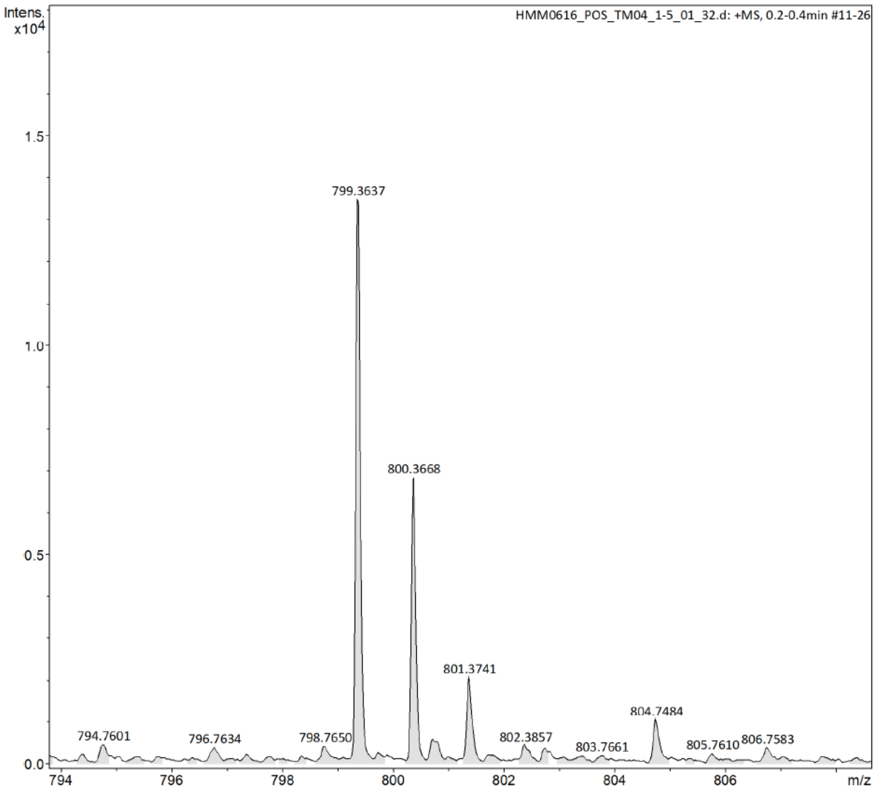

Figure S134. 1H NMR (500 MHz, CDCl3) spectrum of 9.

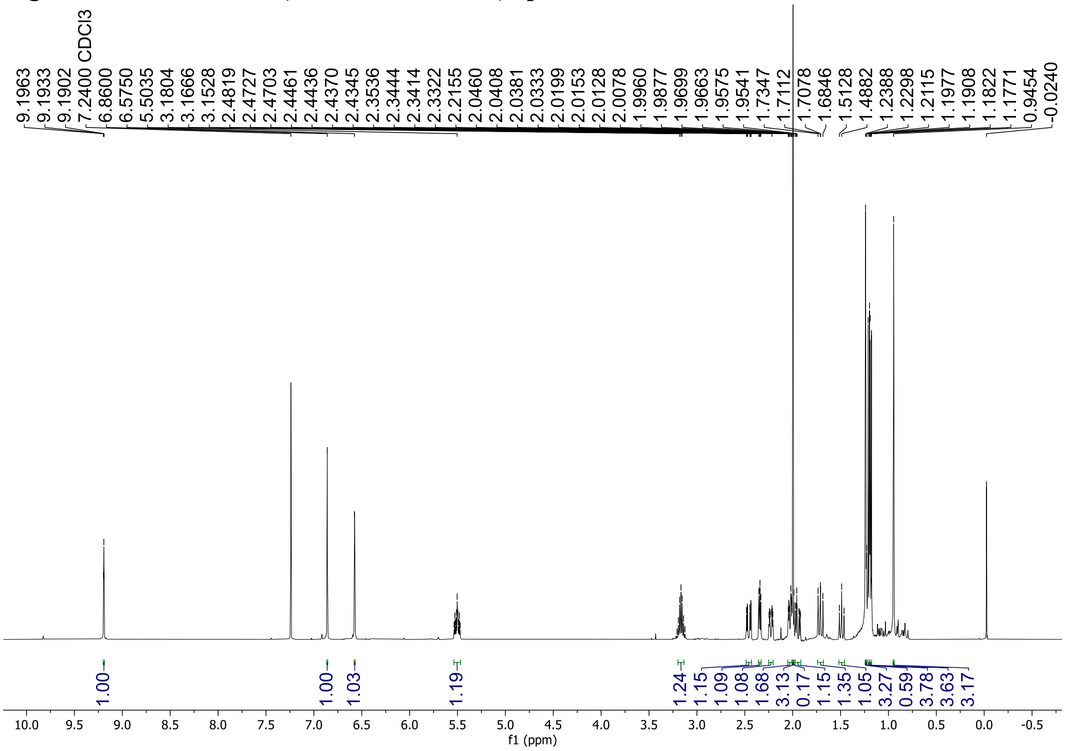

Figure S135. ^{1}H NMR (500 MHz, $CDCl_3$) spectrum of **9** (expansion).

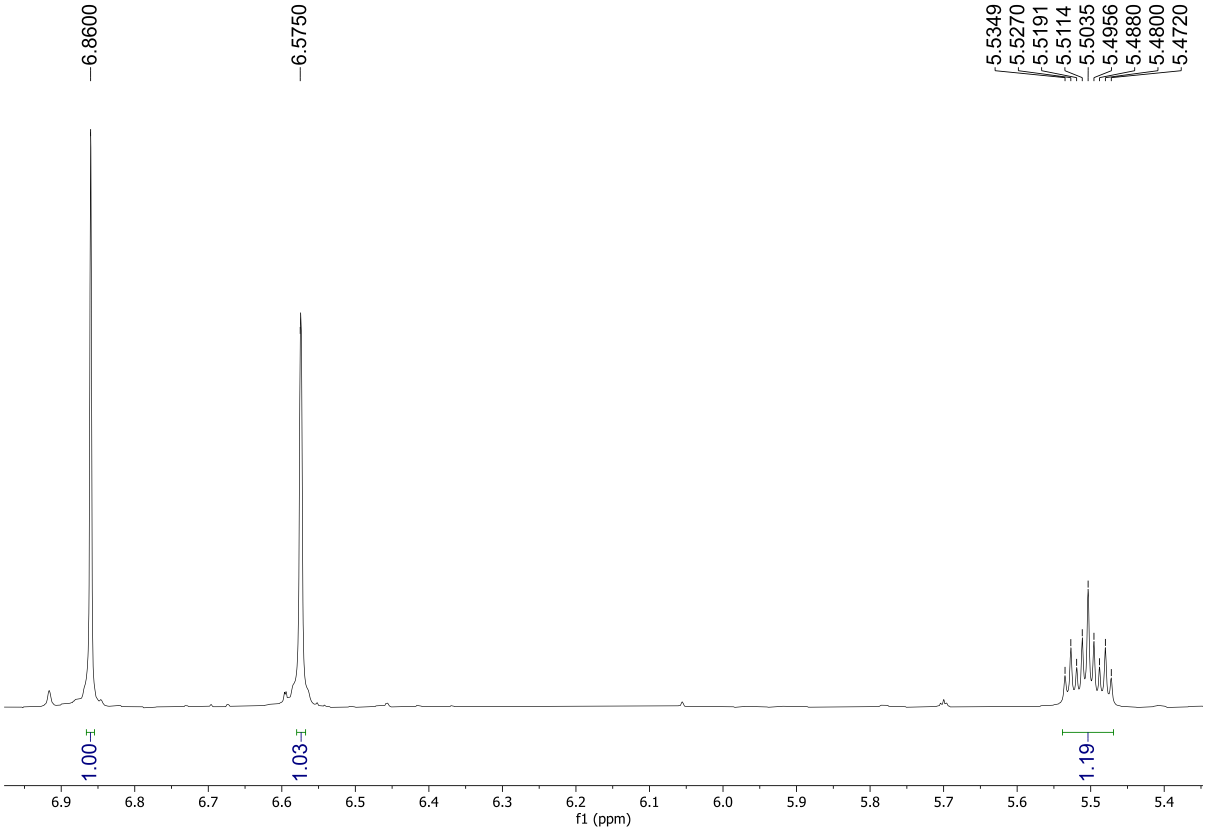

Figure S136. ^{1}H NMR (500 MHz, $CDCl_3$) spectrum of **9** (expansion).

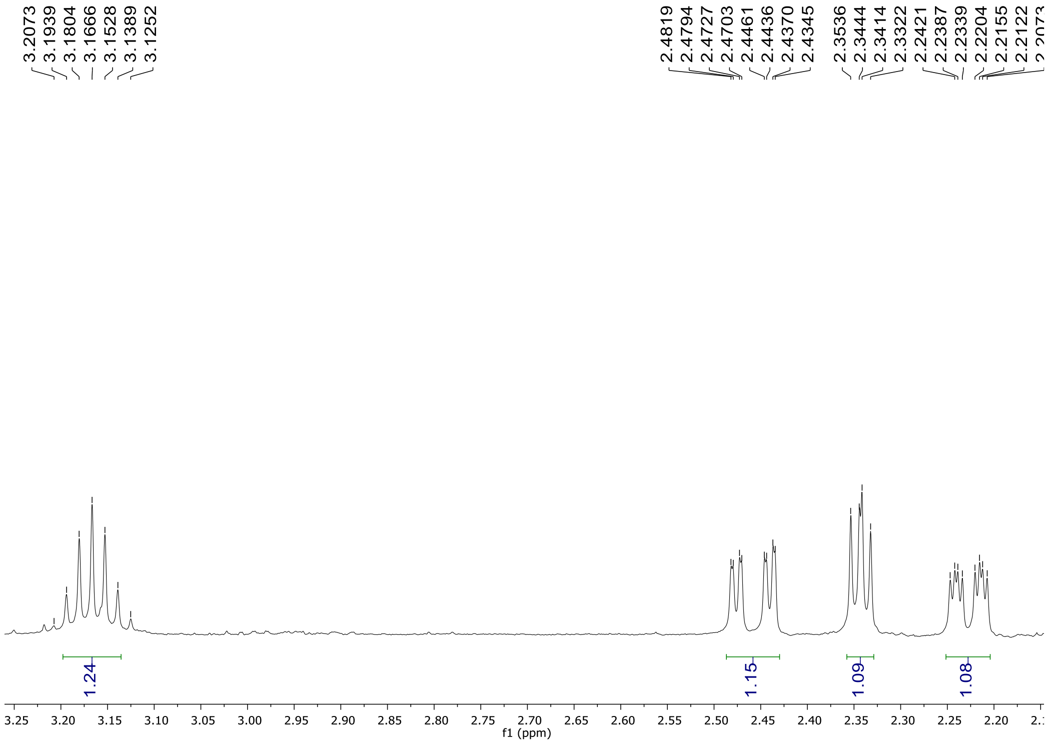

Figure S137. ^{1}H NMR (500 MHz, $CDCl_3$) spectrum **9** (expansion).

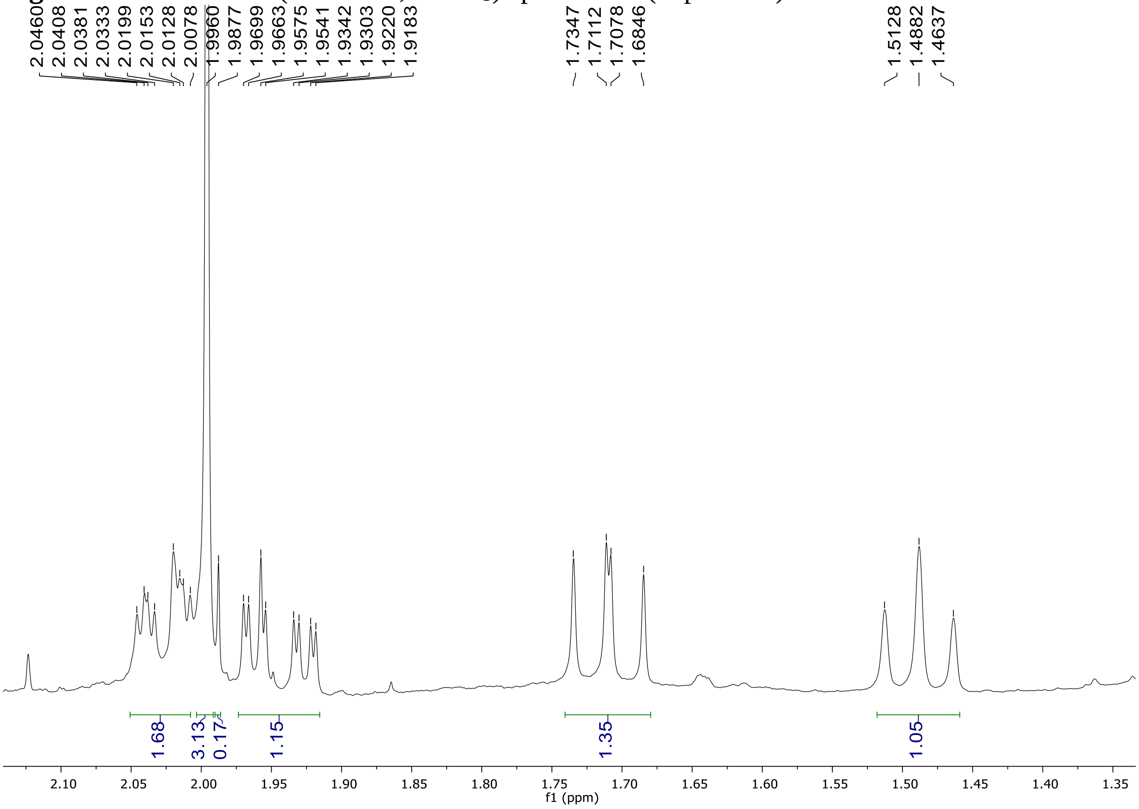

Figure S138. ^{1}H NMR (500 MHz, $CDCl_3$) spectrum of **9** (expansion).

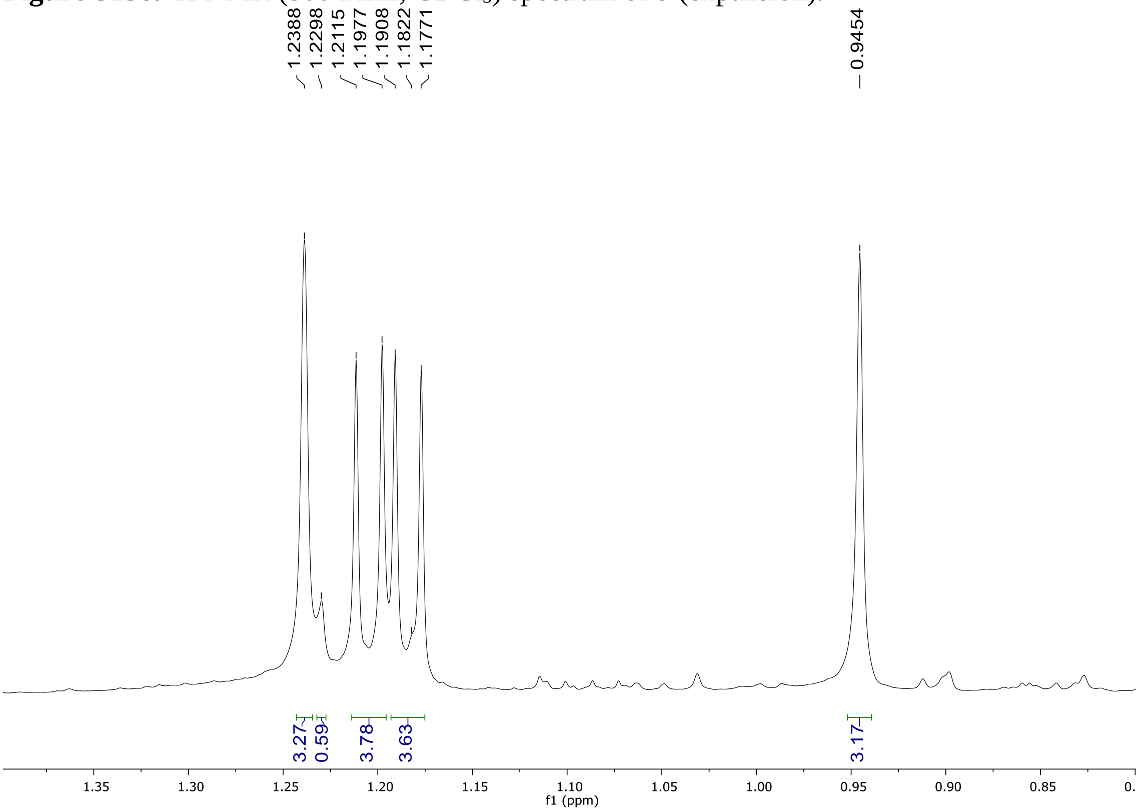

Figure S139. ^{13}C NMR - BB (125 MHz, $CDCl_3$) spectrum of **9**.

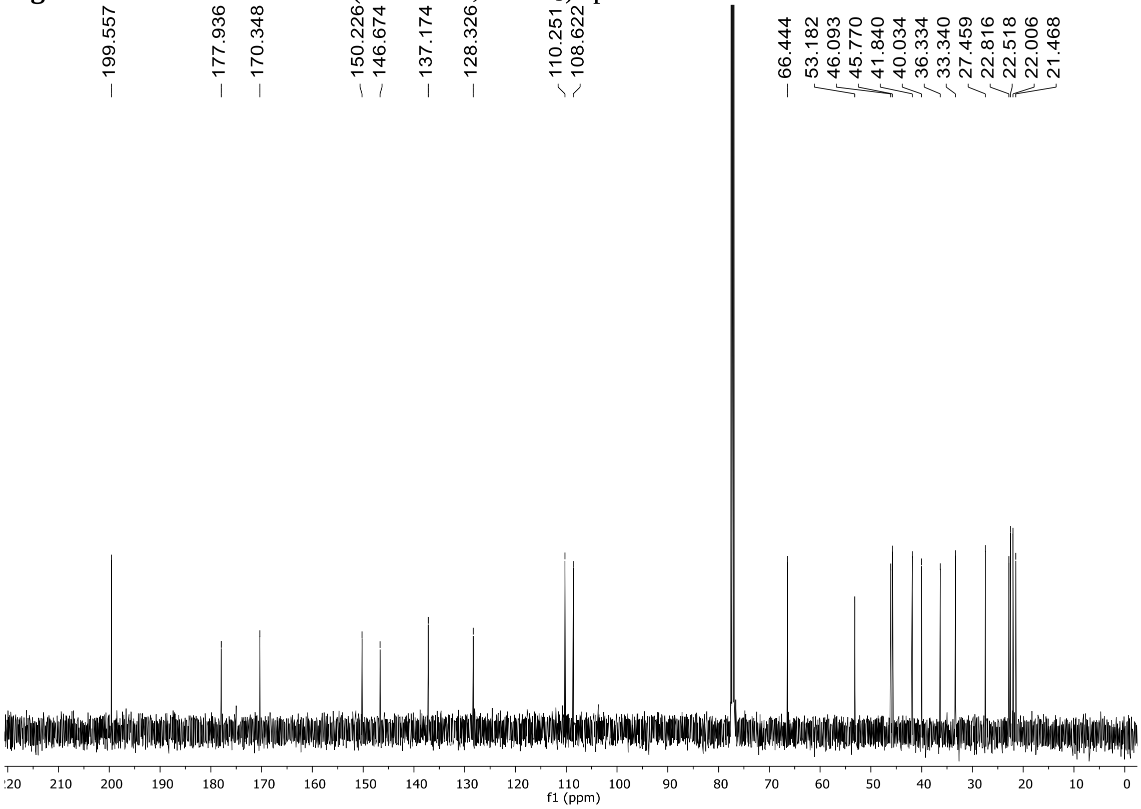

Figure S140. ^{13}C NMR – DEPTQ 135 (125 MHz, $CDCl_3$) spectrum of **9.**

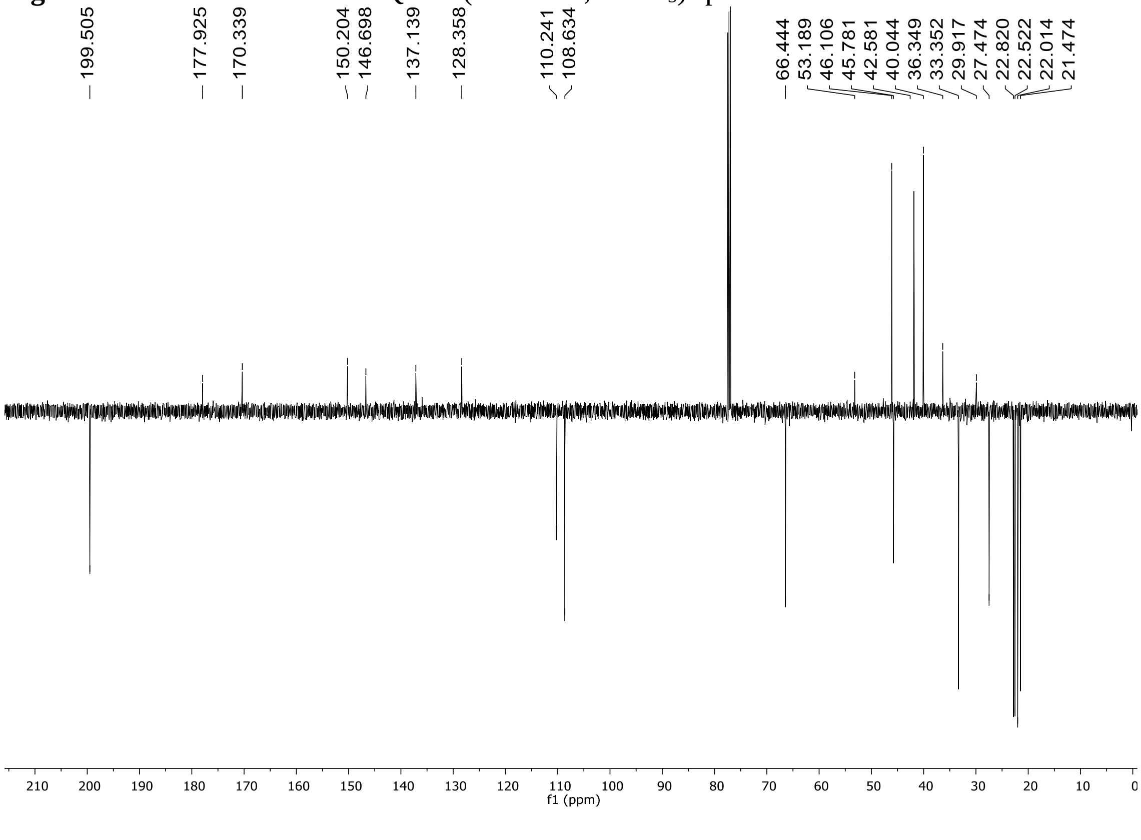

Figure S141. HSQC NMR (500 X 125 MHz, $CDCl_3$) spectrum of **9.**

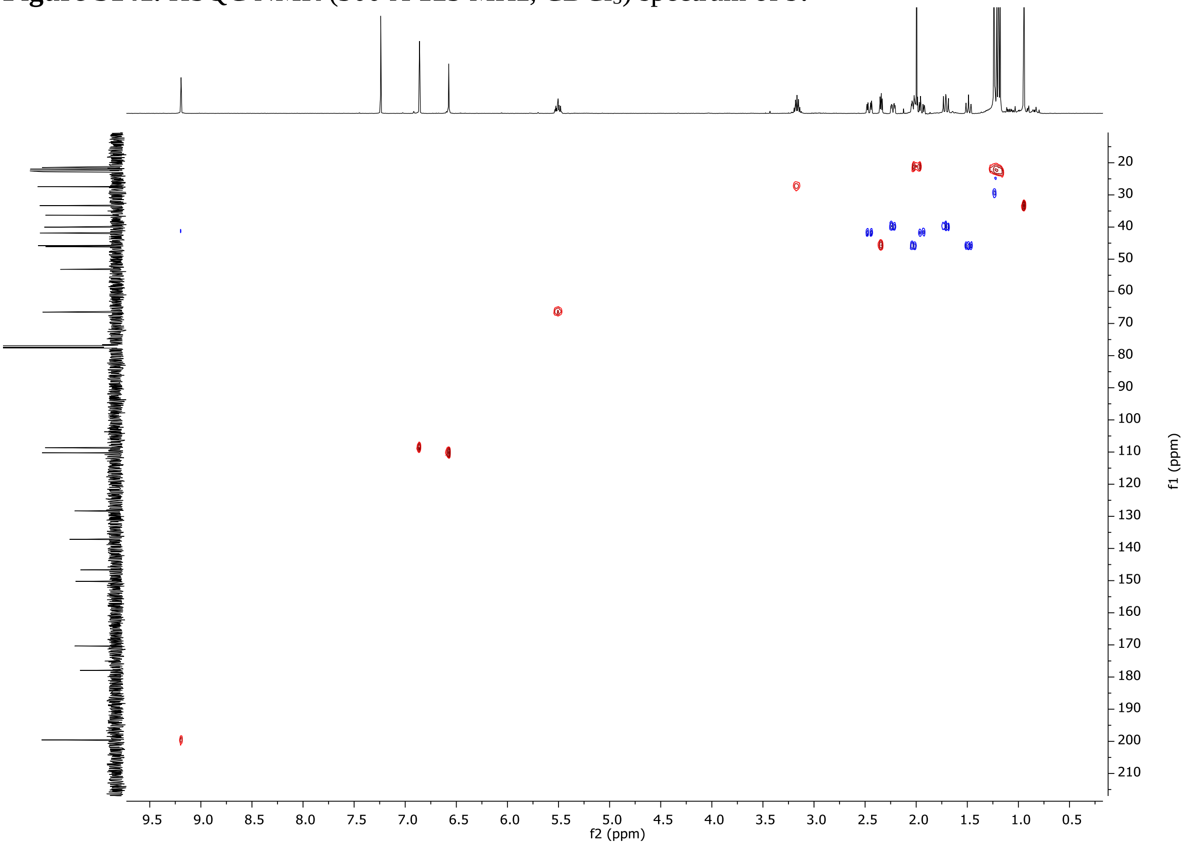

Figure S142. HSQC NMR (500 X 125 MHz, $CDCl_3$) spectrum of **9** (expansion).

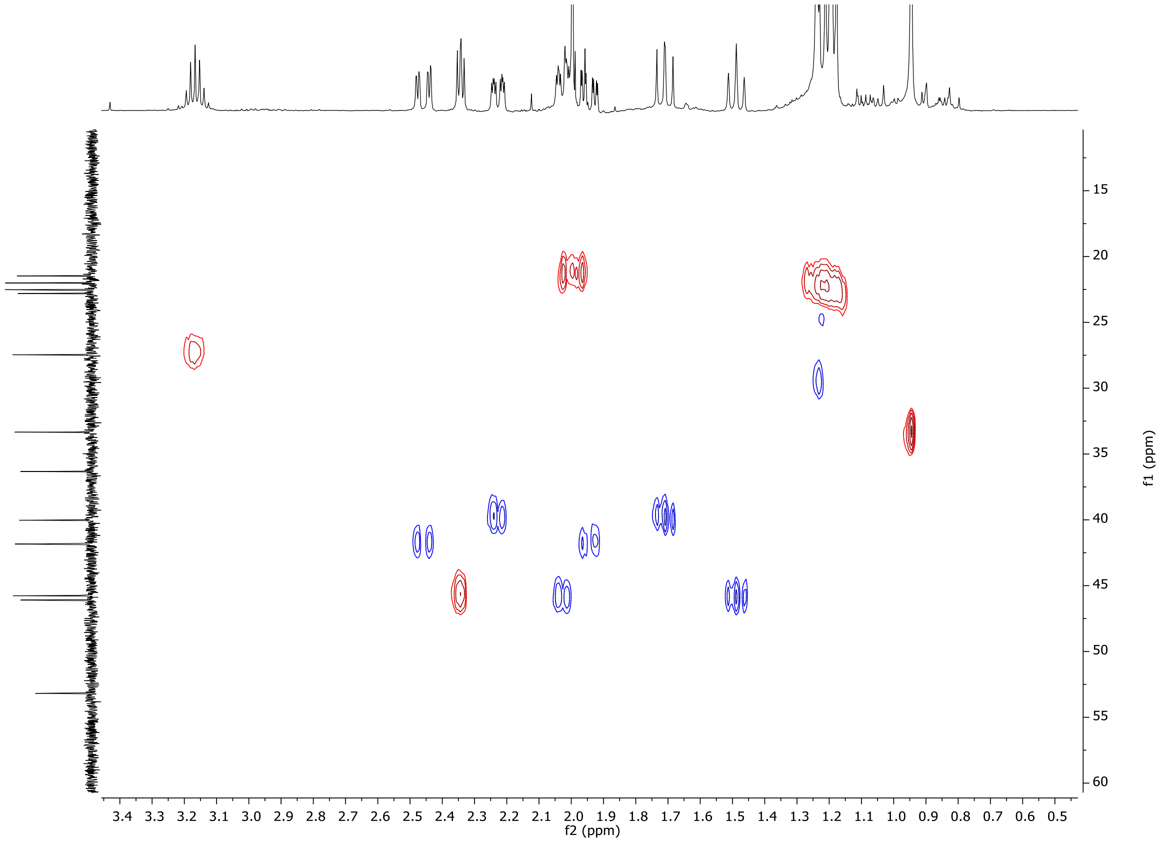

Figure S143. HMBC NMR (500 X 125 MHz, $CDCl_3$) spectrum of **9.**

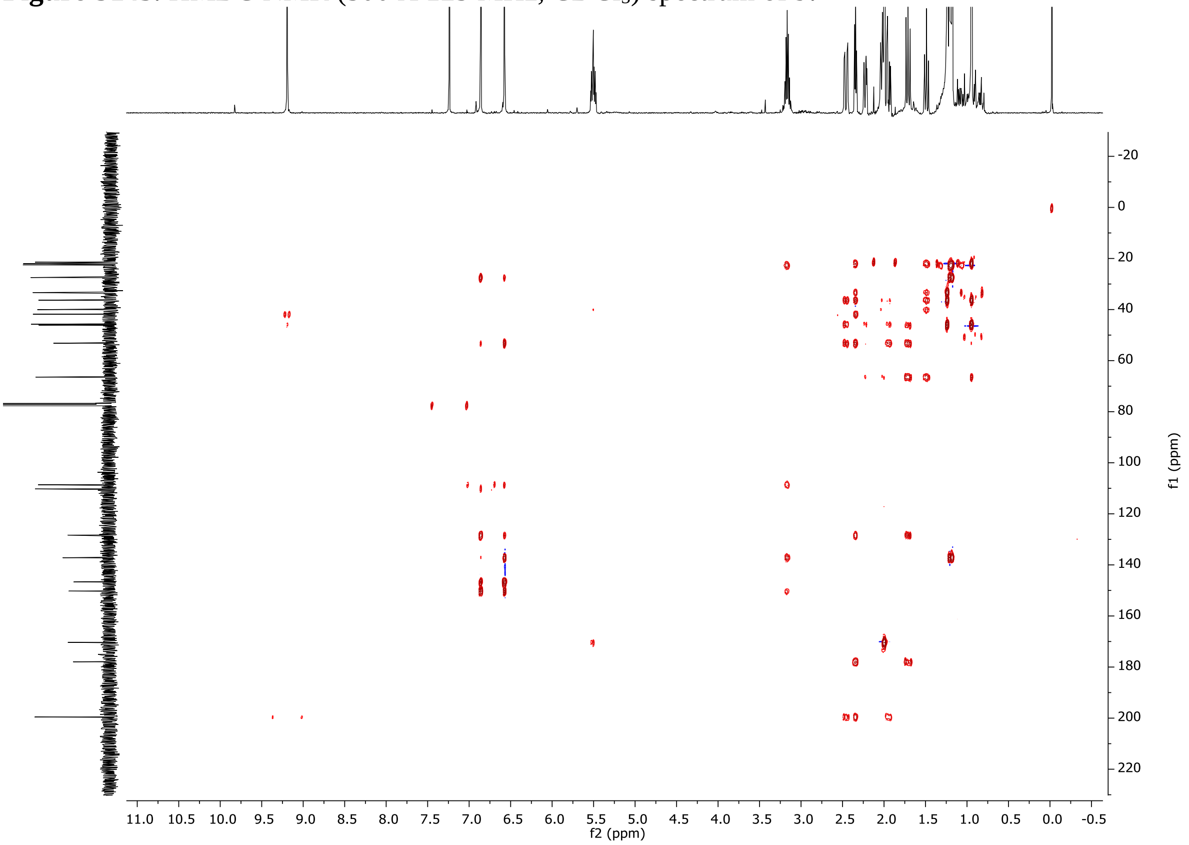

Figure S144. HMBC NMR (500 X 125 MHz, $CDCl_3$) spectrum of **9** (expansion).

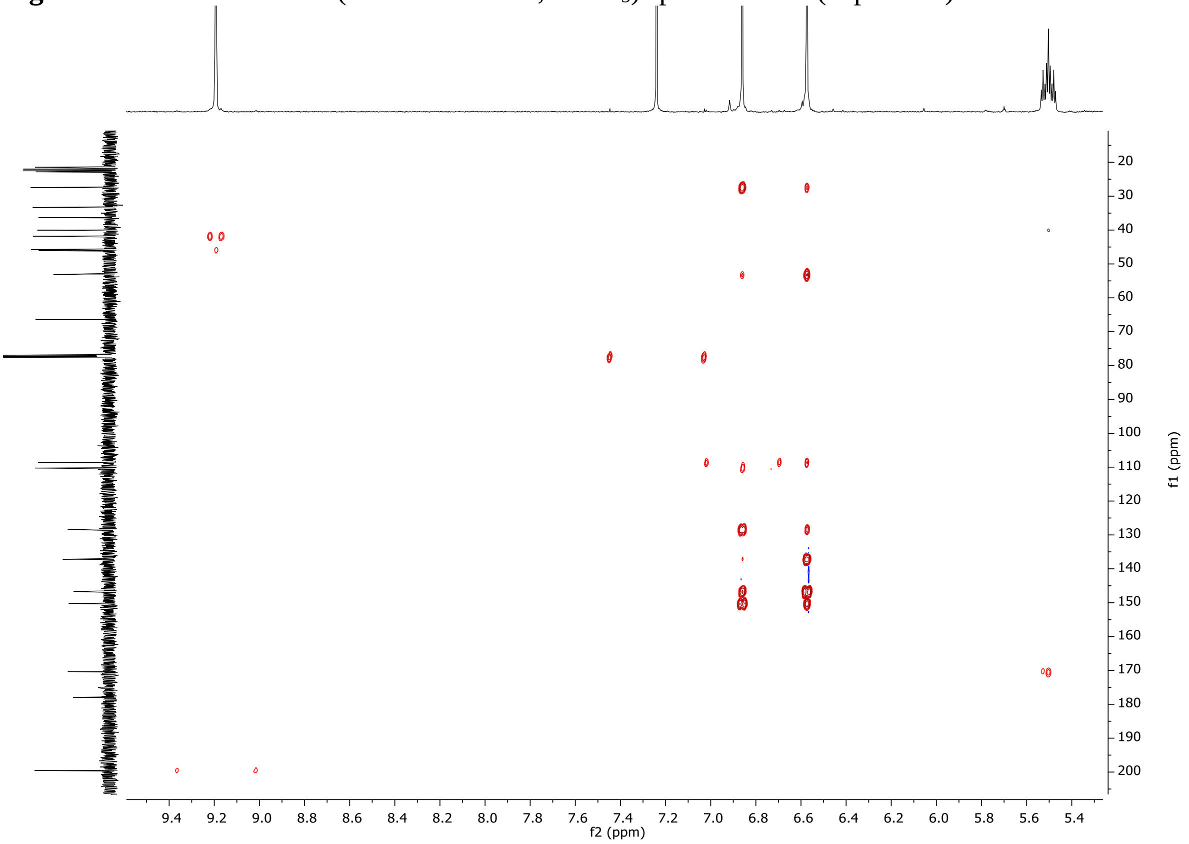

Figure S145. HMBC NMR (500 X 125 MHz, CDCl$_3$) spectrum of **9** (expansion).

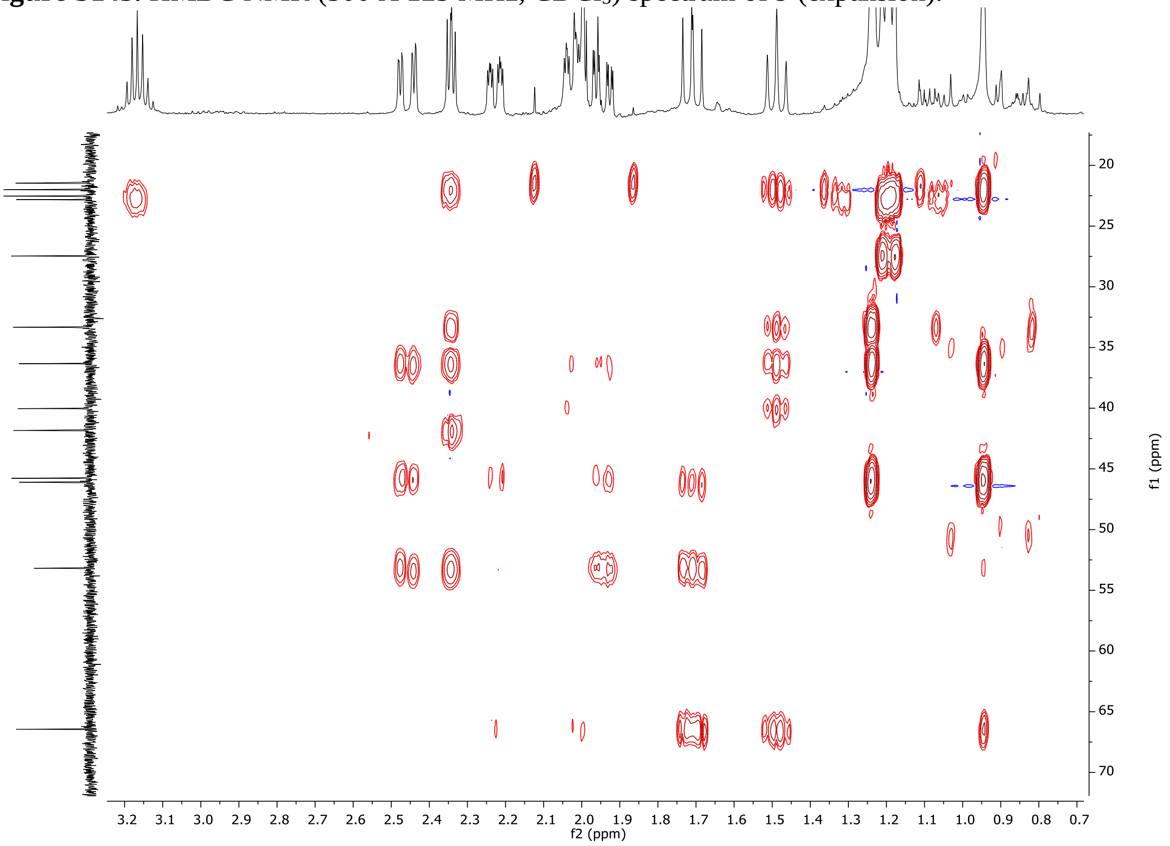

Figure S146. HMBC NMR (500 X 125 MHz, CDCl$_3$) spectrum of **9** (expansion).

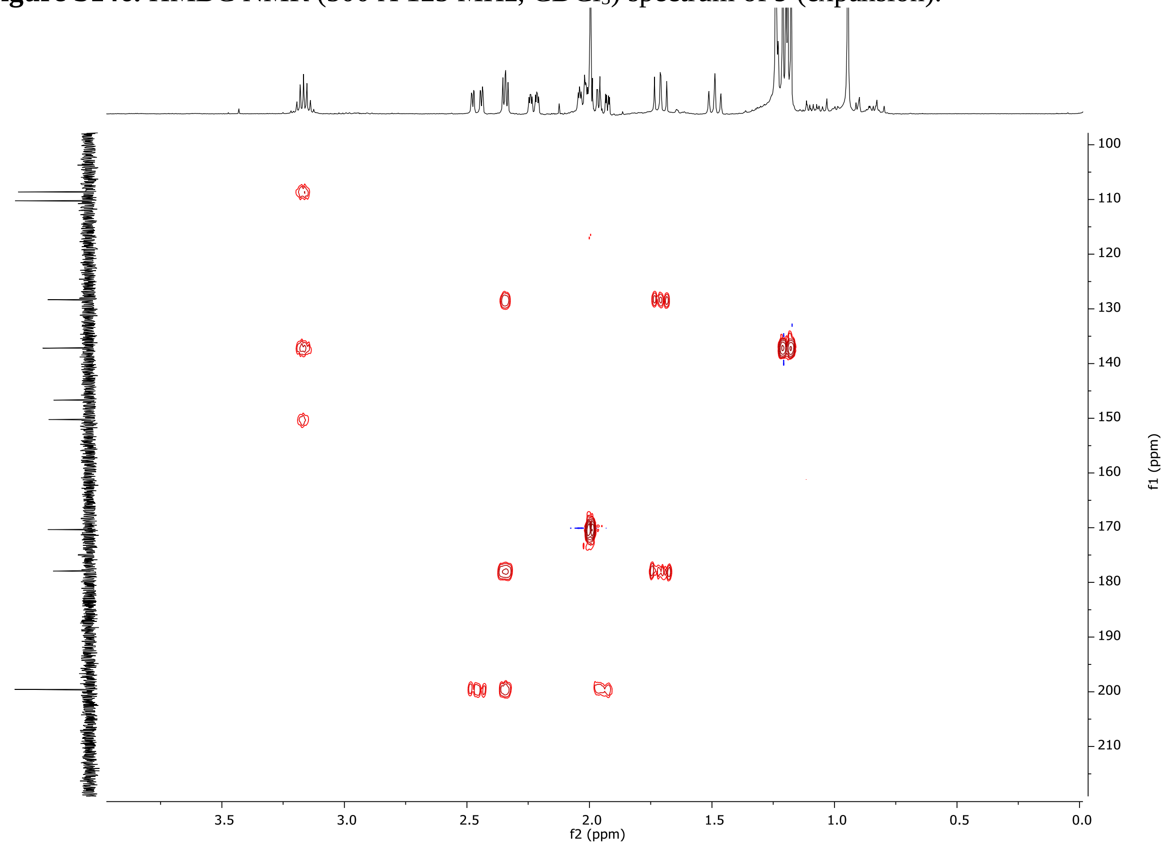

Figure S147. COSY NMR (500 MHz, $CDCl_3$) spectrum of **9.**

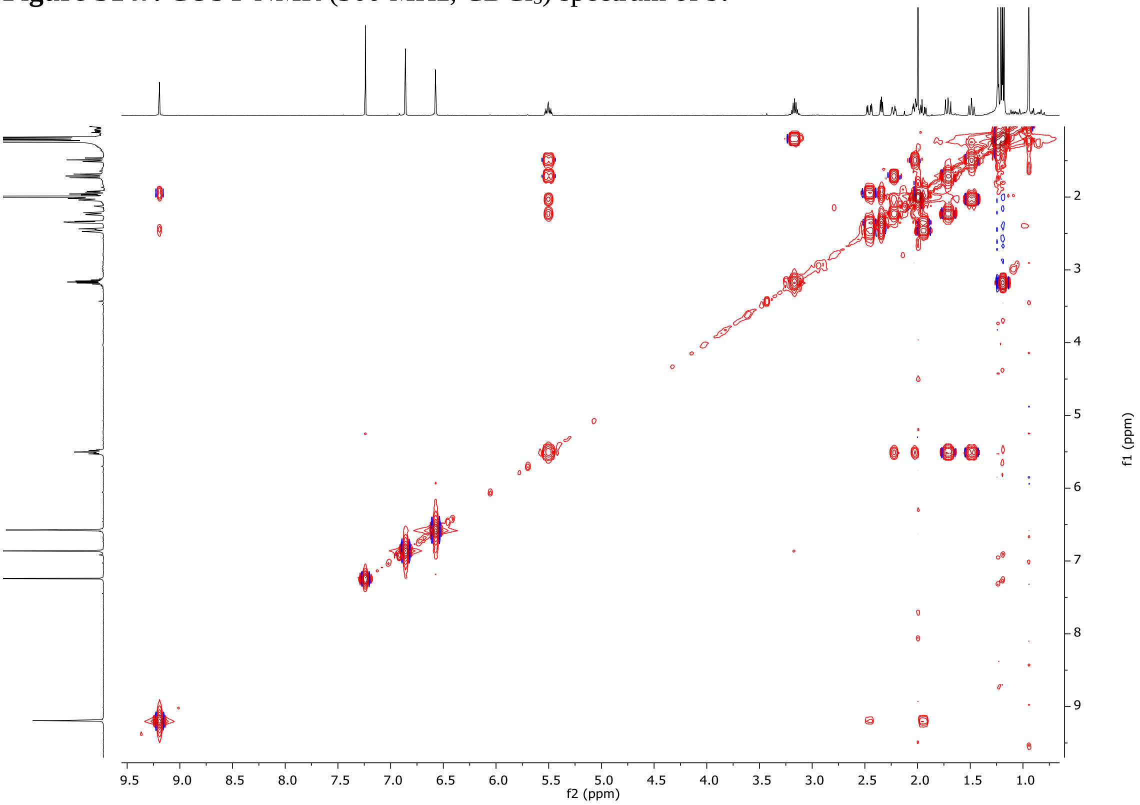

Figure S148. COSY NMR (500 MHz, $CDCl_3$) spectrum of **9** (expansion).

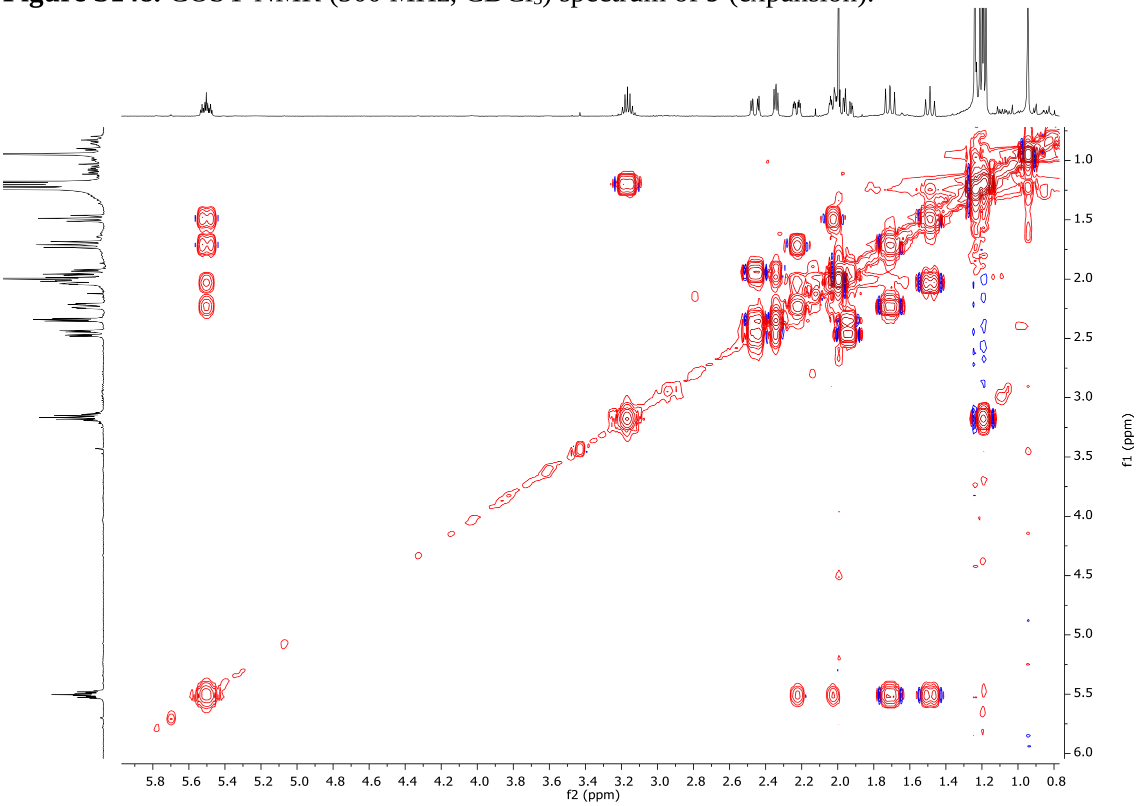

Figure S149. NOESY NMR (500 MHz, $CDCl_3$) spectrum of **9.**

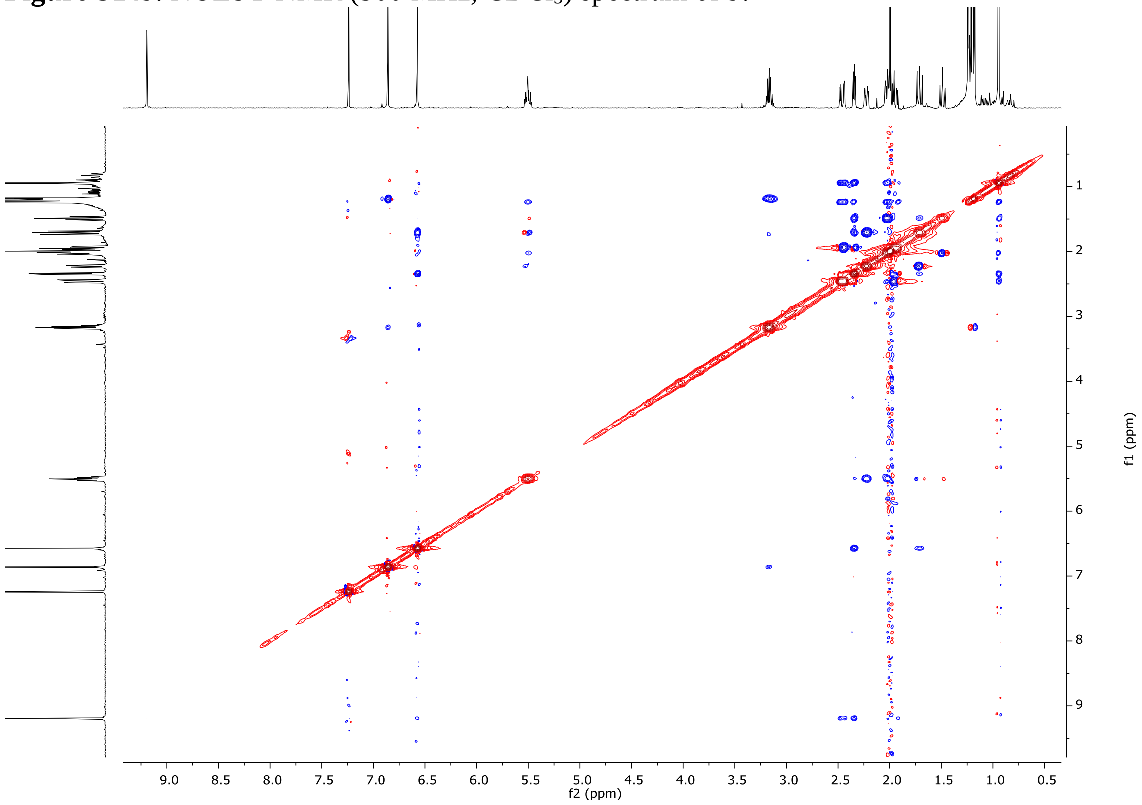

Figure S150. Results of DP4+ analysis of **1**.

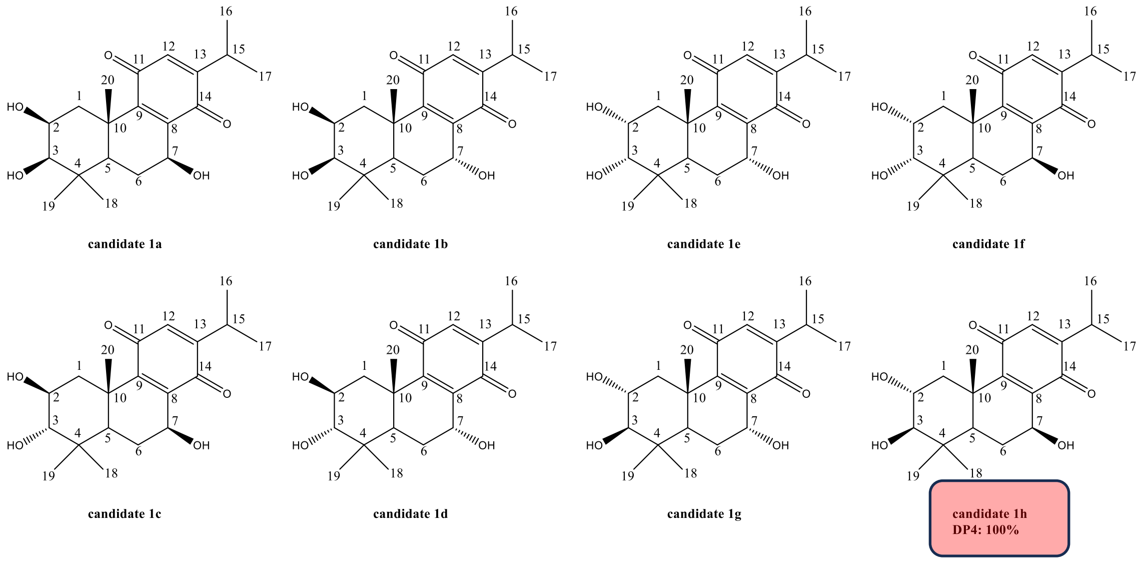

Table S1. Comparison of ^{13}C NMR (δ_{calc}) chemical shifts simulated for all candidates of 1 (GIAO-mPW1PW91/6-31+G(d,p)//B3LYP/6-31G(d)) with the experimental values of the isolated natural product.

Atom	**1a** (δ_{calc})	**1b** (δ_{calc})	**1c** (δ_{calc})	**1d** (δ_{calc})	**1e** (δ_{calc})	**1f** (δ_{calc})	**1g** (δ_{calc})	**1h** (δ_{calc})	**^{13}C RMN Exp.**
**C1**	42.3	40.7	46.2	46.2	45.5	46.1	43.2	44.1	42.3
**C2**	74.9	75.3	70.5	70.9	75.0	74.4	71.4	71.1	68.8
**C3**	78.7	78.9	77.9	77.9	76.2	76.3	83.7	82.9	82.8
**C4**	42.4	43.4	41.2	41.4	40.9	40.8	42.5	42.5	38.9
**C5**	49.8	46.8	46.5	43.1	42.8	46.0	47.5	49.9	48.3
**C6**	28.2	27.8	30.1	30.1	30.6	30.3	27.9	28.6	26.1
**C7**	71.2	67.7	70.2	66.9	67.1	70.4	67.6	70.6	67.9
**C8**	142.9	142.3	141.8	141.7	142.2	142.2	142.2	142.9	141.9
**C9**	151.0	150.0	150.3	151.0	151.2	150.8	150.0	150.2	150.6
**C10**	44.3	44.7	45.4	43.9	43.7	44.8	45.7	46.0	40.0
**C11**	189.0	189.0	188.8	188.8	188.8	188.9	188.6	188.6	188.0
**C12**	131.4	132.3	131.2	130.6	130.9	130.8	131.8	131.5	132.4
**C13**	152.0	152.5	152.4	152.7	152.5	152.3	152.2	152.4	153.6
**C14**	191.5	192.5	191.2	192.3	192.4	191.4	192.5	191.4	190.1
**C15**	33.4	33.5	32.4	32.5	33.3	31.9	33.8	33.4	26.4
**C16**	20.3	20.3	19.8	18.9	19.6	21.9	24.6	25.7	21.5
**C17**	25.5	25.6	23.9	23.7	23.7	22.0	20.3	20.1	21.4
**C18**	30.6	30.7	23.8	24.0	25.9	26.6	29.6	29.1	28.8
**C19**	20.4	18.2	24.6	24.1	26.6	24.7	19.3	20.0	17.0
**C20**	24.3	21.8	27.6	27.0	25.1	26.3	21.5	22.8	21.1
**MAD**	**2.6**	**2.3**	**3.0**	**3.1**	**3.4**	**2.9**	**1.9**	**2.2**	
**RMSD**	**3.2**	**3.1**	**3.7**	**3.7**	**4.2**	**3.7**	**2.6**	**2.8**	

Table S2. Comparison of ^{1}H NMR (δ_{calc}) chemical shifts simulated for all candidates of 1 (GIAO-mPW1PW91/6-31+G(d,p)//B3LYP/6-31G(d)) with the experimental values of the isolated natural product.

Atom	1a (δ_{calc})	1b (δ_{calc})	1c (δ_{calc})	1d (δ_{calc})	1e (δ_{calc})	1f (δ_{calc})	1g (δ_{calc})	1h (δ_{calc})	^{1}H RMN Exp.
**H1α**	1.28	1.32	1.90	1.96	1.49	1.46	1.13	1.04	1.15
**H1β**	3.36	3.30	2.05	2.03	2.73	2.67	3.05	3.07	3.06
**H2**	4.23	4.12	3.50	3.54	4.19	4.20	3.89	3.93	3.82
**H3**	3.18	3.31	3.84	3.74	3.84	3.93	3.08	2.92	3.01
**H5**	1.27	1.73	1.44	1.87	2.36	2.06	1.73	1.27	1.15
**H6α**	2.09	1.93	1.97	1.84	1.97	2.04	1.96	2.13	2.20
**H6β**	1.81	1.88	1.66	1.77	1.79	1.65	1.83	1.75	1.64
**H7**	4.87	4.72	4.87	4.77	4.78	4.91	4.74	4.91	4.79
**H12**	6.48	6.40	6.48	6.41	6.37	6.48	6.47	6.50	6.36
**H15**	2.79	2.80	2.90	2.89	2.84	2.92	2.81	2.86	2.97
**H16 (CH_3)**	1.21	1.19	1.14	1.08	1.15	1.07	1.12	1.09	1.10
**H17 (CH_3)**	1.08	1.09	1.09	1.08	1.12	1.10	1.19	1.17	1.08
**H18 (CH_3)**	1.01	0.98	1.04	1.01	0.98	1.11	1.11	1.05	1.07
**H19 (CH_3)**	1.10	1.10	0.89	0.92	1.06	0.94	0.90	0.92	0.91
**H20 (CH_3)**	1.77	1.66	1.46	1.34	1.29	1.37	1.40	1.47	1.40
**MAD**	**0.16**	**0.17**	**0.17**	**0.19**	**0.20**	**0.15**	**0.08**	**0.06**	
**RMSD**	**0.20**	**0.21**	**0.32**	**0.35**	**0.33**	**0.29**	**0.14**	**0.08**	

Figure S151. Comparison of the observed ECD spectrum of compound **1** in ACN (black solid line) with the calculated [CAM-B3LYP/PCM(ACN)/TZVP] ECD spectra of the Boltzmann average of the 8 lowest-energy conformers identified candidate 1h (2R,3R,5R,7S,10S) (blue solid line) and its enantiomer (2S,3S,5S,7R,10R) (red dashed line) of 1.

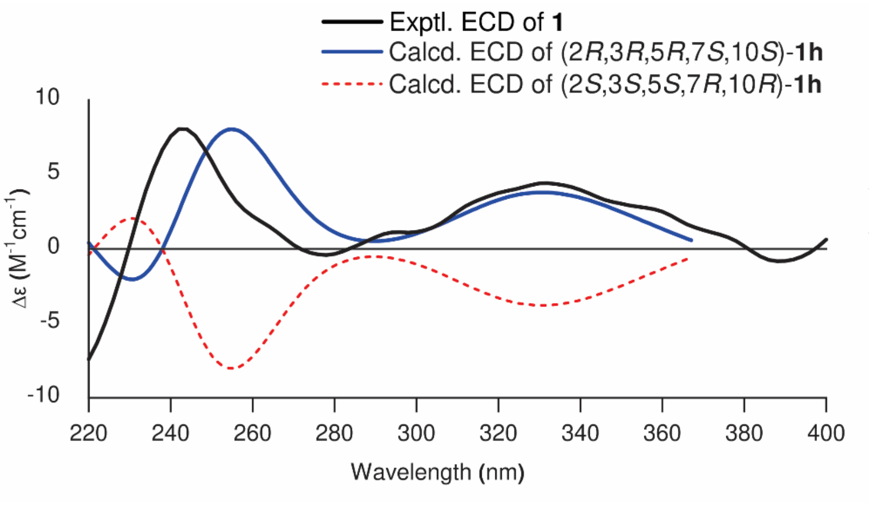

Figure S152. The 8 lowest energy conformers of candidate 1h at B3LYP/6-31G(d) level.

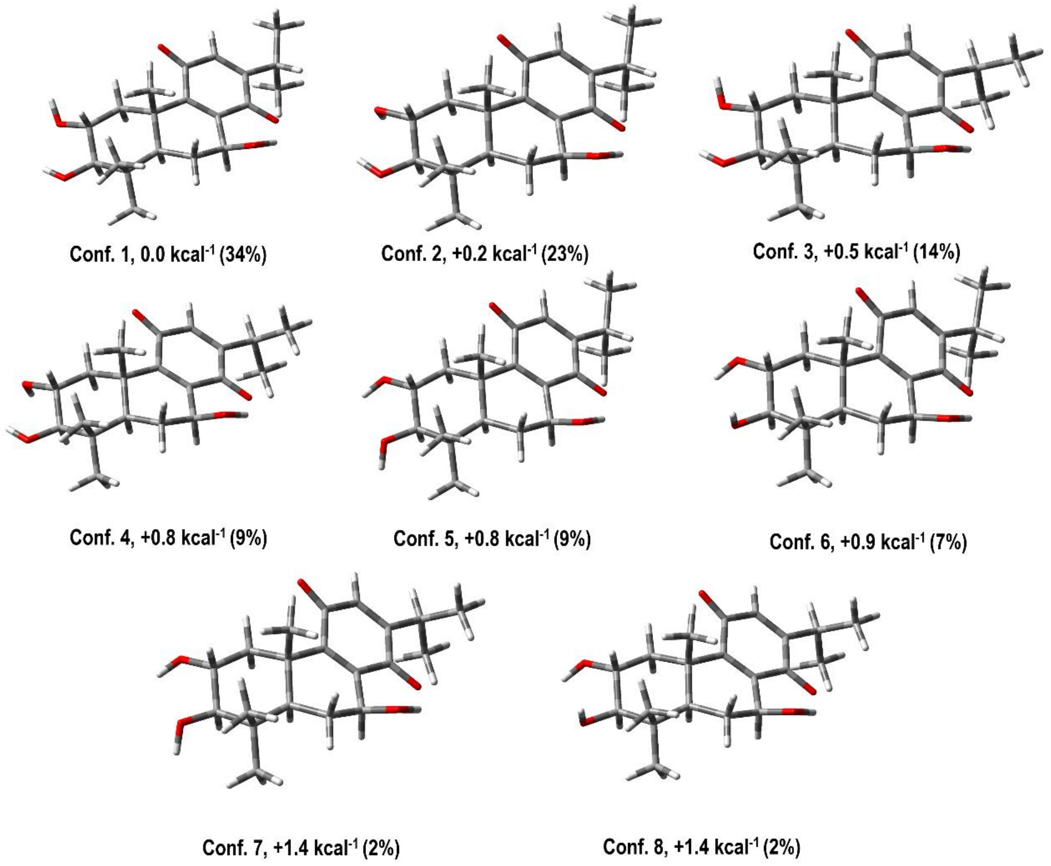

For candidate 1h, 13 conformers with relative energy within 10 kcal mol^{-1} of the lowest energy conformer were selected for further geometry optimized at the B3LYP/6-31G(d) level. Eight conformers were identified with relative energy < 2.5 kcal mol^{-1}, corresponding to more than 99.97% of the total Boltzmann distribution.

Figure S153. Results of DP4+ analysis of **2**.

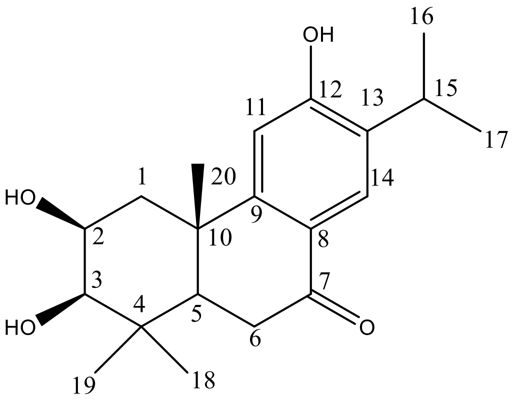

candidate 2a

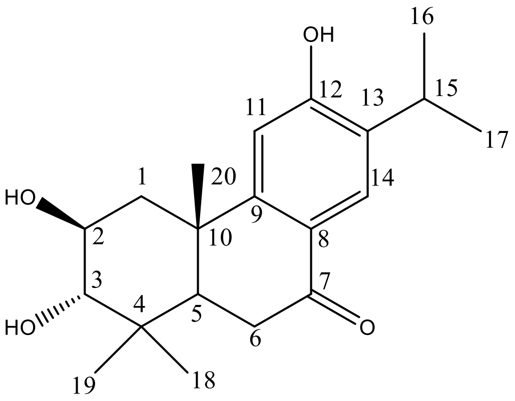

candidate 2b

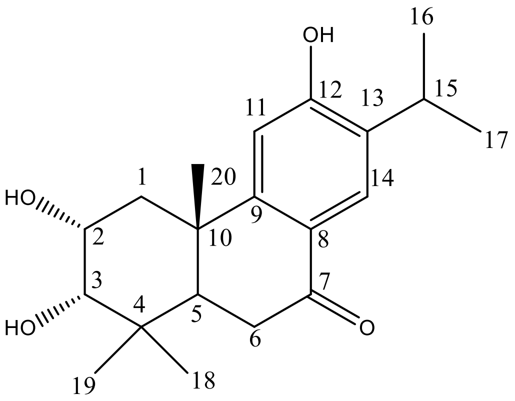

candidate 2c

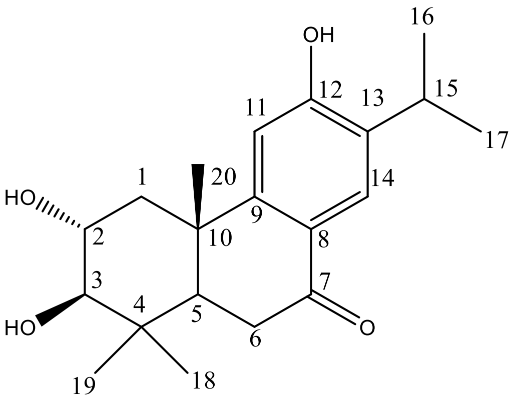

candidate 2d
DP4+: 100%

Table S3. Comparison of ^{13}C NMR (δ_{calc}) chemical shifts simulated for all candidates of 2 (GIAO-mPW1PW91/6-31+G(d,p)//B3LYP/6-31G(d)) with the experimental values of the isolated natural product.

**Atom**	**2a** (δ_{calc})	**2b** (δ_{calc})	**2c** (δ_{calc})	**2d** (δ_{calc})	**^{13}C RMN Exp.**
**C1**	42.8	47.7	42.8	45.4	45.6
**C2**	73.8	69.8	73.8	70.4	69.4
**C3**	78.9	78.8	78.9	83.6	83.5
**C4**	42.5	41.4	42.5	43.2	40.5
**C5**	51.3	48.1	51.3	50.7	50.3
**C6**	37.8	39.7	37.8	38.7	36.6
**C7**	194.5	193.9	194.5	194.2	200.6
**C8**	122.1	123.2	122.1	123.4	123.7
**C9**	154.5	154.0	154.5	153.8	157.2
**C10**	41.5	44.5	41.5	44.6	39.9
**C11**	108.3	110.8	108.3	108.6	110.6
**C12**	157.2	156.3	157.2	156.0	162.6
**C13**	129.9	130.8	129.9	131.1	135.2
**C14**	127.3	127.0	127.3	127.7	127.3

**C15**	34.2	32.1	34.2	31.0	27.9
**C16**	22.5	24.1	22.5	23.5	22.8
**C17**	23.2	23.4	23.2	24.6	22.9
**C18**	21.4	23.8	21.4	28.4	28.6
**C19**	27.1	24.7	27.1	18.1	16.9
**C20**	27.1	30.5	27.1	26.0	24.6
**MAD**	**3.4**	**3.2**	**3.2**	**2.1**	
**RMSD**	**4.3**	**4.0**	**4.1**	**2.9**	

Table S4. Comparison of ^{1}H NMR (δ_{calc}) chemical shifts simulated for all candidates of 2 (GIAO-mPW1PW91/6-31+G(d,p)//B3LYP/6-31G(d)) with the experimental values of the isolated natural product.

Atom	2a (δ_{calc})	2b (δ_{calc})	2c (δ_{calc})	2d (δ_{calc})	^{1}H RMN Exp.
**H1α/H1β**	1.76/2.74	2.55/1.98	2.16/2.48	1.43/2.44	1.56/2.55
**H2**	4.35	3.69	4.23	3.85	3.83
**H3**	3.37	3.87	3.95	3.06	3.02
**H5**	1.91	2.13	2.57	1.93	1.89
**H6/H6'**	2.80/2.45	2.72/2.37	2.70/2.42	2.82/2.48	2.64/2.64
**H11**	6.82	6.7	6.67	6.66	6.76
**H14**	8.32	8.39	8.40	8.47	7.8
**H15**	2.99	3.27	3.26	3.35	3.22
**H16 (CH_3)**	1.29	1.21	1.25	1.24	1.19
**H17 (CH_3)**	1.28	1.27	1.25	1.21	1.21
**H18 (CH_3)**	1.09	0.87	1.11	0.96	1.06
**H19 (CH_3)**	1.00	1.01	0.93	0.96	0.98
**H20 (CH_3)**	1.63	1.4	1.28	1.41	1.27
	**0.17**	**0.20**	**0.17**	**0.10**	
	**0.23**	**0.33**	**0.30**	**0.16**	

Figure S154. Comparison of the observed ECD spectrum of compound 2 in ACN (black solid line) with the calculated [CAM-B3LYP/PCM(ACN)/TZVP] ECD spectra of the Boltzmann average of the 15 lowest-energy conformers identified candidate 2d (2R,3R,5R,10S) (blue solid line) and its enantiomer (2S,3S,5S,10R) (red dashed line) of 2.

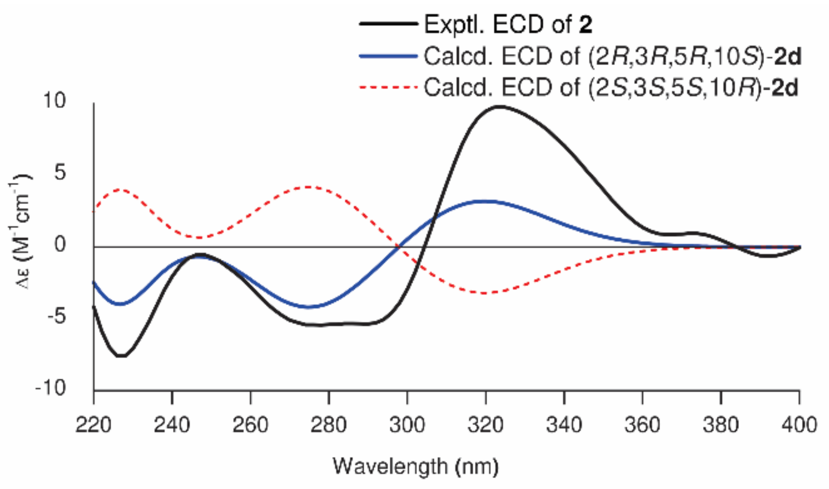

Figure S155. The 15 lowest energy conformers of candidate 2d at B3LYP/6-31G(d) level.

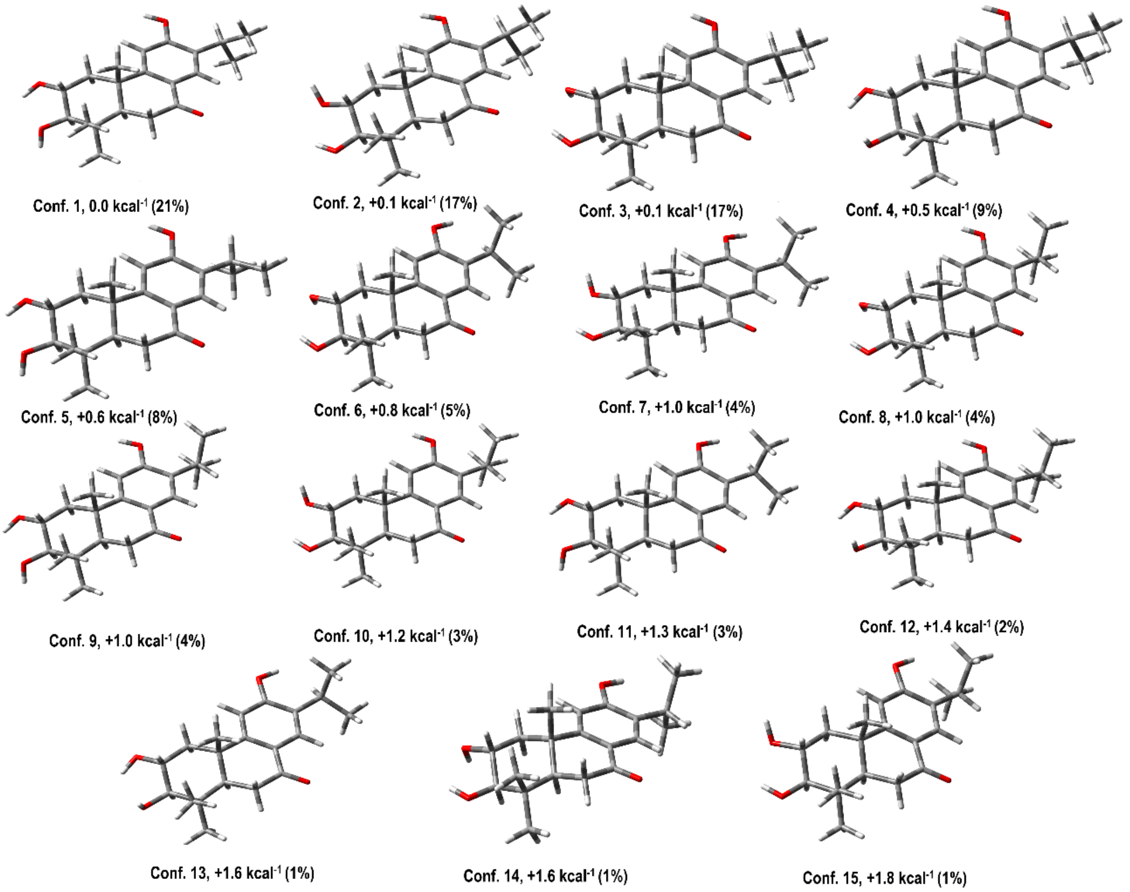

For candidate 2d, 19 conformers with relative energy within 10 kcal mol^{-1} of the lowest energy conformer were selected for further geometry optimized at the B3LYP/6-31G(d) level. 15 conformers were identified with relative energy < 2.0 kcal mol^{-1}, corresponding to more than 98.27% of the total Boltzmann distribution.

Figure S156. Results of DP4+ analysis of 3.

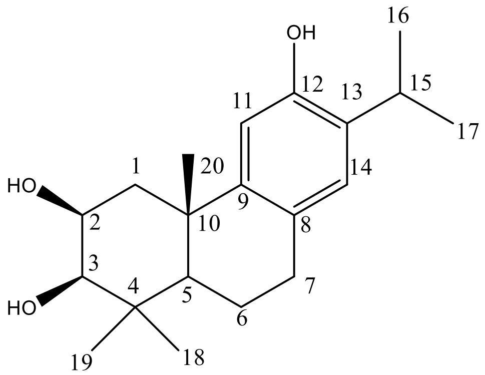

candidate 3a

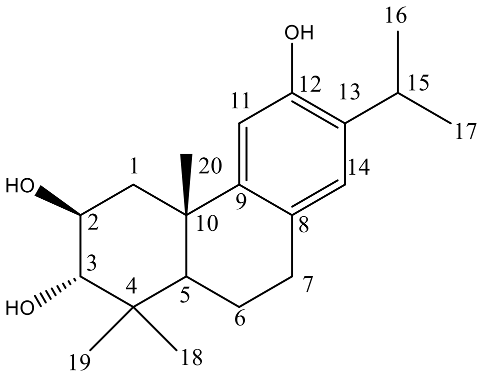

candidate 3b

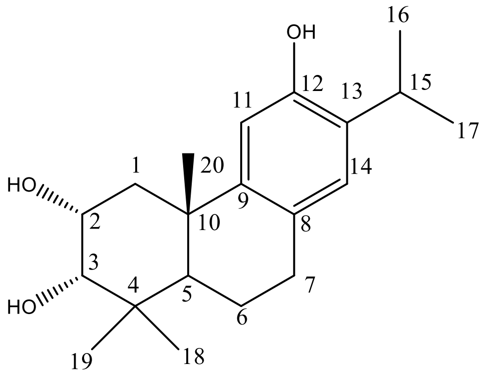

candidate 3c

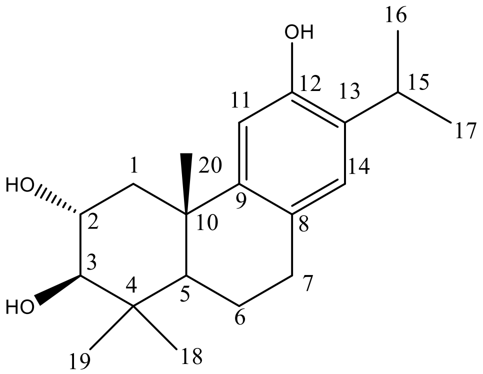

candidate 3d
DP4+: 100%

Table S5. Comparison of ^{13}C NMR (δ_{calc}) chemical shifts simulated for all candidates of 3 (GIAO-mPW1PW91/6-31+G(d,p)//B3LYP/6-31G(d)) with the experimental values of the isolated natural product.

Atom	3a (δ_{calc})	3b (δ_{calc})	3c (δ_{calc})	3d (δ_{calc})	^{13}C RMN Exp.
C1	44.8	48.5	41.6	46.2	46.6
C2	74.8	70.5	69.6	71.5	70
C3	79.1	78.9	79.8	84.3	84.3
C4	42.5	41.6	43.4	43.1	40.5
C5	52.2	49.1	45.5	51.5	51.5
C6	22.6	24.1	22.7	22.9	20.4
C7	34.2	33.7	33.8	34.0	31.1
C8	125.3	125.5	125.7	125.4	126.5
C9	146.1	145.8	145.1	144.6	148
C10	41.1	43.4	45.2	44.3	39.5
C11	107.8	110.3	108.7	108.7	111.6
C12	149.2	149.4	148.7	148.8	153.4
C13	130.2	129.8	129.5	129.8	133.8
C14	126.2	126.5	126.9	127.1	127.4
C15	32.0	31.9	32.6	32.7	27.7

**C16**	24.0	23.9	23.8	23.6	23.3
**C17**	23.4	24.0	23.9	24.1	23.2
**C18**	30.6	24.4	29.1	29.1	29.4
**C19**	18.9	24.3	23.3	18.9	17.4
**C20**	29.3	33.3	27.8	28.3	26.3
**MAD**	**2.4**	**3.0**	**3.0**	**2.1**	
**RMSD**	**2.8**	**3.6**	**3.6**	**2.6**	

Table S6. Comparison of ^{1}H NMR (δ_{calc}) chemical shifts simulated for all candidates of 3 (GIAO-mPW1PW91/6-31+G(d,p)//B3LYP/6-31G(d)) with the experimental values of the isolated natural product.

**Atom**	**3a** (δ_{calc})	**3b** (δ_{calc})	**3c** (δ_{calc})	**3d** (δ_{calc})	**^{1}H RMN Exp.**
**H1α/H1β**	1.61/2.54	2.48/1.90	1.72/2.01	1.30/2.43	1.40/2.49
**H2**	4.26	3.67	4.20	3.88	3.78
**H3**	3.24	3.89	3.42	2.97	2.99
**H5**	1.52	1.71	1.94	1.45	1.32
**H6α/H6β**	1.69/1.85	1.59/1.75	1.57/1.79	1.63/1.80	1.84/1.71
**H7/H7’**	2.82/2.81	2.77/2.77	2.84/2.85	2.81/2.86	2.71/2.82
**H11**	6.64	6.48	6.69	6.64	6.65
**H14**	7.11	7.19	7.27	7.25	6.75
**H15**	3.18	3.24	3.23	3.23	3.16
**H16 (CH_3)**	1.18	1.19	1.23	1.23	1.16
**H17 (CH_3)**	1.21	1.19	1.23	1.22	1.15
**H18 (CH_3)**	1.02	0.89	1.00	1.01	1.07
**H19 (CH_3)**	1.09	1.00	0.82	0.87	0.89
**H20 (CH_3)**	1.56	1.32	1.21	1.25	1.19
	**0.15**	**0.21**	**0.16**	**0.08**	
	**0.20**	**0.33**	**0.24**	**0.12**	

Figure S157. Comparison of the observed ECD spectrum of **3** in ACN (black trace) with the calculated [CAM-B3LYP/PCM(ACN)/TZVP] ECD spectrum of the Boltzmann average of the 14 lowest-energy identified candidate 3d (2R,3R,5R,10S) (blue solid line) and its enantiomer (2S,3S,5S,10R) (red dashed line) of **3**.

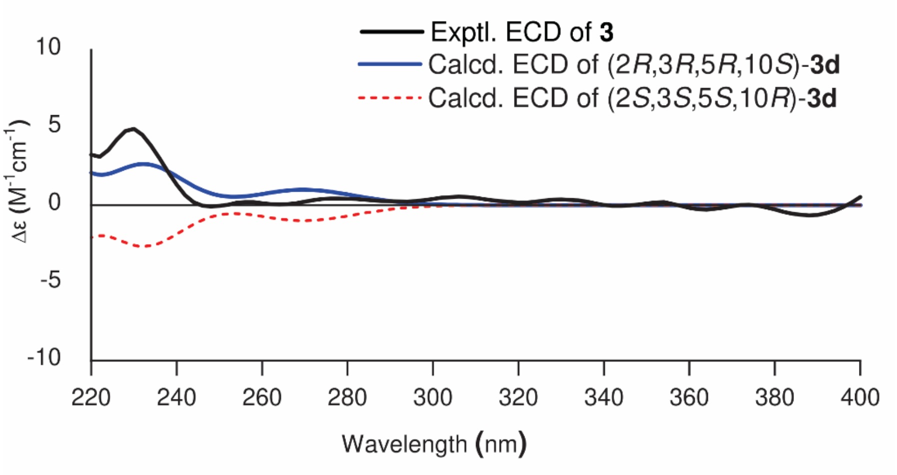

Figure S158. The 14 lowest energy conformers of candidate 3d at B3LYP/6-31G(d) level.

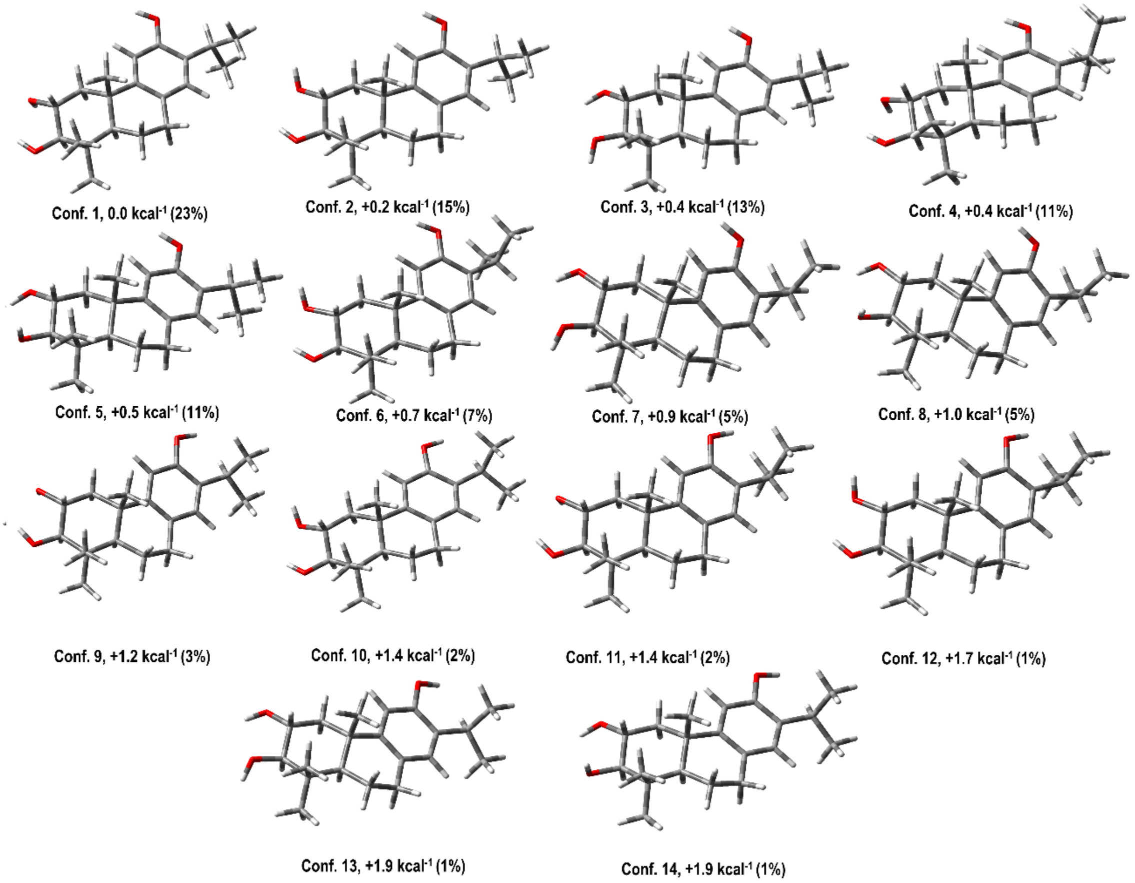

For candidate 3d, 16 conformers with relative energy within 10 kcal mol^{-1} of the lowest energy conformer were selected for further geometry optimized at the B3LYP/6-31G(d) level. 14 conformers were identified with relative energy < 2.0 kcal mol^{-1}, corresponding to more than 98.86% of the total Boltzmann distribution.

Figure S159. Results of DP4+ analysis of **4**.

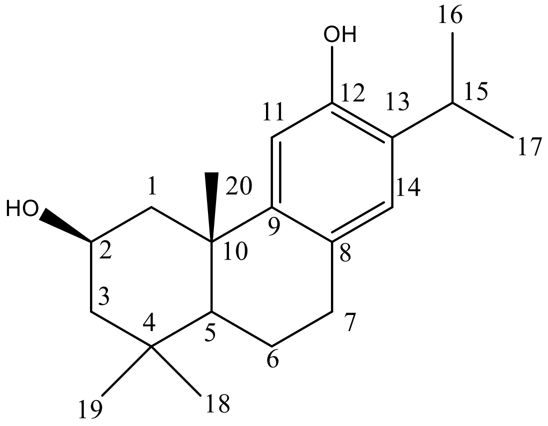

candidate 4a

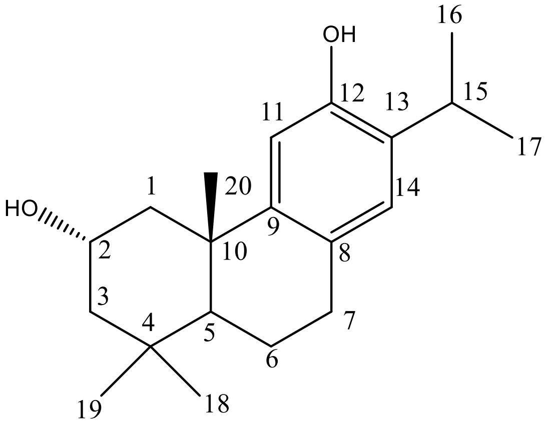

candidate 4b
DP4+: 100%

Table S7. Comparison of ^{13}C NMR (δ_{calc}) and ^{1}H NMR chemical shifts simulated for all candidates of 4 (GIAO-mPW1PW91/6-31+G(d,p)//B3LYP/6-31G(d)) with the experimental values of the isolated natural product.

Carbon	4a (δ_{calc})	4b (δ_{calc})	^{13}C RMN Exp.	Hidrogen	4a (δ_{calc})	4b (δ_{calc})	^{1}H RMN Exp.
**C1**	50.1	49.0	48.9	**H1α/H1β**	2.34/1.67	1.13/2.43	1.27/2.51
**C2**	67.9	67.3	66.1	**H2**	3.95	4.15	3.97
**C3**	46.4	51.3	51.7	**H3/H3’**	1.70/1.42	1.69/1.00	1.81/1.18
**C4**	36.8	38.9	35.7				
**C5**	48.1	51.6	51.7	**H5**	1.55	1.40	1.27
**C6**	24.0	23.2	20	**H6α/H6β**	1.63/1.74	1.67/1.73	1.84/1.67
**C7**	33.9	34.0	30.7	**H7/H7’**	2.77/2.77	2.85/2.82	2.86/2.72
**C8**	125.9	125.5	126.6				
**C9**	146.1	145.0	148.6				
**C10**	44.8	44.6	40.3				
**C11**	110.3	108.4	111.5	**H11**	6.52	6.70	6.66
**C12**	149.3	148.9	153.4				
**C13**	129.7	129.6	133.7				
**C14**	126.4	127.0	127.5	**H14**	7.18	7.22	6.76
**C15**	32.2	32.9	27.8	**H15**	3.24	3.24	3.16
**C16**	23.9	23.8	23.2	**H16 (CH_3)**	1.20	1.24	1.13
**C17**	23.9	24.0	23.3	**H17 (CH_3)**	1.21	1.24	1.16
**C18**	31.6	33.9	34	**H18 (CH_3)**	0.95	0.88	1.01
**C19**	26.6	24.4	23	**H19 (CH_3)**	0.89	0.86	0.97
**C20**	33.5	28.0	26.2	**H20 (CH_3)**	1.31	1.20	1.18
**MAD**	**2.9**	**2.1**			**0.17**	**0.11**	
**RMSD**	**3.4**	**2.7**			**0.29**	**0.14**	

Figure S160. Comparison of the observed ECD spectrum of 4 in ACN (black solid line) with the calculated [CAM-B3LYP/PCM(ACN)/TZVP] ECD spectrum of the Boltzmann average of the 12 lowest-energy conformers identified candidate 4b (2S,5S,10R) (blue solid line) and its enantiomer (2R,5R,10S) (red line) of 4.

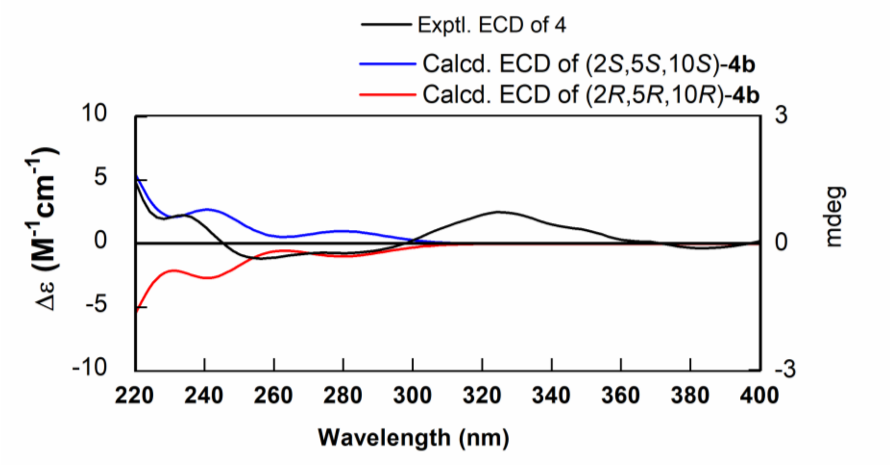

Figure S161. The 12 lowest energy conformers of candidate 4b at B3LYP/6-31G(d) level.

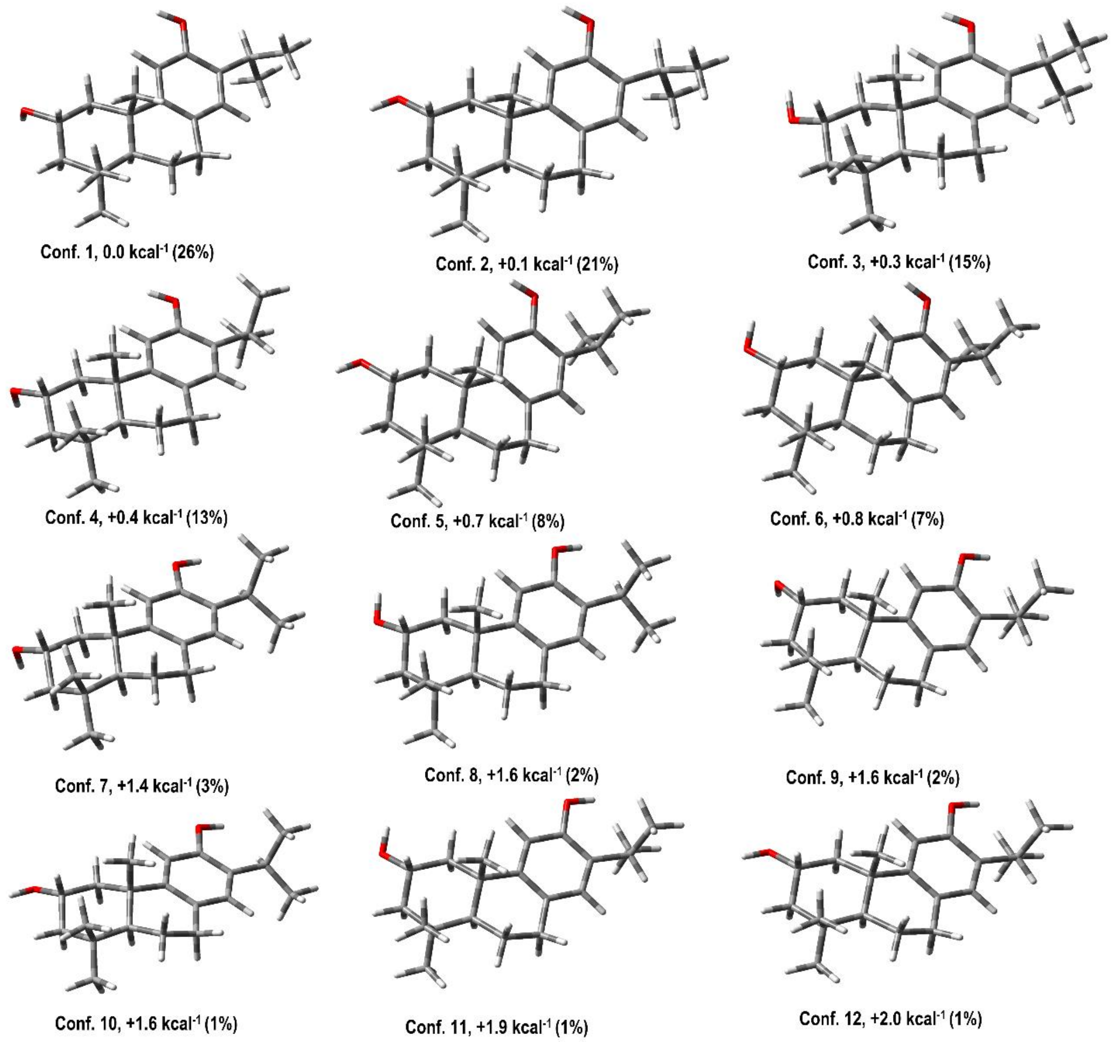

For candidate 4b, 17 conformers with relative energy within 10 kcal mol^{-1} of the lowest energy conformer were selected for further geometry optimized at the B3LYP/6-31G(d) level. 12

conformers were identified with relative energy < 2.5 kcal mol^{-1}, corresponding to more than 99.86% of the total Boltzmann distribution.

Figure S162. Results of DP4+ analysis of **5**.

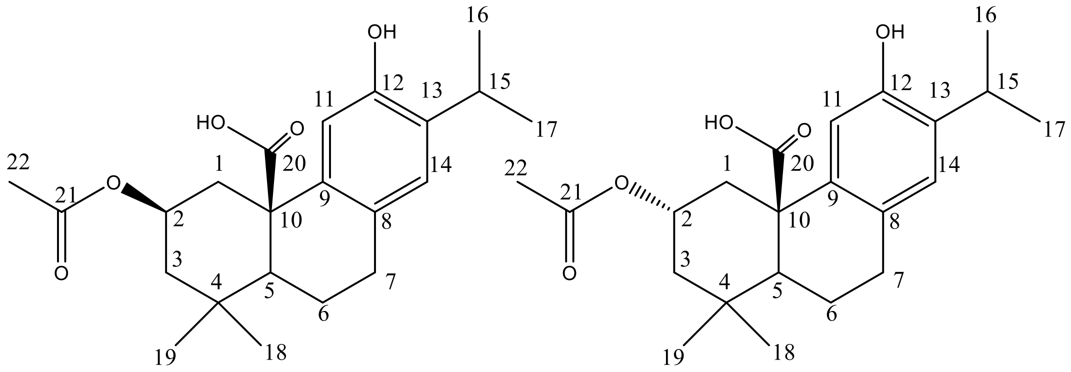

candidate 5a

candidate 5b
DP4+: 100%

Table S8. Comparison of ^{13}C NMR (δ_{calc}) and ^{1}H NMR chemical shifts simulated for all candidates of 5 (GIAO-mPW1PW91/6-31+G(d,p)//B3LYP/6-31G(d)) with the experimental values of the isolated natural product.

Carbon	5a (δ_{calc})	5b (δ_{calc})	^{13}C RMN Exp.	Hidrogen	5a (δ_{calc})	5b (δ_{calc})	^{1}H RMN Exp.
**C1**	43.6	43.3	43	**H1α/H1β**	2.03/2.99	1.21/2.98	1.27/3.09
**C2**	69.7	70.8	70.8	**H2**	5.01	5.43	5.42
**C3**	42.2	47.3	47.7	**H3/H3'**	1.79/1.45	1.85/1.17	1.86/1.27
**C4**	38.1	38.8	35.8				
**C5**	50.7	55.1	53.2	**H5**	1.72	1.44	1.50
**C6**	23.8	22.5	19.6	**H6/H6'**	2.64/1.82	2.95/1.73	2.54/1.86
**C7**	33.1	33.8	30.5	**H7/H7'**	2.85/2.83	2.78/2.81	2.87/2.76
**C8**	127.4	127.6	128.7				
**C9**	137.0	134.5	139.1				
**C10**	54.9	54.4	49.5				
**C11**	110.6	110.3	112.4	**H11**	6.50	6.73	6.68
**C12**	149.3	149.8	153.5				
**C13**	132.4	131.7	135.3				
**C14**	127.5	127.6	128.2	**H14**	7.29	7.32	6.85
**C15**	32.3	33.4	27.9	**H15**	3.21	3.23	3.18
**C16**	23.5	23.8	23.1	**H16 (CH_3)**	1.21	1.26	1.17
**C17**	23.9	23.9	23.2	**H17 (CH_3)**	1.19	1.31	1.17
**C18**	30.6	32.8	32.6	**H18 (CH_3)**	1.05	0.97	1.02
**C19**	24.7	22.8	21.5	**H19 (CH_3)**	0.87	0.86	0.93
**C20**	175.8	172.7	179				
**C21**	22.9	23.0	21.4				
**C22**	169.1	168.6	172.6	**H22 (CH_3)**	2.04	2.03	2.02
**MAD**	**2.5**	**2.4**			**0.11**	**0.10**	
**RMSD**	**2.9**	**3.0**			**0.20**	**0.14**	

Figure S163. Comparison of the observed ECD spectrum of 5 in ACN (black trace) with the calculated [CAM-B3LYP/PCM(ACN)/TZVP] ECD spectrum of the Boltzmann average of the 12 lowest-energy conformers identified for candidate 5b (2S,5S,10R) (blue solid line) and its enantiomer (2R,5R,10S) (red dashed line) of 5.

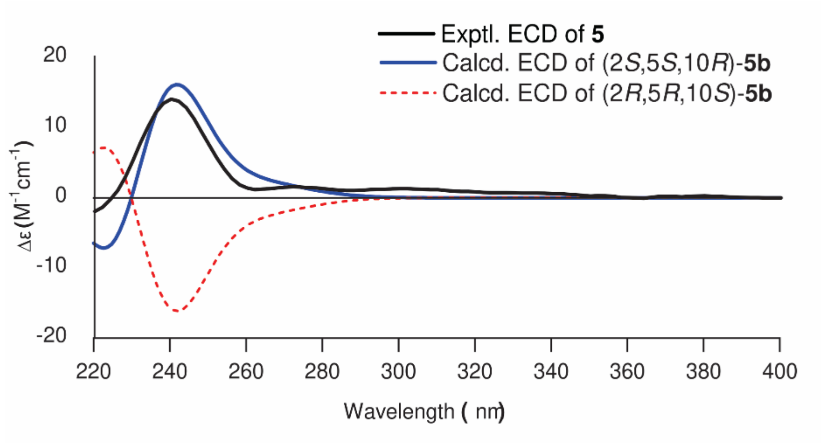

Figure S164. The 12 lowest energy conformers of candidate 5b at B3LYP/6-31G(d) level.

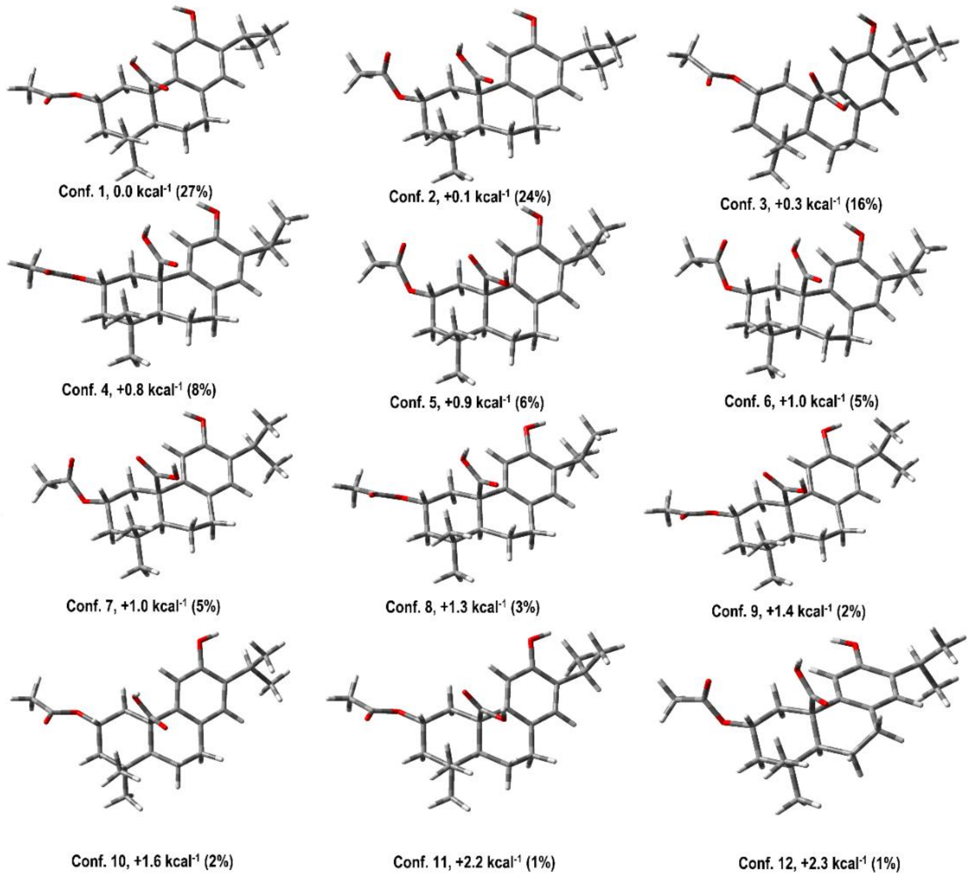

For candidate 5b, 54 conformers with relative energy within 10 kcal mol^{-1} of the lowest energy conformer were selected for further geometry optimized at the B3LYP/6-31G(d) level. 12 conformers were identified with relative energy < 2.5 kcal mol^{-1}, corresponding to more than 98.83% of the total Boltzmann distribution.

Figure S165. Results of DP4+ analysis of 6.

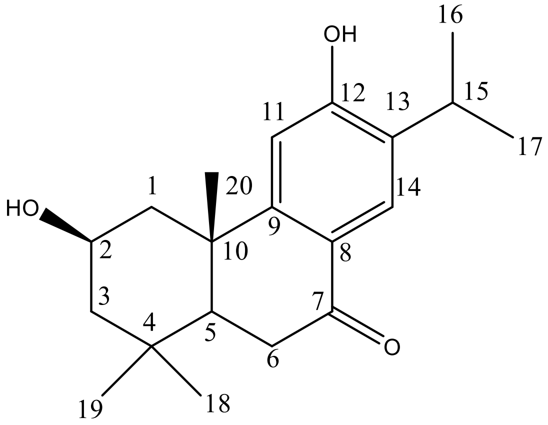

candidate 6a

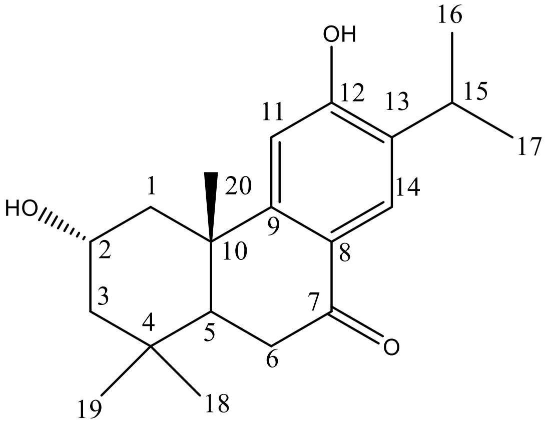

candidate 6b
DP4+: 100%

Table S9. Comparison of ^{13}C NMR (δ_{calc}) and ^{1}H NMR chemical shifts simulated for all candidates of 6 (GIAO-mPW1PW91/6-31+G(d,p)//B3LYP/6-31G(d)) with the experimental values of the isolated natural product.

Carbon	**6a** (δ_{calc})	**6b** (δ_{calc})	^{13}C RMN Exp.	Hydrogen	6a (δ_{calc})	6b (δ_{calc})	^{1}H RMN Exp.
**C1**	49.3	48.1	46.9	**H1α/H1β**	1.76/2.43	1.26/2.48	1.50/2.60
**C2**	67.4	67.1	65.7	**H2**	3.97	4.09	4.13
**C3**	46.4	51.4	50.4	**H3/H3’**	1.74/1.47	1.66/1.11	1.89/1.27
**C4**	36.9	39.0	35				
**C5**	47.3	51.0	48.9	**H5**	1.96	1.85	1.81
**C6**	39.7	38.6	35.9	**H6/H6’**	2.70/2.40	2.73/2.51	2.67/2.54
**C7**	194.4	194.5	198.1				
**C8**	123.4	123.1	124				
**C9**	154.0	154.0	155				
**C10**	45.8	45.4	39.6				
**C11**	110.8	108.4	109.6	**H11**	6.75	6.77	6.73
**C12**	156.5	156.3	159.3				
**C13**	130.4	130.5	133.6				
**C14**	127.0	127.9	127	**H14**	8.38	8.42	7.88
**C15**	33.0	32.3	26.9	**H15**	3.22	3.23	3.15
**C16**	23.4	23.8	22.5	**H16 (CH_3)**	1.24	1.23	1.21
**C17**	23.5	23.5	22.5	**H17 (CH_3)**	1.28	1.27	1.22
**C18**	31.1	33.3	32.7	**H18 (CH_3)**	0.89	0.86	0.96
**C19**	27.0	24.1	22.6	**H19 (CH_3)**	0.94	0.96	1.01
**C20**	30.6	25.7	24.4	**H20 (CH_3)**	1.38	1.35	1.23
**MAD**	**2.7**	**2.2**			**0.17**	**0.10**	
**RMSD**	**3.3**	**2.6**			**0.28**	**0.15**	

Figure S166. Comparison of the observed ECD spectrum of 6 in ACN (black trace) with the calculated [CAM-B3LYP/PCM(ACN)/TZVP] ECD spectrum of the Boltzmann average of the 12 lowest-energy conformers identified for candidate 6b (2S,5S,10R) (blue solid line) and its enantiomer (2R,5R,10S) (red dashed line) of 6.

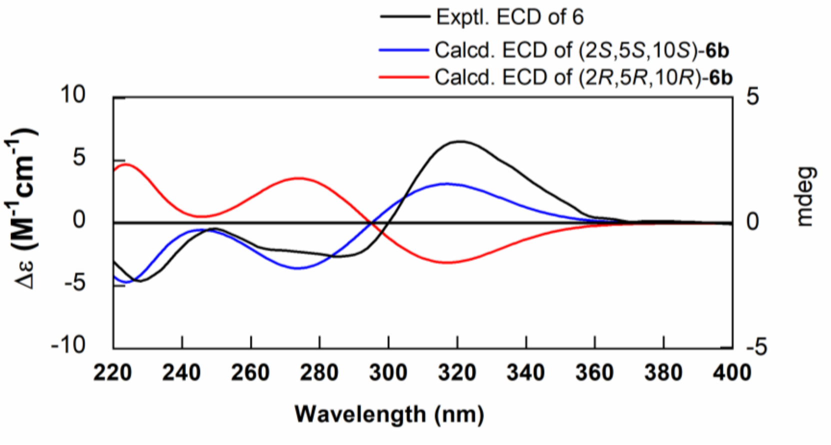

Figure S167. The 12 lowest energy conformers of candidate 6b at B3LYP/6-31G(d) level.

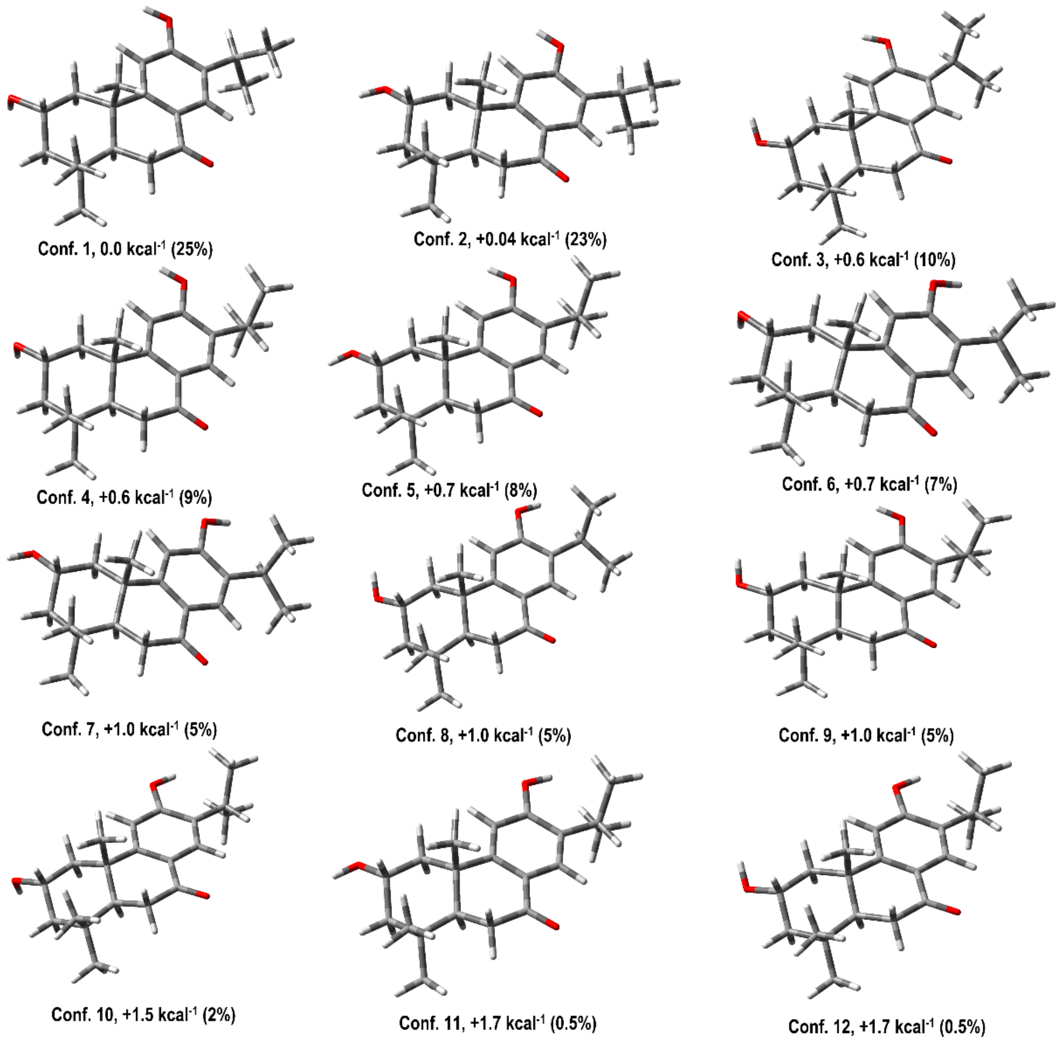

For candidate 6b, 12 conformers with relative energy within 10 kcal mol^{-1} of the lowest energy conformer were selected for further geometry optimized at the B3LYP/6-31G(d) level. 12 conformers were identified with relative energy < 2.5 kcal mol^{-1}, corresponding to more than 100% of the total Boltzmann distribution.

Figure S168. Results of DP4+ analysis of 7.

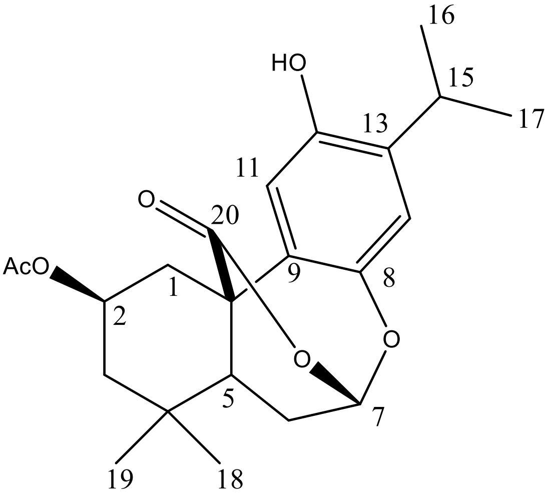

candidate 7a

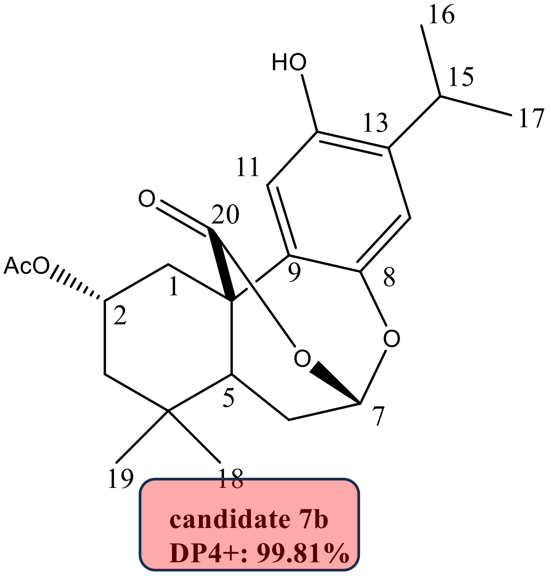

candidate 7b
DP4+: 99.81%

Table S10. Comparison of ^{13}C NMR (δ_{calc}) and ^{1}H NMR chemical shifts simulated for all candidates of 7 (GIAO-mPW1PW91/6-31+G(d,p)//B3LYP/6-31G(d)) with the experimental values of the isolated natural product.

Carbon	7a (δ_{calc})	7b (δ_{calc})	^{13}C RMN Exp.	Hydrogen	7a (δ_{calc})	7b (δ_{calc})	^{1}H RMN Exp.
C1	32.9	30.7	36.8	H1α/H1β	2.39/2.46	2.38/2.80	1.85/2.77
C2	71.9	71.4	67.9	H2	5.06	5.32	5.22
C3	42.8	41.0	45.3	H3/H3’	1.68/1.64	1.45/1.76	1.19/1.97
C4	39.5	37.6	36.3				
C5	51.4	49.8	50.6	H5	2.26	2.72	2.15
C6	32.4	33.6	26.7	H6/H6’	1.79/2.10	2.00/2.38	2.24/2.35
C7	98.3	98.7	95.4	H7	5.73	5.88	5.94
C8	145.4	145.8	145.8				
C9	122.9	123.6	121.7				
C10	53.0	53.2	47.3				
C11	109.7	109.8	112.3	H11	7.66	6.61	6.69
C12	147.4	146.8	148.1				
C13	134.6	134.1	136.7				
C14	117.9	116.9	117.3	H14	7.04	7.06	6.70
C15	31.7	33.0	26.8	H15	3.42	3.17	3.08
C16	23.6	23.3	22.3	H16 (CH_3)	1.20	1.23	1.18
C17	25.4	23.4	22.2	H17 (CH_3)	1.21	1.24	1.19
C18	31.0	32.2	30.8	H18 (CH_3)	0.76	0.79	0.86
C19	27.5	26.4	20.8	H19 (CH_3)	0.89	0.93	0.96

**C20**	169.0	171.1	170.9				
**C21**	22.7	22.9	21.3				
**C22**	169.9	169.4	169.9	**H22 (CH_3)**	2.03	1.97	2.05
**MAD**	**2.5**	**2.7**			**0.19**	**0.13**	
**RMSD**	**3.2**	**3.4**			**0.29**	**0.19**	

Figure S169. Comparison of the observed ECD spectrum of 7 in ACN (black trace) with the calculated [CAM-B3LYP/PCM(ACN)/TZVP] ECD spectrum of the Boltzmann average of the 16 lowest-energy conformers identified for candidate 7b (2S,5S,7S,10R) (blue solid line) and its enantiomer (2R,5R,7R,10S) (red dashed line) of 7.

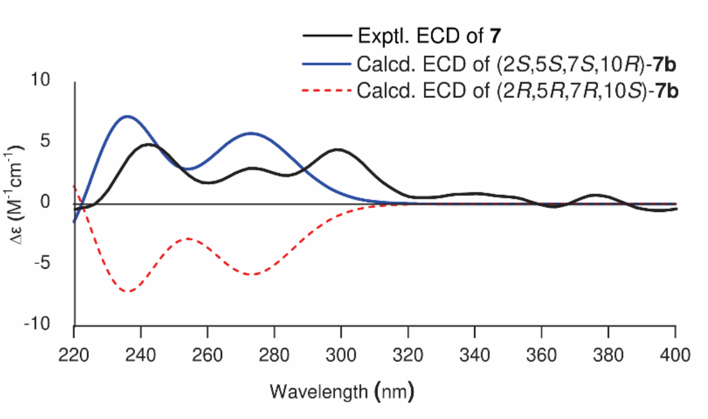

Figure S170. The 16 lowest energy conformers of candidate 7b at B3LYP/6-31G(d) level.

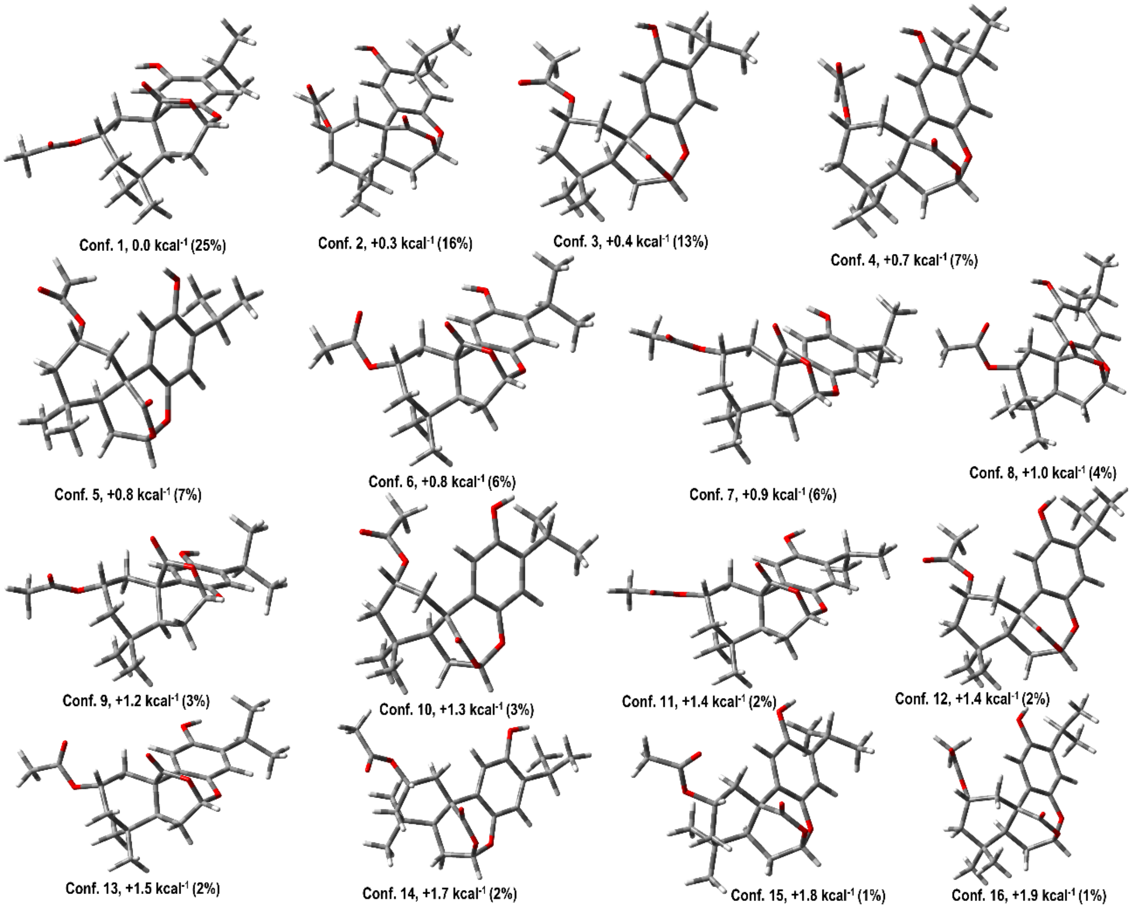

For candidate 7b, 26 conformers with relative energy within 10 kcal mol^{-1} of the lowest energy conformer were selected for further geometry optimized at the B3LYP/6-31G(d) level. 16 conformers were identified with relative energy < 2.0 kcal mol^{-1}, corresponding to more than 98.65% of the total Boltzmann distribution.

Figure S171. Results of DP4+ analysis of 8.

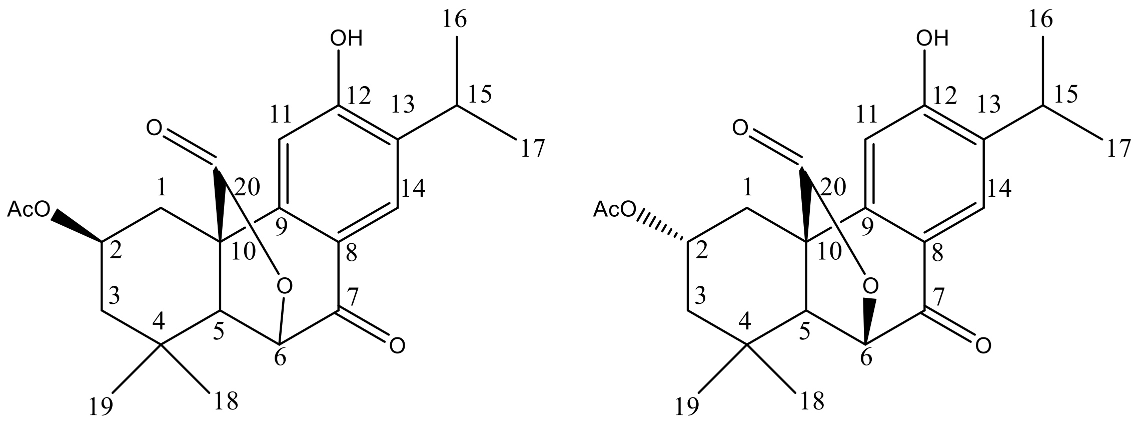

candidate 8a
DP4+: 23.08%

candidate 8b
DP4+: 76.92%

Table S11. Comparison of ^{13}C NMR (δ_{calc}) and ^{1}H NMR chemical shifts simulated for all candidates of 8 (GIAO-mPW1PW91/6-31+G(d,p)//B3LYP/6-31G(d)) with the experimental values of the isolated natural product.

Carbon	8a (δ_{calc})	8b (δ_{calc})	^{13}C RMN Exp.	Hidrogen	8a (δ_{calc})	8b (δ_{calc})	^{1}H RMN Exp.
**C1**	29.0	28.5	32	**H1α/H1β**	2.66/2.15	2.49/2.65	1.76/3.01
**C2**	70.7	71.3	67.3	**H2**	4.75	5.17	5.04
**C3**	41.9	42.0	43.6	**H3/H3'**	1.55/1.89	1.78/1.63	1.88/1.24
**C4**	37.0	35.4	34				
**C5**	64.3	63.7	59.5	**H5**	2.30	2.66	2.41
**C6**	83.7	84.4	81.4	**H6**	4.76	4.76	4.77
**C7**	187.5	188.3	189.1				
**C8**	121.6	120.9	121.8				
**C9**	142.3	142.7	143.8				
**C10**	54.8	54.0	49.2				
**C11**	108.2	107.7	111	**H11**	7.06	6.81	6.71
**C12**	157.6	157.7	159.1				
**C13**	134.0	133.2	136.1				
**C14**	129.7	129.4	128.2	**H14**	8.58	8.44	7.91
**C15**	31.6	33.2	27	**H15**	3.39	3.20	3.16
**C16**	23.4	23.6	22.3	**H16 (CH_3)**	1.29	1.27	1.23

**C17**	23.9	22.4	22.4	**H17 (CH_3)**	1.30	1.33	1.24
**C18**	31.0	32.1	31.4	**H18 (CH_3)**	1.11	1.07	1.07
**C19**	28.2	28.2	22.7	**H19 (CH_3)**	1.06	0.99	1.05
**C20**	175.6	175.9	176				
**C21**	22.8	22.8	21.4				
**C22**	170.2	169.3	170	**H22 (CH_3)**	2.08	1.80	2.06
**MAD**	**2.3**	**2.3**			**0.20**	**0.16**	
**RMSD**	**2.8**	**2.9**			**0.34**	**0.24**	

Figure S172. Comparison of the observed ECD spectrum of 8 in ACN (black trace) with the calculated [CAM-B3LYP/PCM(ACN)/TZVP] ECD spectrum of the Boltzmann average of the 19 lowest-energy conformers identified for candidate 8b (2S,5S,6S,10R) (blue solid line) and its enantiomer (2R,5R,6R,10S) (red dashed line) of 8.

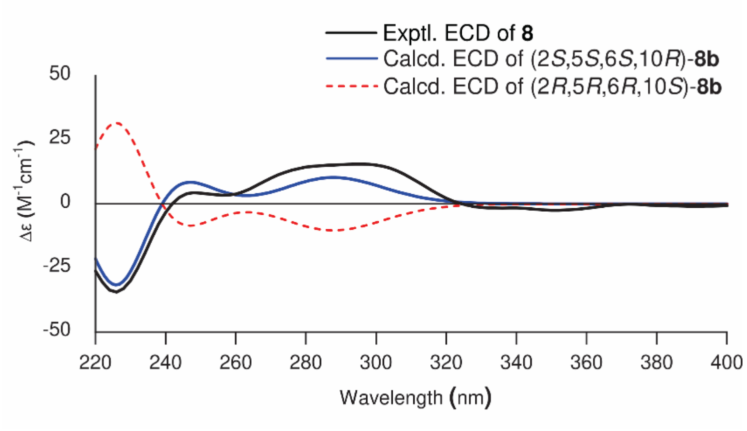

Figure S173. The 19 lowest energy conformers of candidate 8b at B3LYP/6-31G(d) level.

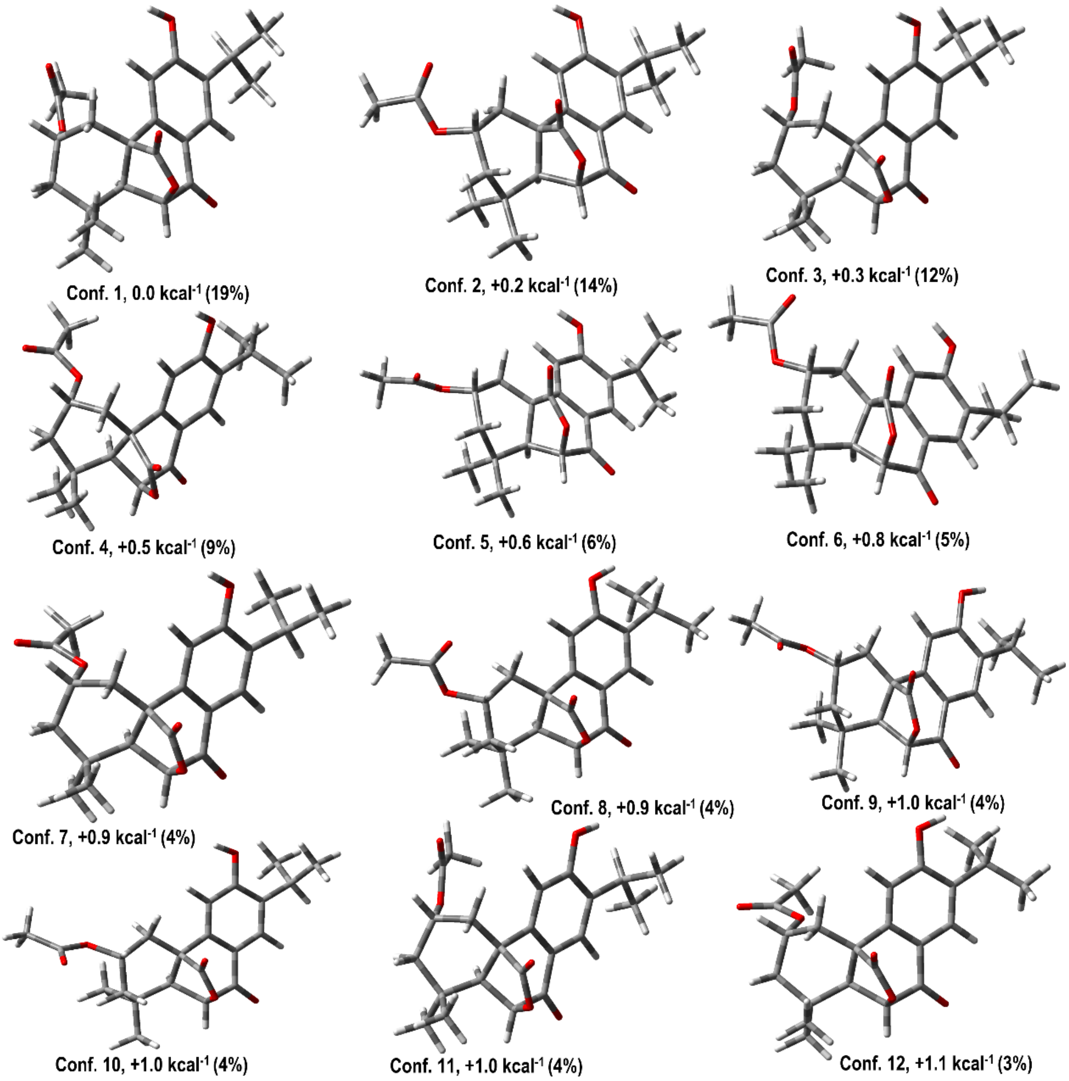

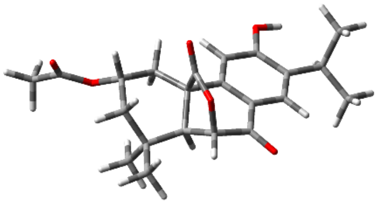

Conf. 13, +1.3 kcal^{-1} (2%)

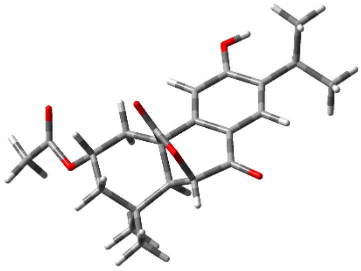

Conf. 14, +1.3 kcal^{-1} (2%)

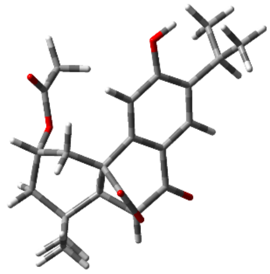

Conf. 15, +1.4 kcal^{-1} (2%)

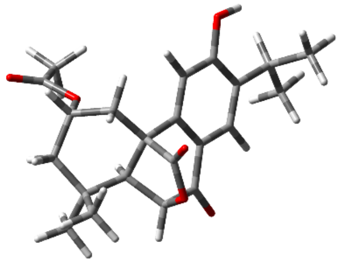

Conf. 16, +1.4 kcal^{-1} (2%)

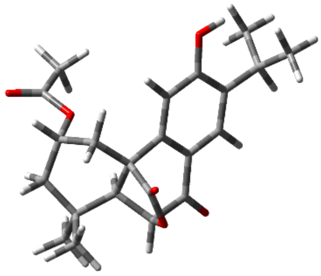

Conf. 17, +1.4 kcal^{-1} (2%)

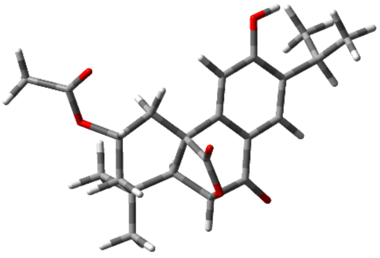

Conf. 18, +1.7 kcal^{-1} (1%)

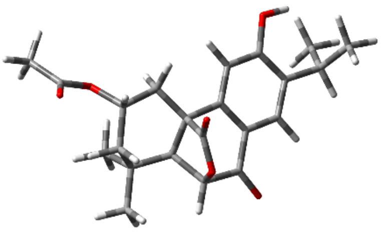

Conf. 19, +1.8 kcal^{-1} (1%)

For candidate 8b, 23 conformers with relative energy within 10 kcal mol^{-1} of the lowest energy conformer were selected for further geometry optimized at the B3LYP/6-31G(d) level. 19 conformers were identified with relative energy < 2.5 kcal mol^{-1}, corresponding to more than 99.99% of the total Boltzmann distribution.

Figure S174. Results of DP4+ analysis of 9.

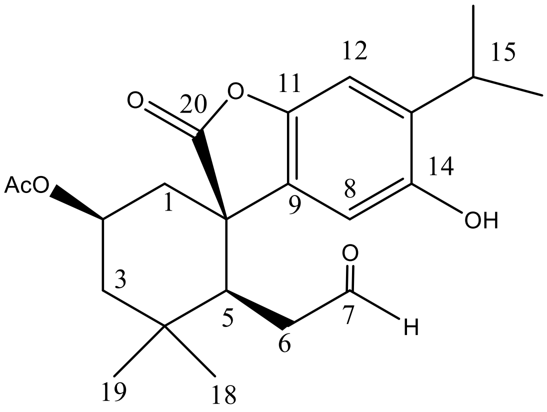

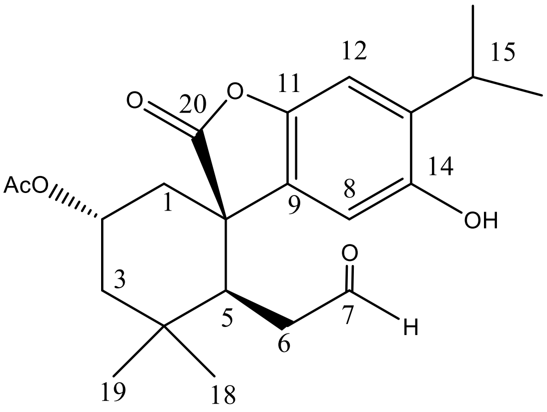

candidate 9a
DP4+: 0%

candidate 9b
DP4+: 100%

Table S12. Comparison of ^{13}C NMR (δ_{calc}) and ^{1}H NMR chemical shifts simulated for all candidates of 9 (GIAO-mPW1PW91/6-31+G(d,p)//B3LYP/6-31G(d)) with the experimental values of the isolated natural product.

Carbon	9a (δ_{calc})	9b (δ_{calc})	^{13}C RMN Exp.	Hidrogen	9a (δ_{calc})	9b (δ_{calc})	^{1}H RMN Exp.
**C1**	41.0	39.7	40	**H1α/H1β**	1.80/2.39	2.64/2.06	1.71/2.23
**C2**	71.0	73.1	66.4	**H2**	5.15	5.11	5.50
**C3**	44.1	42.8	46.1	**H3α/H3β**	1.92/1.78	2.25/2.20	2.03/1.49
**C4**	37.5	38.9	36.3				
**C5**	42.8	46.8	45.7	**H5**	2.95	2.72	2.34
**C6**	46.2	45.8	41.8	**H6/H6'**	2.87/1.80	2.52/1.81	2.46/1.94
**C7**	199.2	201.1	199.5	**H7**	9.79	9.73	9.19
**C8**	108.9	108.0	110.2	**H8**	6.92	6.76	6.57
**C9**	129.1	127.6	128.3				
**C10**	57.1	58.8	53.1				
**C11**	144.0	144.7	146.6				
**C12**	109.5	108.3	108.6	**H12**	7.02	7.24	6.86
**C13**	133.0	134.7	137.1				
**C14**	149.4	148.4	150.2				
**C15**	37.6	32.2	27.4	**H15**	2.91	3.32	3.16
**C16**	22.5	22.4	22.5	**H16 (CH_3)**	1.32	1.25	1.20
**C17**	21.7	24.7	22.8	**H17 (CH_3)**	1.36	1.17	1.18
**C18**	33.6	34.2	33.3	**H18 (CH_3)**	0.66	1.06	0.94
**C19**	25.4	25.3	22	**H19 (CH_3)**	1.47	1.08	1.23
**C20**	177.8	178.2	177.9				
**C21**	22.7	23.4	21.4				
**C22**	169.8	169.8	170.3	**H22 (CH_3)**	2.06	2.05	1.99
**MAD**	**2.2**	**2.2**			**0.23**	**0.20**	
**RMSD**	**3.1**	**2.8**			**0.27**	**0.30**	

Figure S175. Comparison of the observed ECD spectrum of 9 in ACN (black trace) with the calculated [CAM-B3LYP/PCM(ACN)/TZVP] ECD spectrum of the Boltzmann average of the 18 lowest-energy conformers identified for candidate 9b (2S,5S,10R) (blue solid line) and its enantiomer (2R,5R, 10S) (red dashed line) of 9.

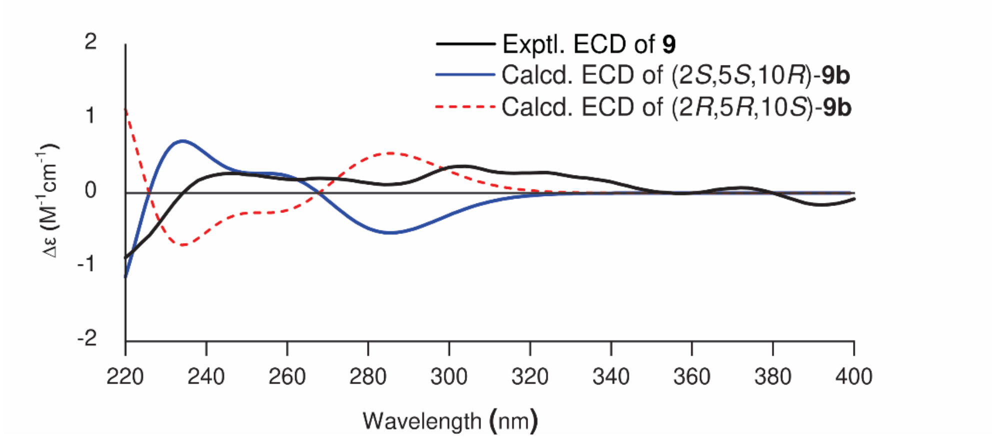

Figure S176. The 18 lowest energy conformers of candidate 9b at B3LYP/6-31G(d) level.

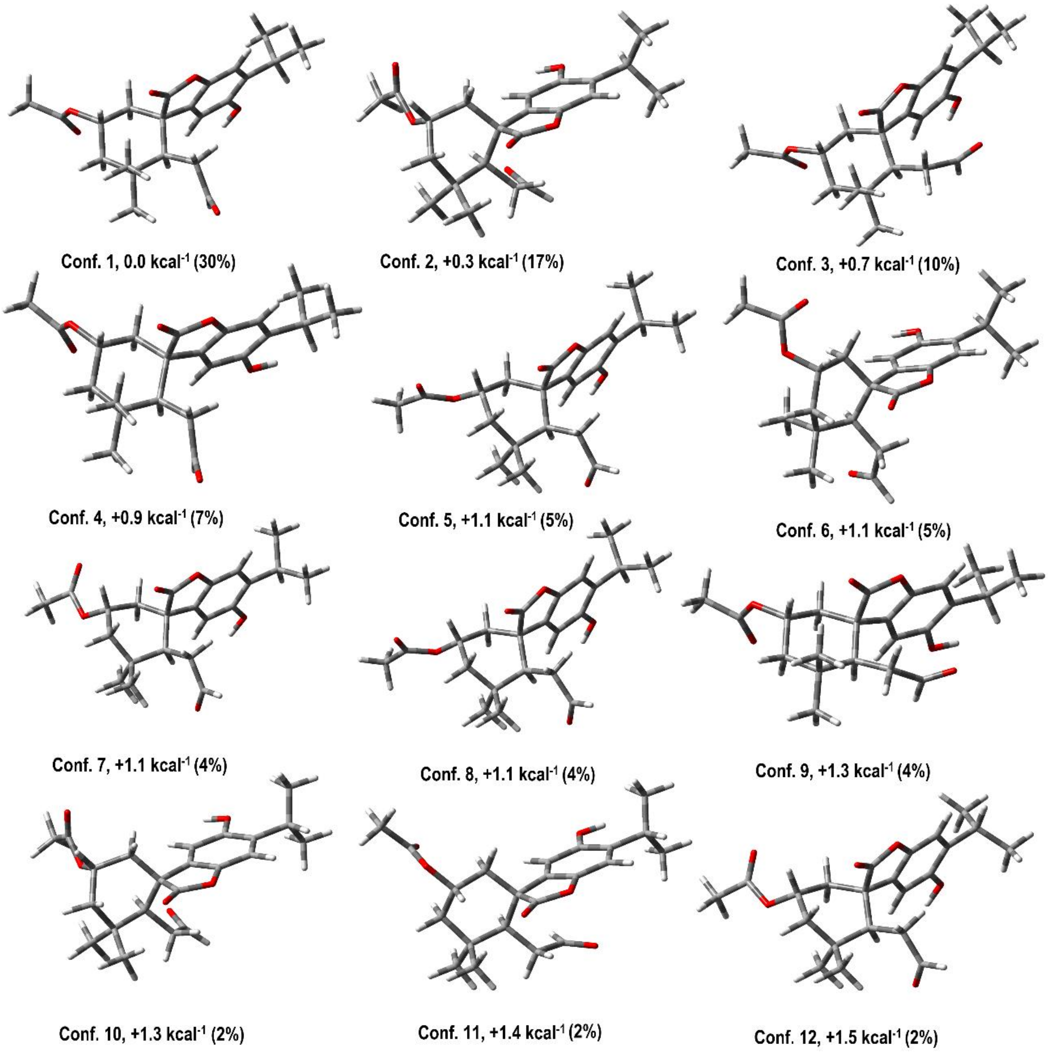

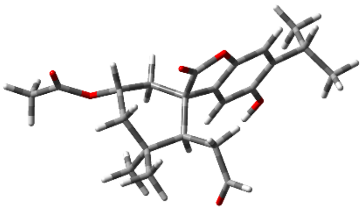

Conf. 13, +1.7 kcal^{-1} (2%)

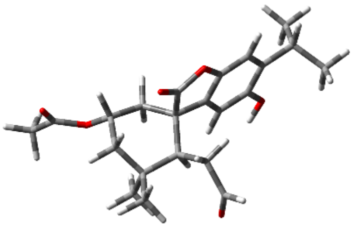

Conf. 14, +1.8 kcal^{-1} (2%)

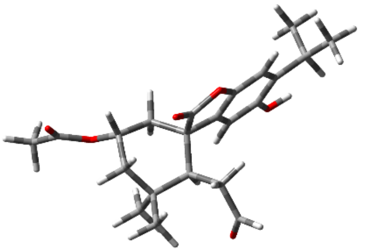

Conf. 15, +2.1 kcal^{-1} (1%)

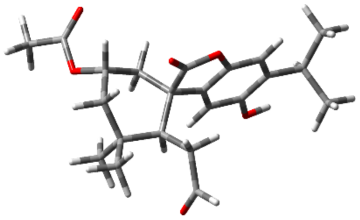

Conf. 16, +2.1 kcal^{-1} (1%)

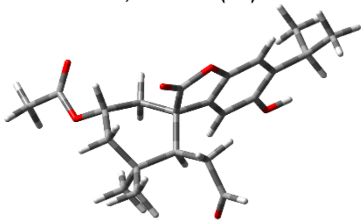

Conf. 17, +2.3 kcal^{-1} (1%)

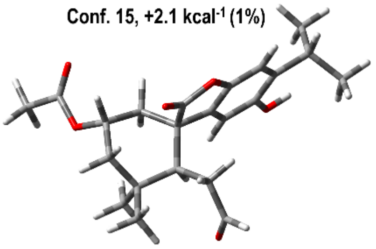

Conf. 18, +2.5 kcal^{-1} (1%)

For candidate 9b, 99 conformers with relative energy within 10 kcal mol^{-1} of the lowest energy conformer were selected for further geometry optimized at the B3LYP/6-31G(d) level. 18 conformers were identified with relative energy < 2.5 kcal mol^{-1}, corresponding to more than 98.34% of the total Boltzmann distribution.
